# Supplementary material for: Machine learning uncovers independently regulated modules in the Bacillus subtilis transcriptome
Source: Nat Commun. 2020 Dec 11;11:6338. doi: 10.1038/s41467-020-20153-9 (PMC7732839; doi:10.1038/s41467-020-20153-9)
Supplement: Supplementary file 1 — Supplementary Information [file 41467_2020_20153_MOESM1_ESM.pdf]

Supplementary Information

Machine learning uncovers independently regulated modules in the *Bacillus subtilis* transcriptome

Authors: Kevin Rychel, Anand V. Sastry, Bernhard O. Palsson

Supplementary Notes 2

Supplementary Note 1: The MalR iModulon perfectly captures the corresponding regulon. 2

Supplementary Note 2: Amino acid metabolism iModulons exhibit generally expected behavior. 2

Supplementary Note 3: The arginine synthesis (AhrC) iModulon. 2

Supplementary Note 4: The CcpA regulon is captured by two iModulons. 2

Supplementary Note 5: The correlations between iModulon activity and regulator expression. 3

Supplementary Note 6: Two Uncharacterized iModulons may have important functions. 3

Supplementary Note 7: Motif discovery reveals consensus sequences for 29 iModulons. 4

Supplementary Note 8: Precision and recall improve as TRN annotations become more complete. 4

Supplementary Methods 4

Obtaining robust independent components. 4

iModulon Threshold Determination. 5

Transcriptional Regulatory Network Annotation. 5

Explained Variance Calculation. 5

Correlation Analysis. 6

Protein Homology. 6

Motif Discovery. 6

Supplementary Figures 7

Supplementary Figure 1: Overview of the data 7

Supplementary Figure 2: Network Analysis 8

Supplementary Figure 3: The MalR iModulon 9

Supplementary Figure 4: Supplementary amino acid iModulon plots 10

Supplementary Figure 5: Graphical representations of hypotheses. 11

Supplementary Figure 6: The CcpA iModulons 12

Supplementary Figure 7: Correlation between iModulon activity and regulator expression 13

Supplementary Figure 8: The genes in sporulation iModulons 14

Supplementary Figure 9: The iModulons with increased thresholds 15

Supplementary References 16

Supplementary Notes

Supplementary Note 1: The MalR iModulon perfectly captures the corresponding regulon.
The dataset contained carbon source transitions from glucose to malate and vice versa[1], which provided rich data for understanding the transcriptomics of malate metabolism. The MalR regulon is composed of 4 genes which are activated by extracellular malate and not directly affected by master regulators such as CcpA[2,3]. ICA produced an iModulon composed of the same 4 genes (Supplementary Figure 2a). The iModulon activity rapidly increased upon malate addition to glucose media and declined over the course of glucose addition to malate media (Supplementary Figure 2b), as would be expected. Six other iModulons also have perfect overlap with their corresponding regulon annotations; they are mostly associated with specific carbon sources (Supplementary Figure 1c, Supplementary Data 7).

Supplementary Note 2: Amino acid metabolism iModulons exhibit generally expected behavior.
Eight iModulons function in amino acid metabolism, either fulfilling synthesis (n = 6) or utilization (n = 2) roles. These iModulons exhibit expected general patterns, but some of them are highlighted in these results because they have unpredicted activity levels under stress or specific lifestyles (See Supplementary Data 9 for a full list of expected and unexpected activity levels for all characterized iModulons). Generally, amino acid synthesis iModulons are less active in rich media compared to minimal media as a result of the exogenous amino acid supply. Over the course of growth in minimal media, their activity tends to decrease as intracellular amino acid stores are built up and protein synthesis for growth declines (Supplementary Figure 3a). The opposite is true of the two utilization iModulons (HutP, RocR/PutR).

Supplementary Note 3: The arginine synthesis (AhrC) iModulon.
The arginine synthesis iModulon provides two interesting insights. Its genes include *argGHCJBDF* and *carAB*, which are known to be repressed in the presence of arginine by AhrC[4]. The first insight is that it also contains *artPQR* (Supplementary Figure 3b), which are arginine importers not known to be transcriptionally regulated by AhrC – given that they are part of the same independent signal in the transcriptome, they likely share this regulation. In addition, the iModulon was unexpectedly downregulated in salt shock, but not after growth in salt (Supplementary Figure 3c); this has not been explored in previous studies. Here, the putative mechanism is less clear. It may involve the production of osmoprotective solutes such as proline[5], which might perturb metabolic networks in such a way that arginine concentrations increase and then downregulate these genes. After proline stores have been established, arginine concentrations appear to be restored. There is also evidence of a proline/arginine metabolic link in another iModulon: the RocR/PutR joint iModulon combines the utilization of both amino acids into one signal. Exploration of this relationship may help to understand broader changes in amino acid metabolism and its regulation under stress conditions.

Supplementary Note 4: The CcpA regulon is captured by two iModulons.
The CcpA iModulons regulate carbon catabolites in different phases of growth (Supplementary Figure 5), which may suggest divergent preferences for carbon catabolites determined by growth phase and starvation state; the same catabolites that are preferred during exponential growth are preferred during germination.

Supplementary Note 5: The correlations between iModulon activity and regulator expression.

Regulatory proteins are often subject to ligand binding or kinase activity, which switches them between active and inactive states. Therefore, the gene expression of a given transcription factor does not usually correlate with the expression of its targets; this is the case with MalR, which is activated through phosphorylation by MalK only when malate is present[3]. Since iModulons combine co-regulated gene expression into easy-to-evaluate activity levels, we can attempt to correlate regulator expression with iModulon activity (Supplementary Methods). As expected, many of these correlations are low (see Supplementary Data 7). For example, MalR activation occurs through a post-transcriptional binary switch, so there is no correlation between MalR gene transcription and iModulon activity (Supplementary Figure 6a). One major exception to this is the sigma factors, which are often only regulated at the expression level. In these cases, we observe much higher correlations between expression and activity, such as with the motility sigma factor, SigD (Supplementary Figure 6b). High correlations are also observed when the regulator undergoes positive feedback, in which case it is a member of its own iModulon (Supplementary Figure 6c).

The Thi-box is a riboswitch that is conserved in all domains of life and regulates the expression of genes for thiamine synthesis and transport. In *B. subtilis*, this sequence is upstream of a transcriptional terminator, which it deactivates in the absence of thiamine[6,7]. We would therefore expect the Thi-box sequence to be constitutively expressed, and its downstream genes to respond to thiamine levels – there would be no correlation between Thi-box RNA expression and the activity levels of its genes. Instead, this relationship had a unique shape which was consistent for all 5 Thi-boxes (Supplementary Figure 6C). We believe that this may be explained by differential degradation of the short Thi-box RNA sequence. Under minimal media conditions, the thi-box sequence does not bind thiamine, so RNA polymerase reads through it and produces a long, relatively stable RNA molecule, which is measured as both high thi-box expression and high thi-box iModulon activity. Under rich conditions, the binding of thiamine terminates transcription, preventing thi-box iModulon activity and producing a short, less stable RNA molecule. Interestingly, this short sequence may be degraded quickly in flasks (evidenced by a lack of apparent Thi-box expression) but appears to remain in biofilms for long enough that it could be measured in this experiment. Little is known about differential RNA degradation in biofilms, but this result motivates further study of that phenomenon.

Supplementary Note 6: Two Uncharacterized iModulons may have important functions.

The *ndhF-ybcCFHI* operon was identified as its own iModulon (Main Fig. 6a). *ndhF* is known to be a subunit of NADH dehydrogenase, but the *ybc* genes have not been characterized at all. Peptide homology (Supplementary Methods) suggests that *ybcC* may form a protein that binds to ndhF, and that *ybcF* may be carbonic anhydrase. The activity levels of this group of genes demonstrate very strong activation under heat shock, as well as repression during cold shock and unusually high germination activity. Perhaps this is a new category of heat-responsive genes; since heat shock is a complex response[8], a small operon like this may have been overlooked in previous studies. We can hypothesize mechanisms through which this operon might benefit the cell: heat shock should upregulate *ndhF* to help to power the heat stress response, *ybcC* might be a chaperone for *ndhF*, and maybe *ybcF* assists in raising the pH after a temperature increase lowers it. We propose gene knockout experiments to validate that these genes play a role in the survival of heat shock.

Another uncharacterized iModulon is the *yrkEFHI* operon. None of these genes have been characterized, but two of them are putative sulfur carriers, and one is a putative sulfurtransferase. This iModulon exhibits consistently low activity except in the two conditions with ten or fifteen minutes of diamide exposure (Main Fig. 6b). Since diamide oxidizes thiols to disulfides, it would make sense for sulfur carriers to be necessary in this condition. Future experiments can be performed to confirm this reasoning and identify transcriptional regulatory mechanisms.

Supplementary Note 7: Motif discovery reveals consensus sequences for 29 iModulons.
The results of a motif search (Supplementary Methods) are shown in Supplementary Data 10 and graphically in Supplementary Data 6. Though the TnrA/PucR iModulon appeared to be a combination of regulons, a conserved motif was observed upstream of all genes, which may suggest that this grouping is co-regulated as opposed to co-stimulated. 13 of the 29 identified motifs match a motif in the PRODORIC database[20], which is the binding site database with the best coverage of Bacillus subtilis. Of the 13 matched sequences, 7 matched expectations based on known binding sites and enriched regulators. The Fur iModulon matched with the Fur binding site from E. coli since the Bacillus sequence was not in the database, suggesting that Fur recognizes a highly conserved binding motif. Interestingly, the CymR iModulon and three sporulation iModulons had motifs which matched global, virulence, or stress response regulators in other organisms.

Supplementary Note 8: Precision and recall improve as TRN annotations become more complete.
By comparing ten years of legacy transcriptional regulatory networks on the Abasy database[9] against our gene sets, we could determine whether the iModulons are highly sensitive to the completeness of TRN annotations. Over time, these annotations become more complete, which leads to an increase in performance metrics of the iModulon enrichments (Supplementary Figure S2d). Our TRN included regulation of untranslated regions, which improved performance compared to all legacy TRNs.

Supplementary Methods

Obtaining robust independent components.
We processed the quality-checked, centered data (**X**) with the Scikit-Learn (v0.19.0) implementation of FastICA[11] using 100 iterations, a convergence tolerance of 10^{-7}, log(cosh(*x*)) as the contrast function, and parallel search. We calculated enough components to reconstruct 99% of the variance as determined by PCA.

After 100 iterations of ICA, the **M** matrices were pooled and clustered with Scikit-Learn DBSCAN[11] (epsilon = 0.1, minimum size = 50) in order to find robust components which appear in each random restart. Since identical components can have opposite signs, we defined distance for this algorithm using a sign-agnostic method:

$$d_{x,y} = 1 - |\rho_{x,y}| \qquad (1)$$

where $d_{x,y}$ is the distance and $\rho_{x,y}$ is the Pearson correlation between components x and y. Components belong to a cluster if $d_{x,y} < 0.1$ with all other components in the cluster. To ensure repeatability, all signs in a cluster were inverted if necessary so that the highest weighted gene would have a positive sign. The centroids of each cluster defined the weightings in **M** and were used to calculate **A**.

This process was repeated 100 times (for a total of 10,000 ICA runs), and components that did not arise in every run were discarded. The result contained 83 robust components.

iModulon Threshold Determination.
The distribution of **M** matrix weights of each gene for a given component consists of a large number of near-zero values along with a small number of genes at the tails. To define the gene set of the iModulon, we need to choose a threshold value that separates the normally distributed near-zero genes from the more meaningful, non-gaussian tails. To do so, we used Scikit Learn's implementation of the D'Agostino K^2 test, which quantifies the skew and kurtosis of the distribution as a measure of gaussianity[11,12]. We iteratively remove the gene with the highest absolute value in the component and calculate the K^2 value until the value falls below a K^2 cutoff value (1300). All genes that were removed are members of the iModulon gene set, and the non-removed genes are sufficiently normally distributed around zero to be considered noise. In all cases discussed in this study, all member genes have positive weights, which allows for easier representation as a set of genes and a simple interpretation of activity.

The cutoff value of 1300 was determined by a sensitivity analysis. Over a range of cutoffs (200 - 2200), we computed the top regulator enrichments and F1 scores as described in the main text (Methods, Regulator Enrichment). The cutoff with the highest mean F1 score was selected. In seven cases (Supplementary Data 3), this cutoff was not appropriate because it removed all genes from the iModulon (5/7) or captured non-important genes (2/7), so the threshold was adjusted to 500 or increased slightly as necessary.

The two cases (MalR and Rex) in which the threshold was increased are shown in Supplementary Figure 8. In these cases, we see the tails of the distribution starting a bit higher than their computed thresholds, and the full tail corresponds to a regulon. For this reason, it was appropriate to raise the thresholds in these cases.

Transcriptional Regulatory Network Annotation.
TRN annotations were originally downloaded from the SubtiWiki database[9]. The iModulons include non-coding RNAs which are left out of those annotations, but gene annotations for those RNAs contained descriptions such as "5' UTR of xxxX". Using these descriptions and the annotated genome locations, we added regulatory connections between the regulators of genes and their corresponding untranslated regions. This TRN was then used to compute precision and recall for all iModulons.

We sought to determine the sensitivity of those scores to the availability and completeness of TRN annotations. To do so, we downloaded legacy TRNs from the Abasy database[13] and computed average precision and recall scores for each regulated iModulon using those annotations.

Explained Variance Calculation.
We reconstructed the dataset using only the iModulons. To do this, we first define a binary matrix $\mathbf{M_{bin}}$, [genes]x[iModulons] (Supplementary Data 5), whose elements are 1 if the row's corresponding gene is in the column's corresponding iModulon, 0 otherwise. We compute **M'** using element-wise multiplication $\mathbf{M}*\mathbf{M_{bin}}$, which removes the effect of all non-significant genes from **M**. We then define **X'** = **M'**$*$**A**, using matrix multiplication to reconstruct the data. We used the scikit-learn explained variance score function[11] to obtain a total explained variance of 72% (Supplementary Figure 1b).

Correlation Analysis.

iModulon activities were compared to regulator gene expression values using scatter plots and a best fit line. The best fit lines are composed of two parts:

$$y = a * c + b \text{ if } x < c \quad (2)$$

$$y = a * x + b \text{ if } x \geq c \quad (3)$$

where x is the expression level of the regulator, y is the iModulon activity level, and a, b, and c are fitting parameters determined by the optimize.curve_fit function in the Python sciPy package[14]. The flat part of the curve, defined by the first equation, represents the minimal activity level required before a correlation is observed; it does not exist for all correlations. The adjusted R^2 value accounts for the k parameters used ($k = 2$ or $k = 3$ depending on whether or not the line required both parts) based on the following equation:

$$R_{adj}^2 = 1 - (1 - R^2)(n - 1)/(n - k - 1) \quad (4)$$

where R^2 is the coefficient of determination.

Protein Homology.

Potential functions of uncharacterized genes were identified by hhblits[15]. Gene sequences were obtained using the genome sequence from GenBank Accession AL009126.3[16]. Gene locations were found in the *Subti*Wiki gene annotations[13], and translated into peptides using Bio Entrez[17]. Peptide sequences were compared to those in the Uniclust30 database[18] with hhblits. This produced detailed output for each gene, from which a homology probability of 95% or above was required for all stated conclusions.

Motif Discovery.

The motifs (Supplementary Data 10) were identified by searching upstream sequences using MEME[19]. Genes were grouped into operons, and only iModulons containing four or more operons were searched. We searched for up to 5 motifs for all widths between 6 and 40 base pairs (bp) in the region from -600 to +100 bp from the transcription start site. We used an E-value threshold of 10^{-3}. When multiple motifs surpassed the E-value threshold, we selected the one which occurred in the upstream sequences of the most operons.

All found motifs were compared against the PRODORIC database[20] using Tomtom[21], with an E-value threshold of 0.01 and allowing incomplete matches.

Supplementary Figures

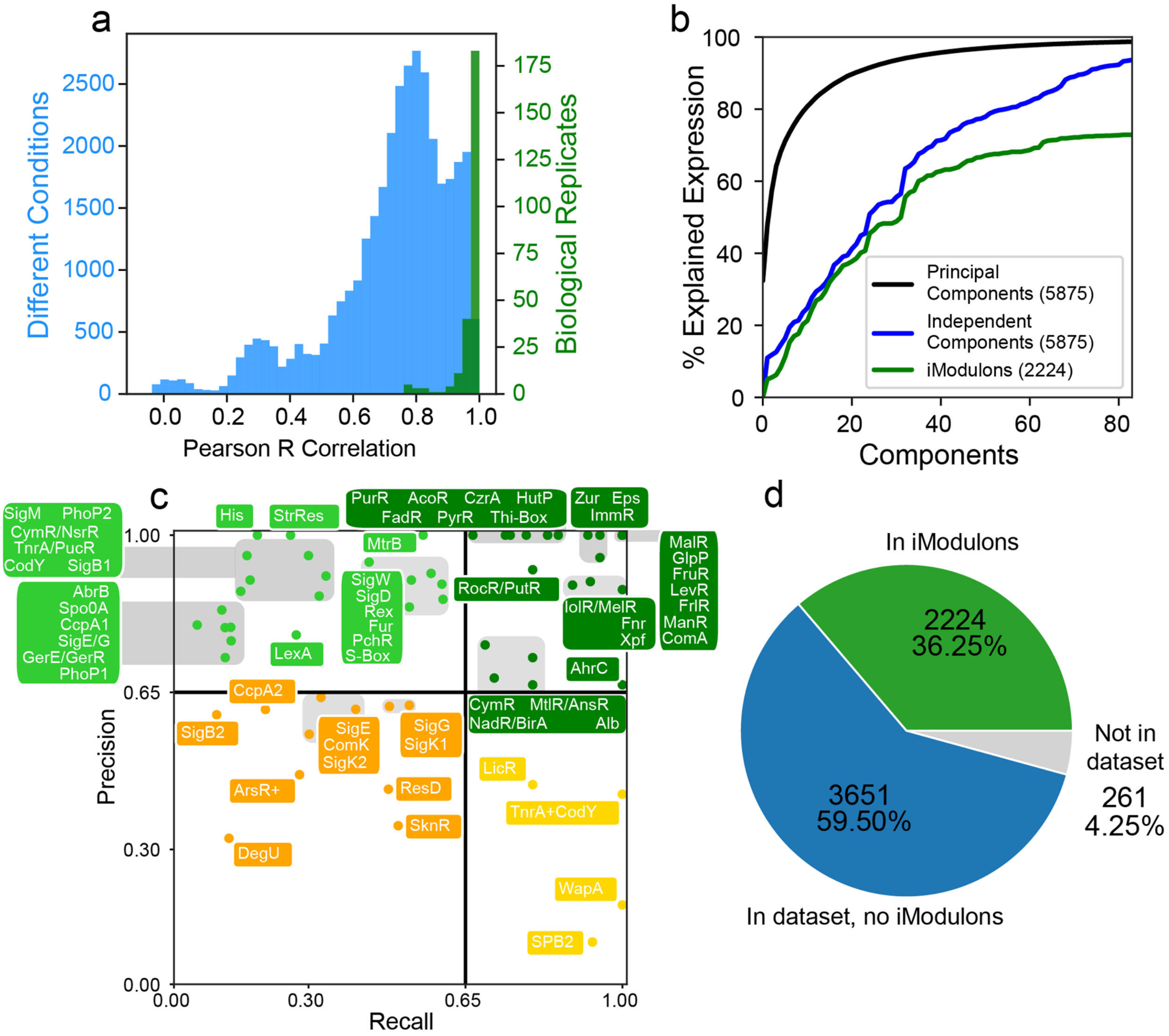

Supplementary Figure 1: Overview of the data

a. Histogram of Pearson R correlations in gene expression between different conditions (blue) and biological replicates (green). High correlation between replicates indicates high quality data. Three noisy samples were removed from the initial dataset to enforce this behavior. **b.** Variance in the expression data using PCA, the full **M** and **A** matrices (independent components) and the thresholded **M** matrix (iModulons). Parentheses in the legend indicate the number of genes used for the backprojection of the original data. While PCA explains more expression quantitatively, the components it obtains are less biologically interpretable than those of ICA. Thresholding the independent components to obtain iModulons causes a loss of only ~20% of the explained variance, which indicates that most of the variance captured by ICA is in the iModulon member genes. **c.** Labeled version of Main Fig. 1c, with iModulon short names shown near their precision/recall coordinates. When space required that labels be grouped together, the relative positions of the label and point were maintained as best as possible (e.g. vertically listed names correspond to each point in order of decreasing precision). **d.** Pie chart of genomic

feature presence, using the reference gene list available from SubtiWiki. The excluded 261 features (gray) were not included because they are rRNAs, had very low expression, or were not known at the time the dataset was generated.

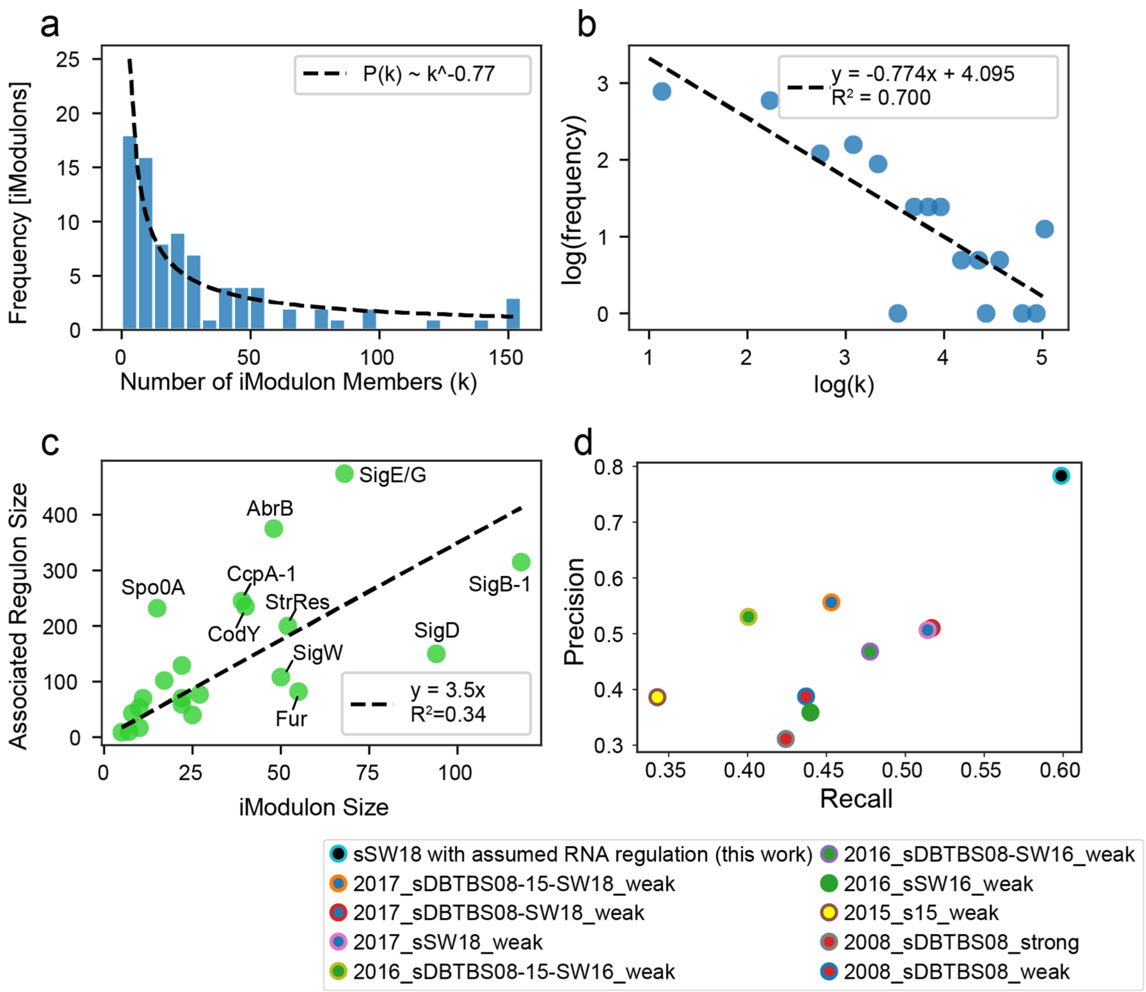

Supplementary Figure 2: Network Analysis

a. Histogram of iModulon sizes, with a power law fit shown in dotted lines. **b.** Logarithm of (a), showing the best fit line used to produce the dotted line and its R^2 value, 0.70. Note that the slope of this line is lower than that of typical regulatory networks, which usually have $P(k) \sim k^{-2}$ [citation[22]]. We used 25 bins for this analysis. **c.** Scatter plot of subset iModulon size versus the size of associated regulons. A slight correlation is observed, with the typical global regulon being 3.5 times the size of its associated iModulon. iModulons above this line, such as Spo0A, capture relatively small subsets whereas iModulons below capture relatively larger subsets. Differences in recall may be due to differences in binding strength distributions for the regulators and their target genes, or be artifacts of the condition space in the selected dataset. **d.** Scatter plot of mean precision and recall for all regulated iModulons using different TRN annotations. The center color of each dot corresponds to the year of the TRN. The legend contains the year, database source, and lowest evidence requirement (weak or strong) for this database (see Abasy[13]). Scores are sensitive to the level of annotation, but improve as the annotations become more complete.

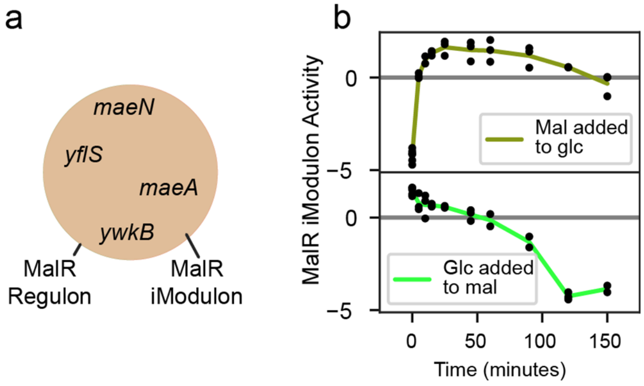

Supplementary Figure 3: The MalR iModulon
a. Venn diagram of the MalR iModulon and MalR regulon. Precision and recall are 100%. **b.** MalR iModulon activity over time for malate addition to glucose media (top) and glucose addition to malate media (bottom). Activity falls slowly after glucose addition (from approximately 0 to -5) and rises rapidly upon malate addition (from -5 to approximately 0).

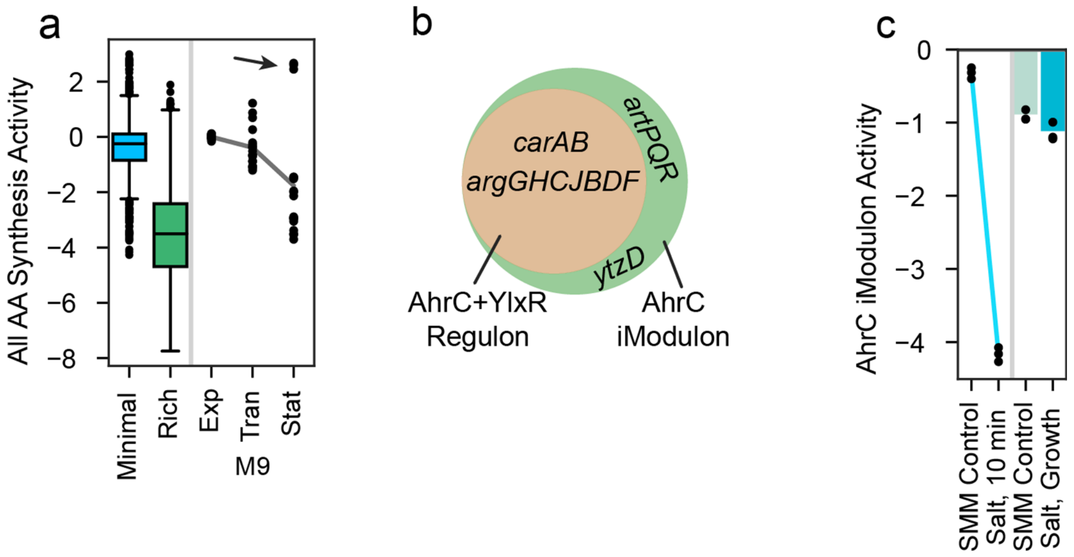

Supplementary Figure 4: Supplementary amino acid iModulon plots

a. Box plot and time course of all 6 amino acid synthesis iModulons in media with casamino acids (n=139 samples), without casamino acids (n=86 samples), and over three growth phases in M9 media. 'Exp', 'Tran' and 'Stat' refer to exponential (n=3 samples), transition (n=3 samples), and stationary phase (n=3 samples), respectively. Box plots were generated using the default matplotlib behavior, in which the center line represents the median (Q2), box bounds represent the upper (Q3) and lower (Q1) quartiles, whiskers represent either the extrema or 1.5*[Q3-Q1] (whichever creates a shorter whisker), and data outside the whiskers are plotted as individual points. Activity follows expected trends based on amino acid stimulus presence. Outliers (arrow) for M9 growth are from the CodY iModulon, the only synthesis iModulon which increased over this time course. **b-c.** The arginine synthesis (AhrC) iModulon. **b.** Venn diagram of the arginine synthesis (AhrC) iModulon and regulon; the regulon contains additional arginine-related genes (*artPQR* and *ytzD*) that are not known to be regulated by AhrC. **c.** AhrC iModulon activity in osmotic stress conditions. This iModulon is surprisingly downregulated by salt shock. N = 3 samples for all conditions shown.

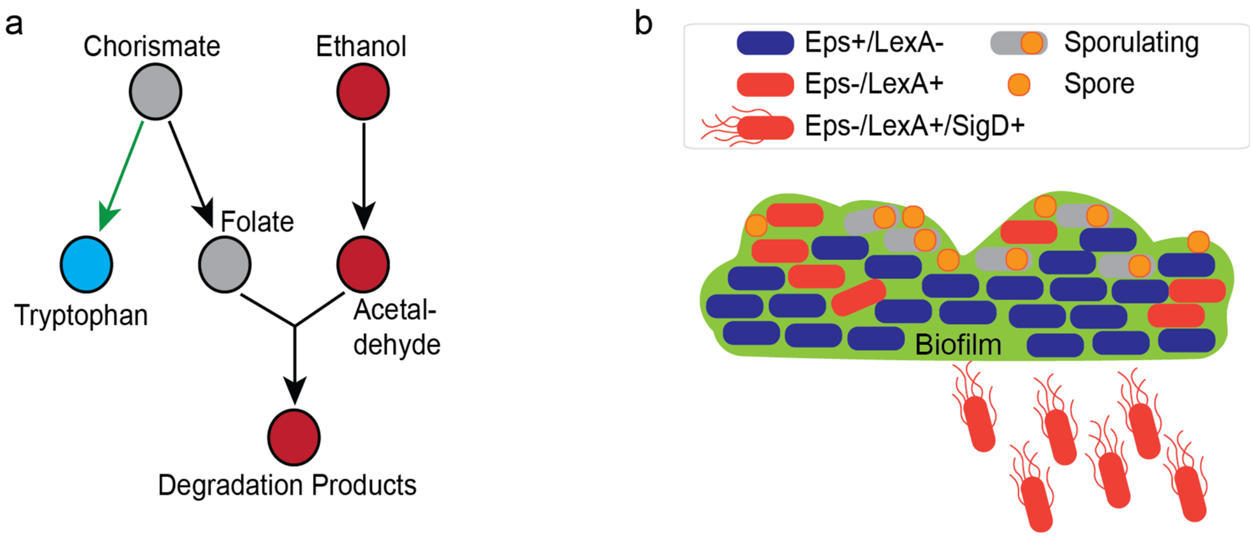

Supplementary Figure 5: Graphical representations of hypotheses.
a. Potential pathway for tryptophan loss in the media during ethanol stress (see Main Fig. 2a), with intermediates removed. In the presence of ethanol, flux from chorismate may be diverted to replenish degraded folate. **b.** Cross-section of biofilm morphology, with the cellular switches between biofilm (Eps, exopolymeric substances) production, LexA (DNA damage response) expression, and sporulation. Swarming cells (SigD+) are likely to only arise from LexA+, Eps- cells (see Main Fig. 2c).

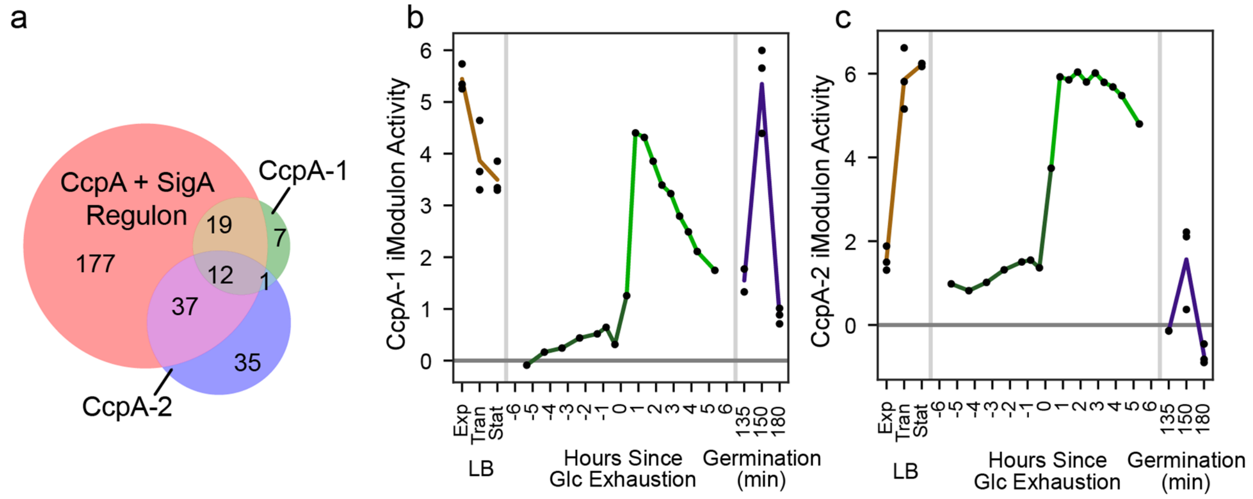

Supplementary Figure 6: The CcpA iModulons

CcpA-1 contains mostly sugar metabolism enzymes (ribose, sucrose, mannose, trehalose, lichenan, etc.) while CcpA-2 contains a mix of genes including those for inositol consumption, tricarboxylic acid permeability, and acetyl-CoA utilization (Full set of genes: Supplementary Data 5). **a.** Venn diagram of gene membership for these iModulons and their matched regulon. **b-c.** Activity of CcpA iModulons for three experiments: growth in LB media ('Exp', 'Tran' and 'Stat' refer to exponential, transition, and stationary phase, respectively), glucose (Glc) exhaustion, and germination. Dots indicate individual samples and lines pass through means. **b.** CcpA-1 is active during exponential growth and germination, but declines in stationary phase and during glucose exhaustion. **c.** CcpA-2 is active during stationary phase and throughout the first 5 hours of glucose exhaustion, and comparatively less active during germination.

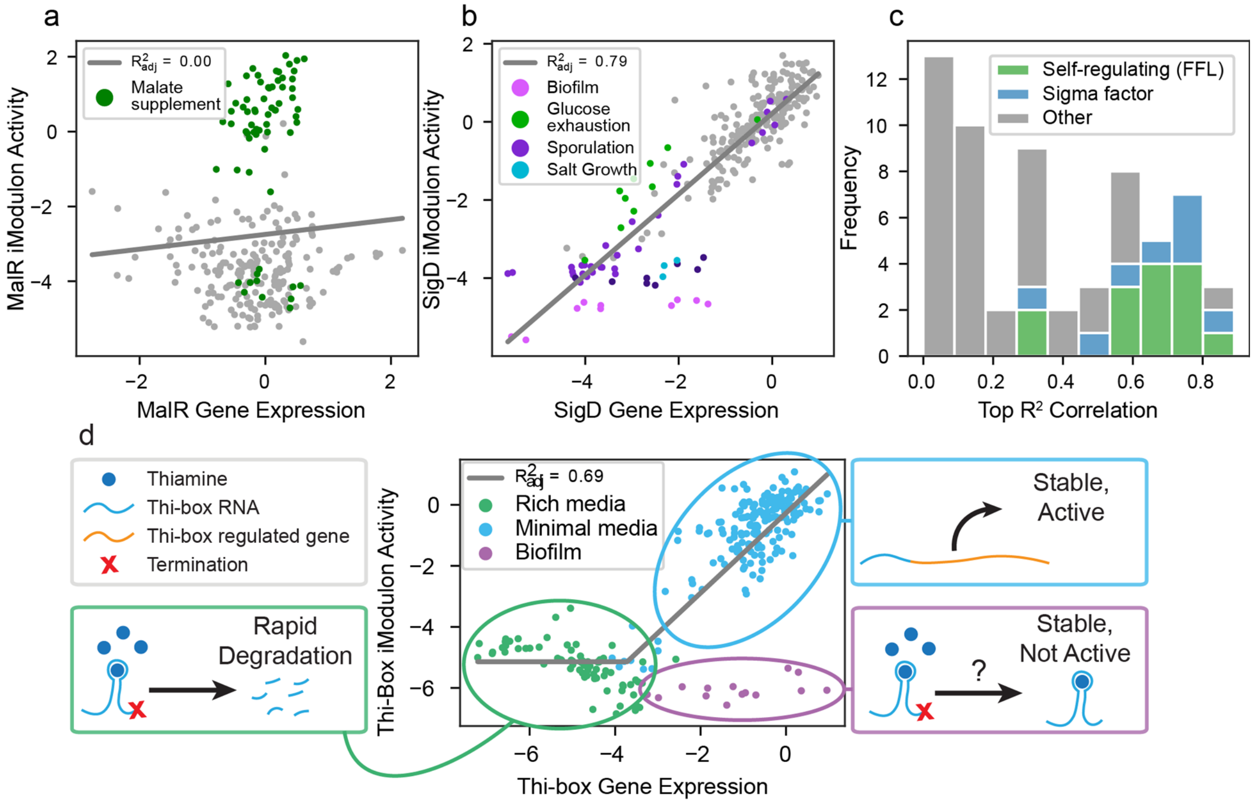

Supplementary Figure 7: Correlation between iModulon activity and regulator expression
Each plot contains an iModulon's transcriptional regulator expression on the x axis and the corresponding activity level on the y axis. **a.** MalR is a typical transcription factor that responds to kinase activity downstream of malate binding. The activity is therefore not correlated with regulator expression but is increased with malate supplementation. **b.** SigD is the sigma factor governing motility, which is regulated at the transcriptional level, so a correlation between activity and expression is observed. Four experiments that exhibit low activity are highlighted; their activity matches expectations from literature. **c.** Histogram of correlations for all iModulons with a known regulator. Self-regulating iModulons are those that contain a TF which also regulates them (FFL: Feed-Forward Loop). Higher correlations are observed for FFL and sigma factor-regulated iModulons. **d.** This Thi-box transcript precedes *thiC*, and the other 4 Thi-box transcripts exhibit similar patterns. A broken line was used for this regression (Supplementary Methods). When activity is low, the expressed RNA contains only the short thi-box sequence, which appears to be degraded quickly in the rich media condition (flasks) but not in the biofilm condition. The thi-box RNA appears to be stable in biofilms for an unknown reason.

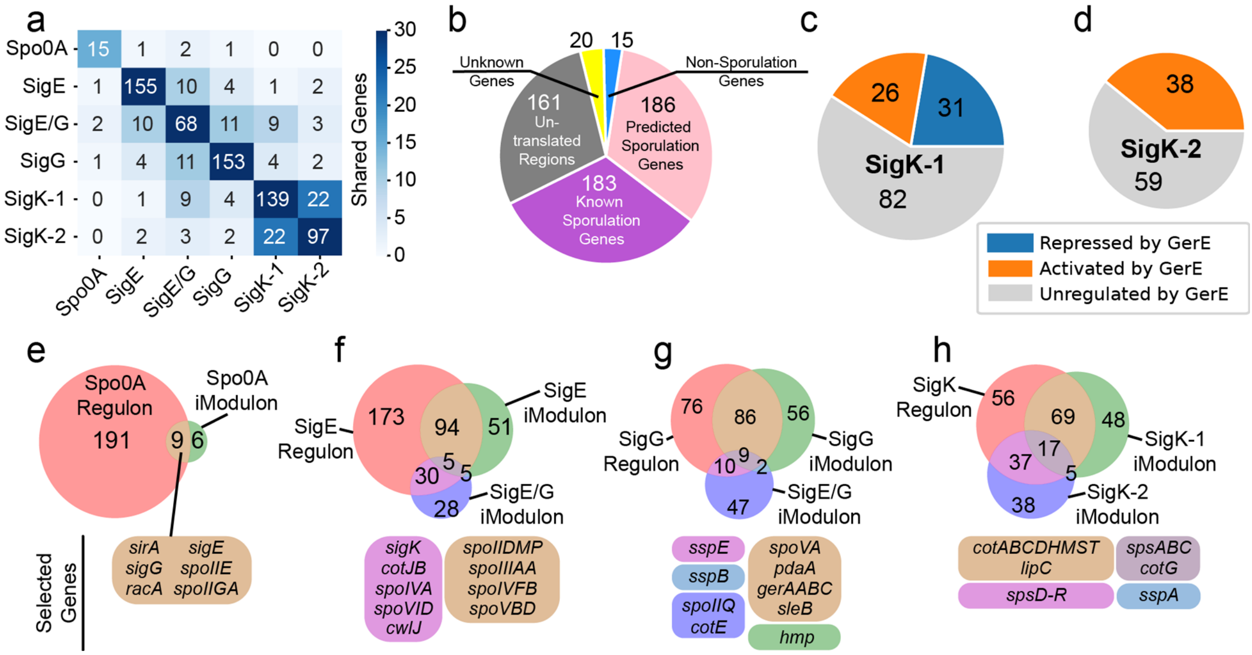

Supplementary Figure 8: The genes in sporulation iModulons

a. Heatmap displaying the number of shared genes (off-diagonal) and self-genes (diagonal) for each iModulon. Many genes are shared between iModulons. **b.** A pie chart of gene type for all transcripts in any of the sporulation iModulons. The 20 “Unknown Genes” are listed in Supplementary Data 10. **c-d.** Pie charts showing GerE interactions with the two SigK iModulons. Pies are sized relative to number of genes, and numbers in slices indicate a number of genes. SigK-1 contains genes repressed by GerE, while SigK-2 does not. **e-h.** Venn diagrams of known regulons and relevant iModulons for each of the enriched regulators. Boxes list important sporulation genes that are present in the subset with a matching color. The genes listed have functions that include transcriptional/post-translational regulation, chromosome segregation, spore coat production, DNA protection, germination initiation, and mother cell lysis.

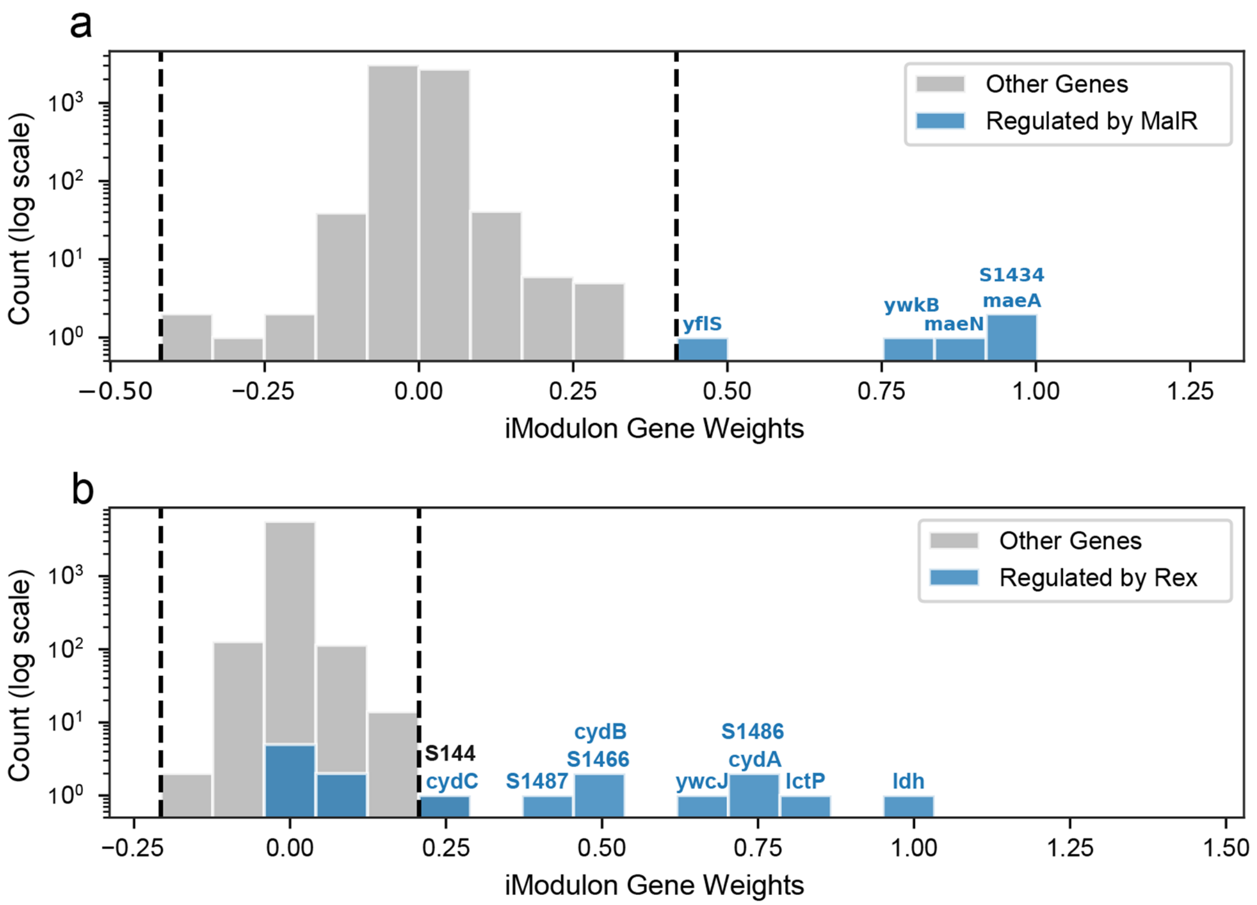

Supplementary Figure 9: The iModulons with increased thresholds
Both histograms show gene or RNA names of iModulon members above their corresponding bars. **a.** Histogram of gene weights for the MalR iModulon, reflecting the curated increase in threshold. **b.** Histogram of gene weights for the Rex iModulon, also reflecting the curated increase in threshold. In both cases, the updated threshold visually corresponds to both the start of the tail and the regulon enrichment. For most other iModulons, the automatically computed threshold already corresponded to this point in the distribution.

Supplementary References

1. Buescher, J. M. *et al.* Global Network Reorganization During Dynamic Adaptations of *Bacillus subtilis* Metabolism. *Science* **335**, 1099–1103 (2012).
2. Doan, T. *et al.* The *Bacillus subtilis ywkA* gene encodes a malic enzyme and its transcription is activated by the YufL/YufM two-component system in response to malate. *Microbiology (Reading, Engl.)* **149**, 2331–2343 (2003).
3. Tanaka, K., Kobayashi, K. & Ogasawara, N. The *Bacillus subtili*s YufLM two-component system regulates the expression of the malate transporters MaeN (YufR) and YflS, and is essential for utilization of malate in minimal medium. *Microbiology (Reading, Engl.)* **149**, 2317–2329 (2003).
4. Garnett, J. A., Marincs, F., Baumberg, S., Stockley, P. G. & Phillips, S. E. V. Structure and function of the arginine repressor-operator complex from *Bacillus subtilis*. *J. Mol. Biol.* **379**, 284–298 (2008).
5. Brill, J., Hoffmann, T., Bleisteiner, M. & Bremer, E. Osmotically controlled synthesis of the compatible solute proline is critical for cellular defense of *Bacillus subtilis* against high osmolarity. *J. Bacteriol.* **193**, 5335–5346 (2011).
6. Winkler, W., Nahvi, A. & Breaker, R. R. Thiamine derivatives bind messenger RNAs directly to regulate bacterial gene expression. *Nature* **419**, 952–956 (2002).
7. Rodionov, D. A., Vitreschak, A. G., Mironov, A. A. & Gelfand, M. S. Comparative genomics of thiamin biosynthesis in procaryotes. New genes and regulatory mechanisms. *J. Biol. Chem.* **277**, 48949–48959 (2002).
8. Schumann, W. The *Bacillus subtilis* heat shock stimulon. *Cell Stress Chaperones* **8**, 207–217 (2003).
9. Escorcia-Rodríguez, J. M., Tauch, A. & Freyre-González, J. A. Abasy Atlas v2.2: The most comprehensive and up-to-date inventory of meta-curated, historical, bacterial regulatory networks, their completeness and system-level characterization. *Comput Struct Biotechnol J* **18**, 1228–1237 (2020).
10. Sastry, A. V. *et al.* The *Escherichia coli* transcriptome mostly consists of independently regulated modules. *Nat Commun* **10**, (2019).
11. Pedregosa, F. Scikit-learn: Machine Learning in Python. *MACHINE LEARNING IN PYTHON* 6.
12. D'Agostino, R. B. & Belanger, A. A Suggestion for Using Powerful and Informative Tests of Normality. *The American Statistician* **44**, 316–321 (1990).
13. Zhu, B. & Stülke, J. *Subti*Wiki in 2018: from genes and proteins to functional network annotation of the model organism *Bacillus subtilis*. *Nucleic Acids Res.* **46**, D743–D748 (2018).
14. Oliphant, T. Python for Scientific Computing. *Computing in Science & Engineering* **9**, 10–20 (2007).
15. Remmert, M., Biegert, A., Hauser, A. & Söding, J. HHblits: lightning-fast iterative protein sequence searching by HMM-HMM alignment. *Nat Methods* **9**, 173–175 (2012).
16. Clark, K., Karsch-Mizrachi, I., Lipman, D. J., Ostell, J. & Sayers, E. W. GenBank. *Nucleic Acids Res* **44**, D67–D72 (2016).
17. Cock, P. J. A. *et al.* Biopython: freely available Python tools for computational molecular biology and bioinformatics. *Bioinformatics* **25**, 1422–1423 (2009).
18. Mirdita, M. *et al.* Uniclust databases of clustered and deeply annotated protein sequences and alignments. *Nucleic Acids Res* **45**, D170–D176 (2017).
19. Bailey, T. L. *et al.* MEME Suite: tools for motif discovery and searching. *Nucleic Acids Res* **37**, W202–W208 (2009).
20. Münch, R. *et al.* PRODORIC: prokaryotic database of gene regulation. *Nucleic Acids Res.* **31**, 266–269 (2003).

21. Gupta, S., Stamatoyannopoulos, J. A., Bailey, T. L. & Noble, W. S. Quantifying similarity between motifs. *Genome Biol* **8**, R24 (2007).
22. Freyre-González, J. A. *et al.* Lessons from the modular organization of the transcriptional regulatory network of *Bacillus subtilis*. *BMC Systems Biology* **7**, 127 (2013).
23. Nicolas, P. *et al.* Condition-Dependent Transcriptome Reveals High-Level Regulatory Architecture in Bacillus subtilis. *Science* **335**, 1103–1106 (2012).
24. Arrieta- Ortiz, M. L. *et al.* An experimentally supported model of the *Bacillus subtilis* global transcriptional regulatory network. *Molecular Systems Biology* **11**, 839 (2015).
25. Eichenberger, P. *et al.* The program of gene transcription for a single differentiating cell type during sporulation in *Bacillus subtilis*. *PLoS Biol.* **2**, e328 (2004).
26. Wang, S. T. *et al.* The forespore line of gene expression in *Bacillus subtilis*. *J. Mol. Biol.* **358**, 16–37 (2006).
