# Supplementary material for: Machine learning uncovers independently regulated modules in the Bacillus subtilis transcriptome
Source: Nat Commun. 2020 Dec 11;11:6338. doi: 10.1038/s41467-020-20153-9 (PMC7732839; doi:10.1038/s41467-020-20153-9)
Supplement: Supplementary file 3 — Description of Additional Supplementary Files [file 41467_2020_20153_MOESM3_ESM.pdf]

Description of Additional Supplementary Files

Supplementary Data 1: Growth Conditions

File: Supplementary Data 1-5.xlsx

Sheet: "1 - Growth Conditions"

Supplementary Data 1 contains a list of all conditions used, which were generated by Nicolas, *et al.*, 2012. We removed three noisy samples (see Methods). The columns describe the following:

- *Sample_n:* sample number
- *Condition:* name of the condition
- *Experiment:* name of the experiment. Activity plots in the main figures contain gray lines separating unique experiments.
- *Experiment_description:* Description of the experiment and conditions, copied from the original dataset
- *Cells_collected:* description of collected cells, copied from the original dataset
- *X_label:* the label used for the condition in the activity plots in the main figures, if applicable
- *Exp_label:* the experiment label used (appears floating below the *x_label*) in the activity plots, if applicable
- *Control:* Most relevant control group for this sample, if applicable. Used to determine statistical significance in the activity plots for Supplementary Data 6.
- *Color:* Hex code for the condition's color, used for plots
- *Exp_color:* Hex code for the experiment's color, used for time course plots
- *Ref:* Reference that generated the data. Parentheses indicate specific members of the original group that rain the experiments.

Supplementary Data 2: Expression Data (X)

File: Supplementary Data 1-5.xlsx

Sheet: "2 - X - Expression Data"

For unprocessed expression data, see the original dataset (Nicolas, et al., 2012). Rows indicate genes and non-coding RNAs, and columns indicate samples. Each value is a log-transformed microarray expression value that has been centered such that the mean of the baseline condition (M9_exp) for all genes is 0.

Supplementary Data 3: TRN Structure (M)

File: Supplementary Data 1-5.xlsx

Sheet: "3 - M - TRN Structure"

This matrix links the genes to their corresponding iModulons. Columns indicate iModulons (see Supplementary Data 7 for a description of each iModulon), rows indicate genes or non-coding RNAs. The values represent the weight of each gene in determining the activity of each iModulon. The top two rows are thresholds: The top row was automatically computed (Methods), and the second row was curated in seven cases to retain genes (if the threshold was decreased) or remove excessive noise from an otherwise meaningful iModulon (if the threshold was increased). All genes with an absolute value in the column greater than the curated threshold are considered to be members of the iModulon.

Supplementary Data 4: Activity (A)

File: Supplementary Data 1-5.xlsx

Sheet: "4 - A - Activity"

This matrix contains the condition-dependent activity of each iModulon. Rows indicate iModulons (Supplementary Data 7) and columns indicate conditions (Supplementary Data 1). Values indicate how active the iModulon is in the condition, with zero indicating the activity in the baseline condition (M9exp) and each unit being approximately one log(expression value) if the iModulon had only one gene.

Supplementary Data 5: iModulon Presence

File: Supplementary Data 1-5.xlsx

Sheet: "5 - iModulon Presence"

Rows are genes and non-coding RNAs and columns are iModulons; a 1 indicates that the gene is in the iModulon and a 0 indicates that it is not. This is simply a thresholded version of the **M** matrix (Supplementary Data 3).

Supplementary Data 6: iModulon Dashboards

File: Supplementary Data 6 - Dashboards.pdf

This PDF contains automatically generated summaries of each iModulon. Note that the gene lists and counts here will include non-coding RNAs, which were usually omitted for simplicity in the main text.

- *Title:* n - Short name - long name. N corresponds to the iModulon number in Supplementary Data 3-5
- *Biological function:* brief description of the function of the iModulon's genes
- *Regulon:* includes the category that the iModulon falls into (see Main Fig. 1C) and the string of regulators. The regulator may be a boolean combination with '/' denoting union of regulons and '+' denoting intersection.
- *Plot 1:* Scatter plot of mean gene expression (Supplementary Data 2) and iModulon gene weight (Supplementary Data 3), with horizontal lines indicating the weight threshold and colors indicating gene category annotations from *Subti*Wiki. Gene categories of iModulon member genes are listed in the legend, with the number of member genes in each category in parentheses.
- *Plot 2:* Semi-log histogram of gene weights. Regulated genes are colored as shown in the legend, and member genes are listed above the appropriate bars.
- *Plot 3:* Activity level of the iModulon across all conditions (mean ± standard deviation). Stars indicate statistically significant conditions relative to their matched control (FDR < 0.05, Supplementary Data 7, Methods), and shaded backgrounds indicate significant correlation with time (Pearson R > 0.8, FDR < 0.05).
- *Plot 4:* Venn diagram of the known regulon (red), the annotated iModulon genes (green), and the unannotated iModulon genes (blue). Numbers indicate the size of the subset.
- *Plot 5:* Scatter plot(s) of regulator expression and iModulon activity (see Figure 3D-F). A best fit line and adjusted R^2 value is shown. If there are multiple regulators, the adjusted R^2 was computed for all of them and the top 3 regulators are shown, sorted with the highest

correlation on the left. Colors of points match those in plot 3 and are listed in Supplementary Data 1.

- *Motifs:* If the iModulon contained genes from four or more operons, the genome sequence was searched for upstream consensus motifs (see Supplementary Methods). If there was a consensus, and if that consensus matches a known motif, the results are shown in the bottom right of the dashboard.

Visit imodulondb.org for more information and interactive versions of these dashboards.

Supplementary Data 7: Summary table of iModulons

File: Supplementary Data 7.xlsx

Numbering is consistent with Supplementary Data 3-6.

- *Regulator(s):* All regulator annotations were downloaded from *Subti*Wiki[13]. Where multiple regulators are listed, '+' indicates the intersection of regulons, and '/' with brackets indicates the union of separate regulons.
- Mode of Regulation: For each regulator in regulator(s), this column lists the mode of regulation, which may be activation, repression, sigma factor, antitermination, RNA switch, or the ambiguous "regulation". The word "regulation" indicates complex modulation or a mix or repression and activation within the iModulon.
- Enrichment P-value: P-values were computed with the two-sided Fisher Exact test in scipy, and multiple comparison correction was not performed.
- Top Activity-TF R^2_{adj}: The R^2 correlation between the iModulon activity and its matched regulator, adjusted for the minimum activity level before a correlation is observed, is given. If there are multiple regulators, all R^2_{adj} were computed and the maximum is reported. See Supplementary Methods.

Supplementary Data 8: Uncharacterized and divergently regulated genes

File: Supplementary Data 8.xlsx

Genes were included if they are coding sequences with their description, product, or function annotated as "unknown" on *Subti*Wiki, or if they are not known to be regulated by the iModulon's regulator. These genes and gene/regulator relationships are opportunities for discovery. For the complete list of genes in any iModulon, use the iModulon presence matrix, Supplementary Data 5.

Supplementary Data 9: Activating Conditions

File: Supplementary Data 9.xlsx

Expected and unexpected activating conditions for characterized iModulons. Expectations are based on the known mechanism of the enriched regulator combination and the expected function of the iModulon. Elucidating the mechanisms of unexpected activation is a potential path for discovery.

Supplementary Data 10: Motif Discovery

File: Supplementary Data 10.xlsx

The top identified motif (Supplementary Methods) for each iModulon that has one is shown in this table. If the motif matched a motif in the PRODORIC database (E < 0.01), then the identifier for the similar motif, its associated transcription factor (TF), and E-value are shown. If the "Similar Motif TF" does not contain a dash and organism name, then the organism is *Bacillus subtilis*.

Supplementary Data 11: Uncharacterized genes in sporulation iModulons

File: Supplementary Data 11.xlsx

Genes were included in this table if:

1. They are a member of at least one sporulation iModulon
2. They are coding sequences or pseudogenes with their description, product, or function annotated as "unknown" on SubtiWiki
3. They were not categorized as "Known Sporulation Protein" or "Predicted Sporulation Protein" on SubtiWiki

After generating the table, we cross-checked the genes with three major publications: Arietta-Ortiz, et al., which predicted a full TRN that captures sporulation; Eichenberger, *et al.*, which described transcription in the mother cell; and Wang, *et al.*, which described transcription in the forespore. The latter two papers used knock-out (KO), overexpression, and promoter prediction data as evidence for gene-regulator relationships. The prediction from Arietta-Ortiz and evidence from Eichenberger and Wang are listed in this table where applicable. Rows highlighted in yellow are genes without a pre-existing literature connection to sporulation, but that are involved in the iModulons of sporulation.
