# Supplementary material for: Machine learning uncovers independently regulated modules in the Bacillus subtilis transcriptome
Source: Nat Commun. 2020 Dec 11;11:6338. doi: 10.1038/s41467-020-20153-9 (PMC7732839; doi:10.1038/s41467-020-20153-9)

Supplementary Data 6: iModulon Dashboards

Machine learning uncovers independently regulated modules in the Bacillus subtilis transcriptome

Authors: Kevin Rychel, Anand V. Sastry, Bernhard O. Palsson

This PDF contains automatically generated summaries of each iModulon. Note that the gene lists and counts here will include non-coding RNAs, which were usually omitted for simplicity in the main text.

- *Title:* N - Short Name - Long Name. N corresponds to the iModulon number in Supplementary Data 3-5
- *Biological function:* brief description of the function of the iModulon's genes
- *Regulon:* includes the category that the iModulon falls into (see Main Fig. 1c) and the string of regulators. The regulator may be a boolean combination with '/' denoting union of regulons and '+' denoting intersection.
- *Plot 1:* Scatter plot of mean gene expression (Supplementary Data 2) and iModulon gene weight (Supplementary Data 3), with horizontal lines indicating the weight threshold and colors indicating gene category annotations from *Subti*Wiki. Gene categories of iModulon member genes are listed in the legend, with the number of member genes in each category in parentheses.
- *Plot 2:* Semi-log histogram of gene weights. Regulated genes are colored as shown in the legend, and member genes are listed above the appropriate bars.
- *Plot 3:* Activity level of the iModulon across all conditions (mean ± standard deviation). Stars indicate statistically significant conditions relative to their matched control (FDR < 0.05, Supplementary Data 7, Methods), and shaded backgrounds indicate significant correlation with time (Pearson R > 0.8, FDR < 0.05).
- *Plot 4:* Venn diagram of the known regulon (red), the annotated iModulon genes (green), and the unannotated iModulon genes (blue). Numbers indicate the size of the subset.
- *Plot 5:* Scatter plot(s) of regulator expression and iModulon activity (see Figure 3D-F). A best fit line and adjusted R^2 value is shown. If there are multiple regulators, the adjusted R^2 was computed for all of them and the top 3 regulators are shown, sorted with the highest correlation on the left. Colors of points match those in plot 3 and are listed in Supplementary Data 1.
- *Motifs:* If the iModulon contained genes from four or more operons, the genome sequence was searched for upstream consensus motifs (see Supplementary Methods). If there was a consensus, and if that consensus matches a known motif, the results are shown in the bottom right of the dashboard.

Visit imodulondb.org for more information and interactive versions of these dashboards.

0 - FadR – Fatty Acids

Biological Function:
Fatty acid degradation

Well-defined regulon:
FadR

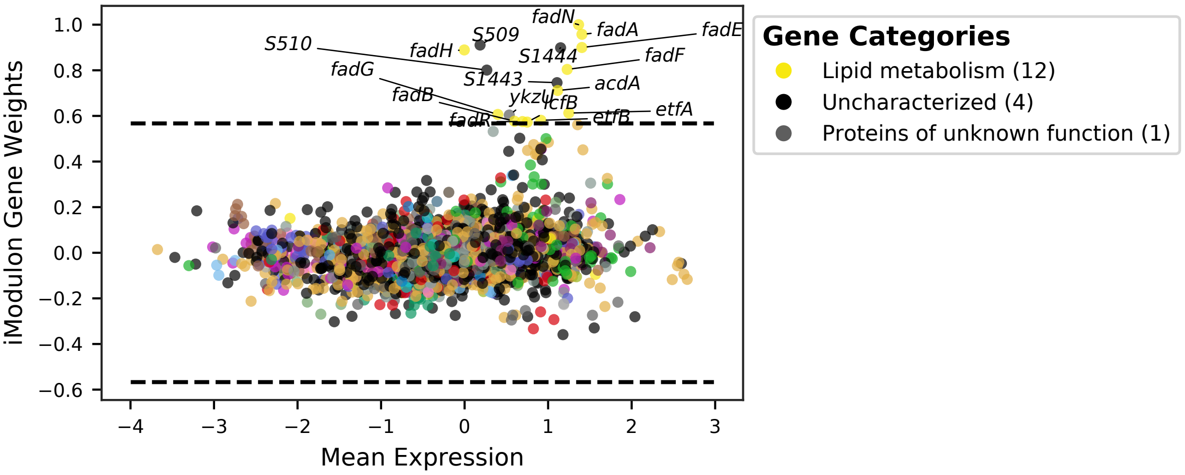

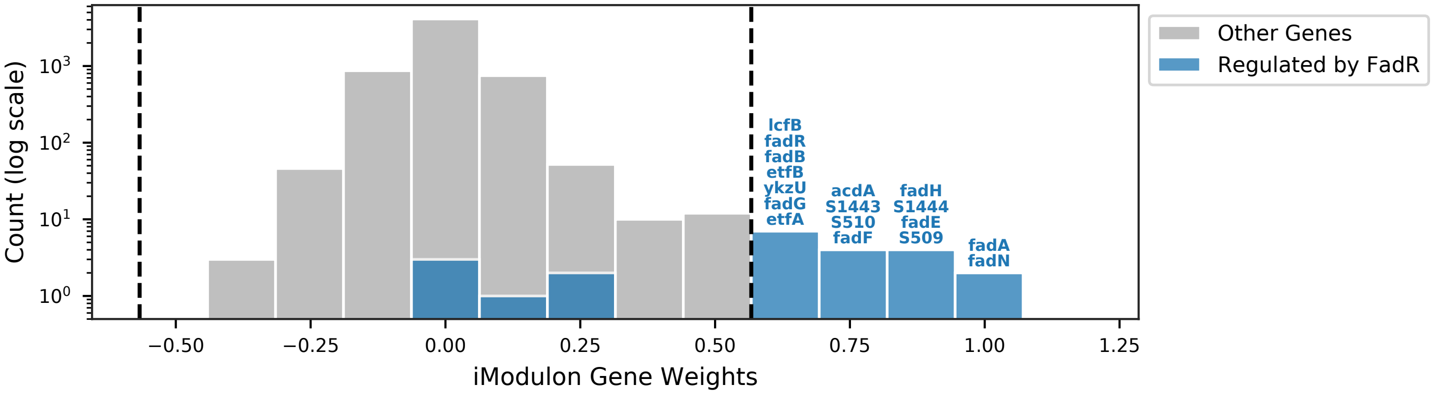

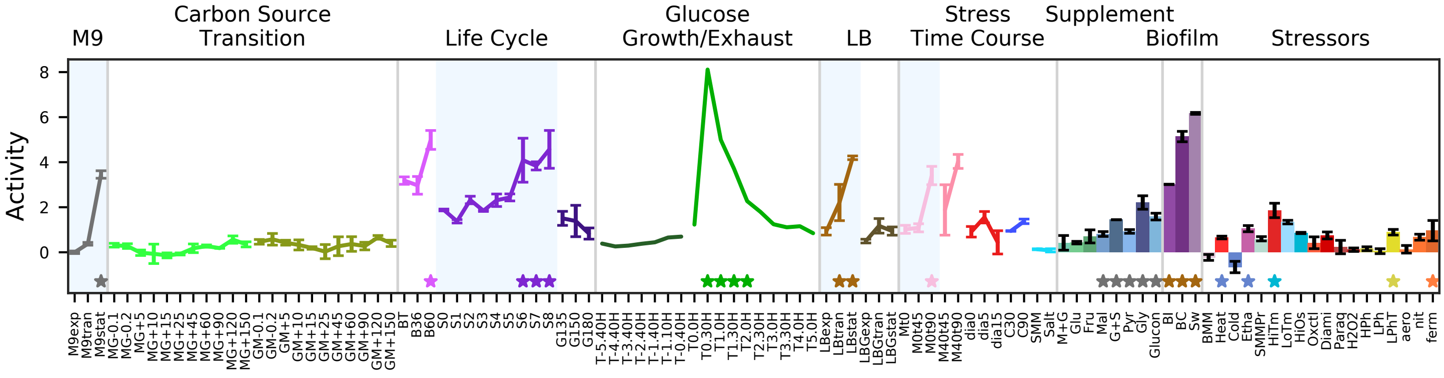

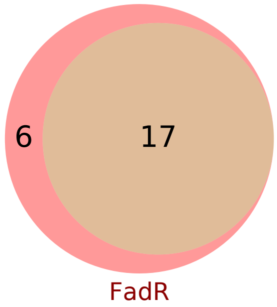

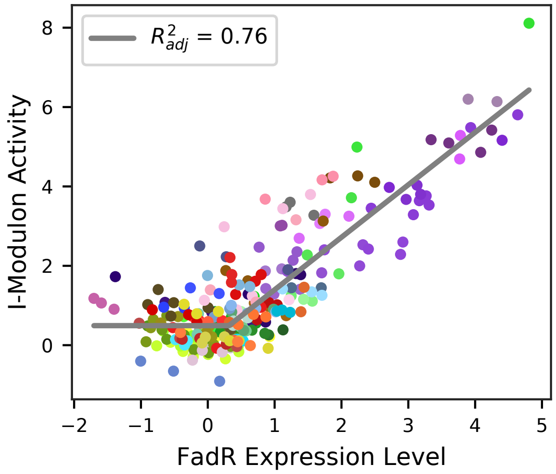

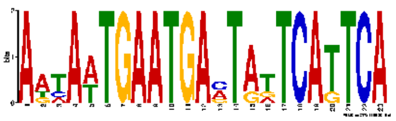

Motif E-value: 2.40e-10
Operons with Upstream Motif: 71%

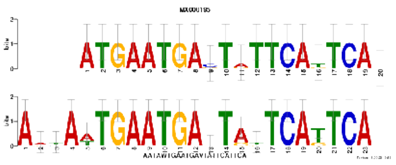

Motif similar to FadR (E = 5.34e-12)

1 - MalR – Malate

Biological Function:
Malate uptake and utilization

Well-defined regulon:
MalR

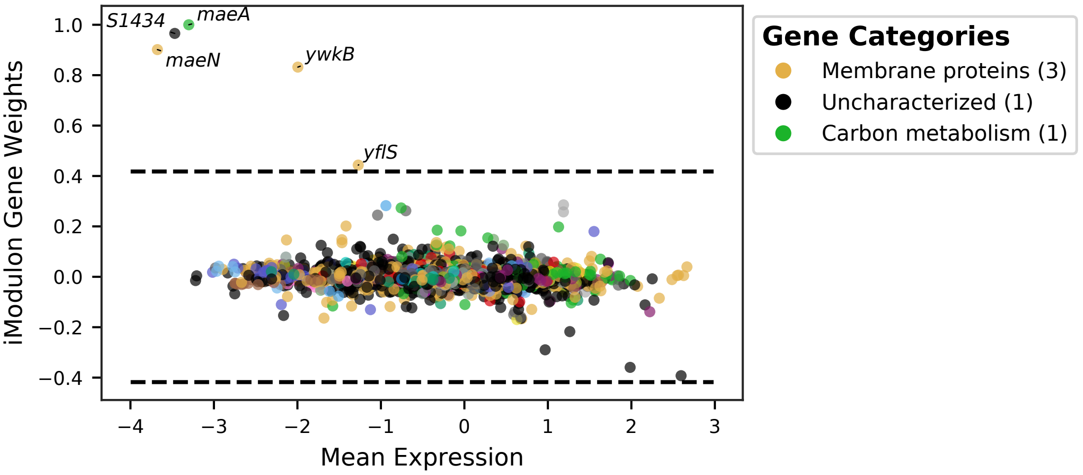

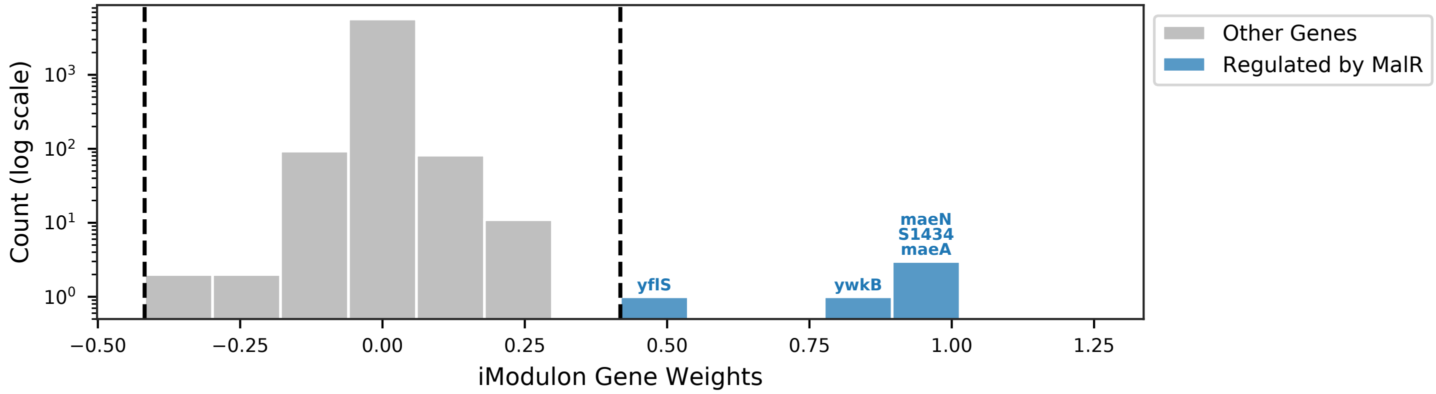

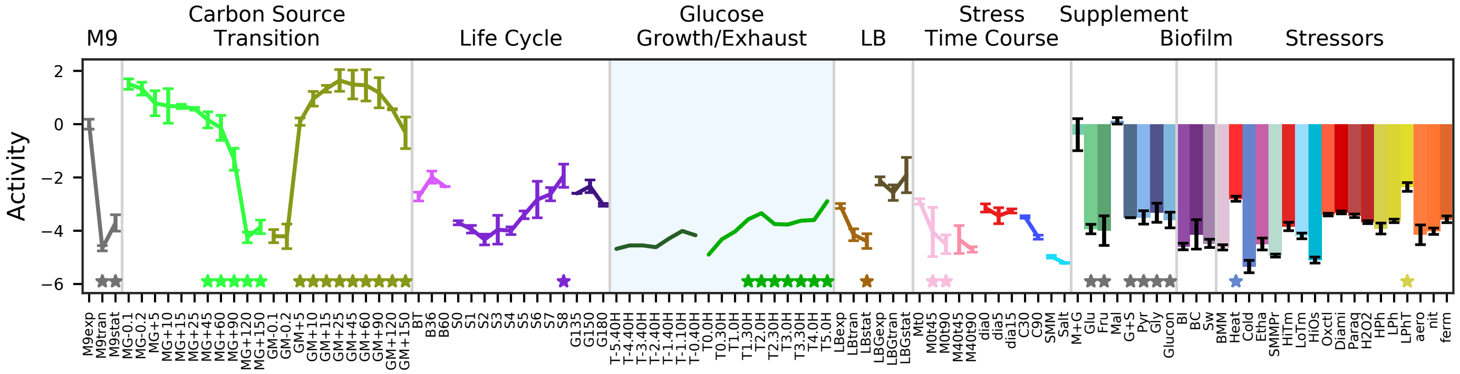

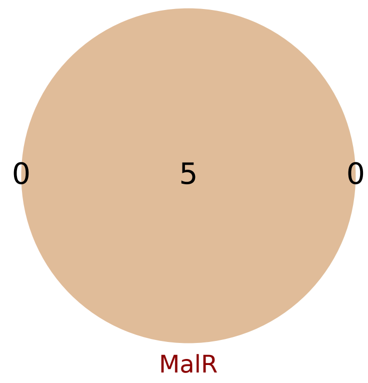

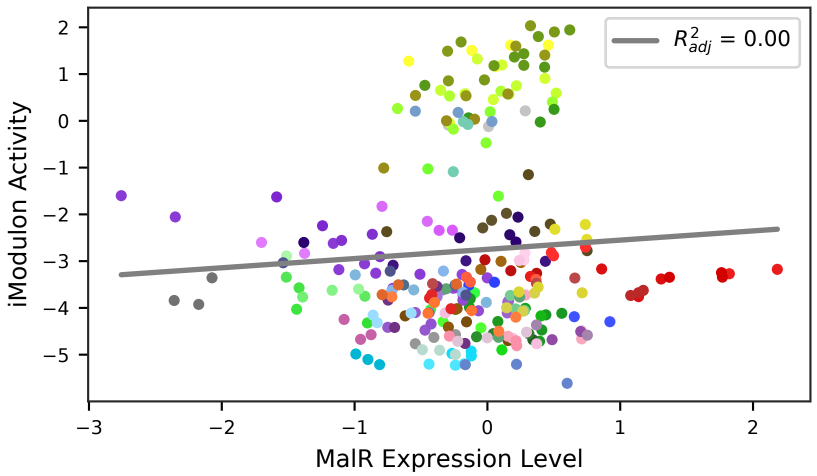

2 - GlpP – Glycerol

Biological Function:
Glycerol uptake and utilization

Well-defined regulon:
GlpP

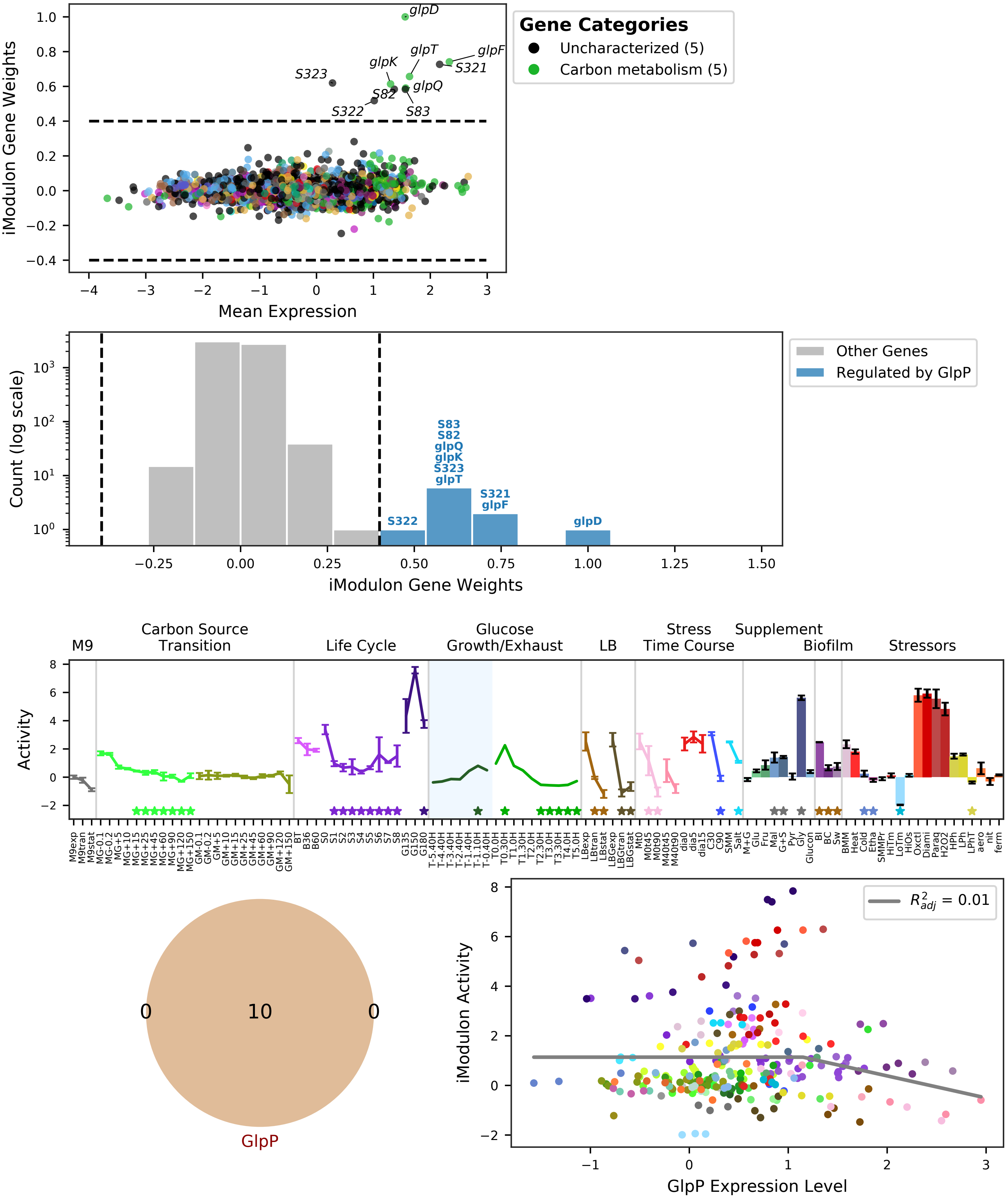

3 - FruR – Fructose

Biological Function:
Fructose uptake and utilization

Well-defined regulon:
FruR

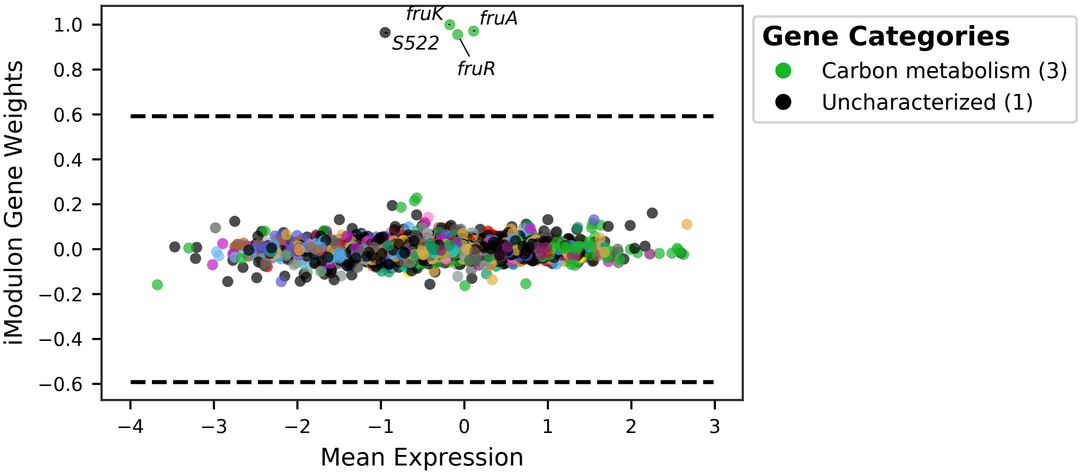

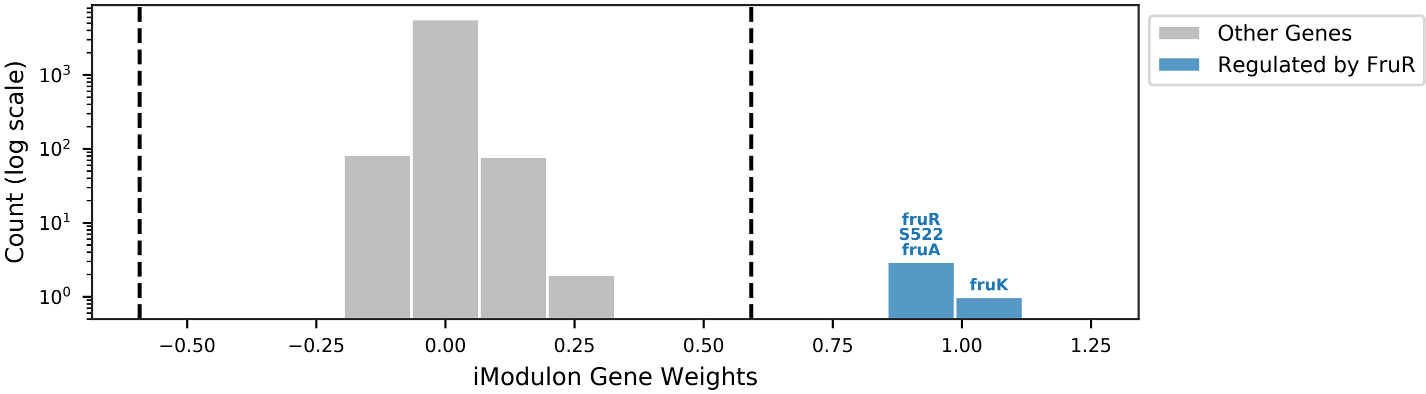

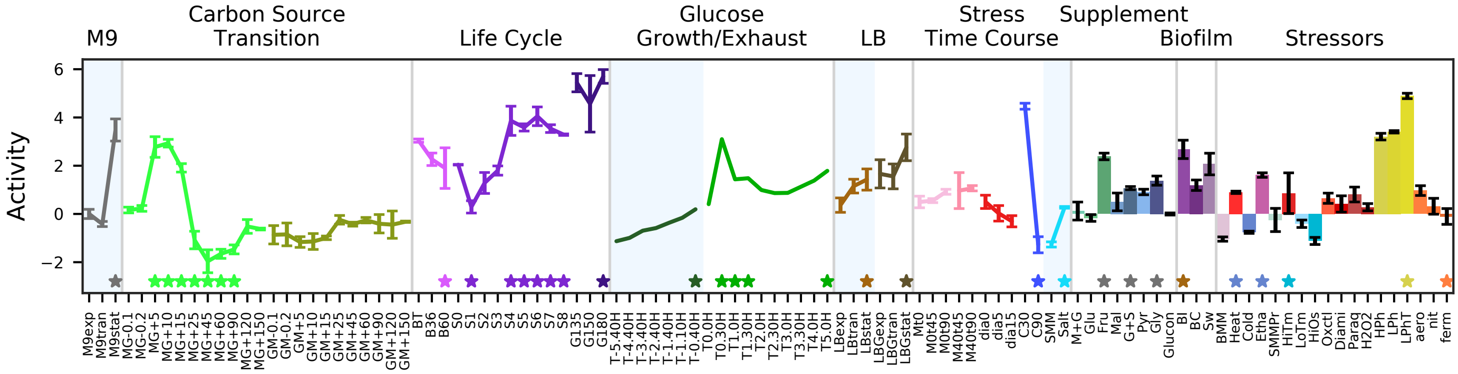

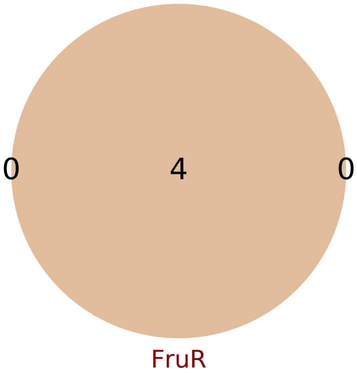

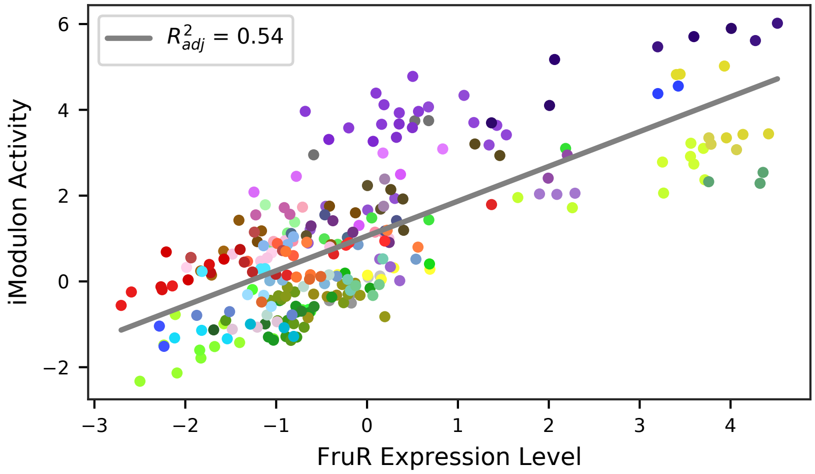

4 - LevR – Levan and Fructose

Biological Function:
Fuctose uptake and phosphorylation, degradation of the exopolysaccharide levan into fructose

Well-defined regulon:
LevR

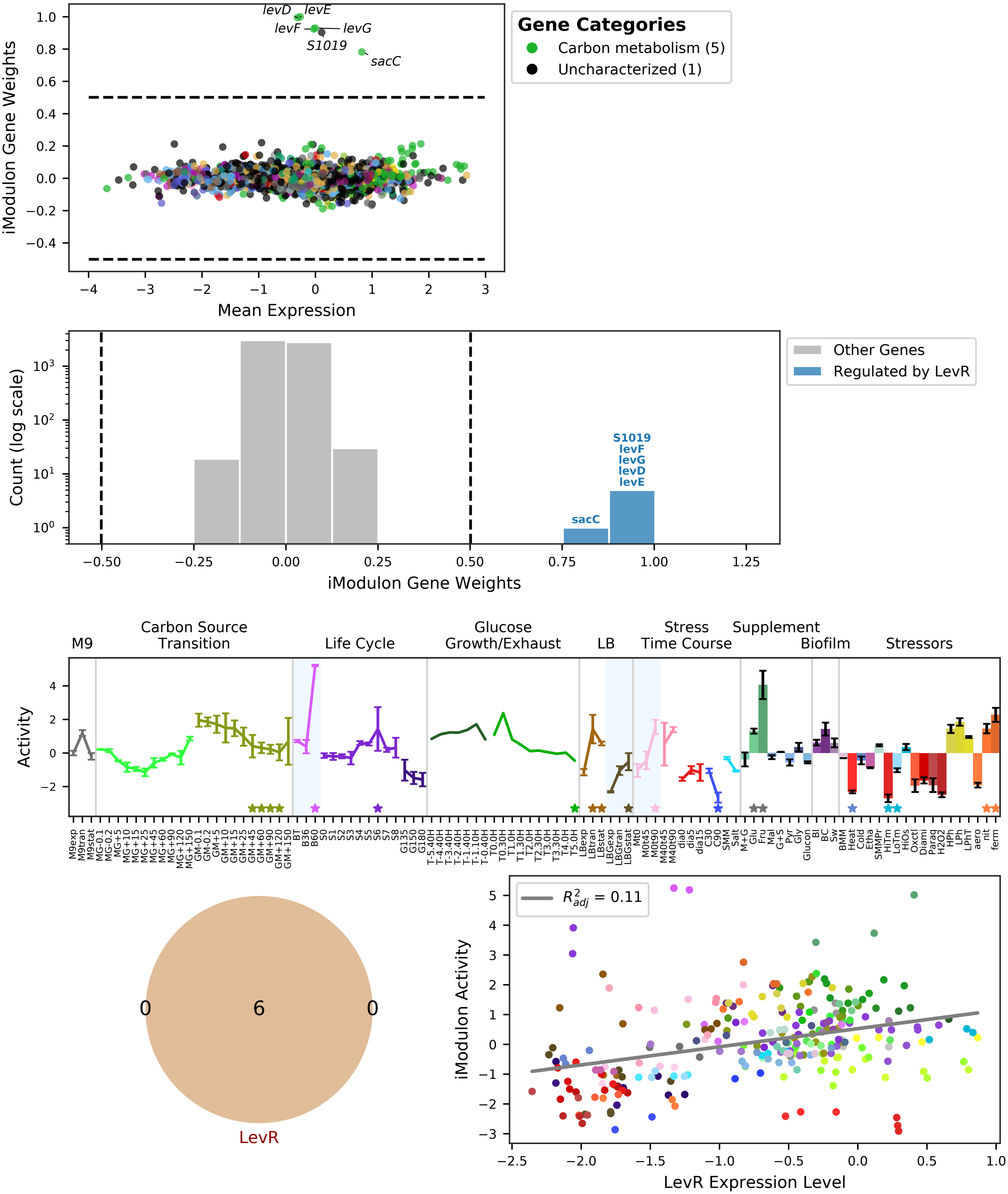

5 - FrlR – Amino Sugars

Biological Function:
Amino sugar uptake and metabolism

Well-defined regulon:
FrlR

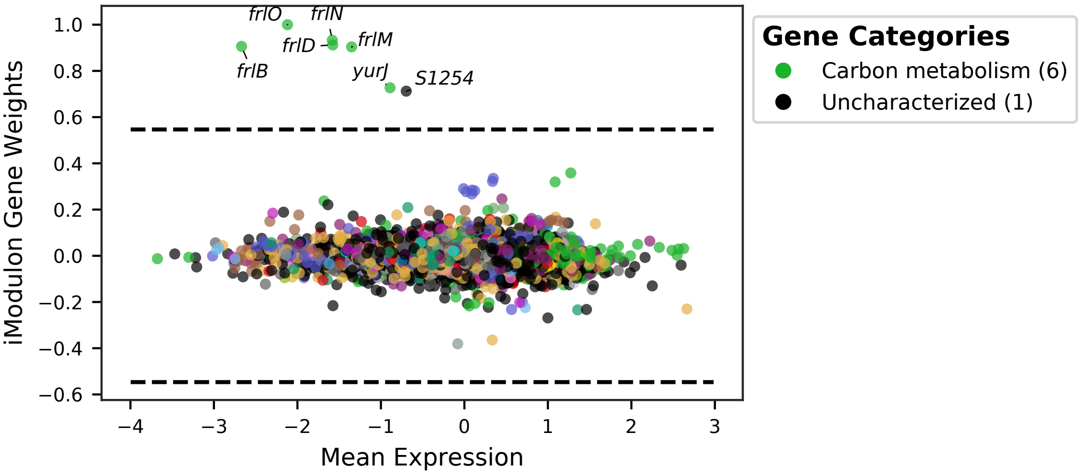

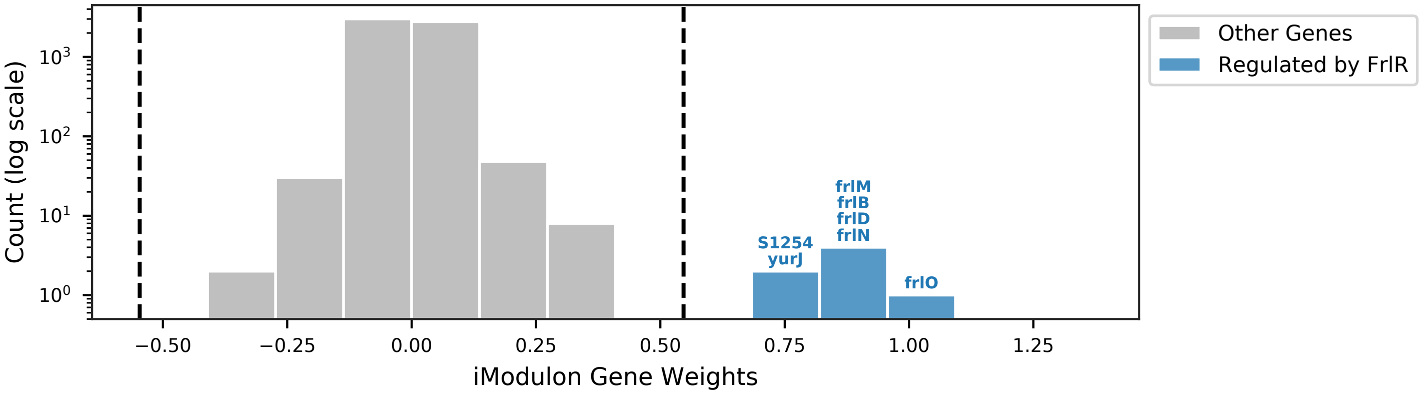

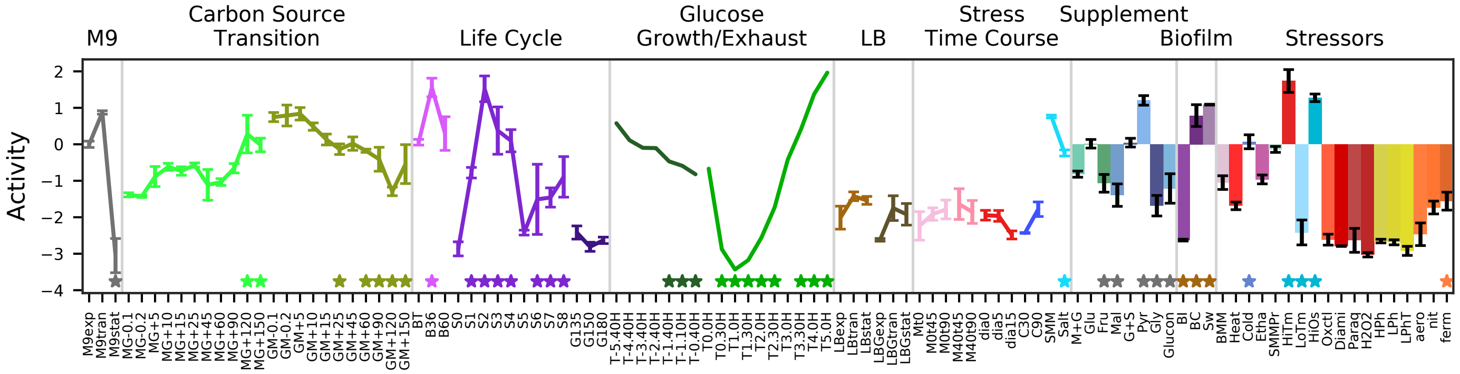

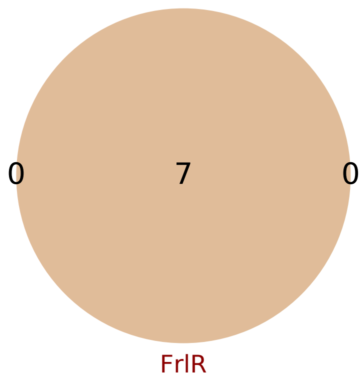

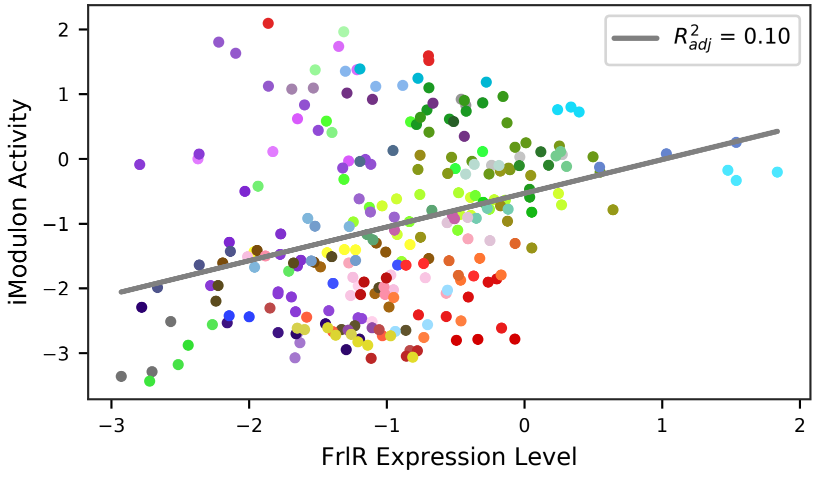

6 - ManR – Mannose

Biological Function:
Utilization of mannose for cell wall synthesis. Needed for exponential growth in LB media (absent glucose) or in the presence of fructose

Well-defined regulon:
ManR

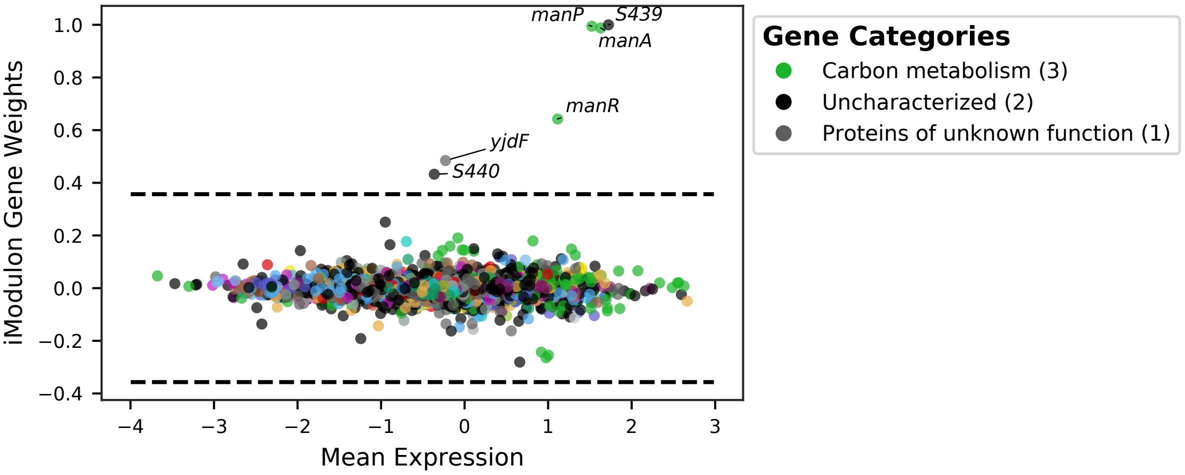

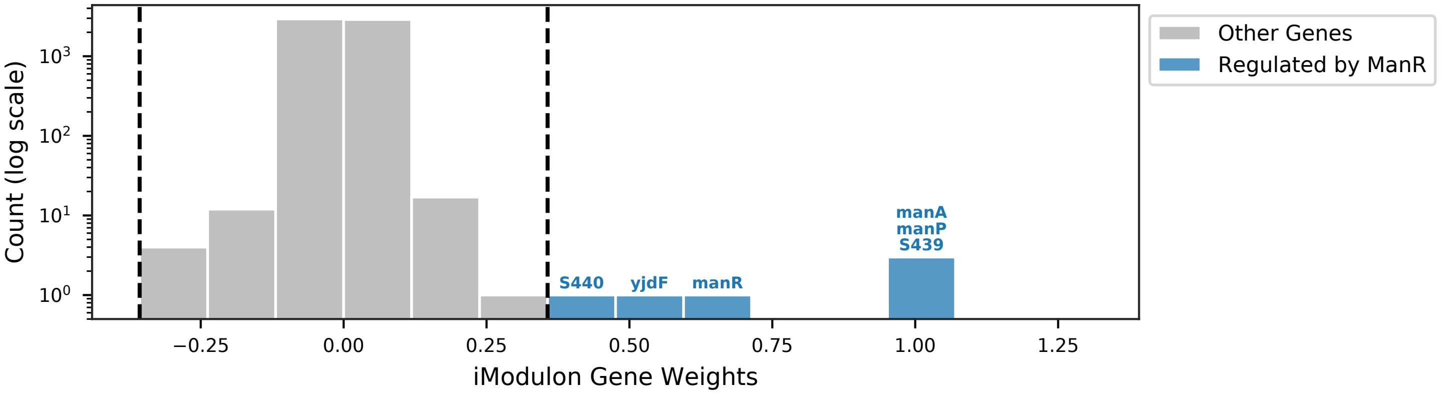

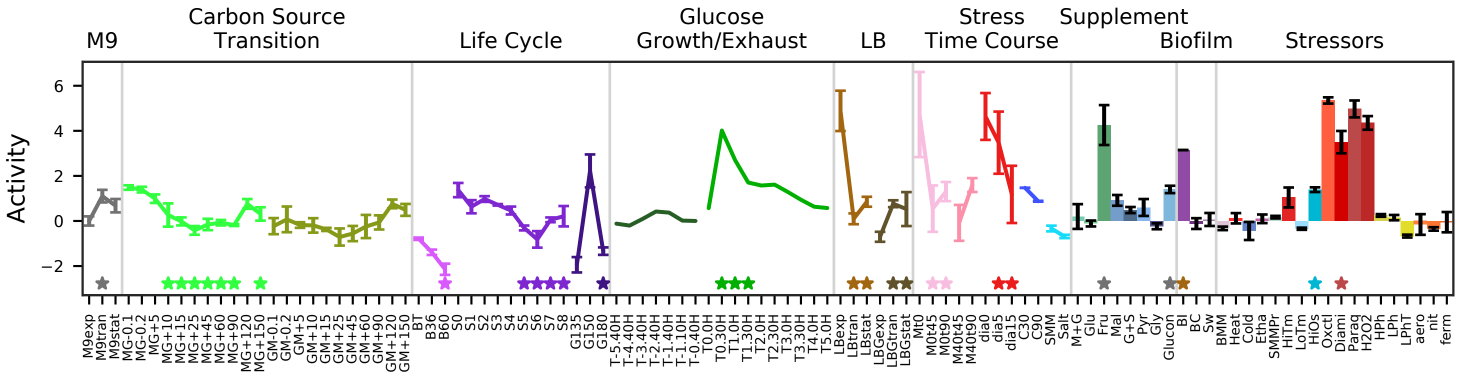

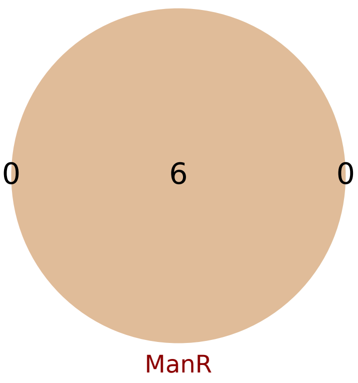

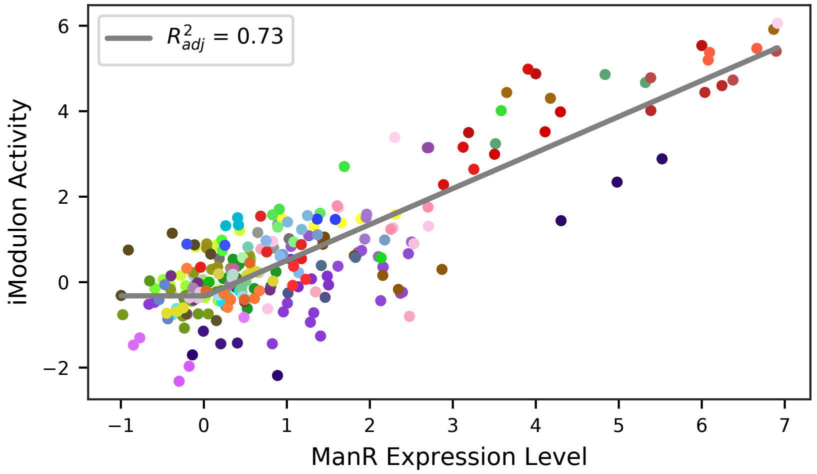

7 - AcoR – Acetoin

Biological Function:
Utilization of acetoin, extracellular energy storage

Well-defined regulon:
AcoR

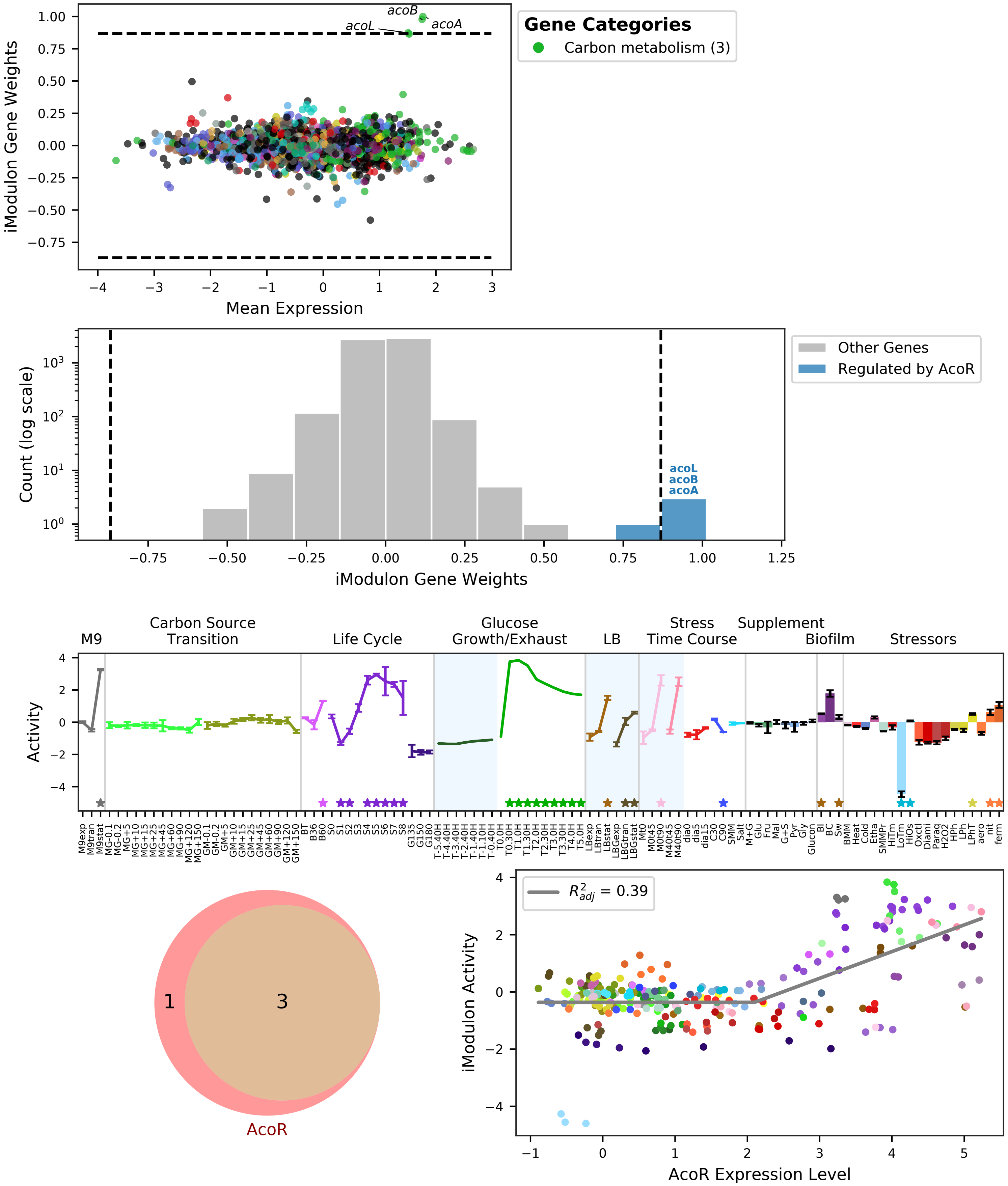

8 - LicR – Lichenan

Biological Function:
Uptake, phosphorylation, and utilization of lechenin, a glucan found in soil

Contains unknown genes and known regulon:
LicR

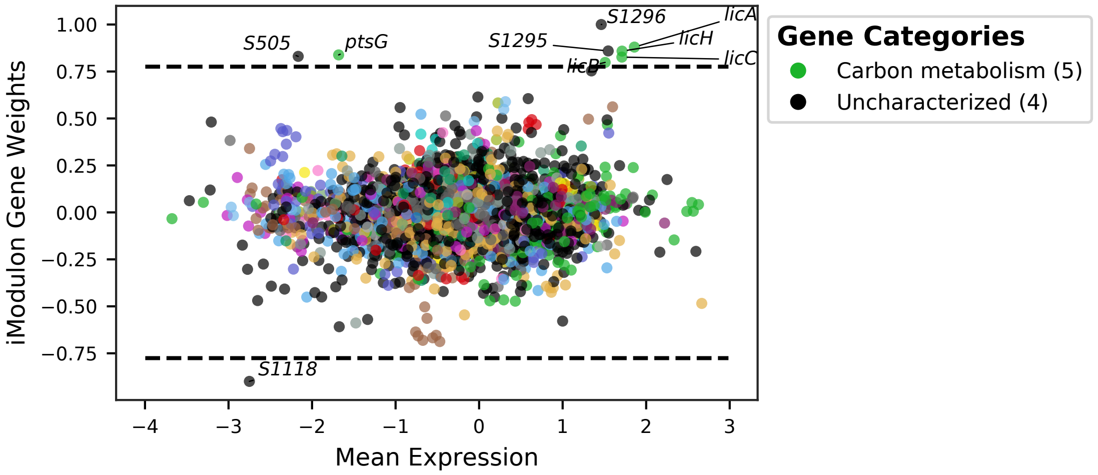

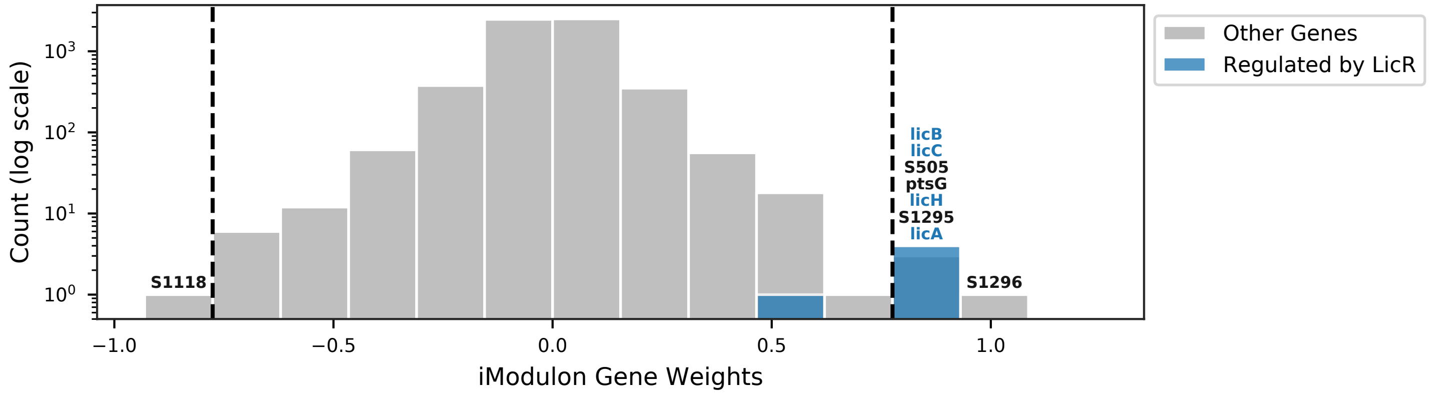

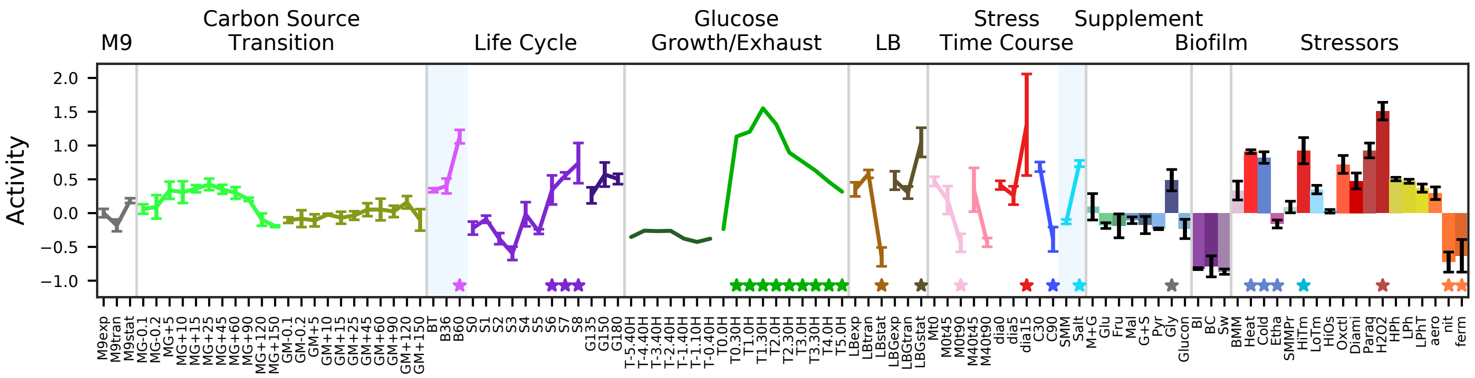

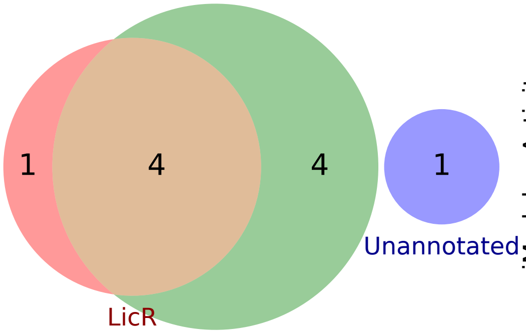

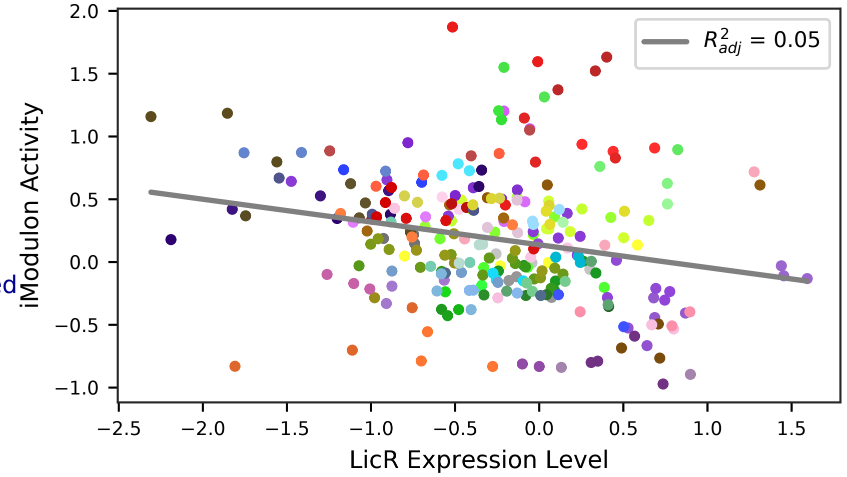

9 - CcpA-1 – Low Glucose 1

Biological Function:
Uptake and utilization of alternative carbon sources: ribose, sucrose, salicin, beta-glucosides, lichenan, glucomannon, trehalose, G3P, etc. High expression in exponential phase on LB media

Subset of known regulon:
CcpA + SigA

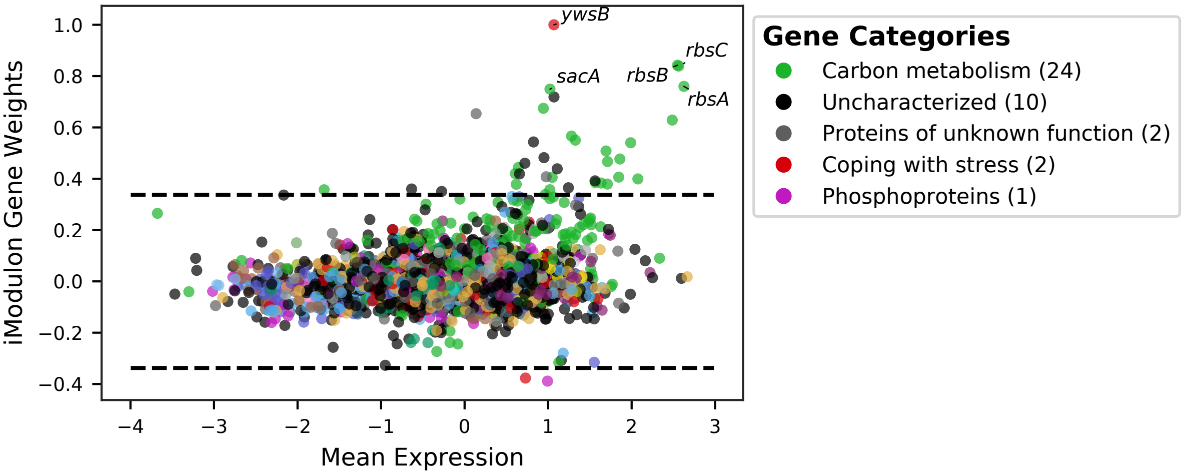

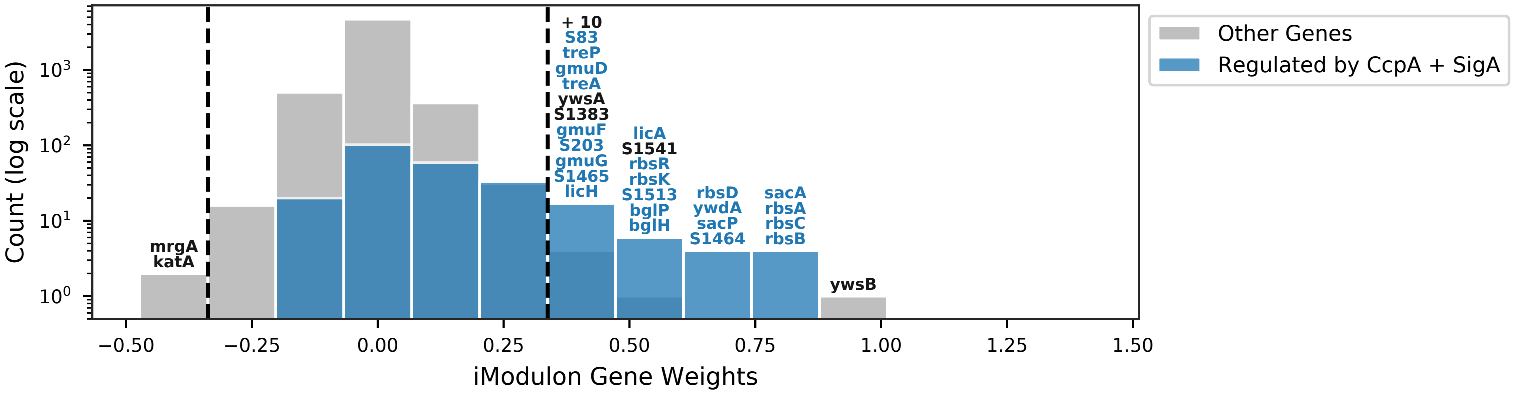

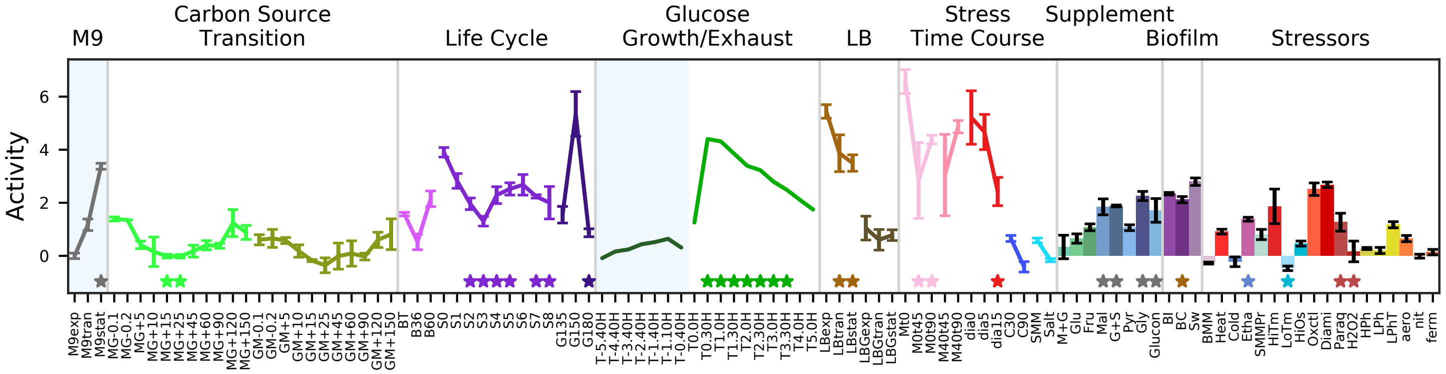

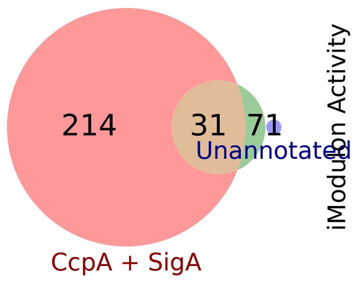

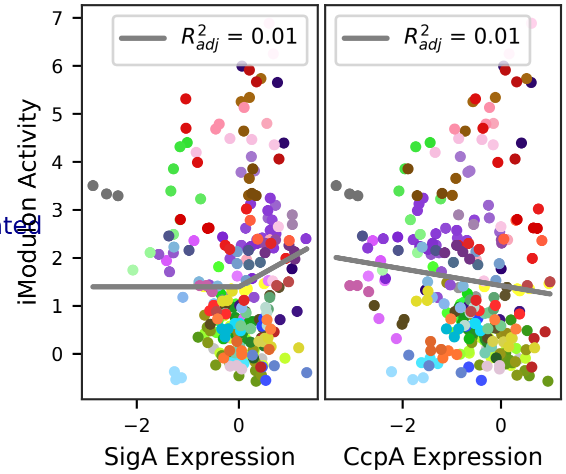

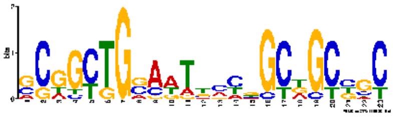

Motif E-value: 2.70e-04
Operons with Upstream Motif: 67%

10 - CcpA-2 – Low Glucose 2

Biological Function:
Uptake, utilization, and regulation of alternative carbon sources: TCA intermediates, ribose, maltodextrin, galacto-oligosaccharides, acetoin, gluconate, etc. Suppression of glucose pathways. Stationary phase.

Enriched for known regulon:
[CcpA + SigA] / [CcpN]

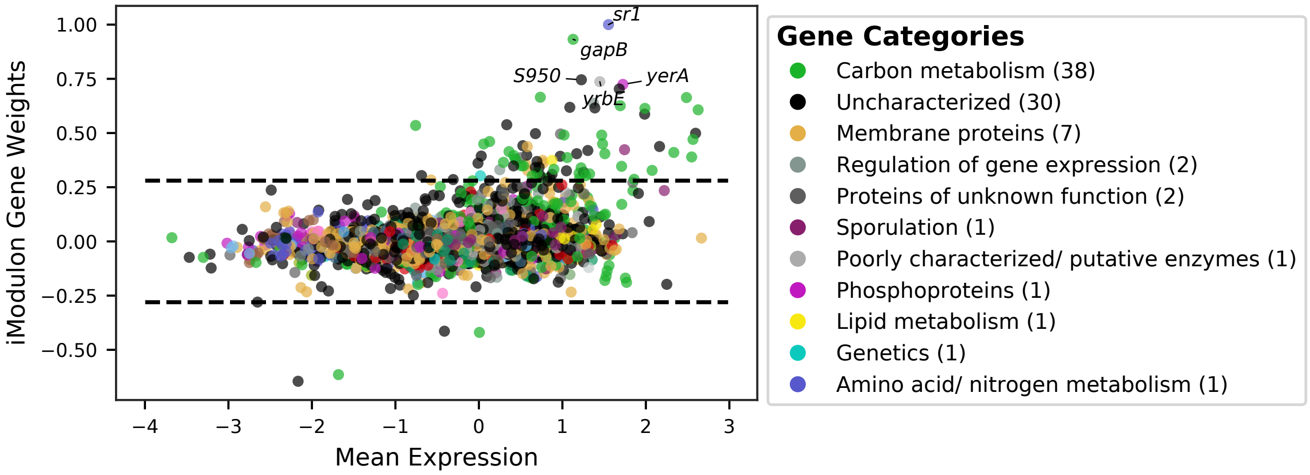

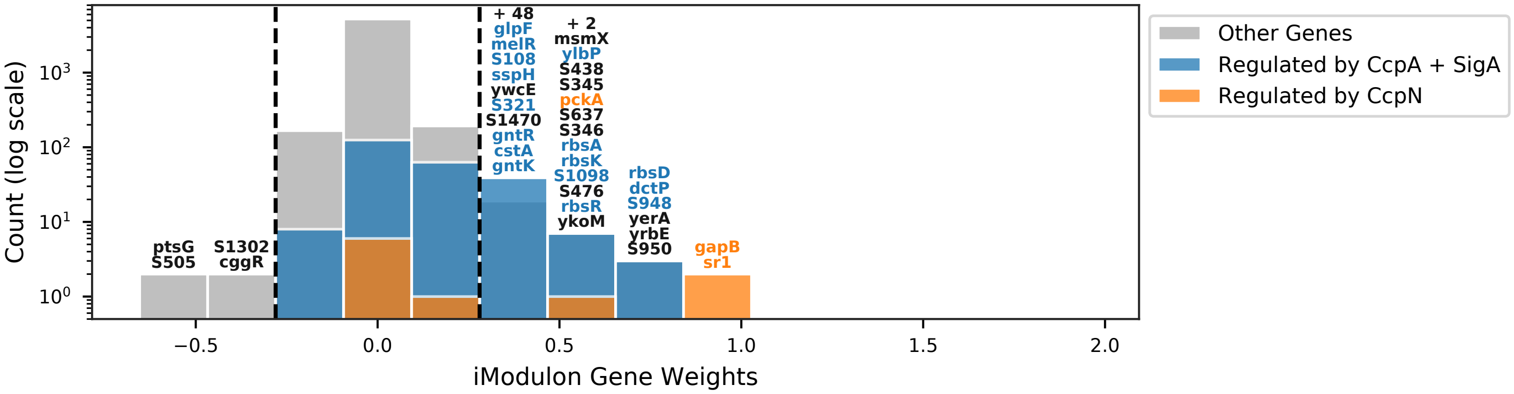

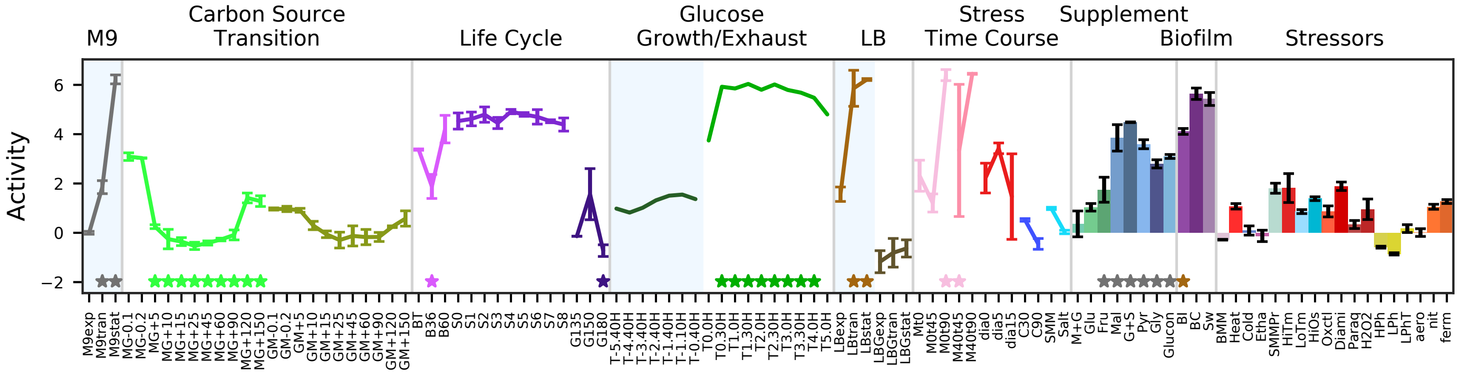

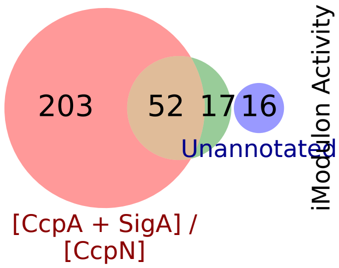

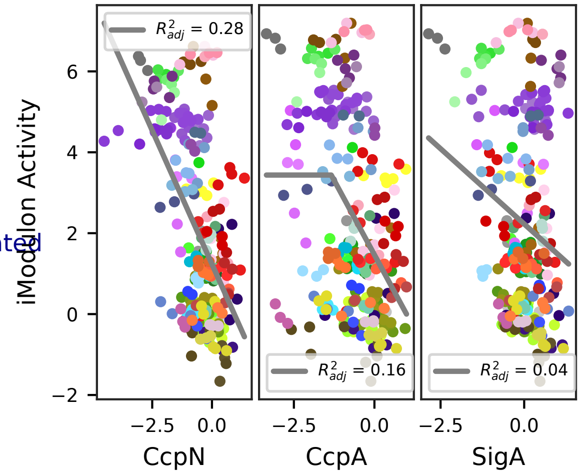

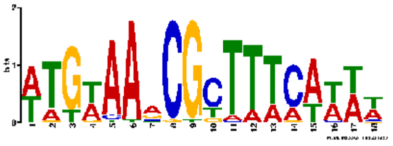

Motif E-value: 8.20e-07
Operons with Upstream Motif: 72%

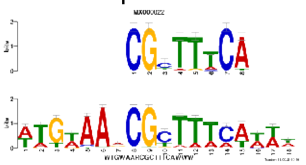

Motif similar to CcpA (E = 7.74e-05)

11 - His – Histidine Synthesis

Biological Function:
Synthesis of histidine

Subset (single operon) of known regulon:
YlxR

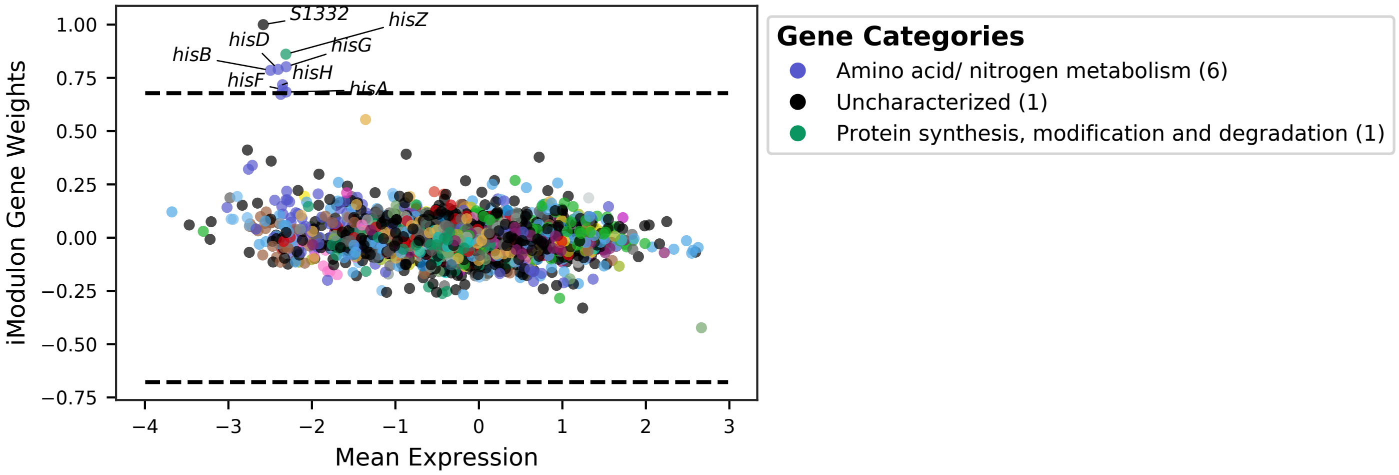

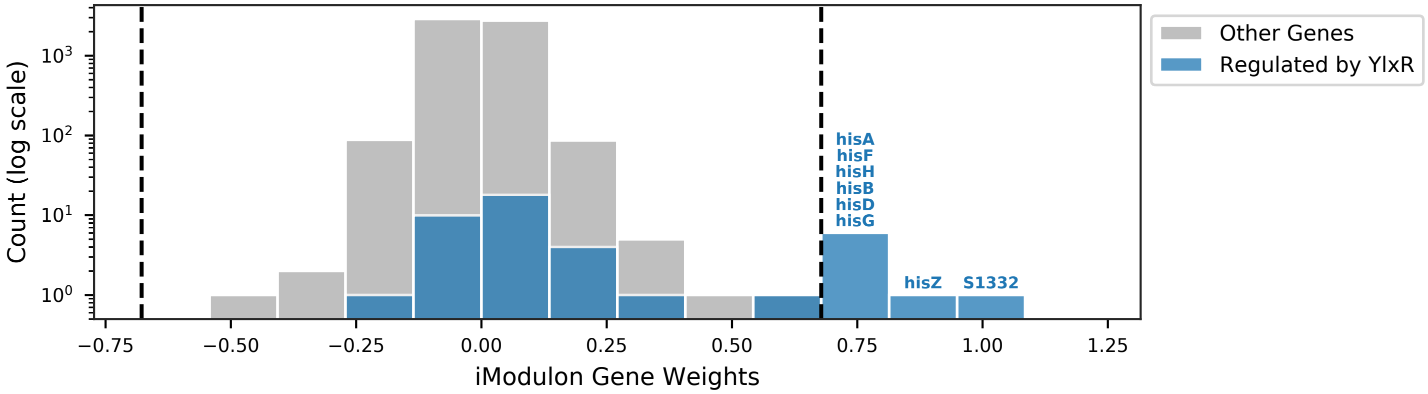

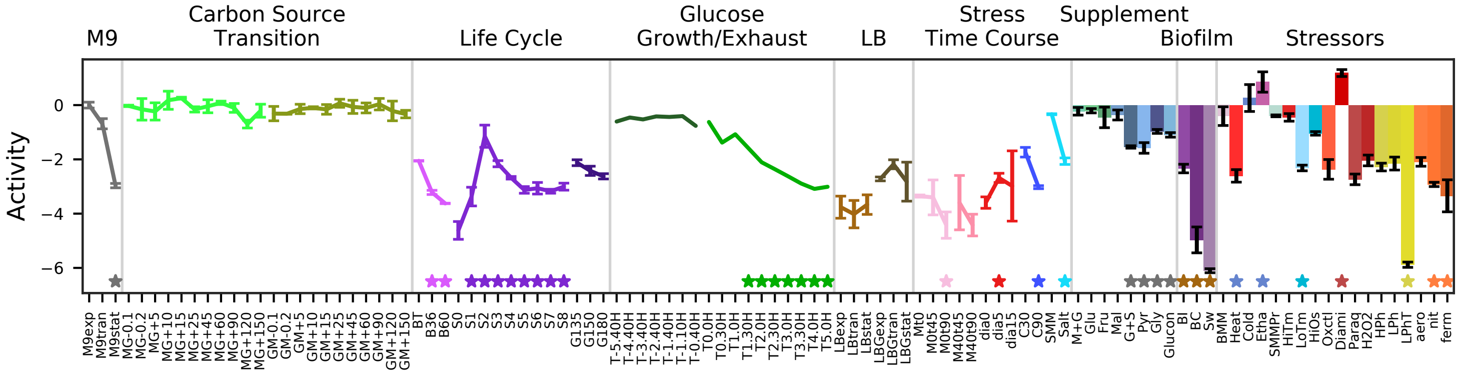

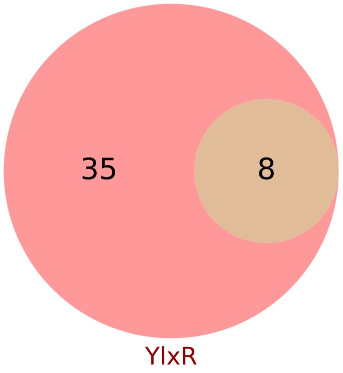

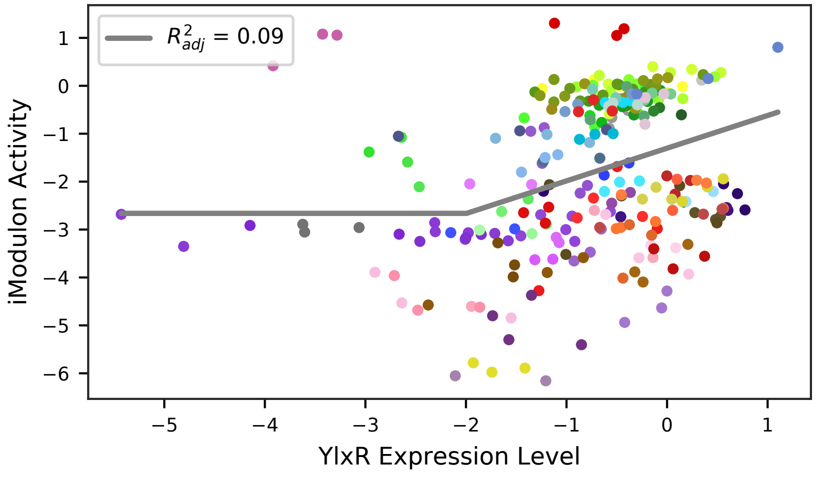

12 - AhrC – Arginine

Biological Function:
Uptake and biosynthesis of arginine

Well-defined regulon:
AhrC + YlxR

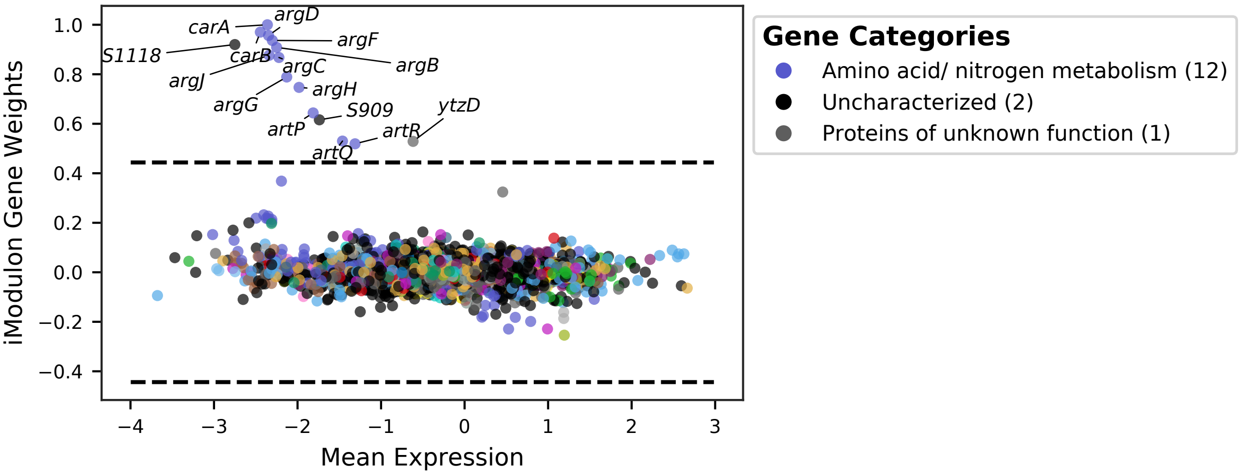

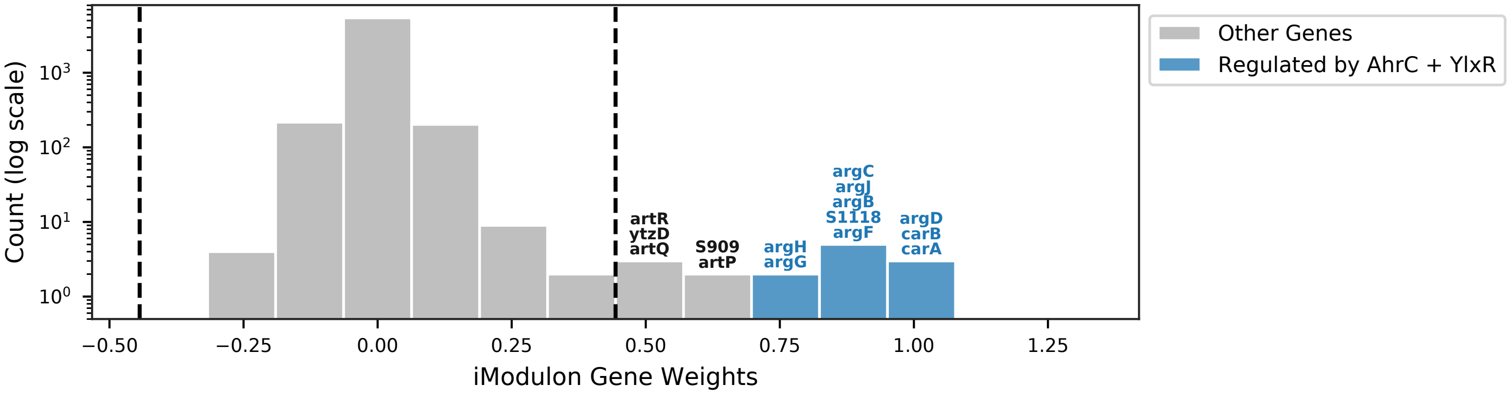

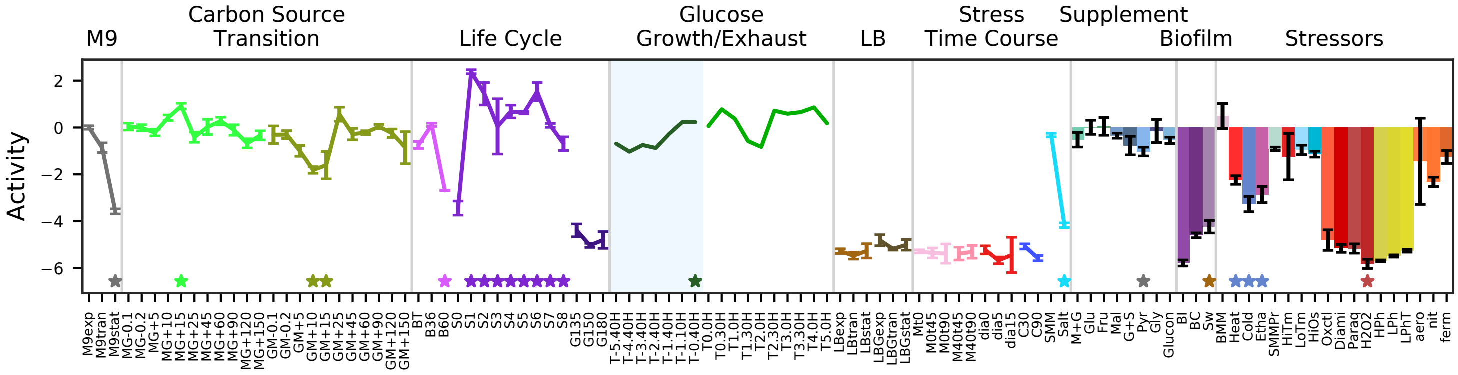

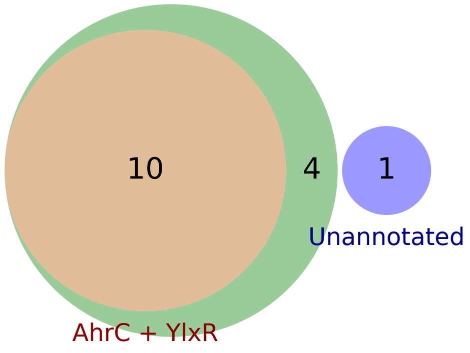

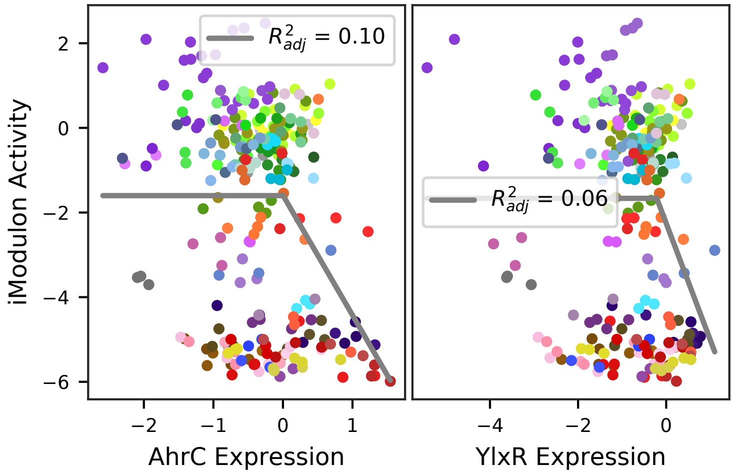

13 - MtrB – Tryptophan

Biological Function:
Biosynthesis of tryptophan

Subset (single operon) of known regulon:
MtrB

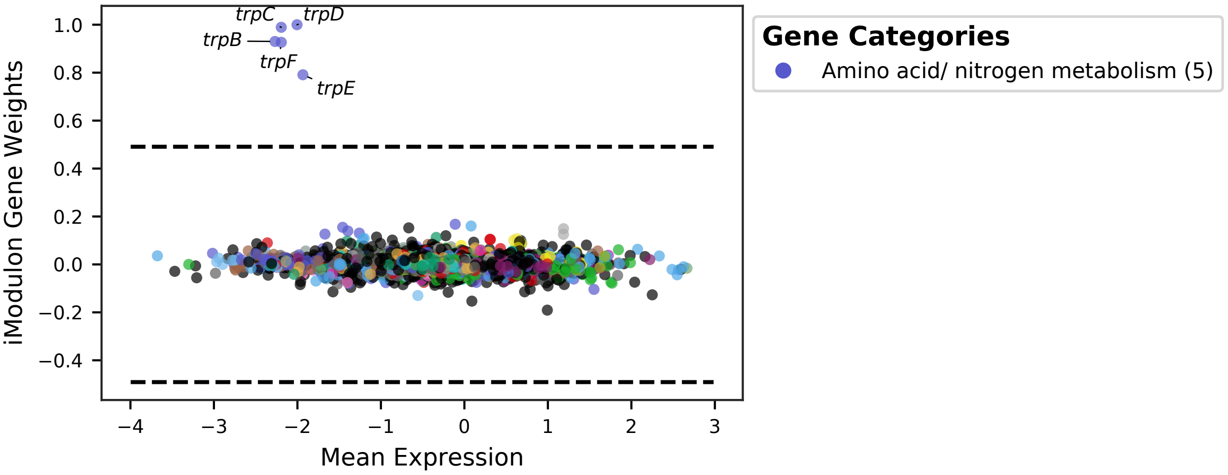

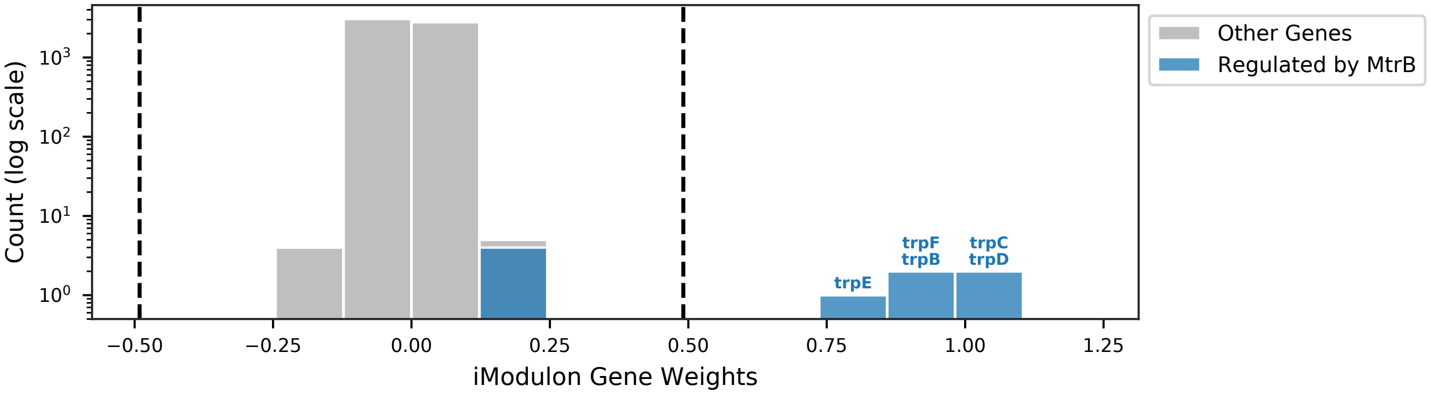

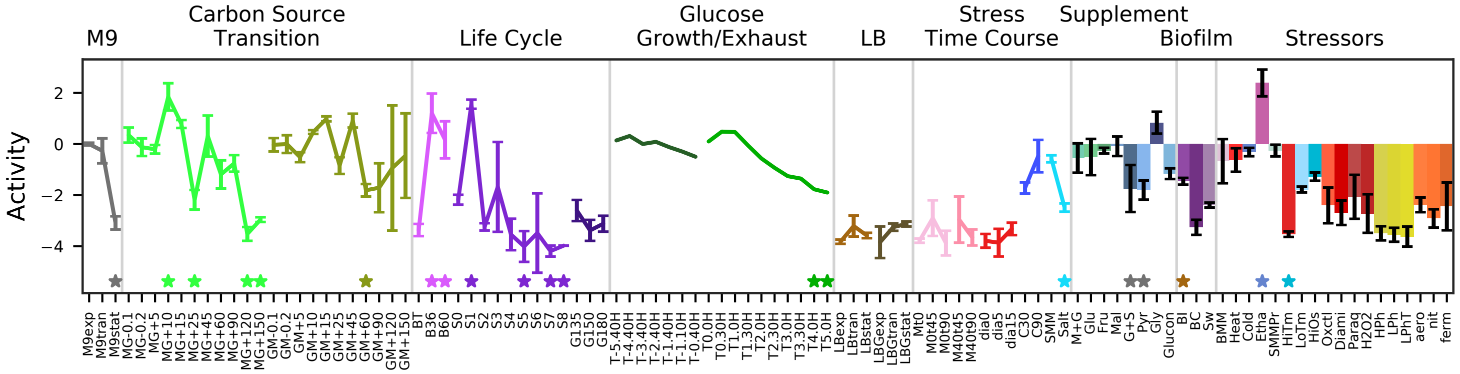

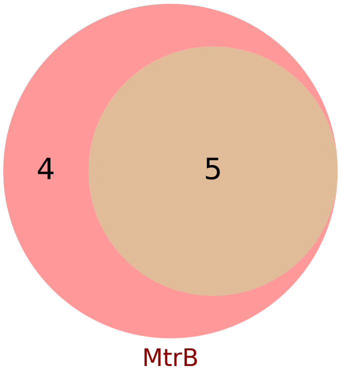

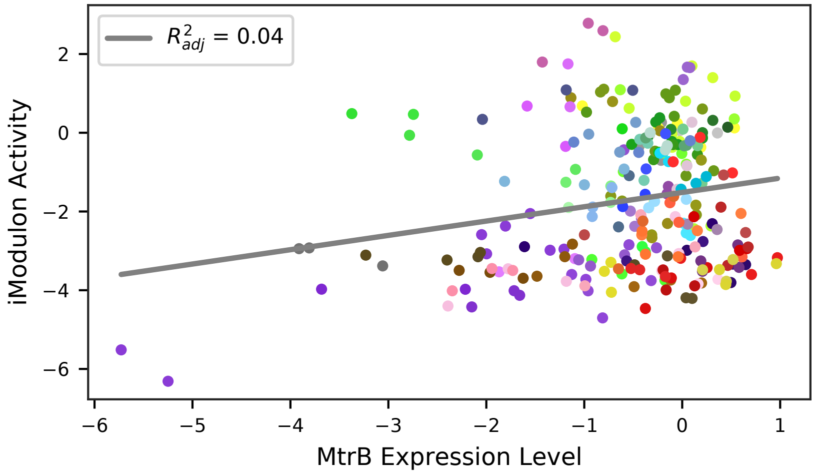

14 - S-Box – Methionine

Biological Function:
Methionine salvage and biosynthesis

Subset of known regulon:
S-box

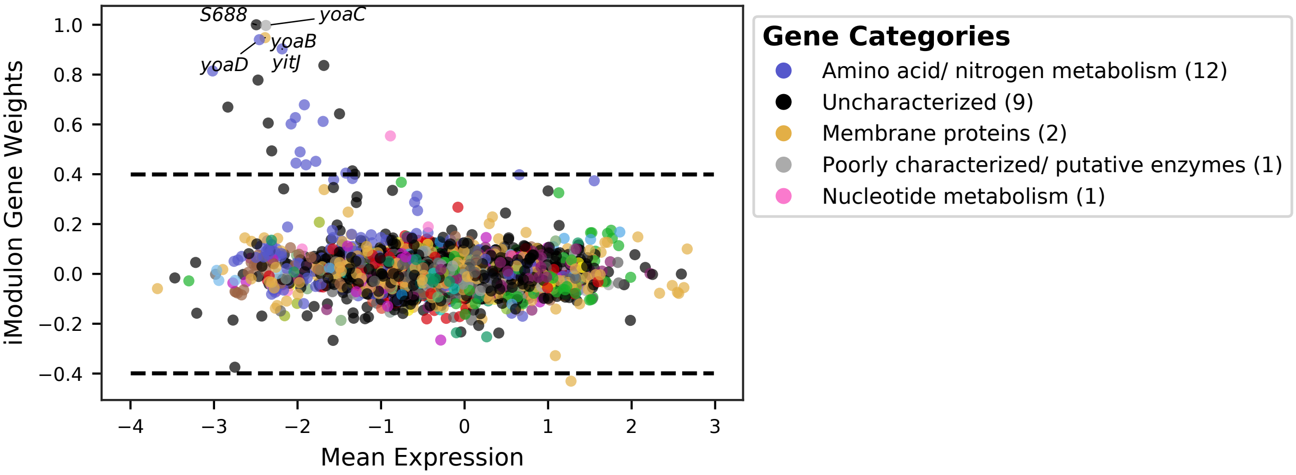

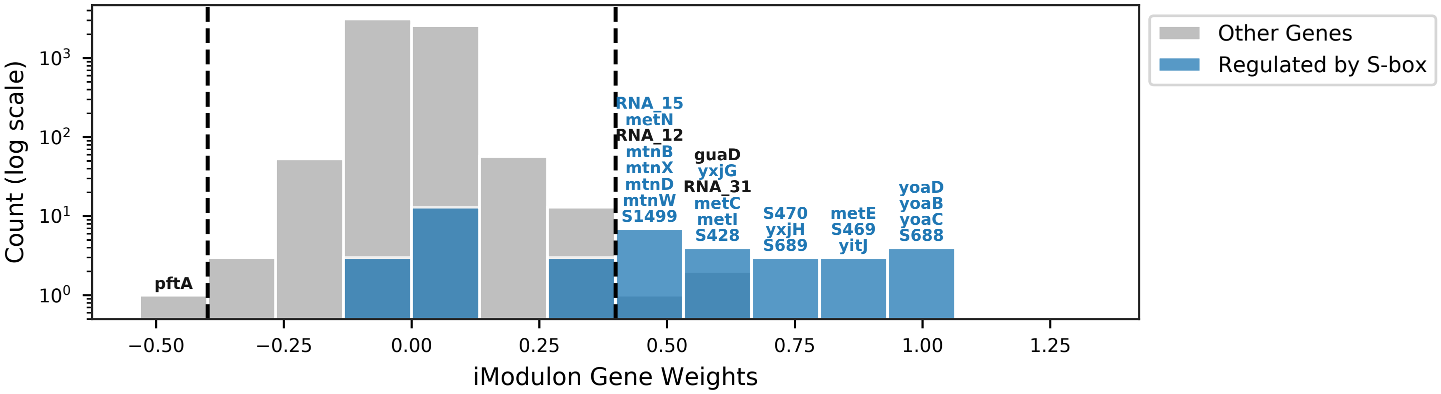

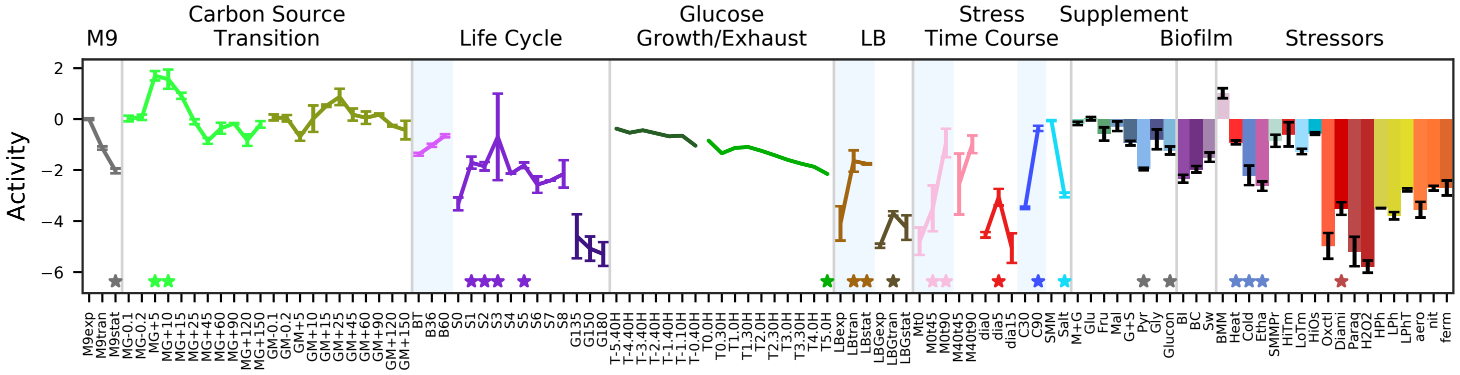

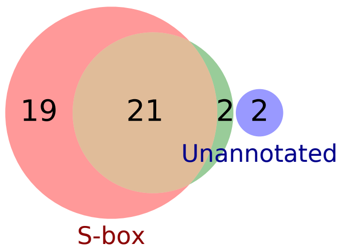

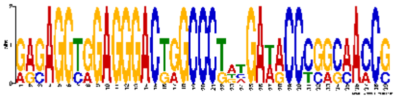

Motif E-value: 1.10e-15
Operons with Upstream Motif: 40%

15 - TnrA + CodY – Leucine, Methionine, and Threonine

Biological Function:
Synthesis and salvage of leucine, methionine, threonine

Contains unknown genes and known regulon:
[TnrA + CodY + ThrR] / [TnrA + CodY + T-box + CcpA + SigA]

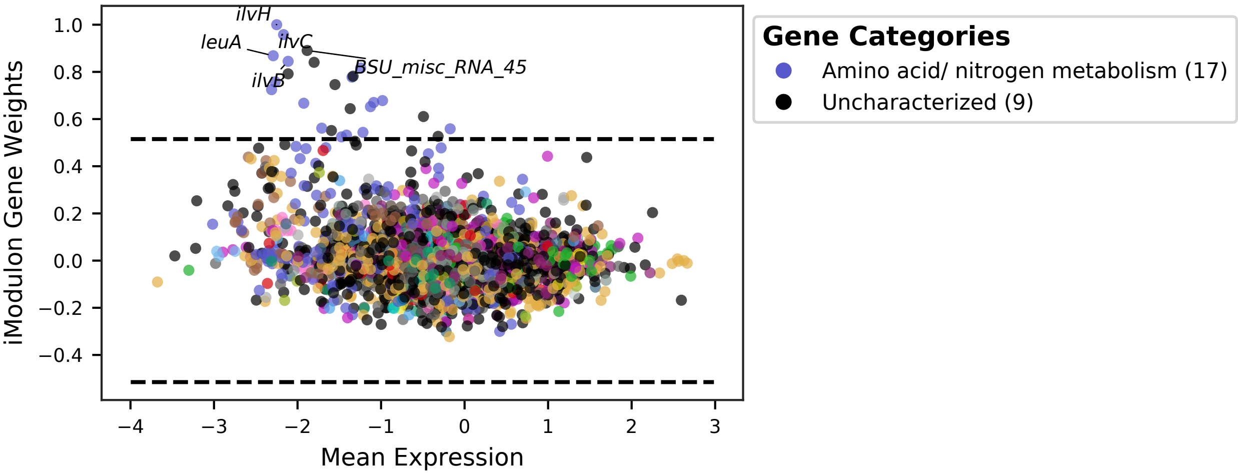

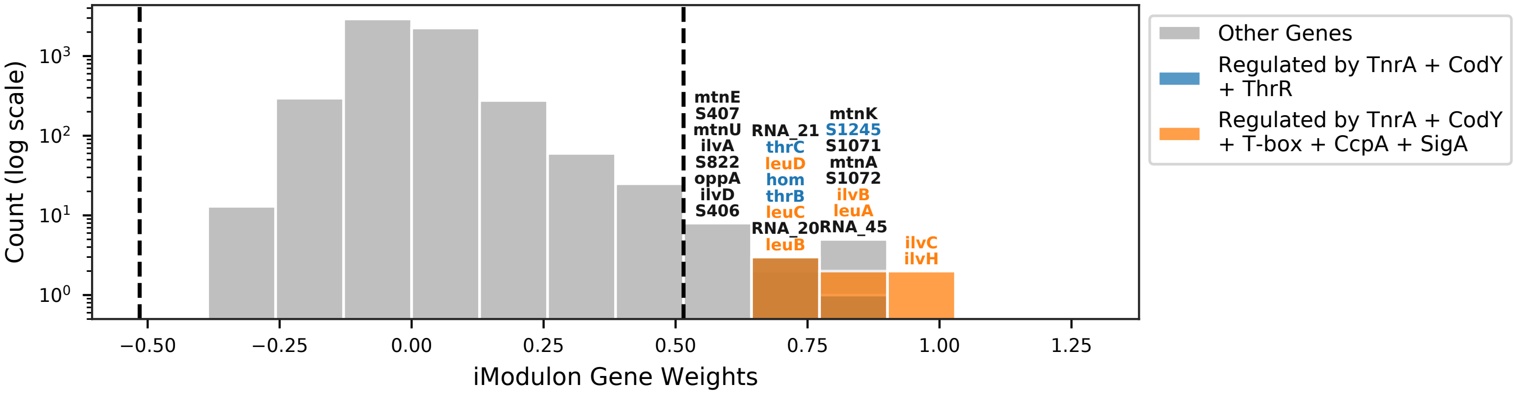

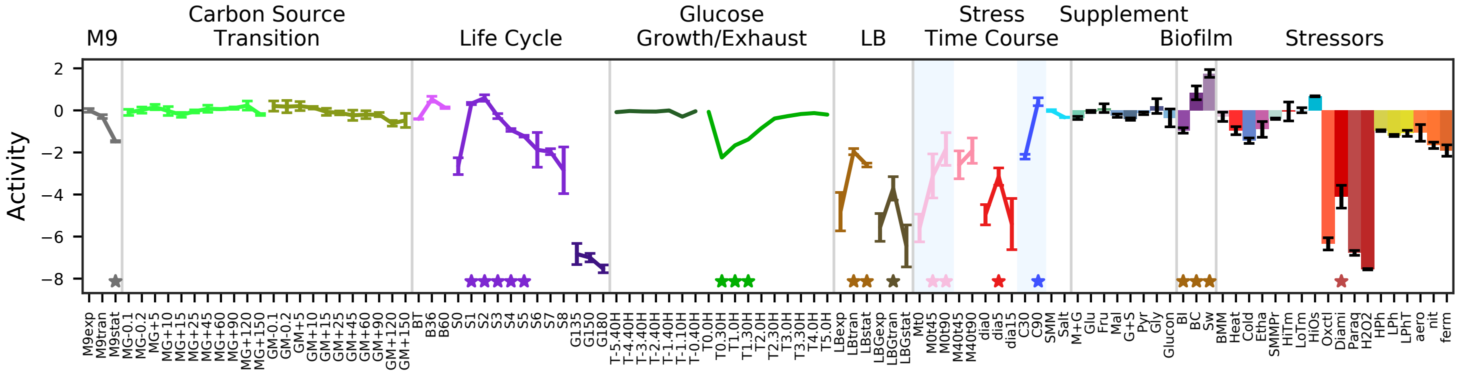

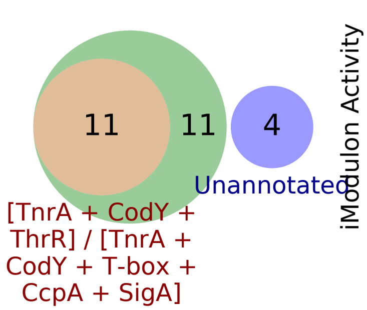

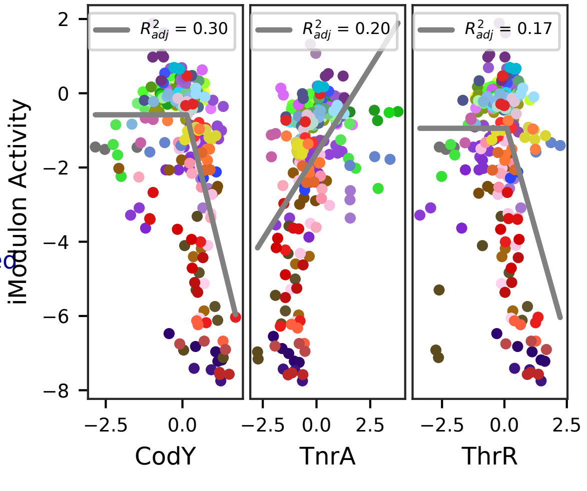

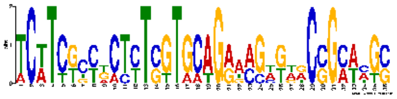

Motif E-value: 2.70e-05
Operons with Upstream Motif: 62%

16 - CodY – BCAA Limitation

Biological Function:
Uptake of di-/oligopeptides, uptake of guanosine, cell wall metabolism

Subset of known regulon:
CodY

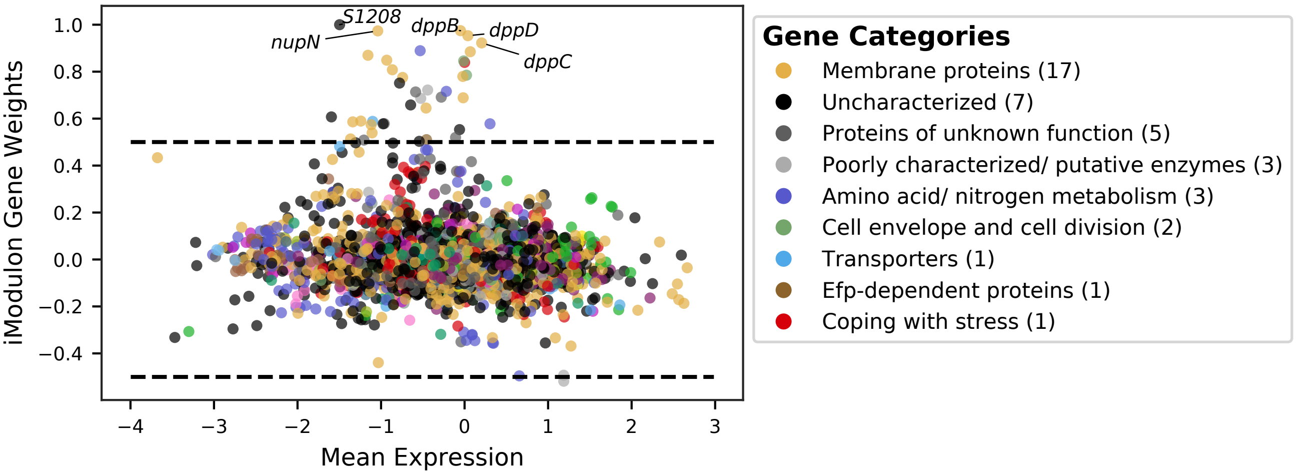

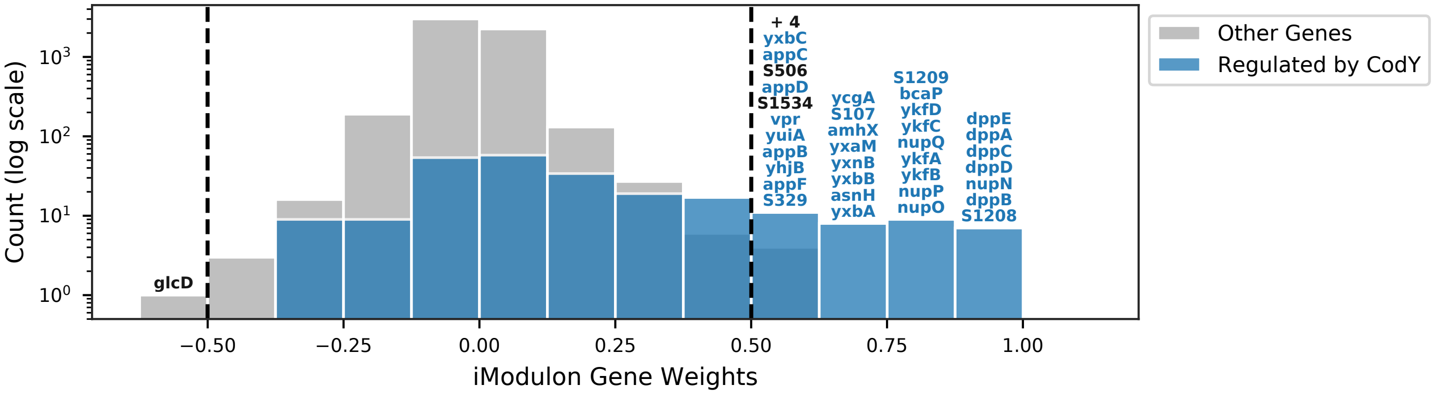

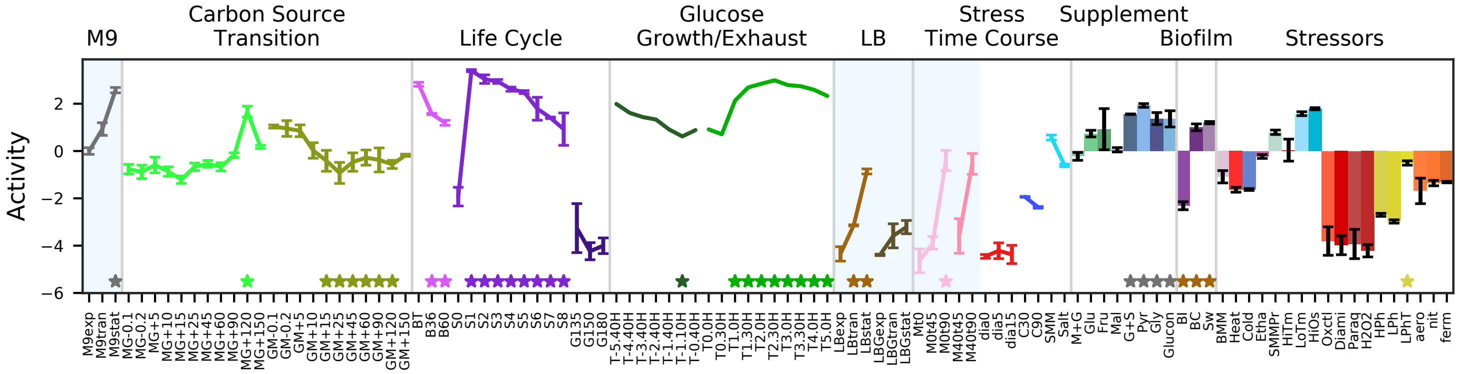

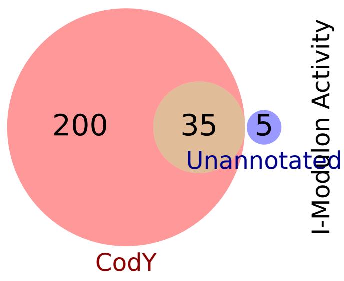

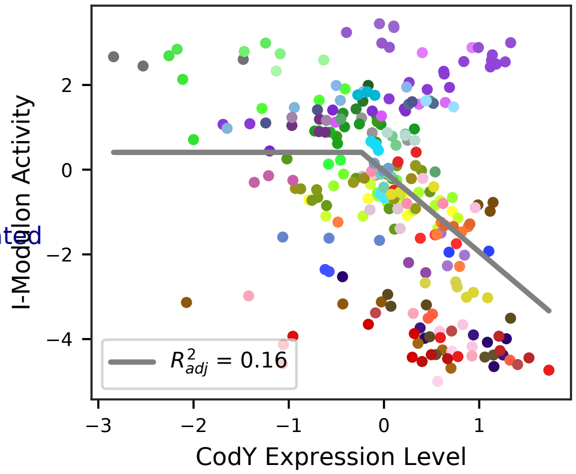

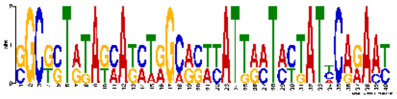

Motif E-value: 2.90e-09
Operons with Upstream Motif: 35%

17 - HutP – Histidine Utilization

Biological Function:
Histidine uptake and utilization

Well-defined regulon:
HutP

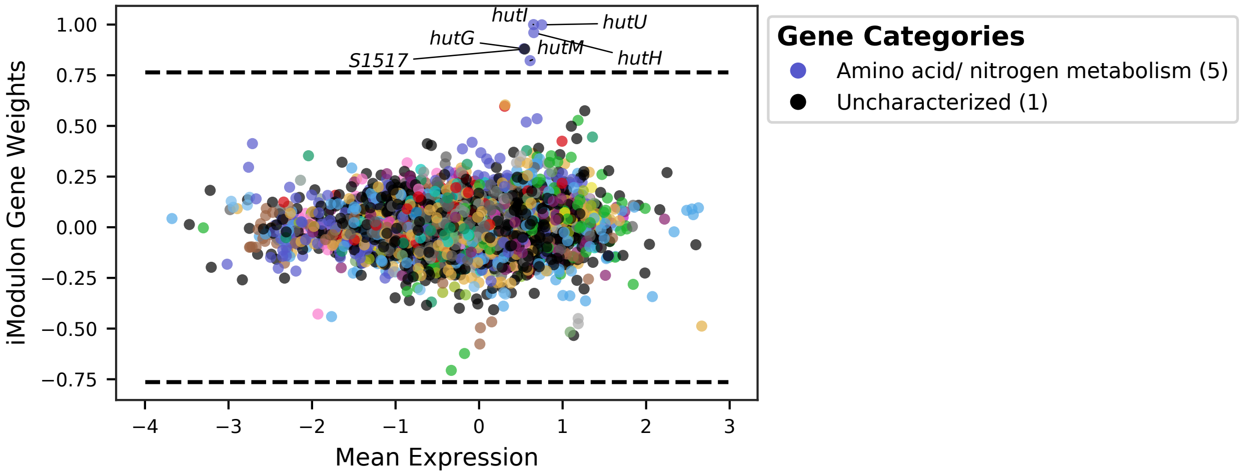

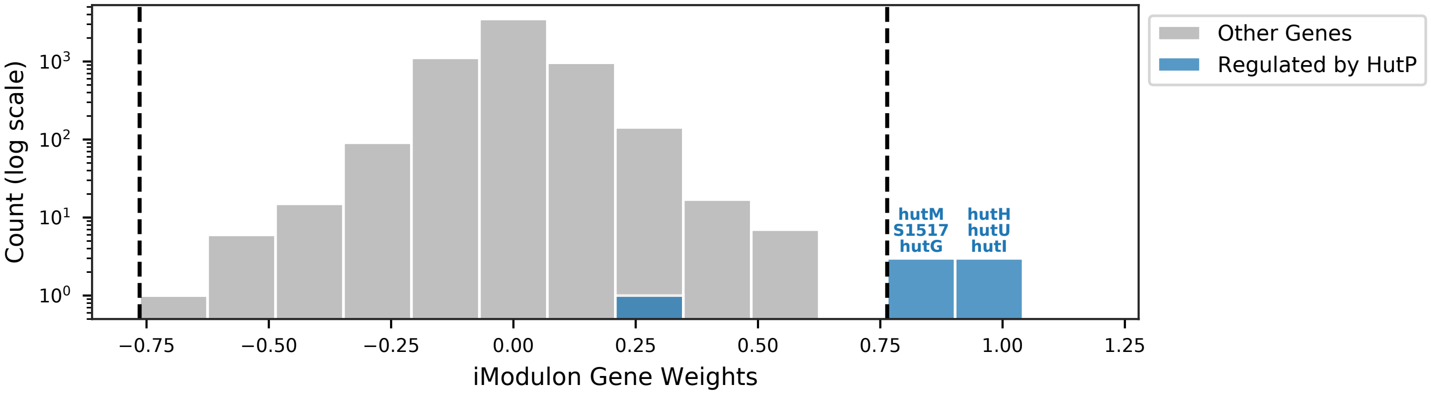

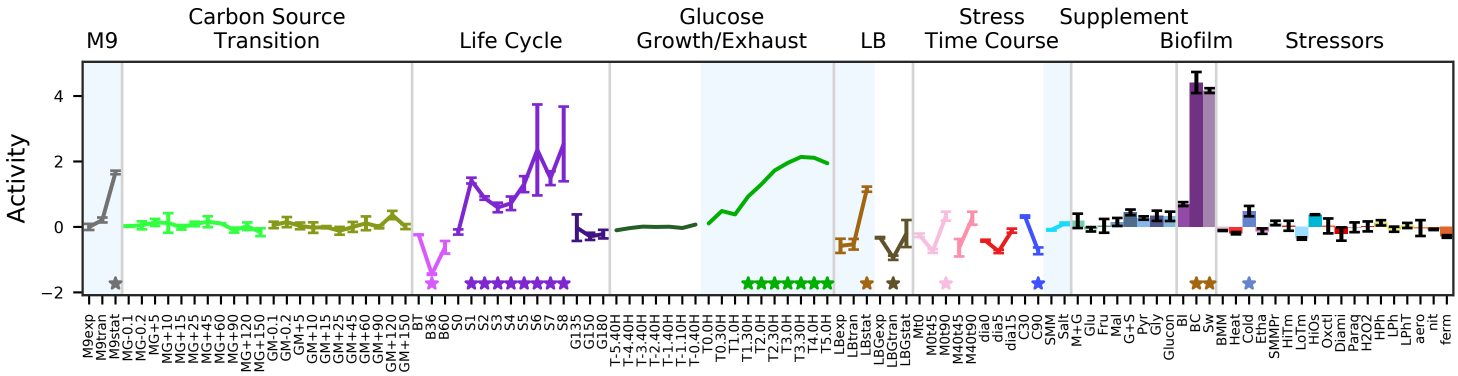

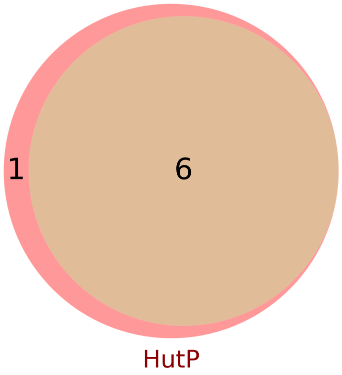

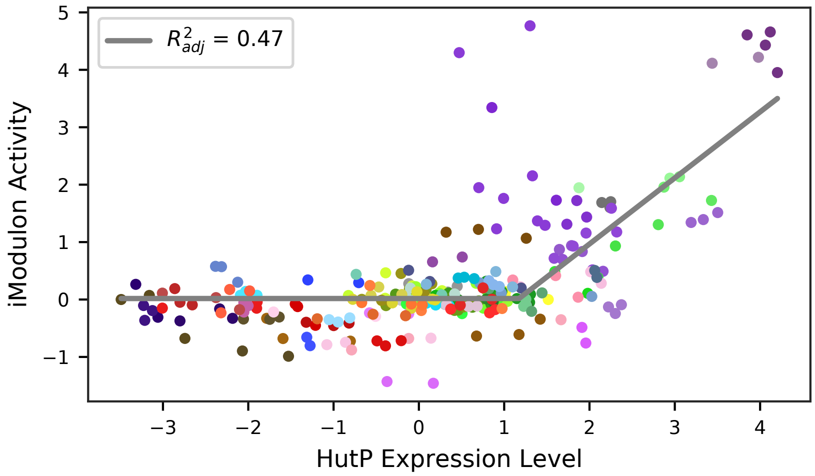

18 - RocR / PutR – Arginine and Proline Utilization

Biological Function:
Utilization of arginine, ornithine, citrulline and proline as carbon and energy sources

Well-defined regulon:
[SigL + AhrC + RocR] / [Spo0A + SigA + CodY + PutR]

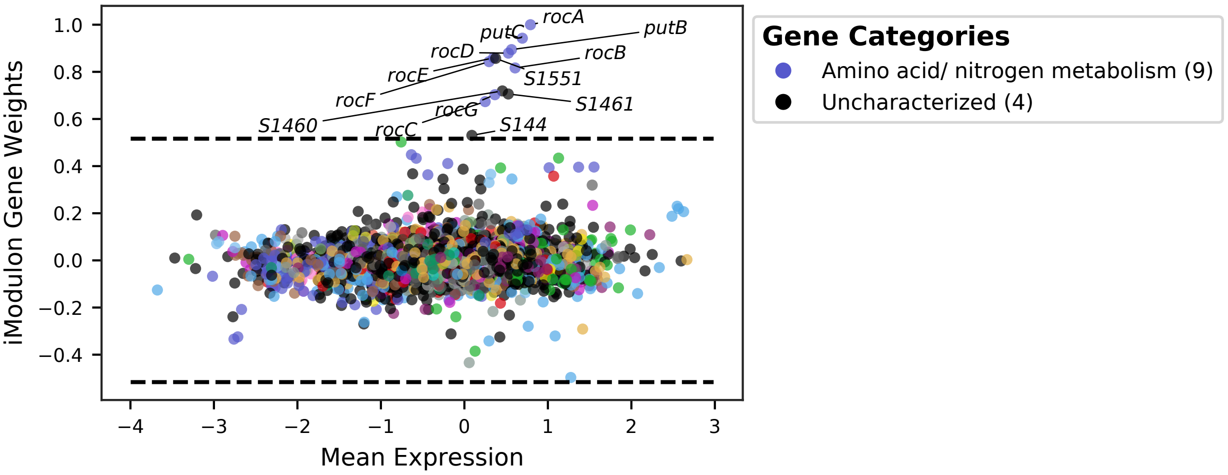

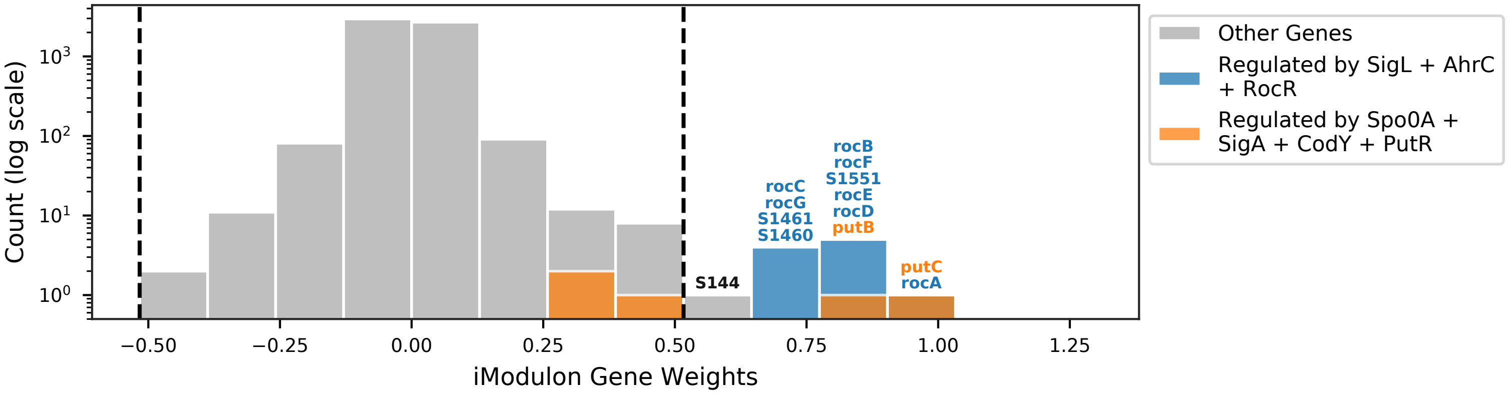

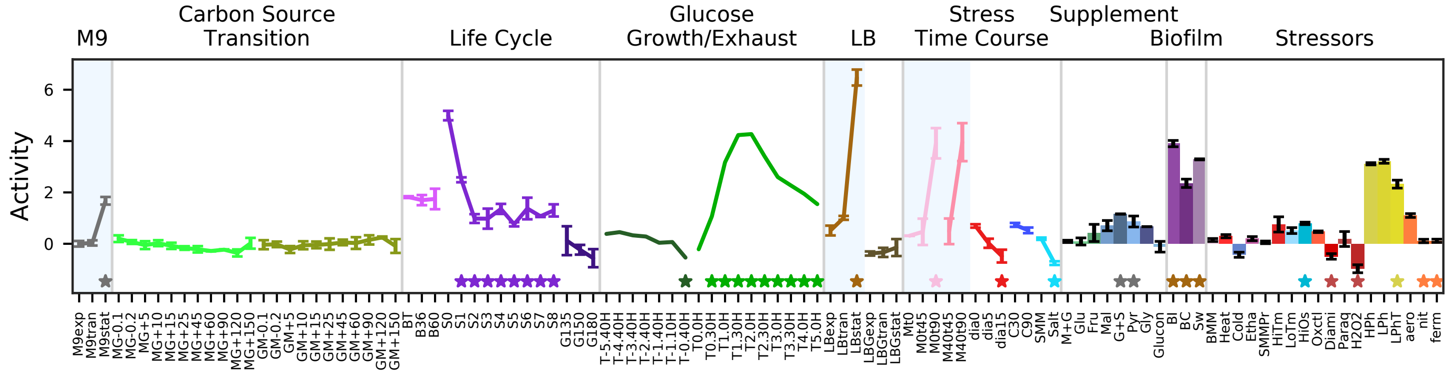

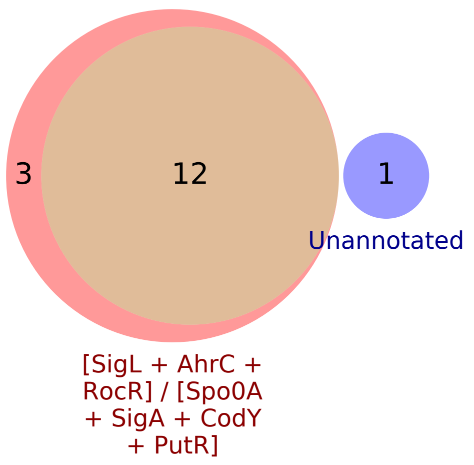

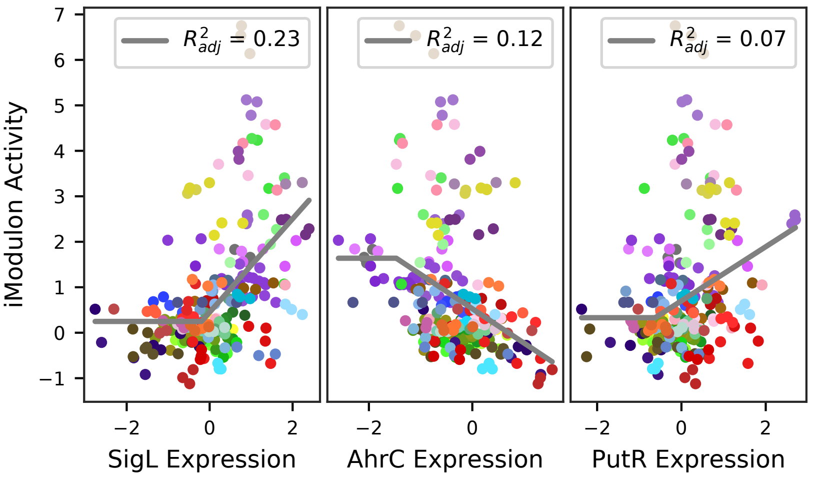

19 - PyrR – Pyrimidines

Biological Function:
Synthesis of pyrimidines

Well-defined regulon:
PyrR

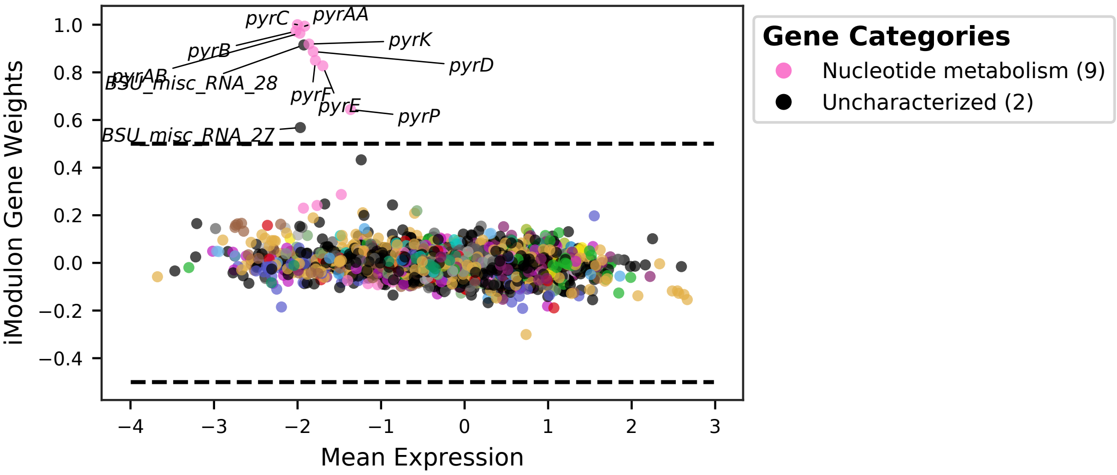

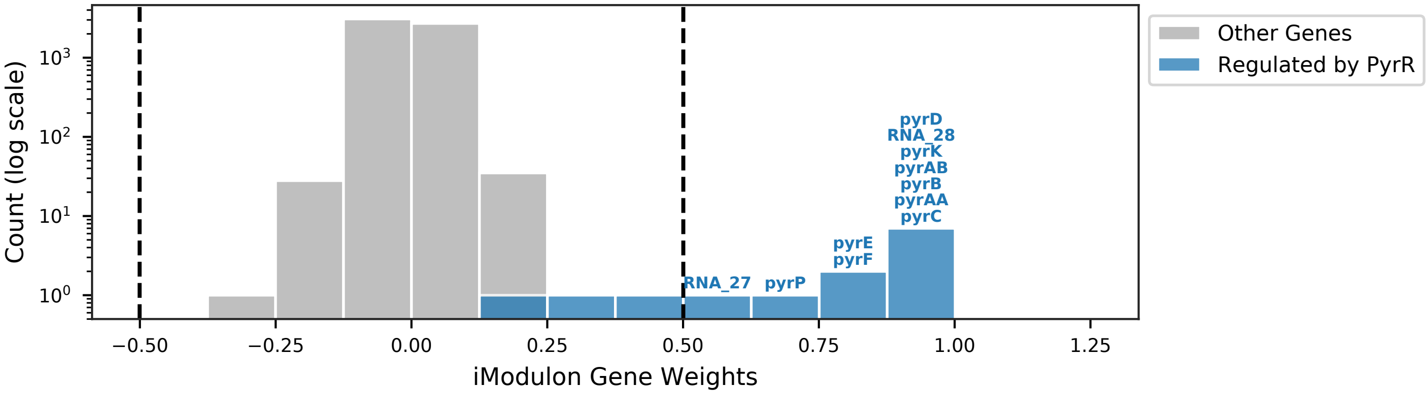

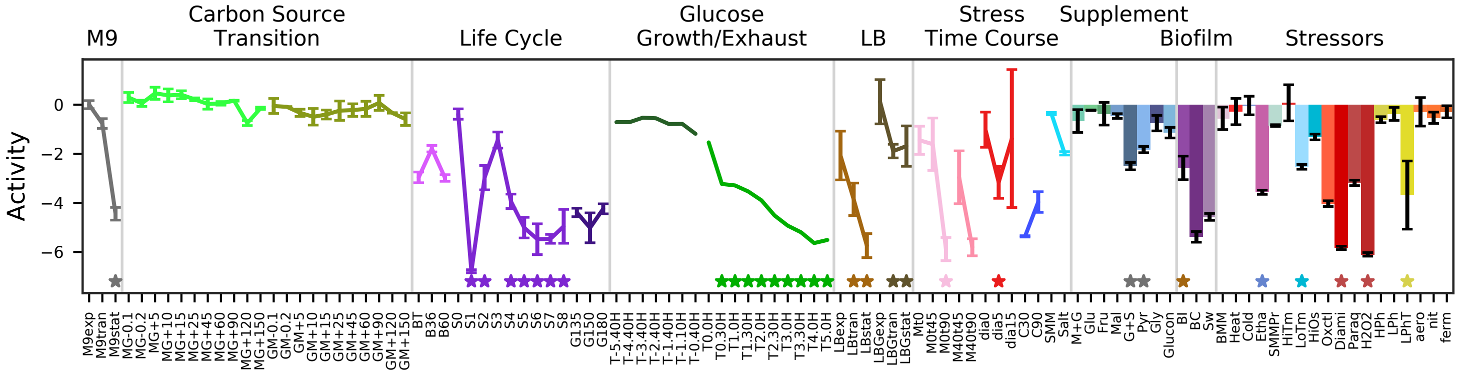

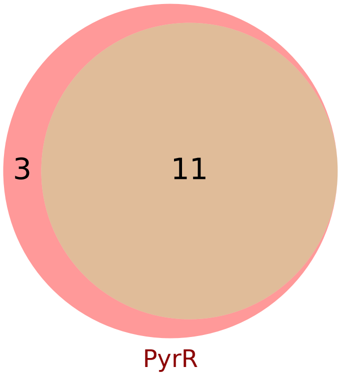

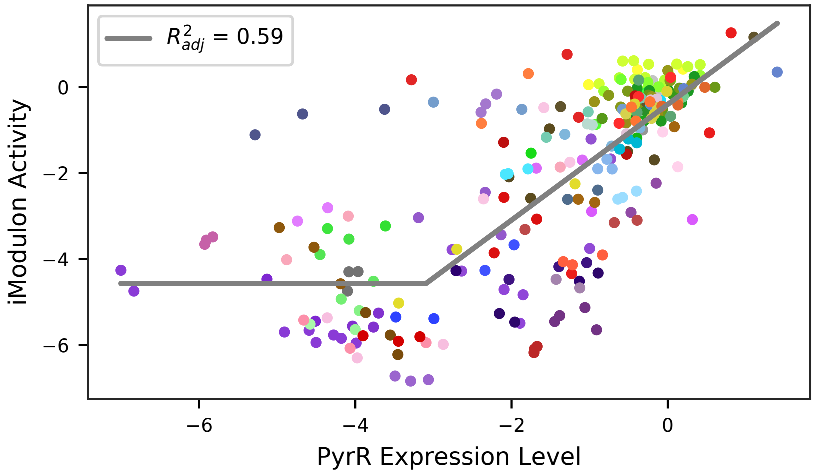

20 - PurR – Purines

Biological Function:
Synthesis of purine, xanthine and guanine uptake and conversion into purines

Well-defined regulon:
PurR

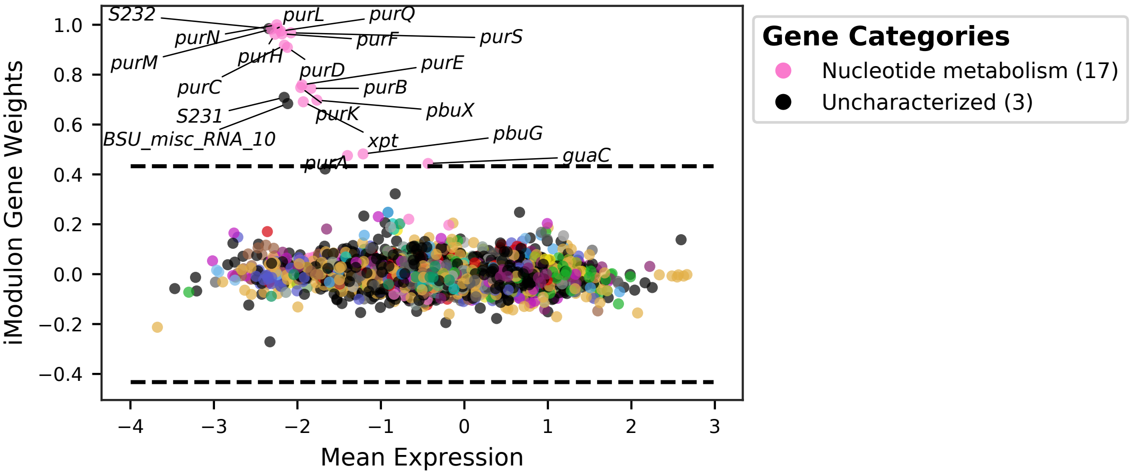

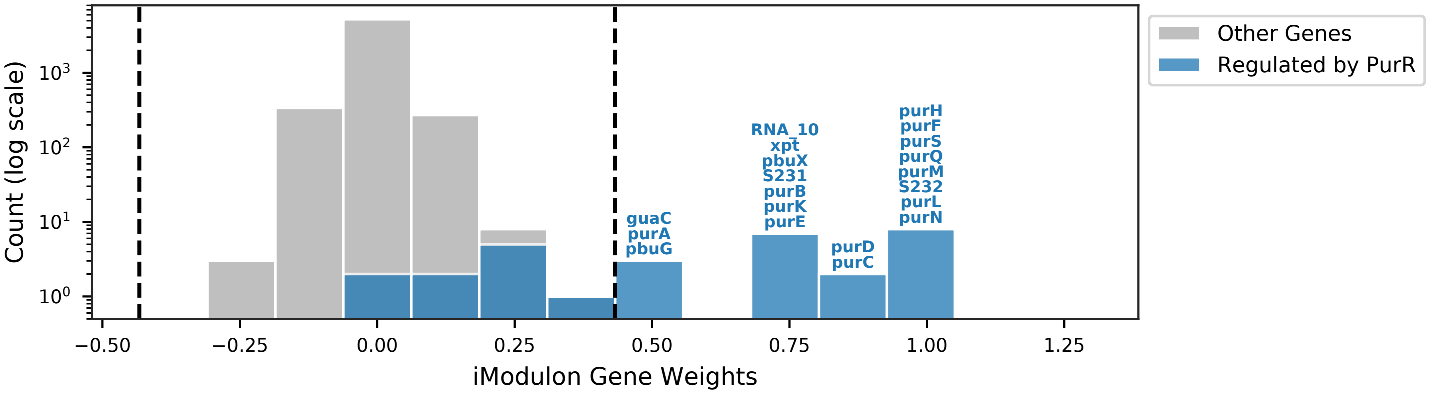

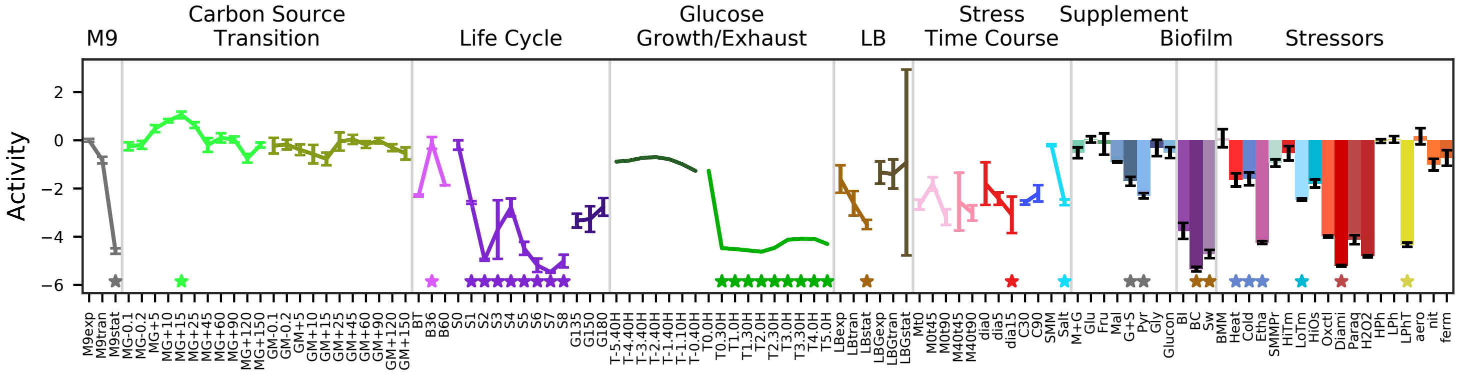

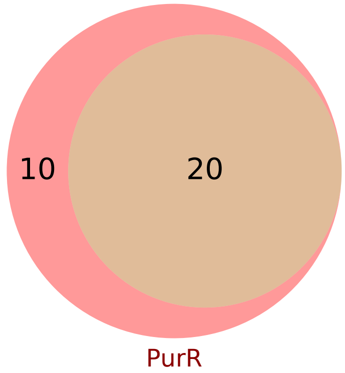

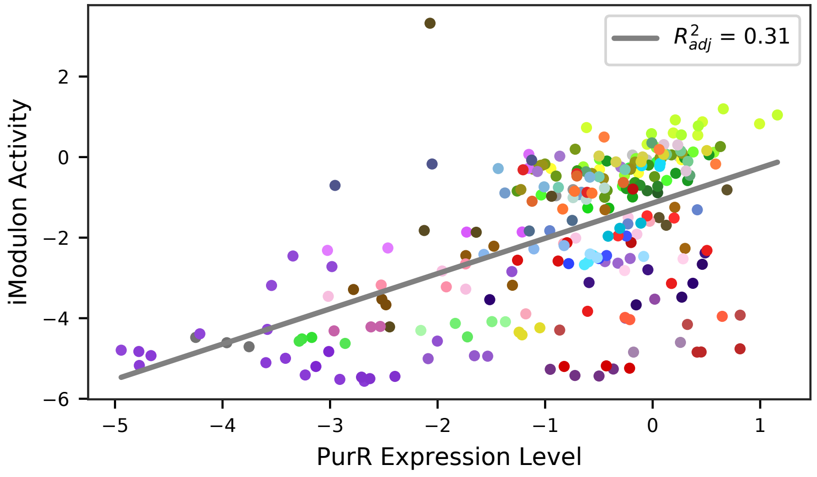

21 - Thi-Box – Thiamine

Biological Function:
Synthesis and uptake of thiamine

Well-defined regulon:
Thi-box

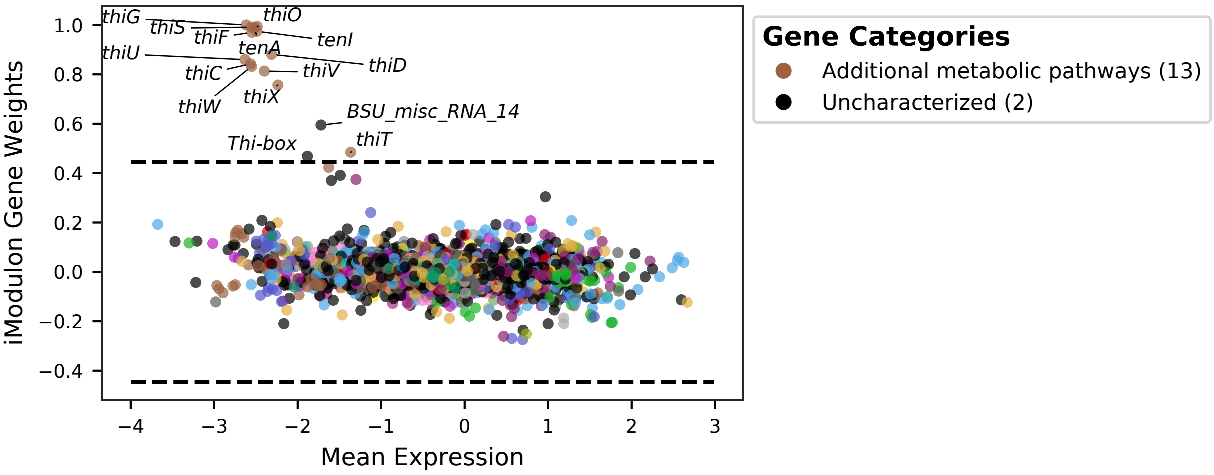

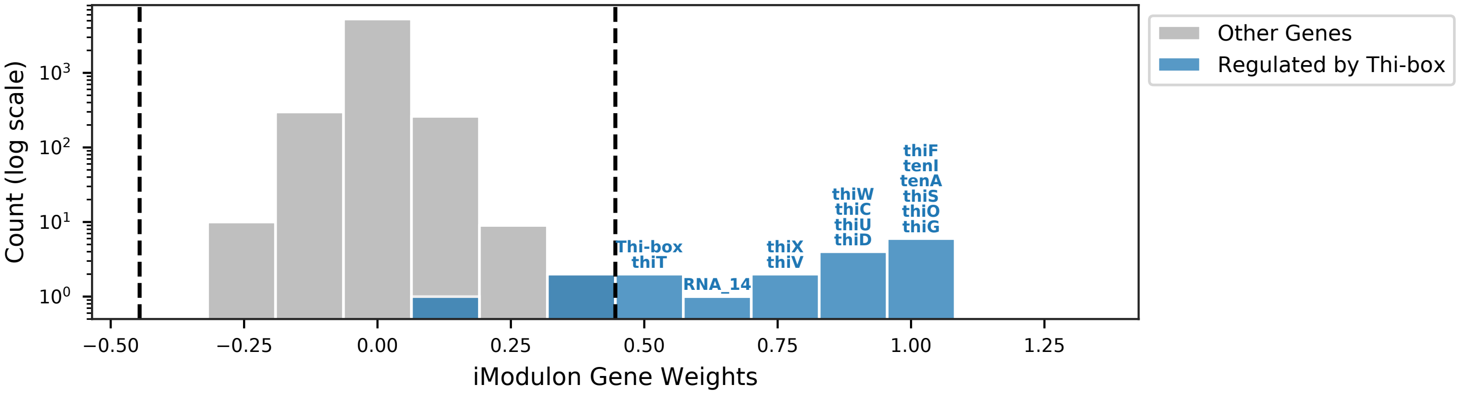

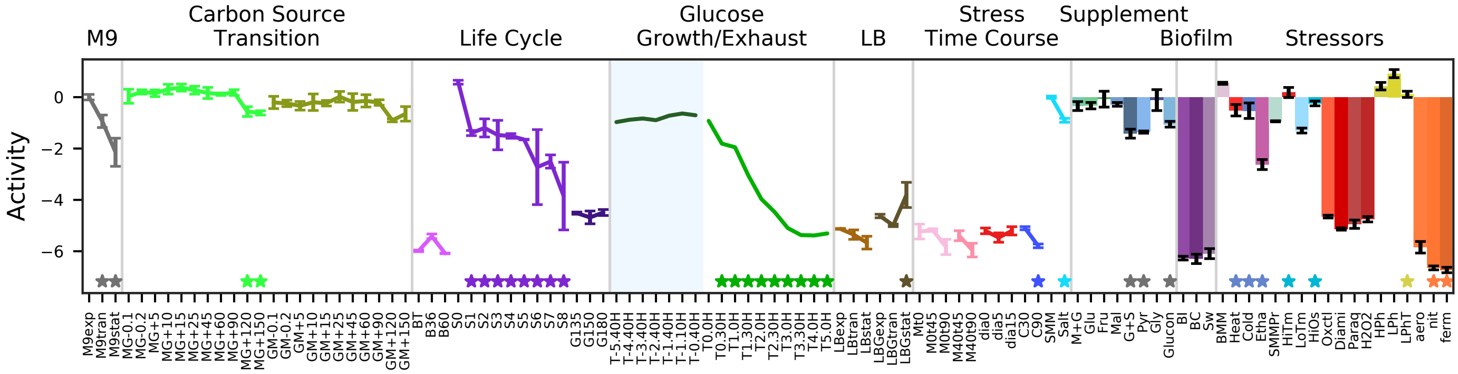

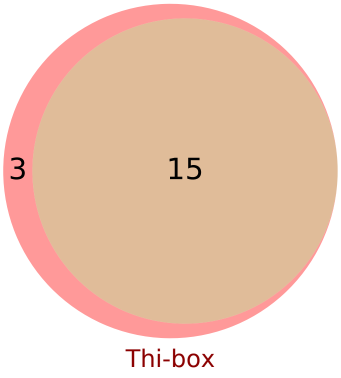

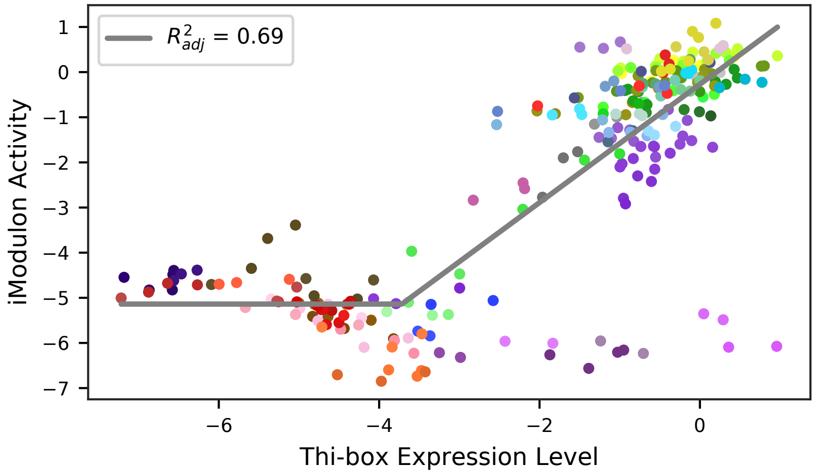

22 - PchR – Pulcherrimin

Biological Function:
Extracellular synthesis and import of pulcherrimin for iron acquisition

Subset of known regulon:
PchR

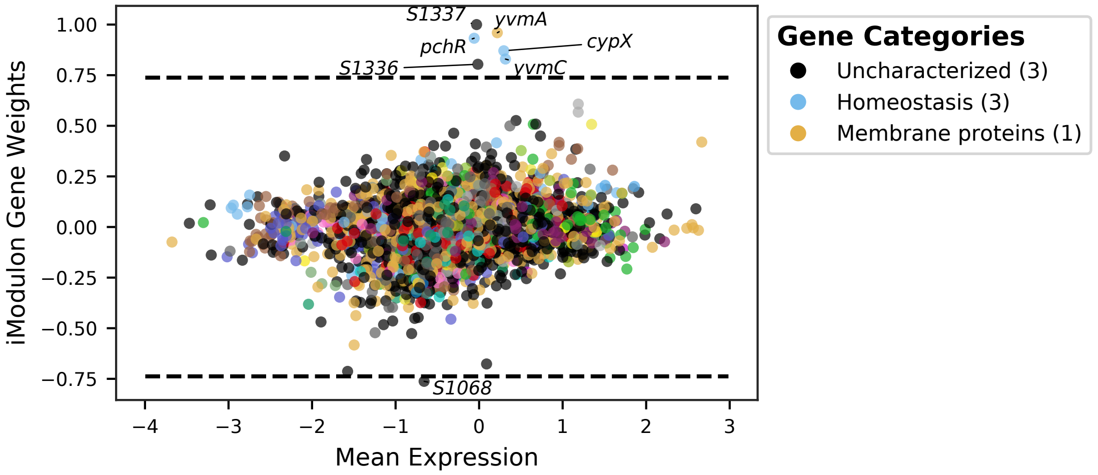

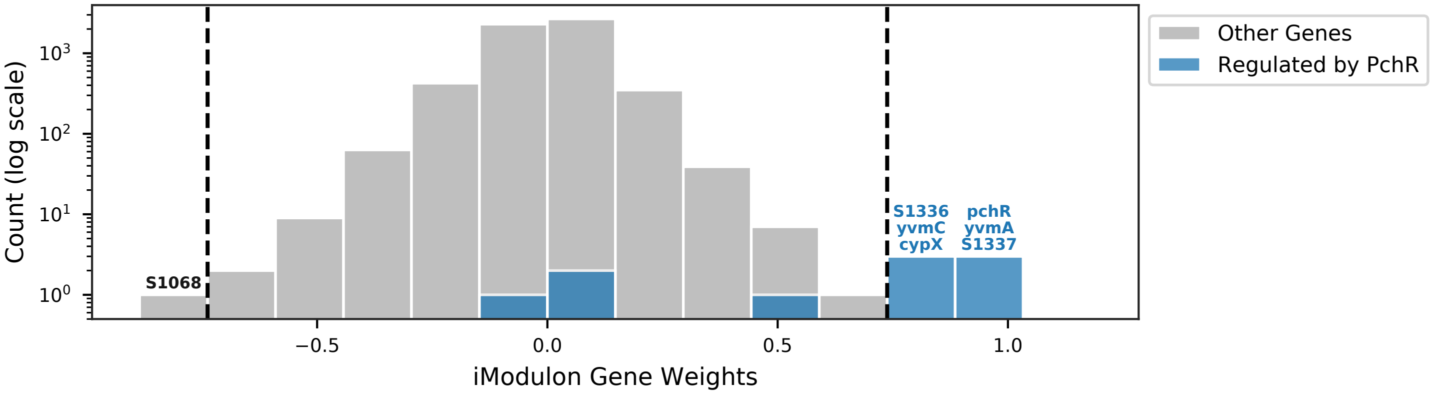

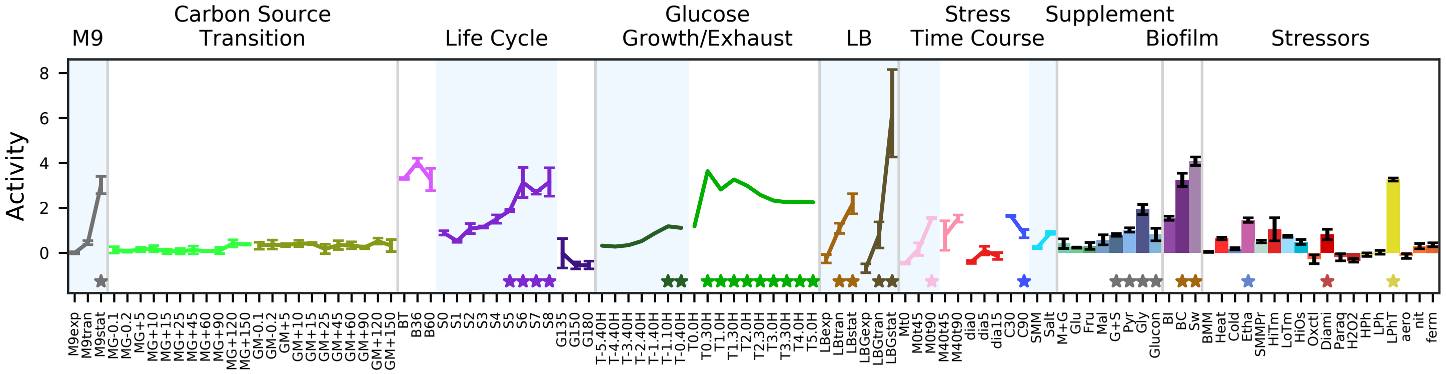

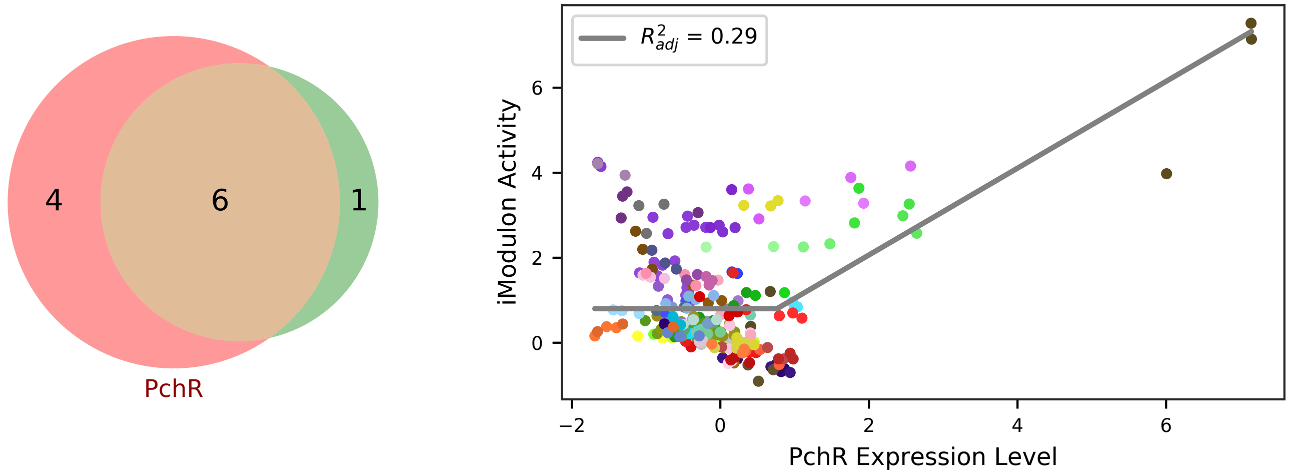

23 - Fur – Iron Limitation

Biological Function:
Production and transport of siderophores for iron chelation, replacement of ferredoxins in the ETC with flavodoxins, RNA chaperones for iron-limited conditions, heme degradation

Subset of known regulon:
Fur

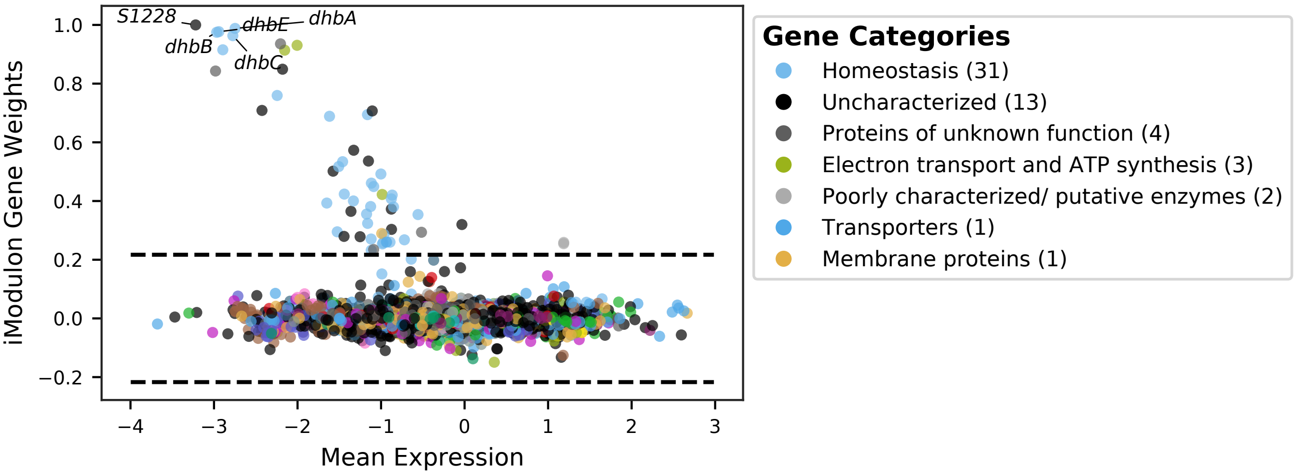

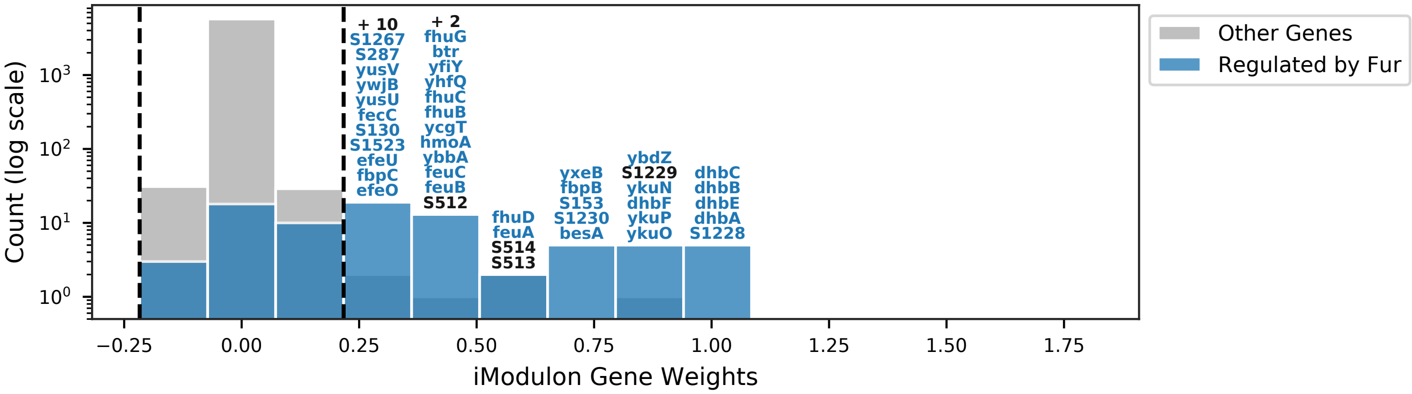

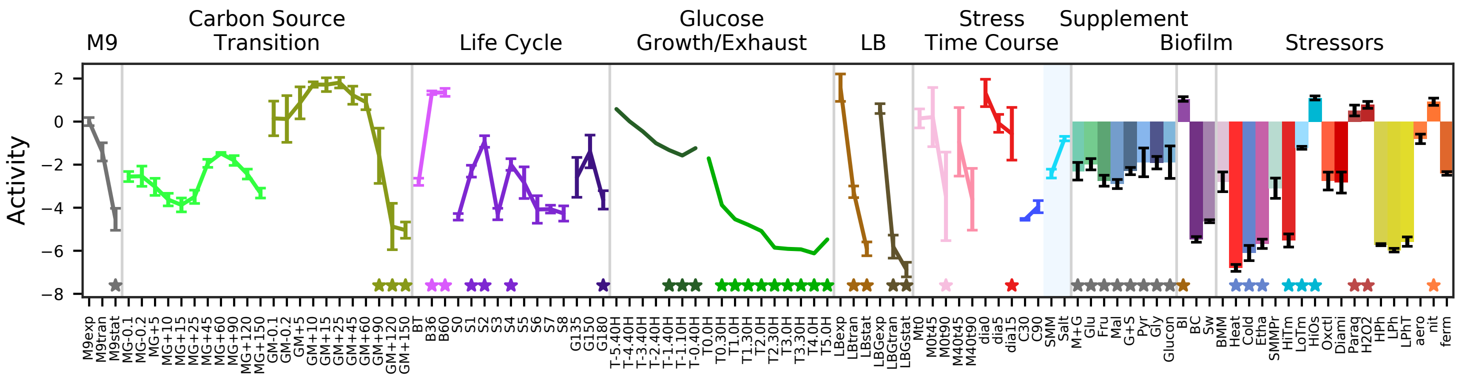

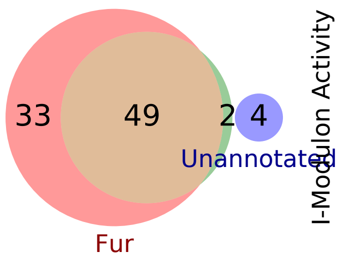

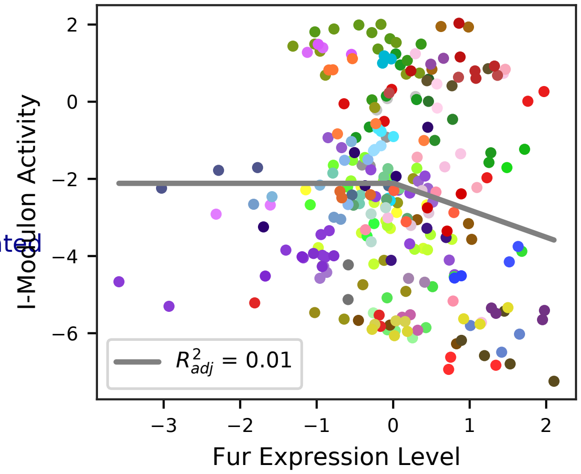

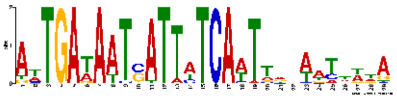

Motif E-value: 1.20e-66
Operons with Upstream Motif: 78%

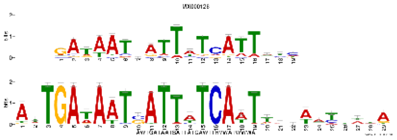

Motif similar to Fur -
E. coli (E = 2.47e-05)

24 - PhoP-1 – Phosphate Limitation 1

Biological Function:
High-affinity phosphate uptake, teichuronic acid production pseudogene.

Subset of known regulon:
PhoP + SigA

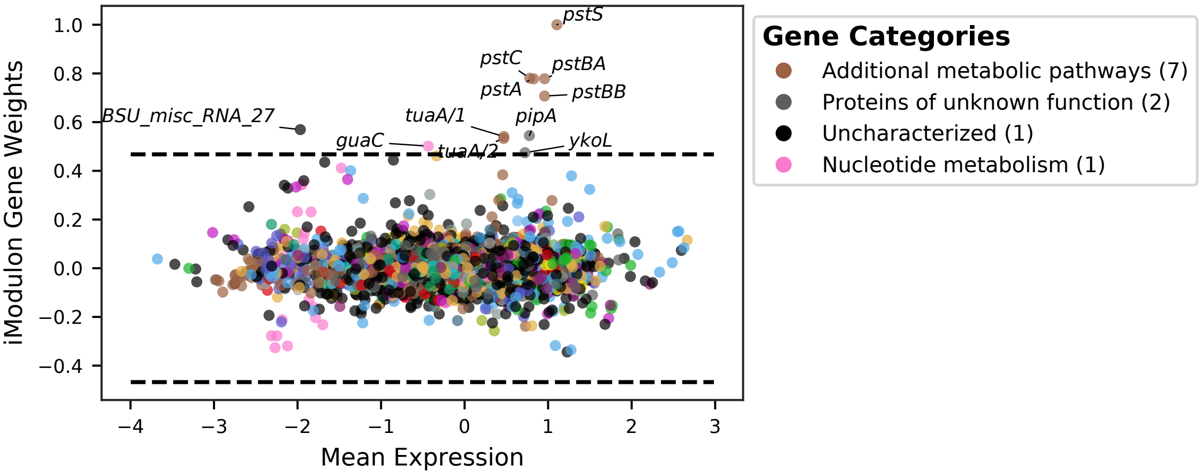

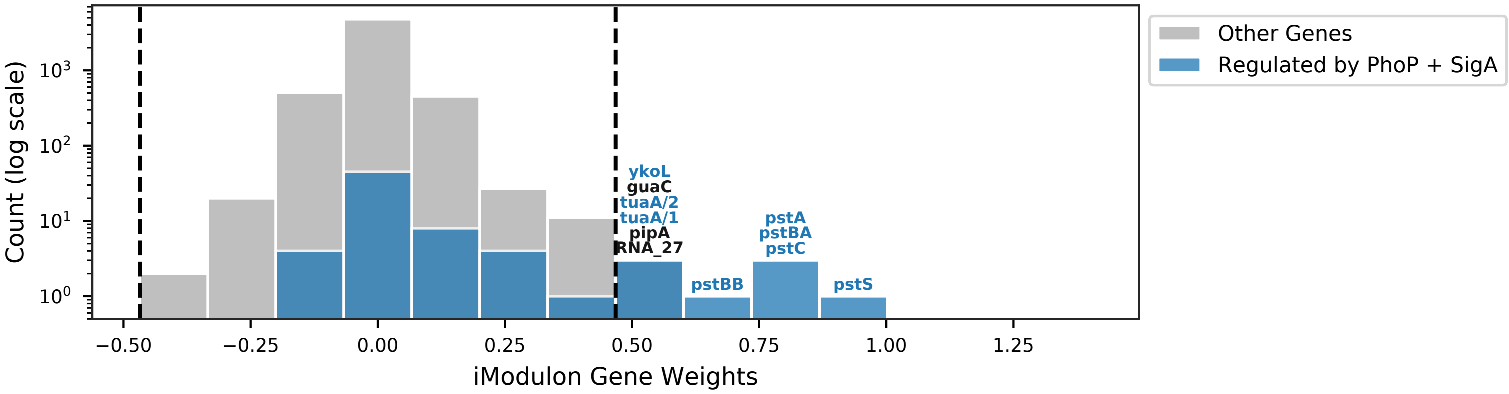

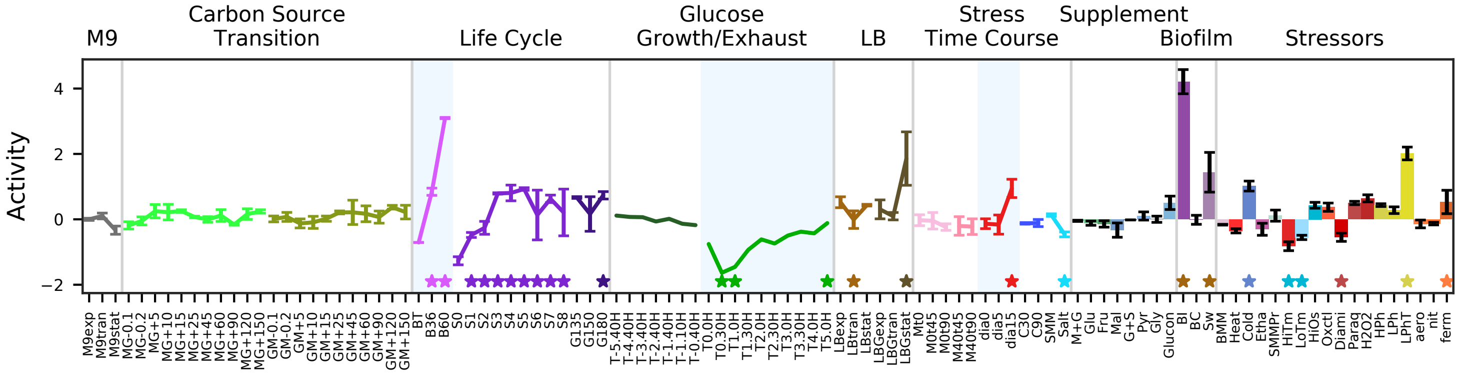

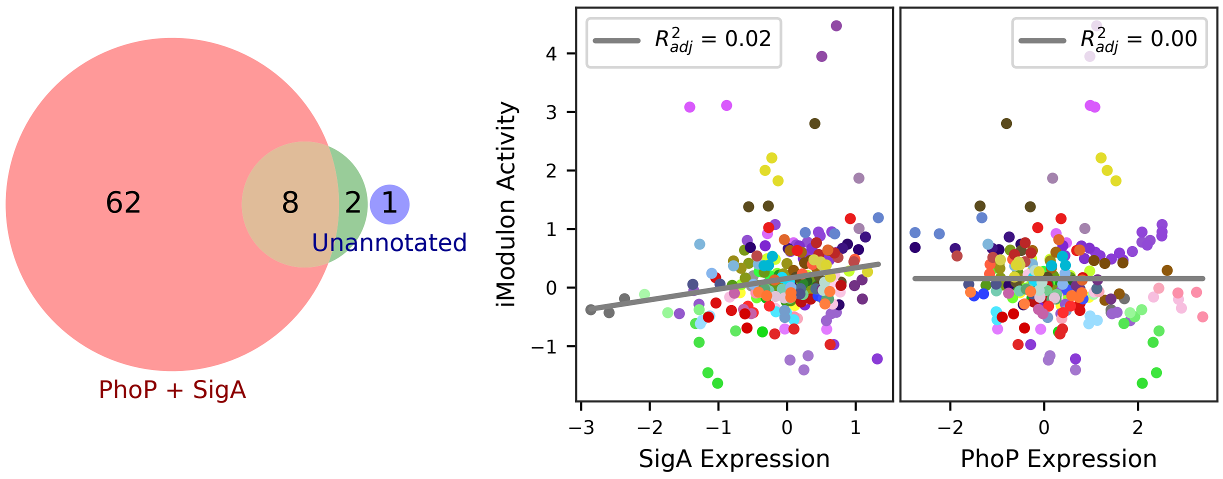

25 - PhoP-2 – Phosphate Limitation 2

Biological Function:
Degradation of wall teichoic acid to salvage phosphate, synthesis of teichuronic acid to replace wall, high-affinity phosphate uptake.

Subset of known regulon:
PhoP + SigA

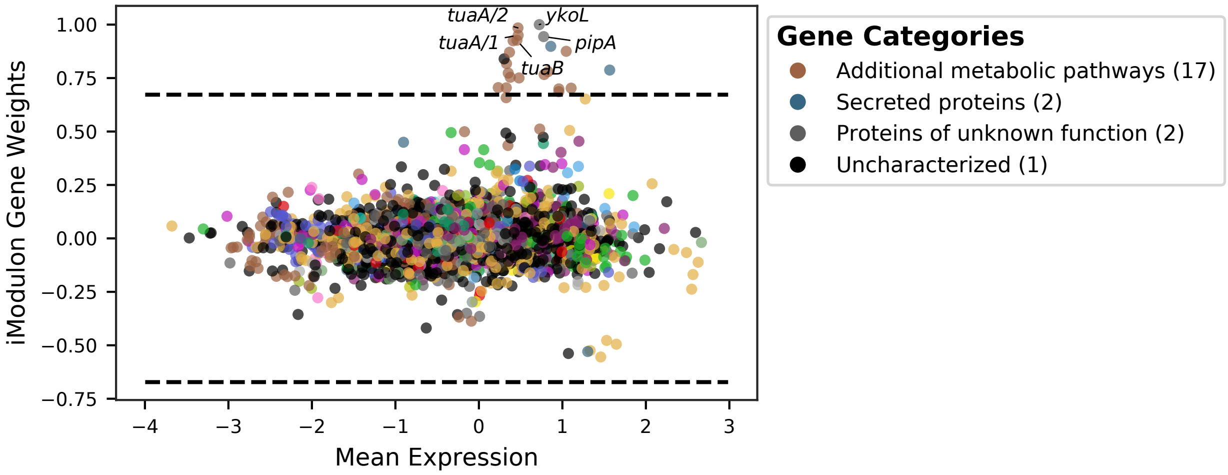

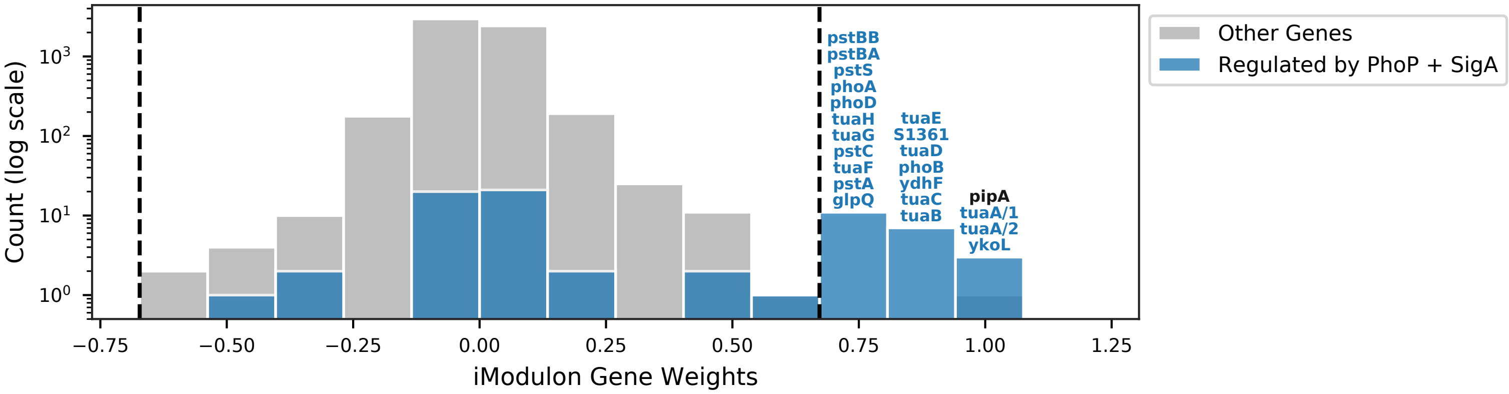

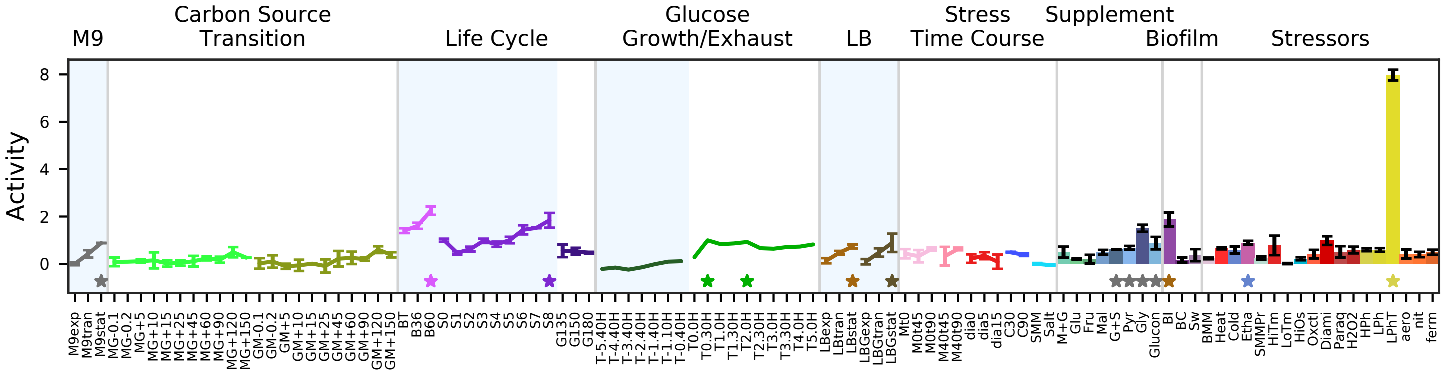

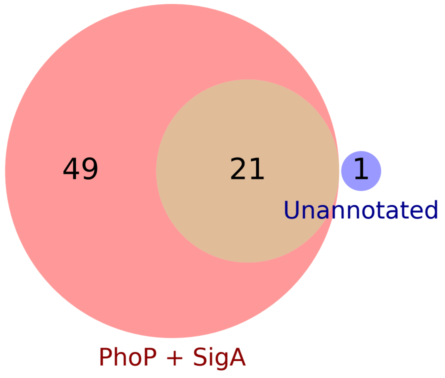

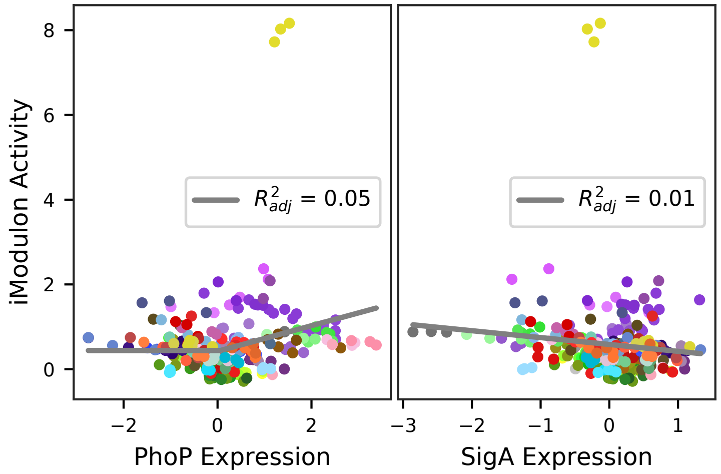

26 - Zur – Zinc Limitation

Biological Function:
Zinc uptake, zinc metallochaperones, alternatives to zinc-containing proteins

Well-defined regulon:
Zur

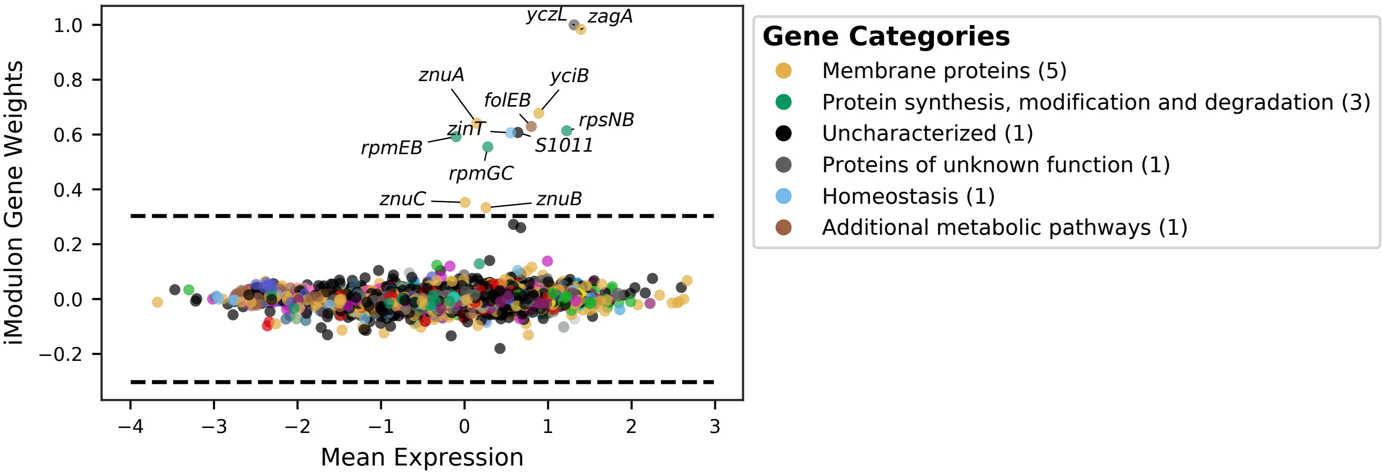

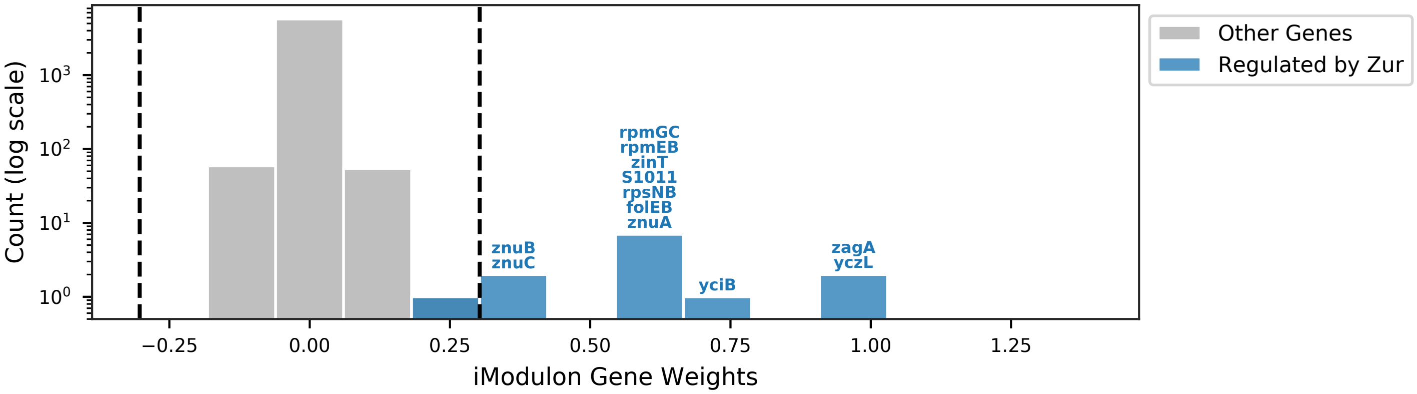

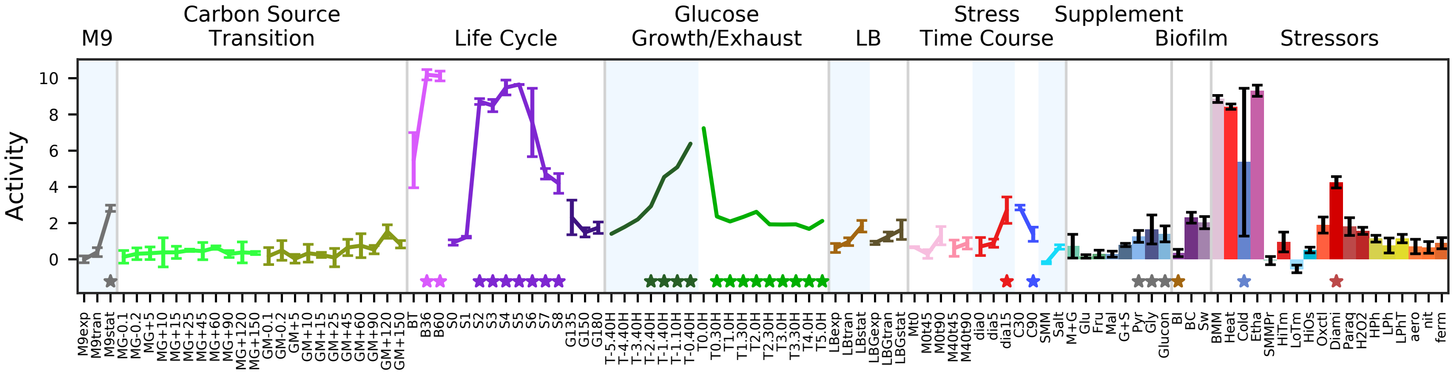

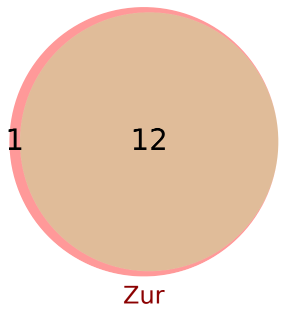

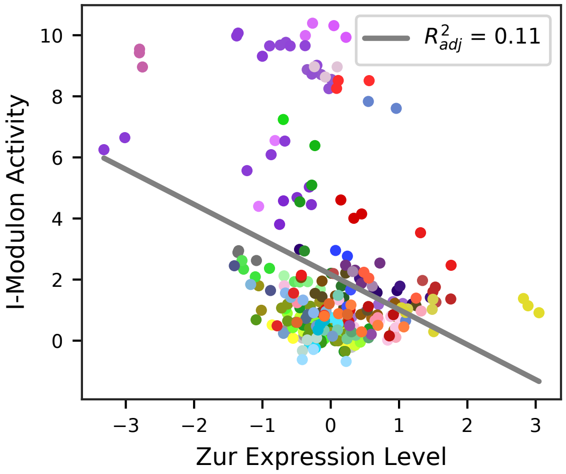

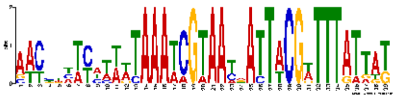

Motif E-value: 4.50e-19
Operons with Upstream Motif: 78%

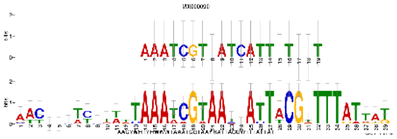

Motif similar to Zur (E = 1.09e-03)

27 - CzrA – Toxic Cation Export

Biological Function:
Resistance to Zn, Cu, Co, Ni, and Cd

Well-defined regulon:
CzrA

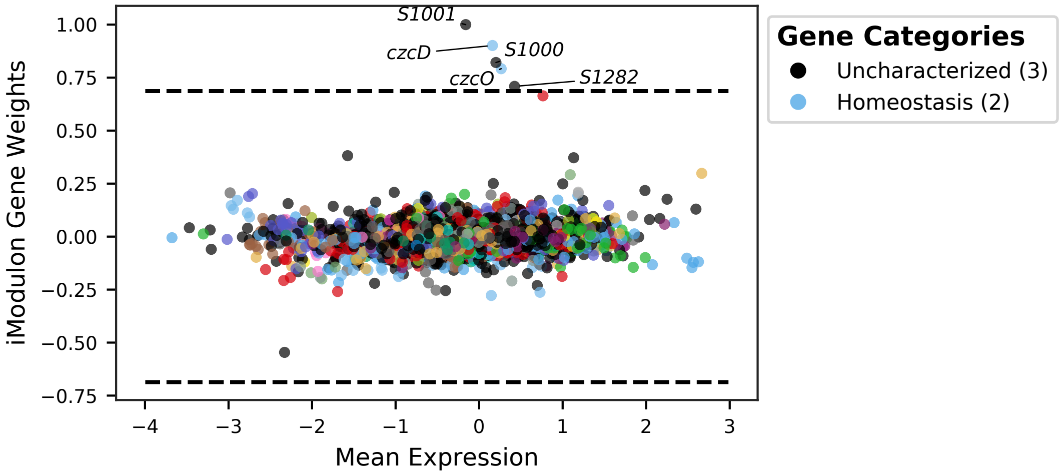

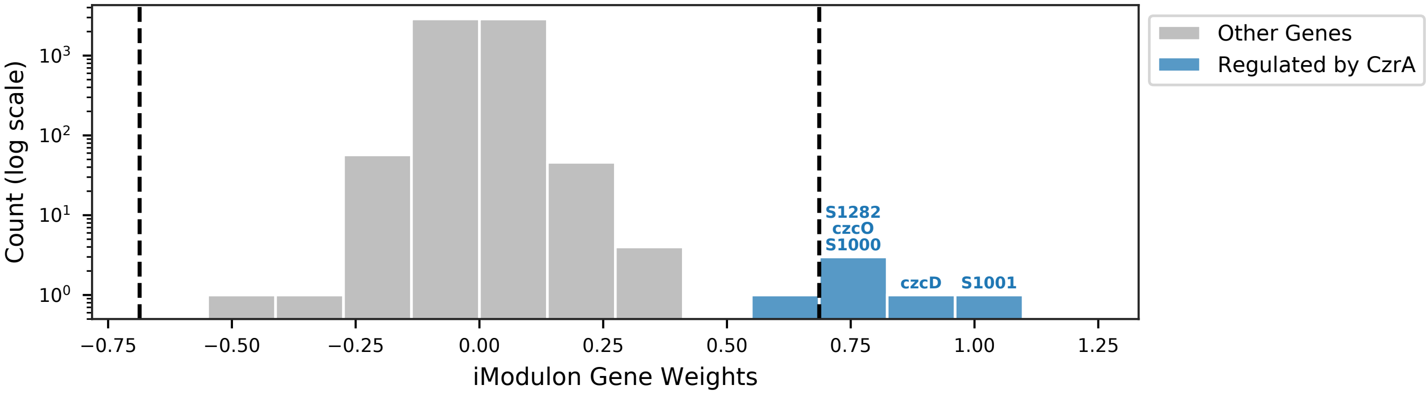

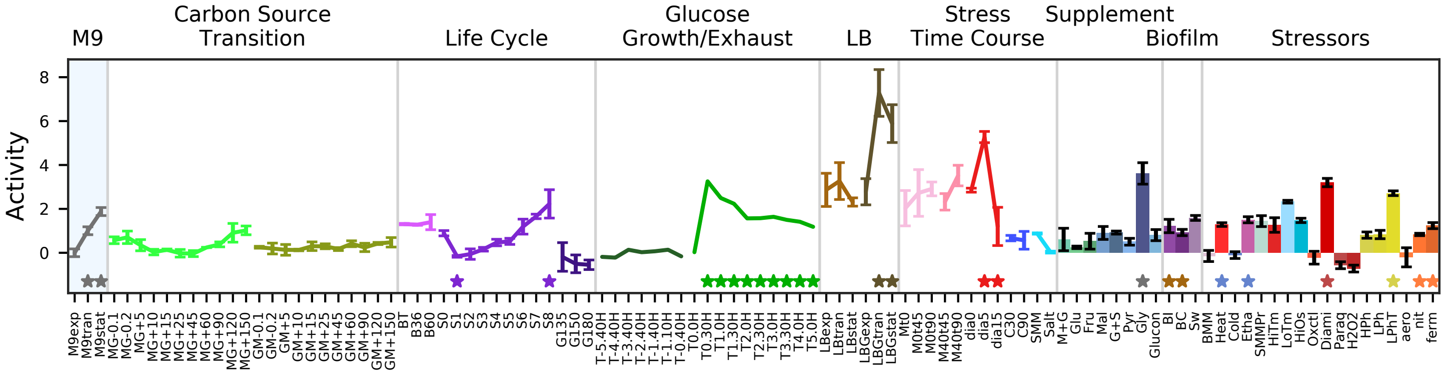

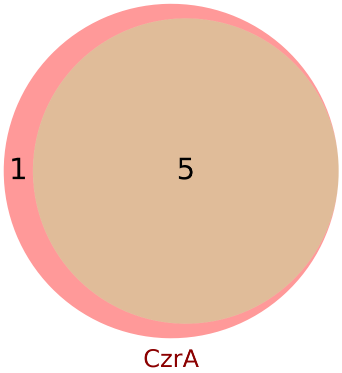

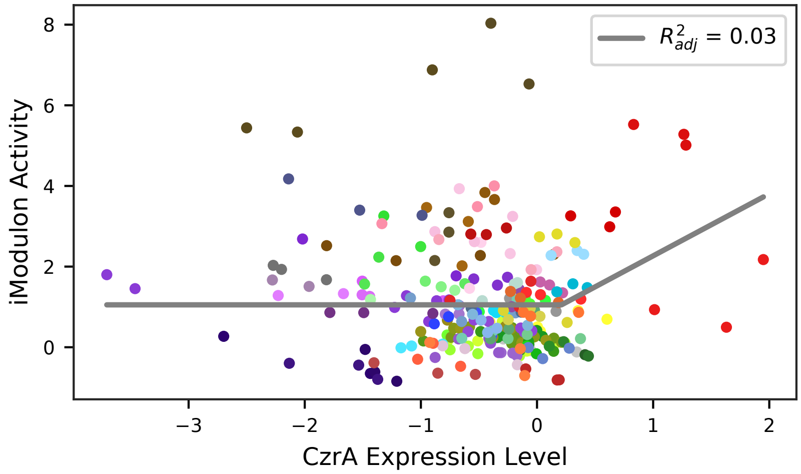

28 - CymR – Sulfur

Biological Function:
Sulfur metabolism in response to oxidative stress. Utilization and detoxification of S-(2-succino)cysteine, methionine-cysteine conversion, siroheme biosynthesis

Well-defined regulon:
CymR

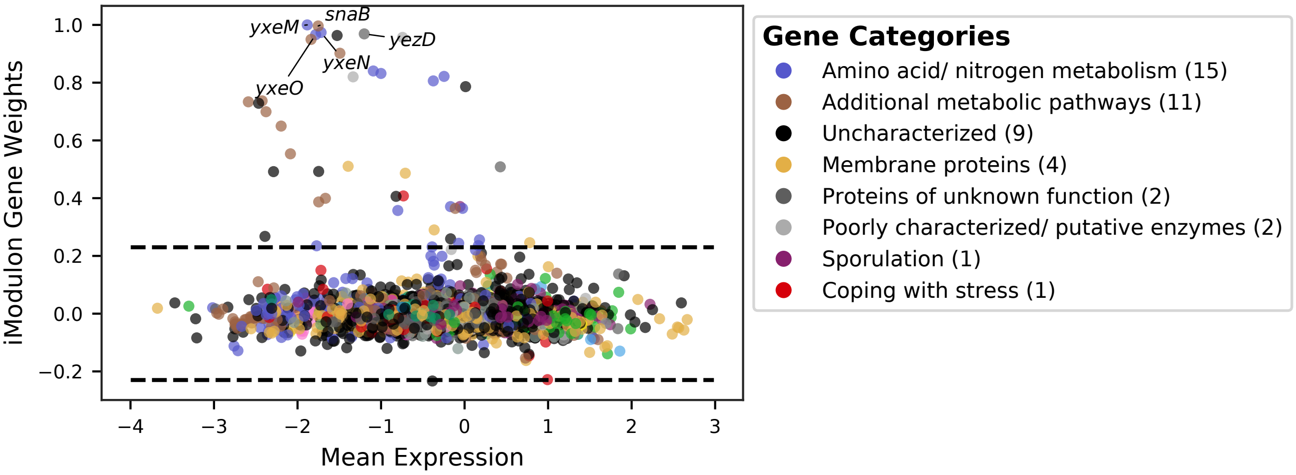

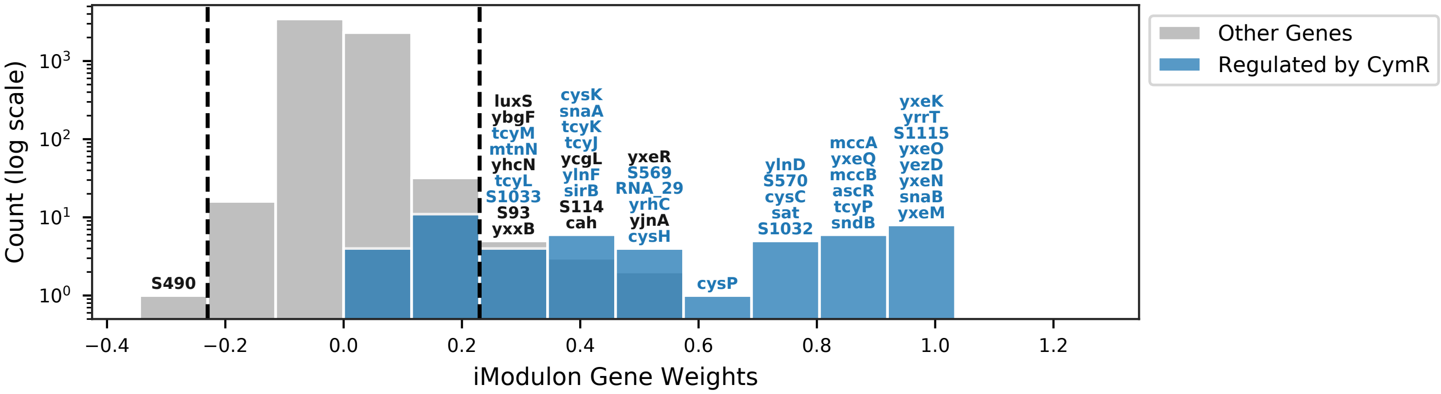

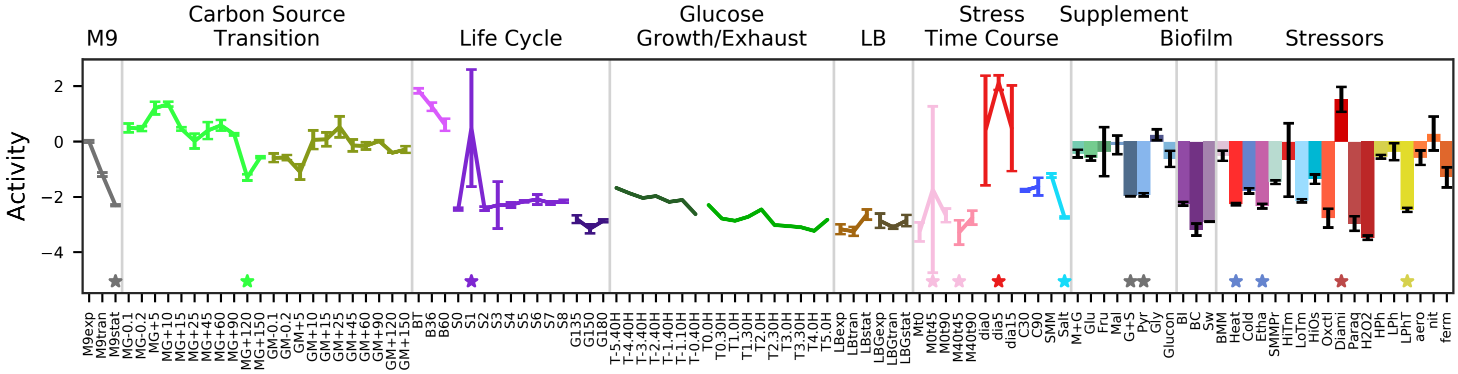

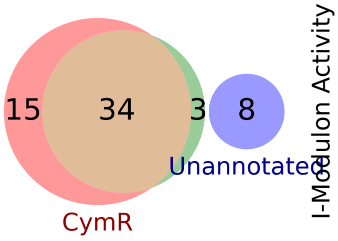

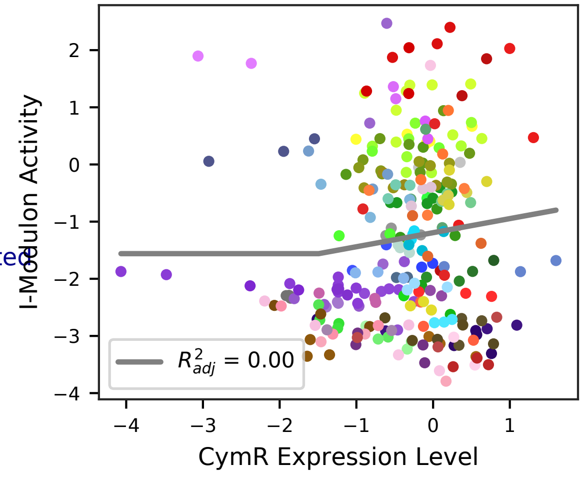

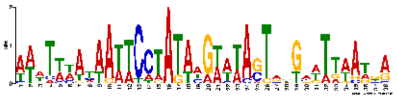

Motif E-value: 1.30e-06
Operons with Upstream Motif: 58%

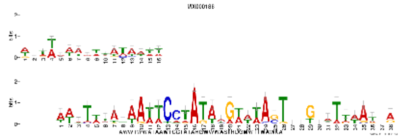

Motif similar to SarA - S. aureus (E = 2.84e-04)

29 - Yrk – Putative Sulfur Carriers

Biological Function:
Putative sulfur carriers. High activity under diamide stress

No known regulator

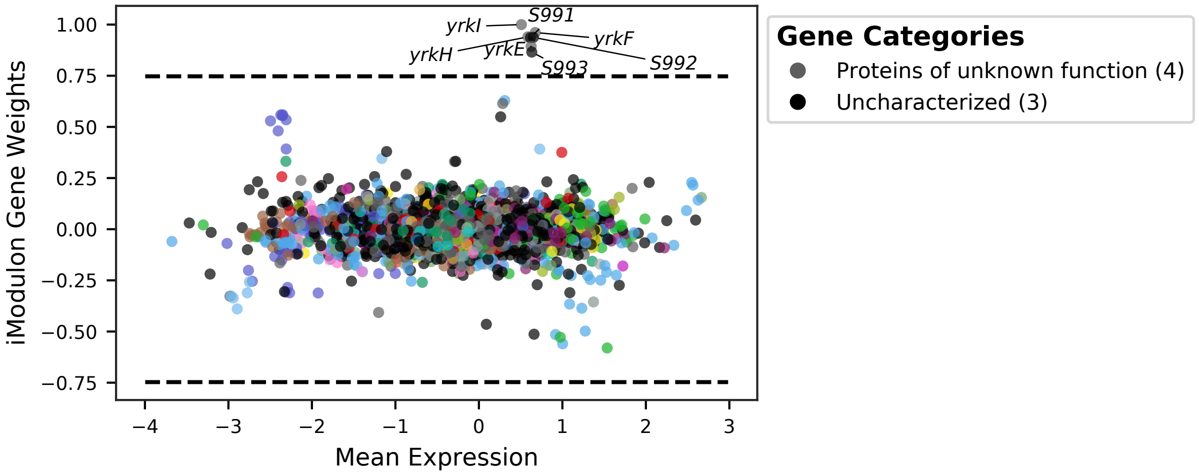

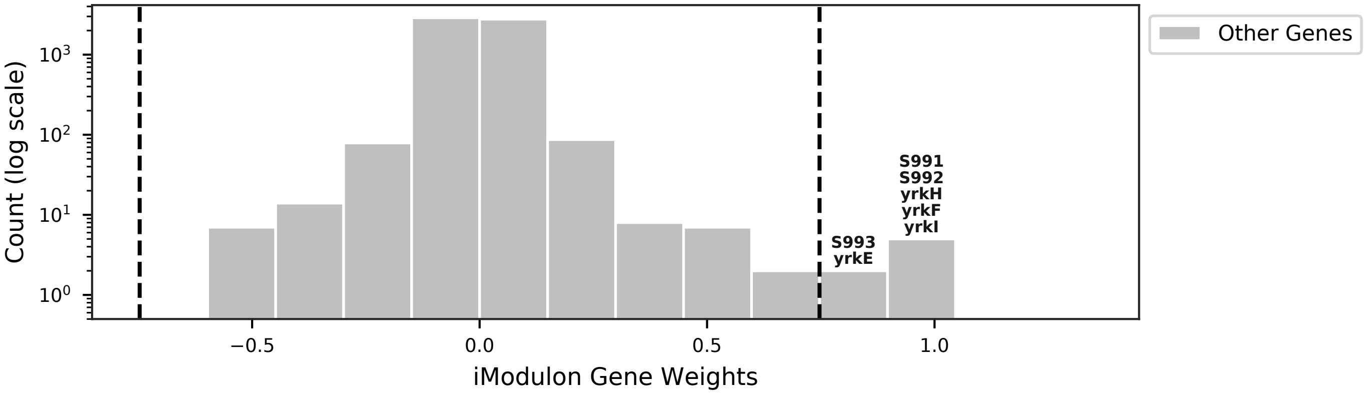

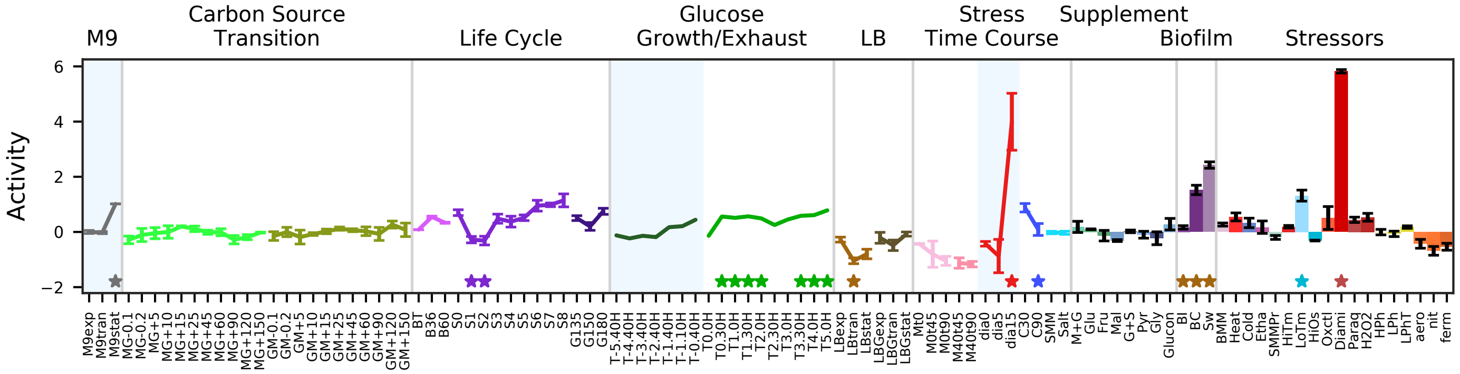

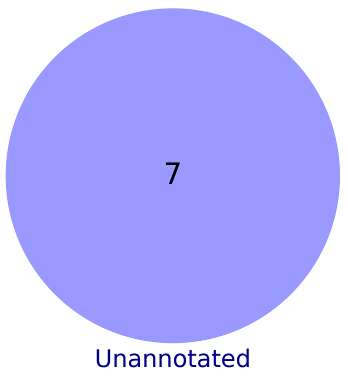

30 - ResD – Oxygen Limitation

Biological Function:
Anaerobic nitrate respiration, bacteriocin production, heme, cytochrome, and ATP synthesis, copper homeostasis

Enriched for known regulon:
ResD

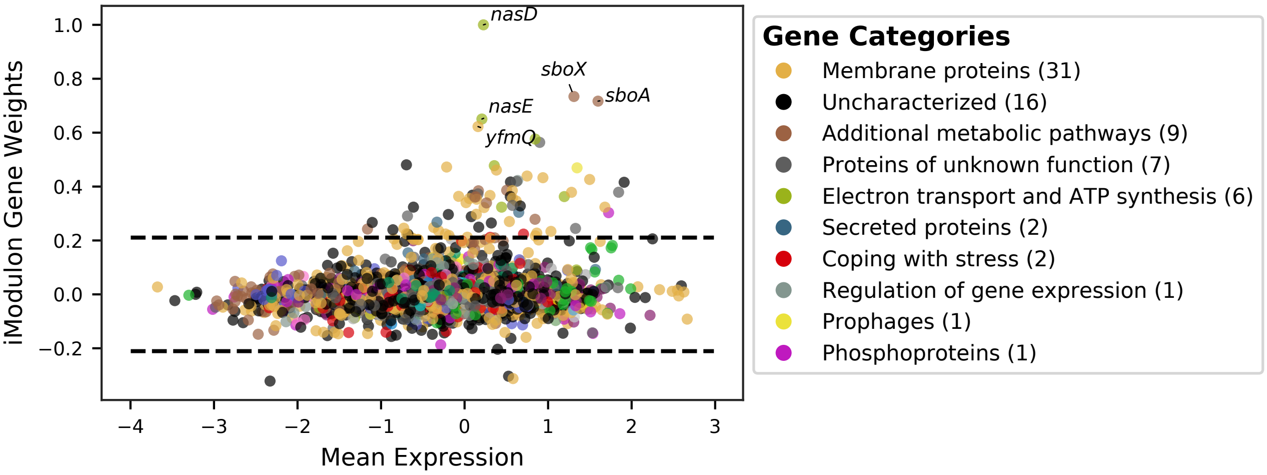

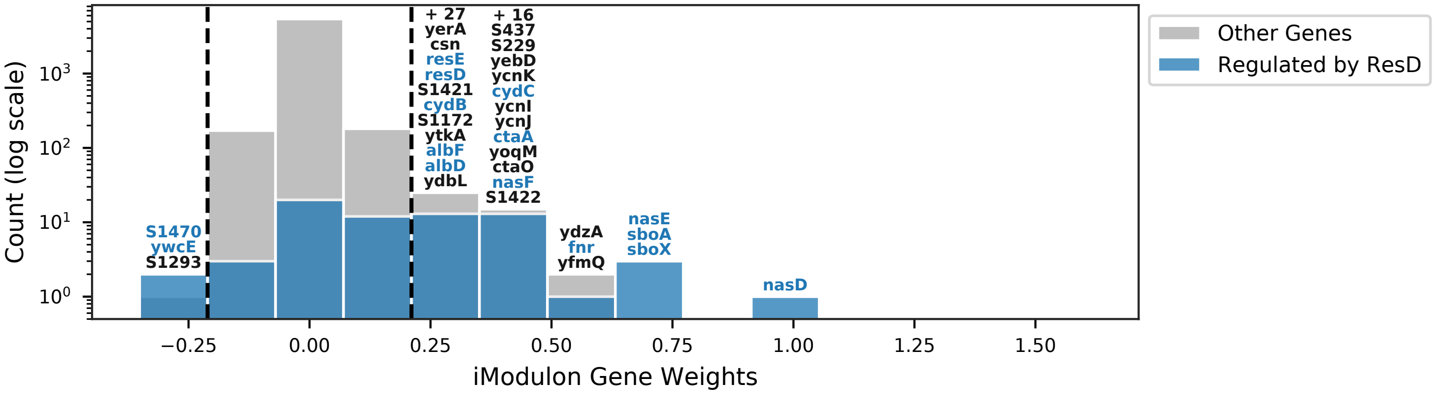

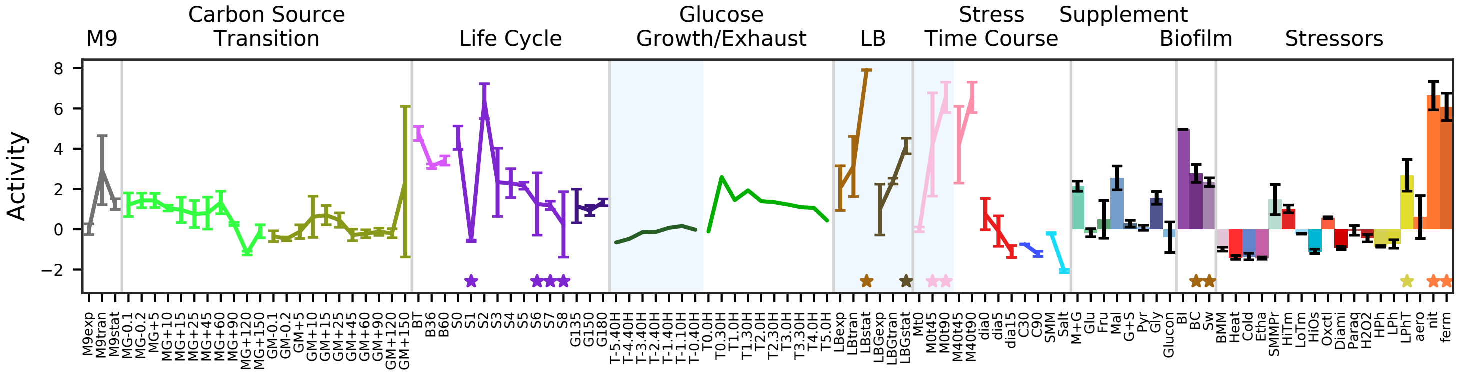

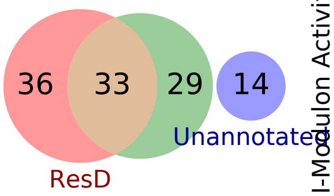

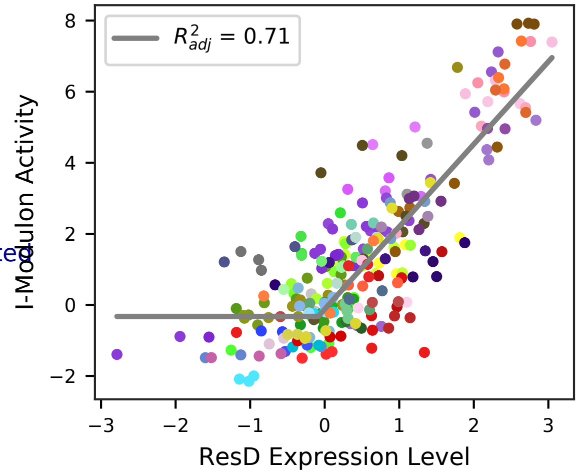

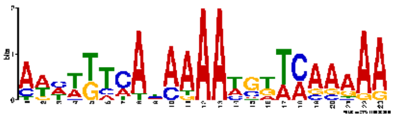

Motif E-value: 9.80e-04
Operons with Upstream Motif: 82%

31 - Fnr - Nitrate Respiration

Biological Function:
Nitrate respiration and nitrite extrusion

Well-defined regulon:
Fnr + SigA

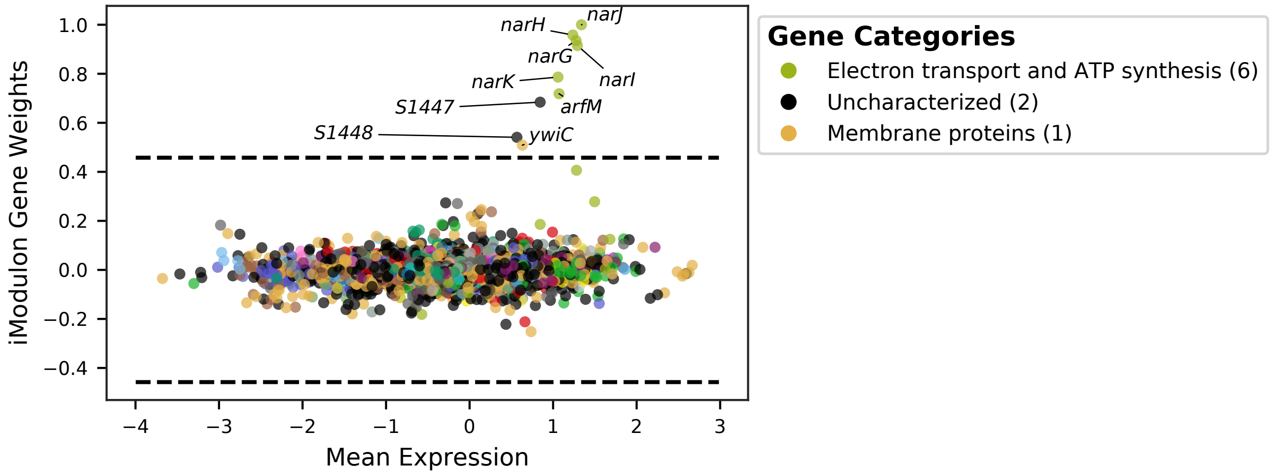

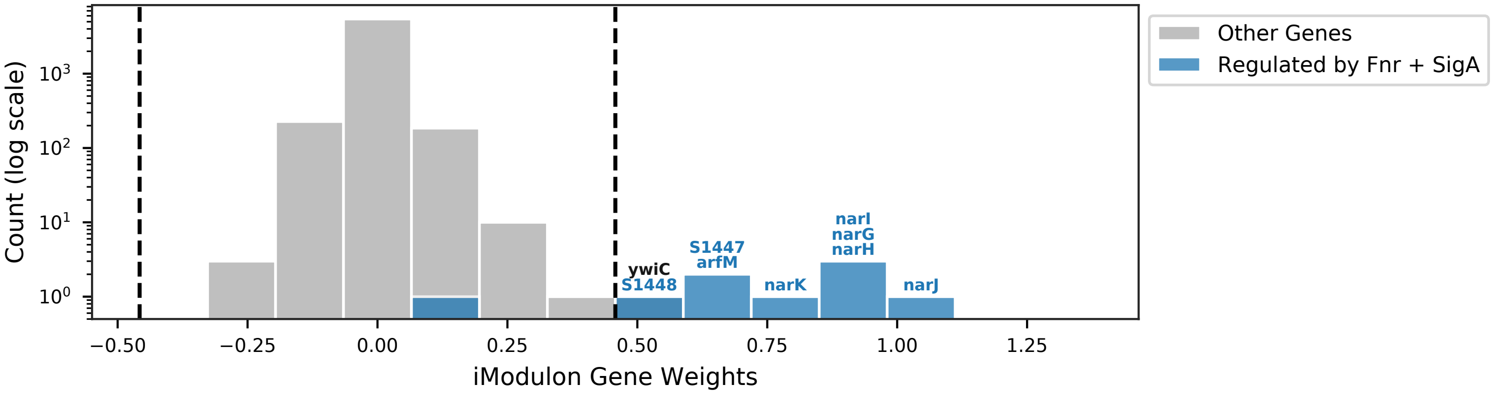

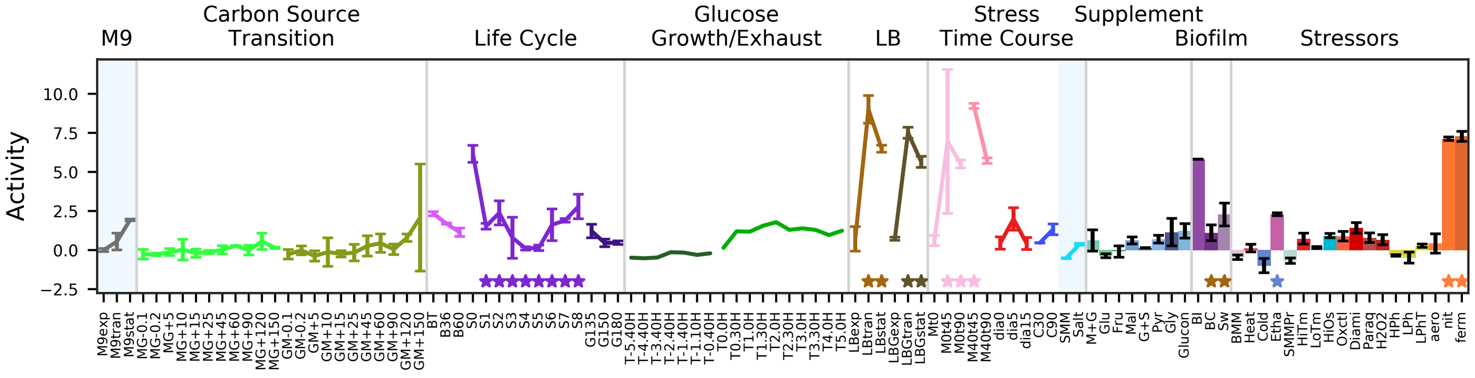

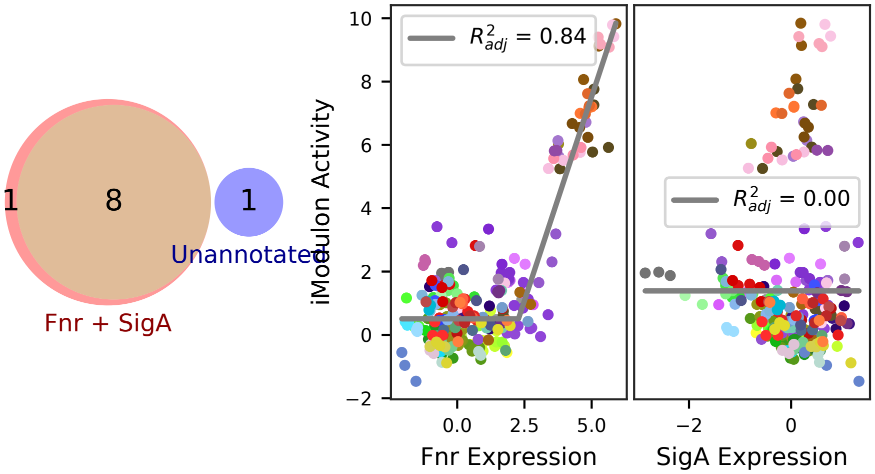

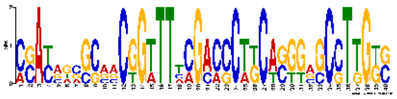

Motif E-value: 1.60e-05
Operons with Upstream Motif: 100%

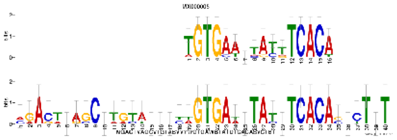

Motif similar to Fnr (E = 2.37e-08)

32 - Rex – Overflow Metabolism

Biological Function:
Overflow metabolism (LDH) and cytochrome BD oxidation for respiration in anaerobic conditions

Subset of known regulon:
Rex

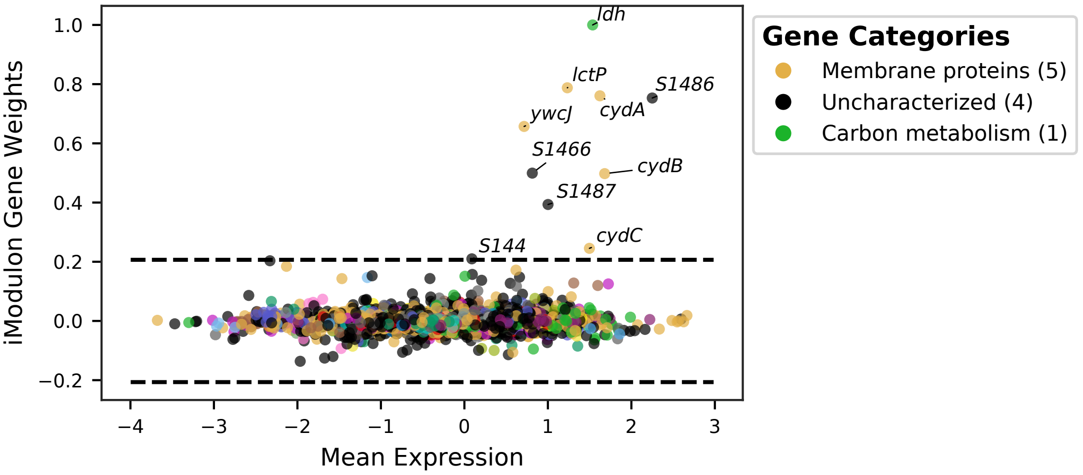

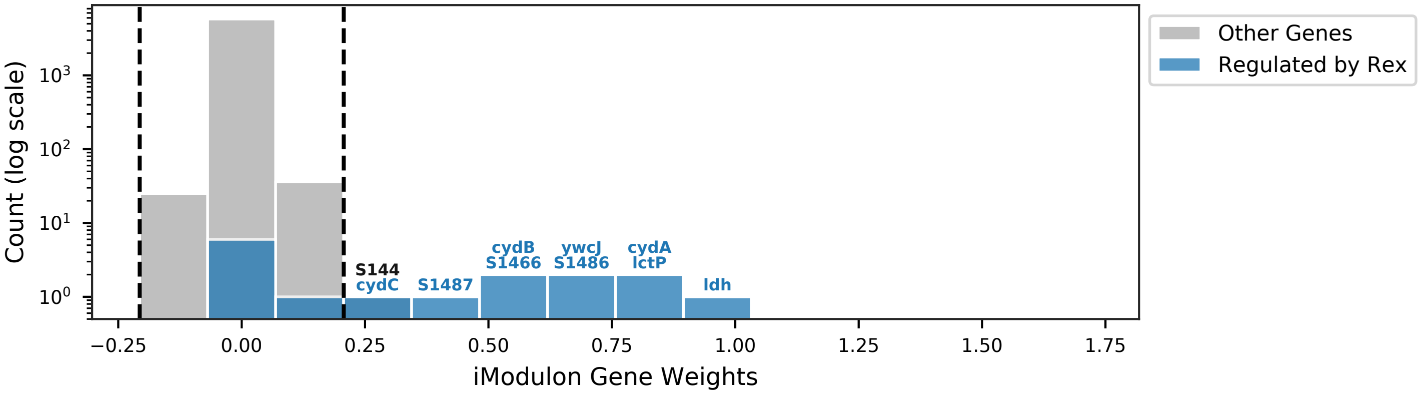

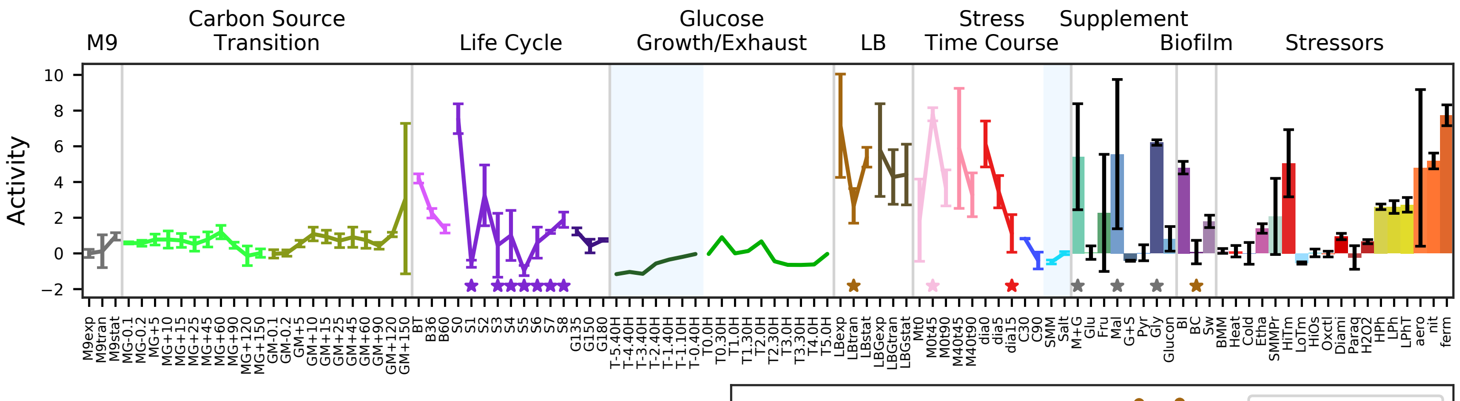

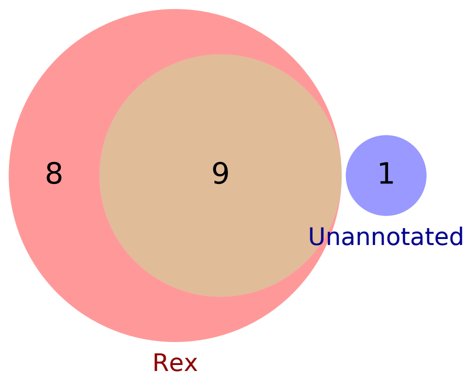

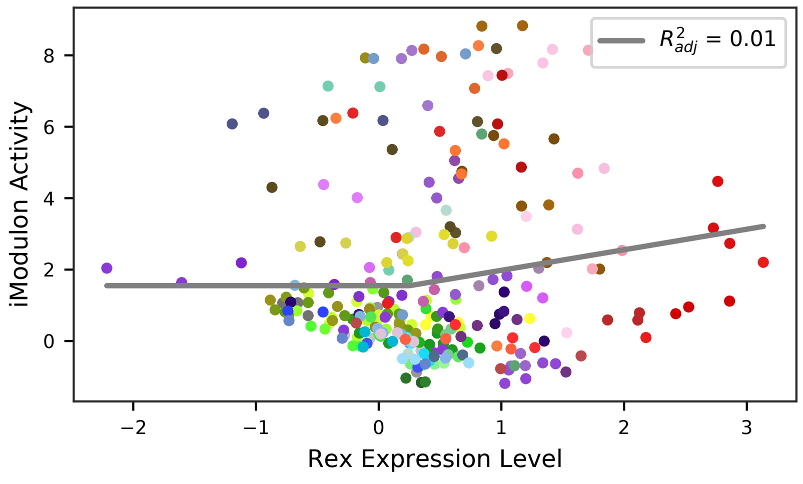

33 - LexA – DNA Repair

Biological Function:
SOS response for DNA protection and repair

Subset of known regulon:
LexA

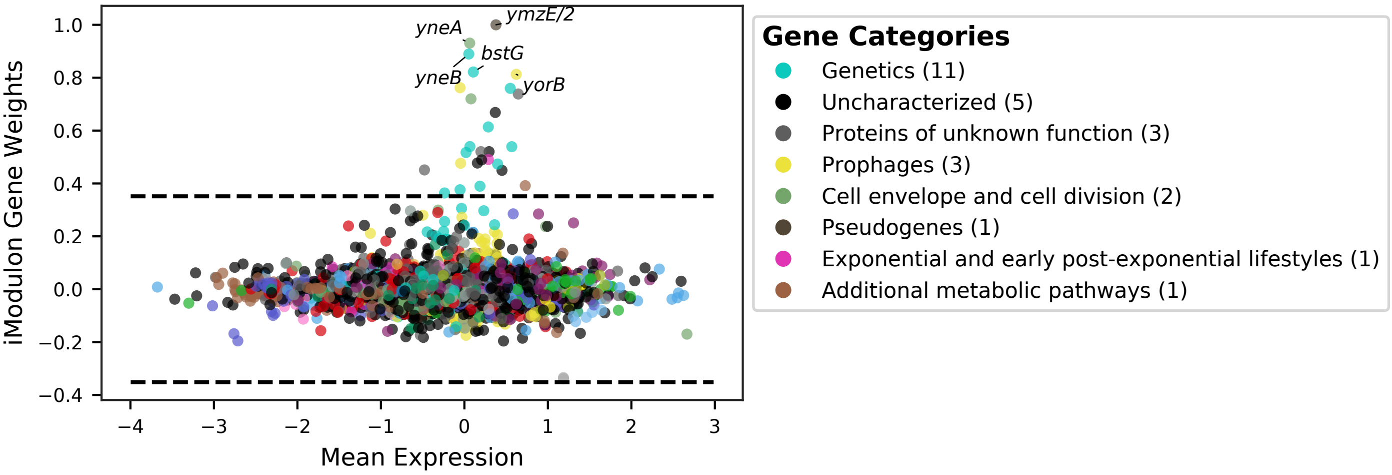

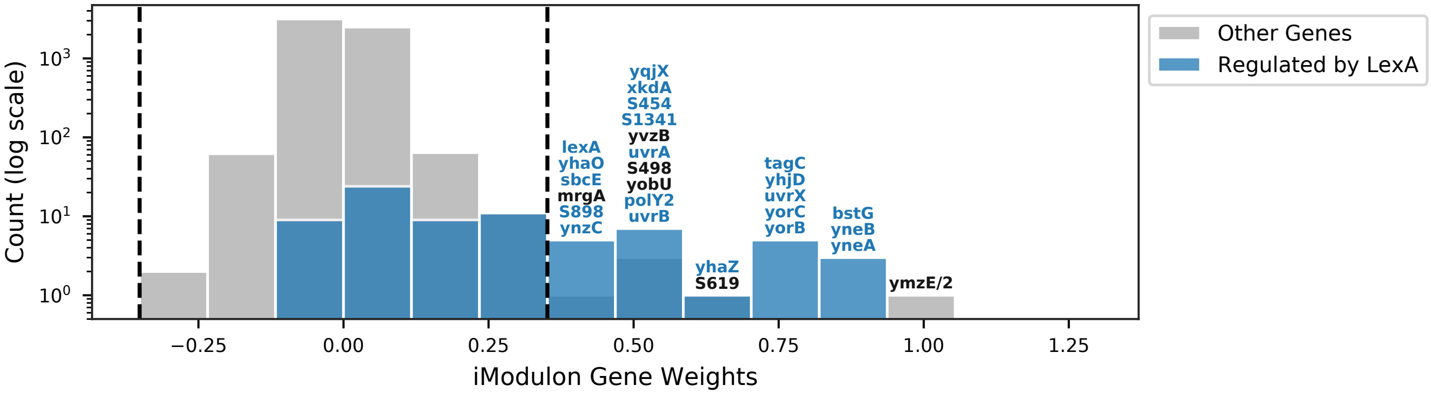

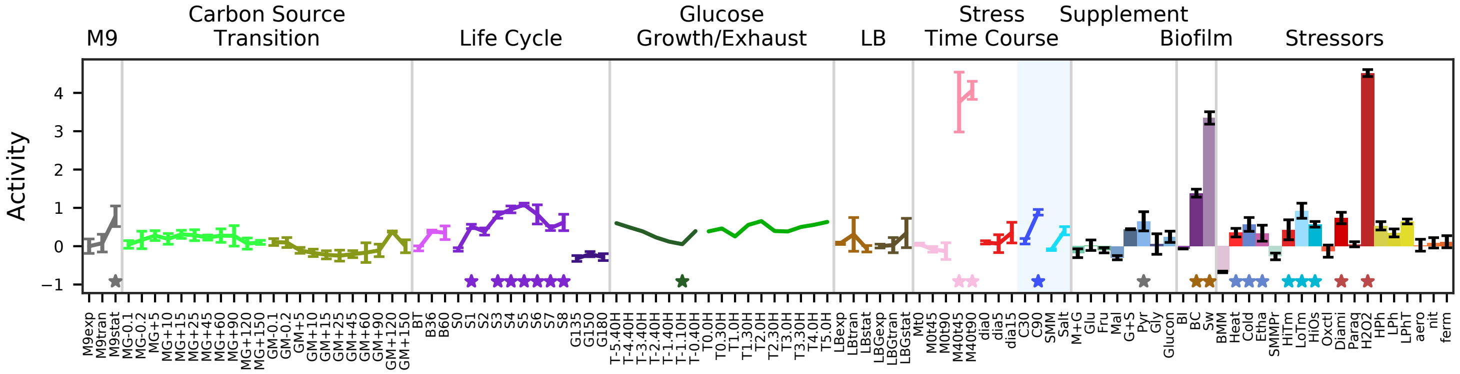

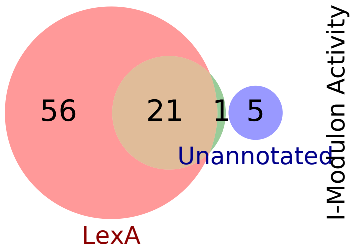

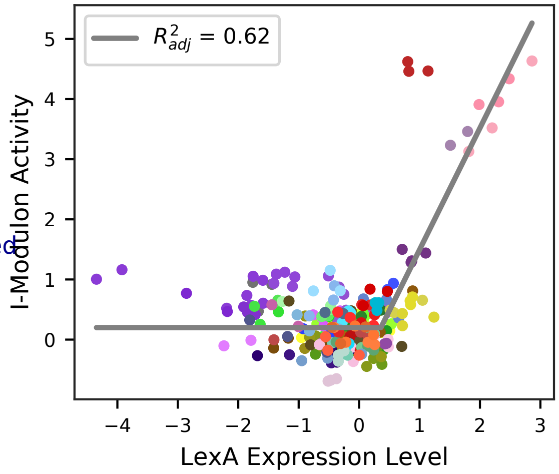

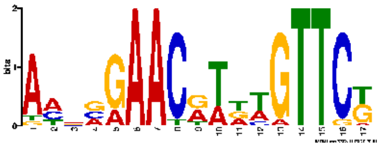

Motif E-value: 2.00e-09
Operons with Upstream Motif: 87%

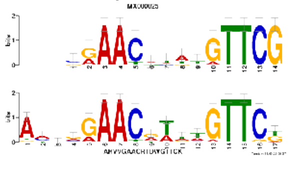

Motif similar to LexA (E = 3.21e-08)

34 - SigW – Cell Wall Stress

Biological Function:
Adaptation to membrane active agents such as cefuroxime, antimicrobials from B. amyloliquefaciens, sdpC, and nisin. Control of membrane fluidity. Contains many unknown proteins.

Subset of known regulon:
SigW

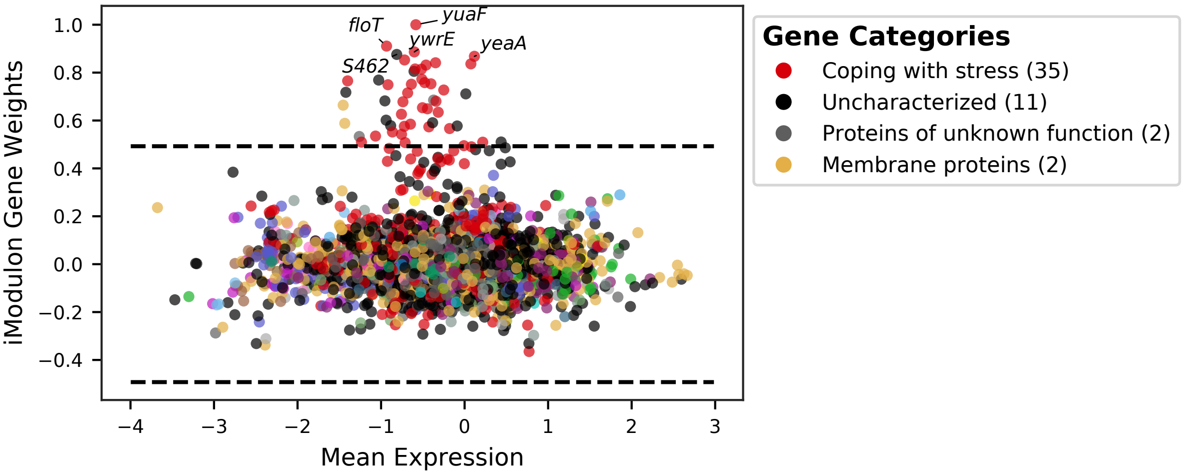

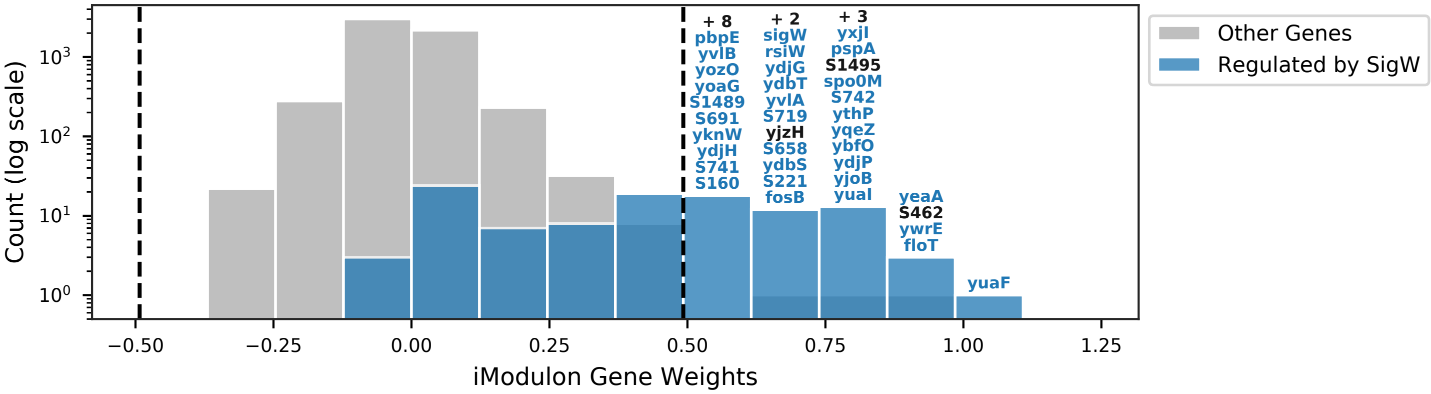

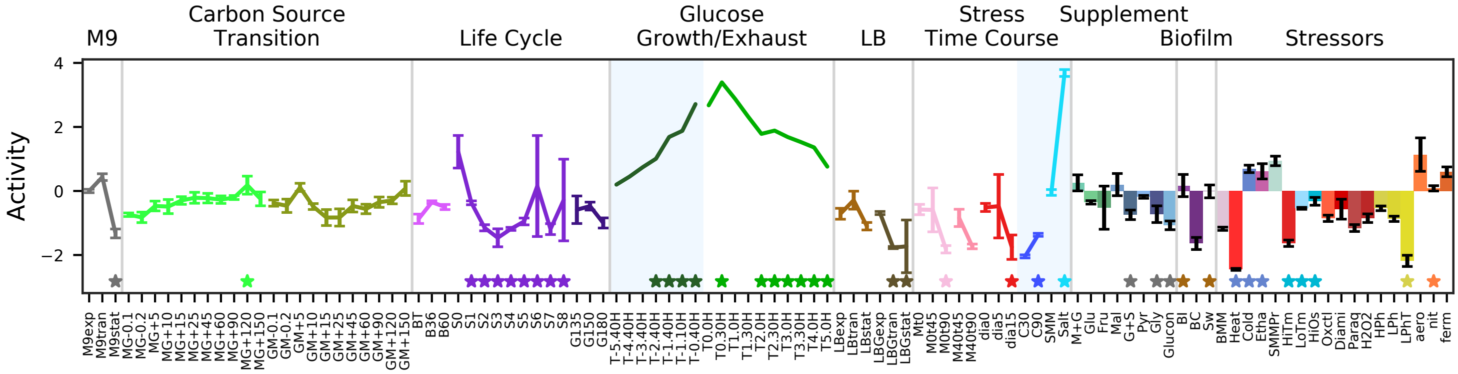

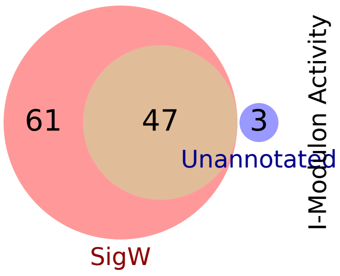

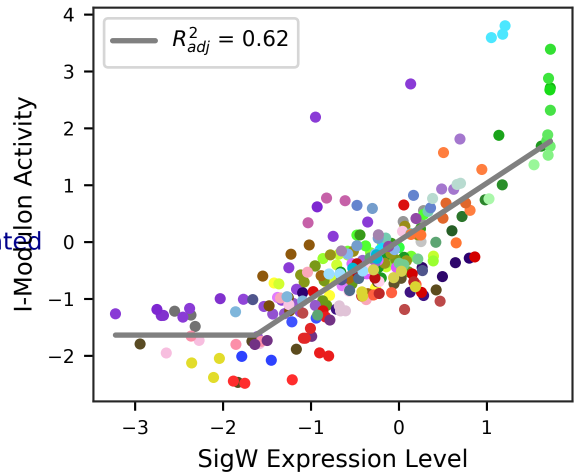

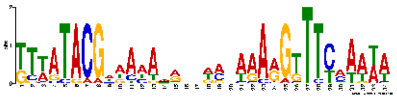

Motif E-value: 6.40e-05
Operons with Upstream Motif: 55%

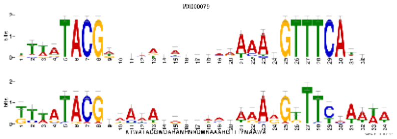

Motif similar to SigW (E = 1.51e-15)

35 - SigM – Membrane Stress

Biological Function:
General stress proteins, alarmones, lipid carriers and flippases, DNA repair, inhibition of septation. Contains many UTRs and some unknown proteins.

Subset of known regulon:
SigM

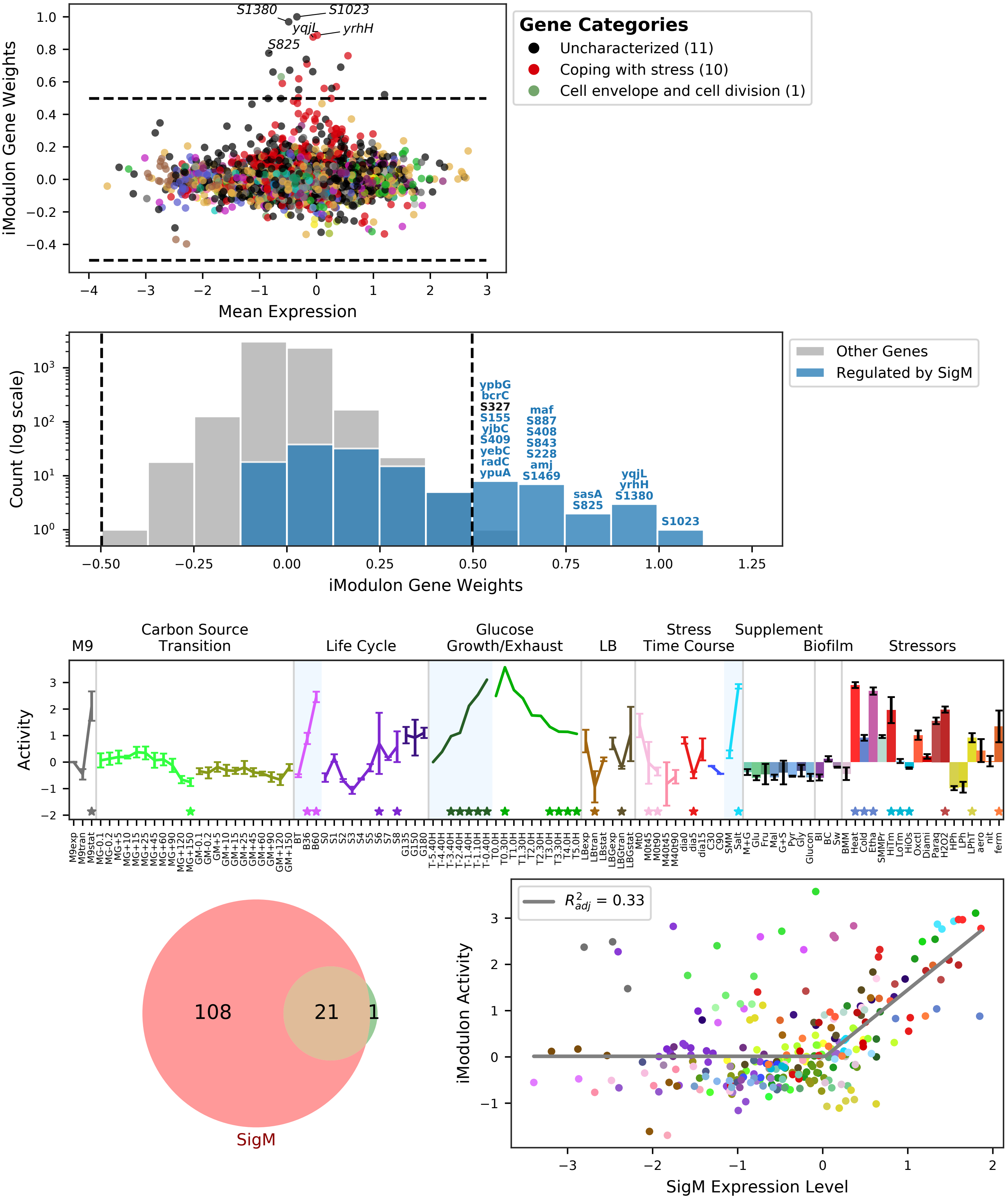

36 - Ybc – Uncharacterized Operon

Biological Function:
Contains one subunit of NADH dehydrogenase with several unknown genes. Categorized as prophage.
Responds to heat shock.

No known regulator

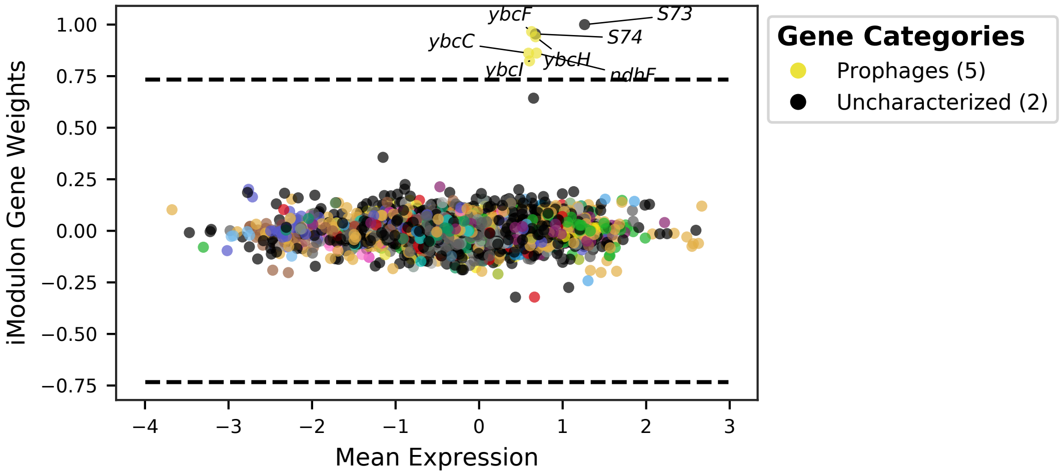

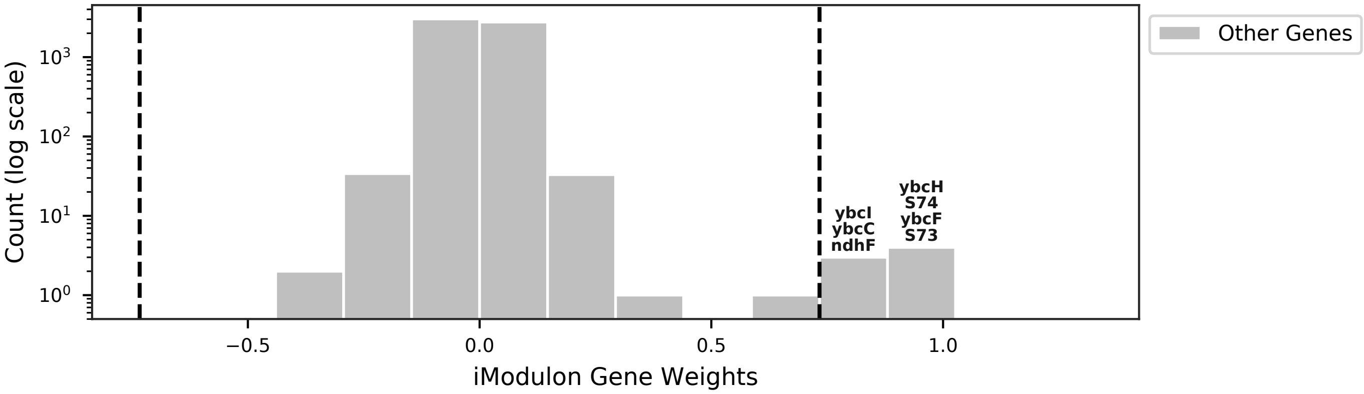

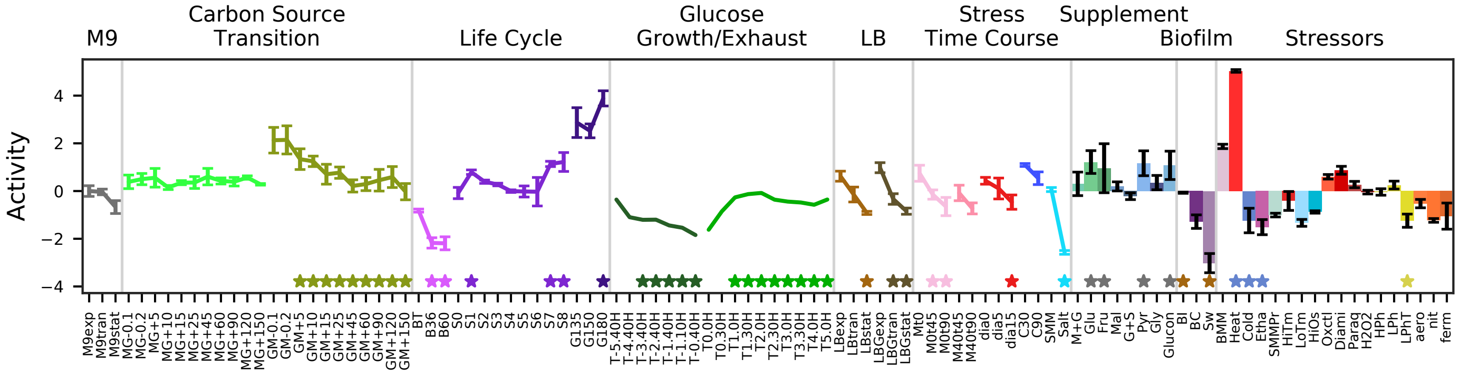

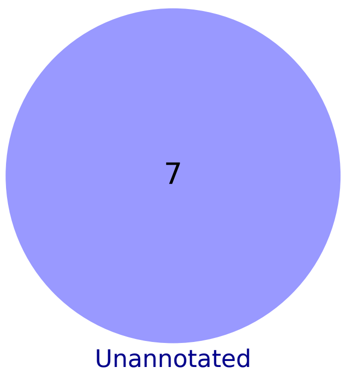

37 - SigB-1 – General Stress 1

Biological Function:
Survival of general stressors including ethanol, salt, paraquat, heat, and freeze-thaw

Subset of known regulon:
SigB

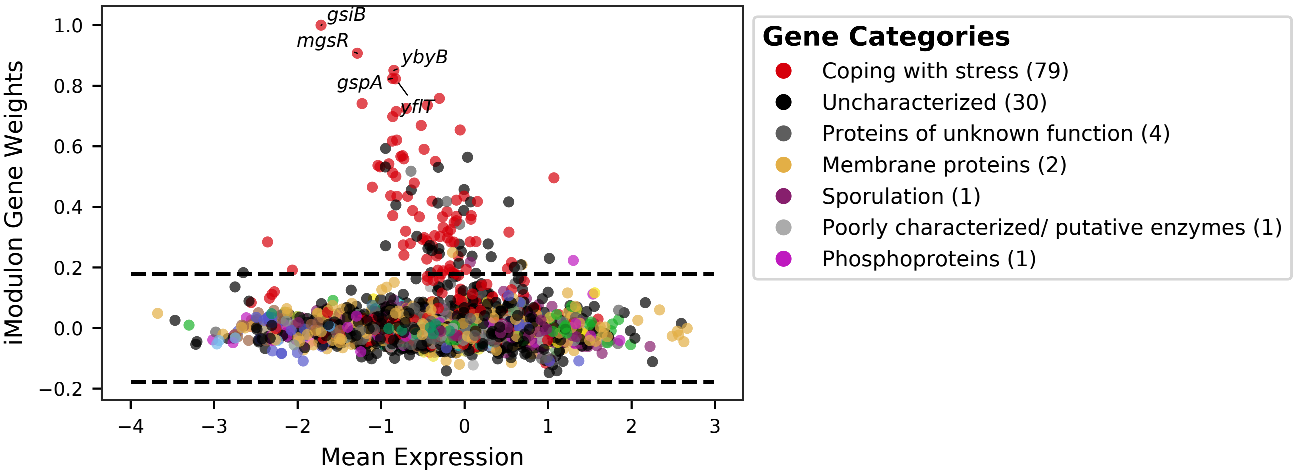

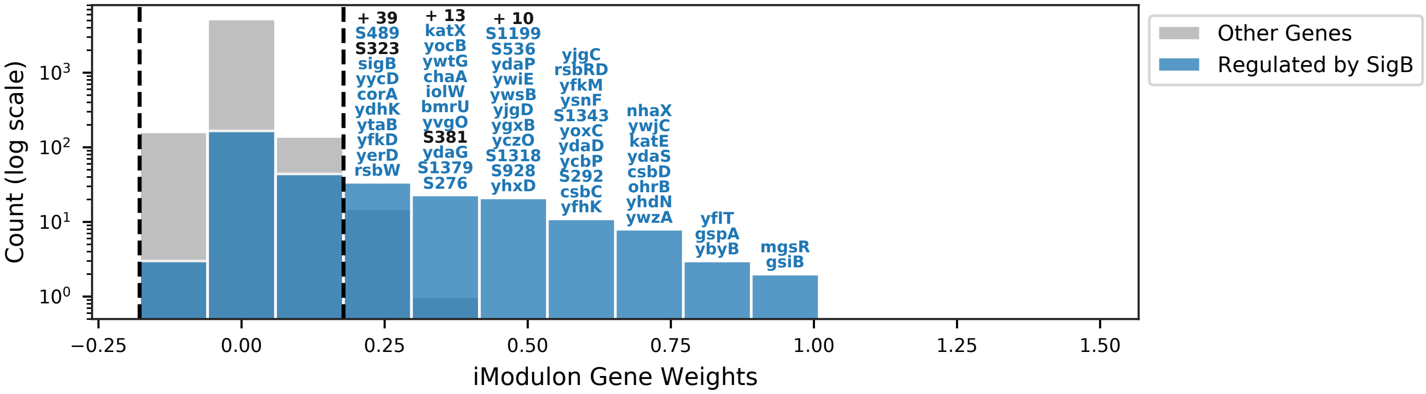

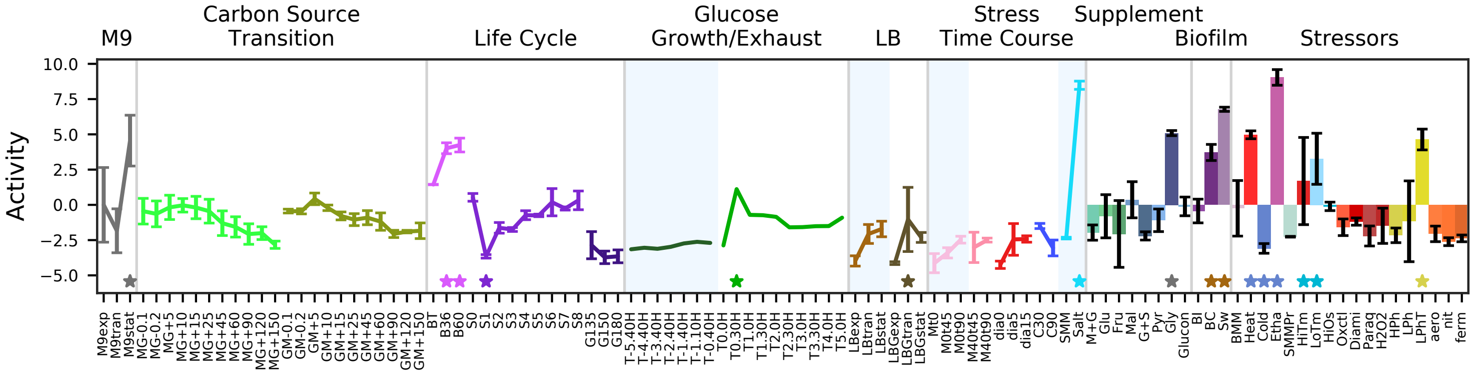

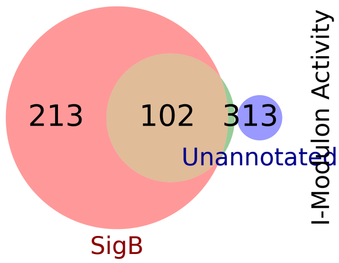

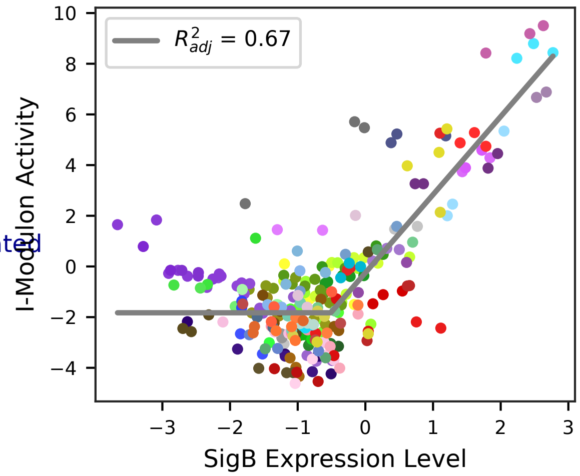

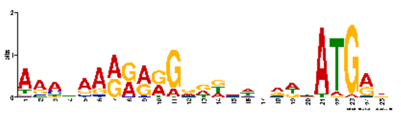

Motif E-value: 8.00e-21
Operons with Upstream Motif: 86%

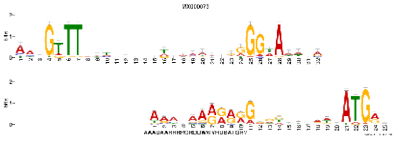

Motif similar to SigB (32-mer) (E = 8.65e-05)

38 - SigB-2 – General Stress 2

Biological Function:
Likely accounting for microarray saturation in the ethanol condition of the SigB-1 iModulon by negatively weighting highly expressed ethanol genes

Enriched for known regulon:
SigB

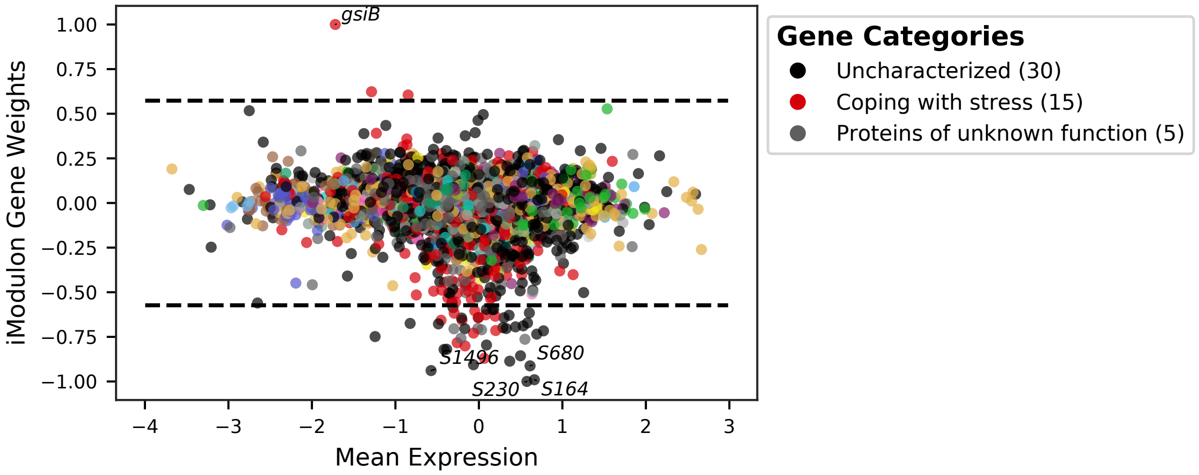

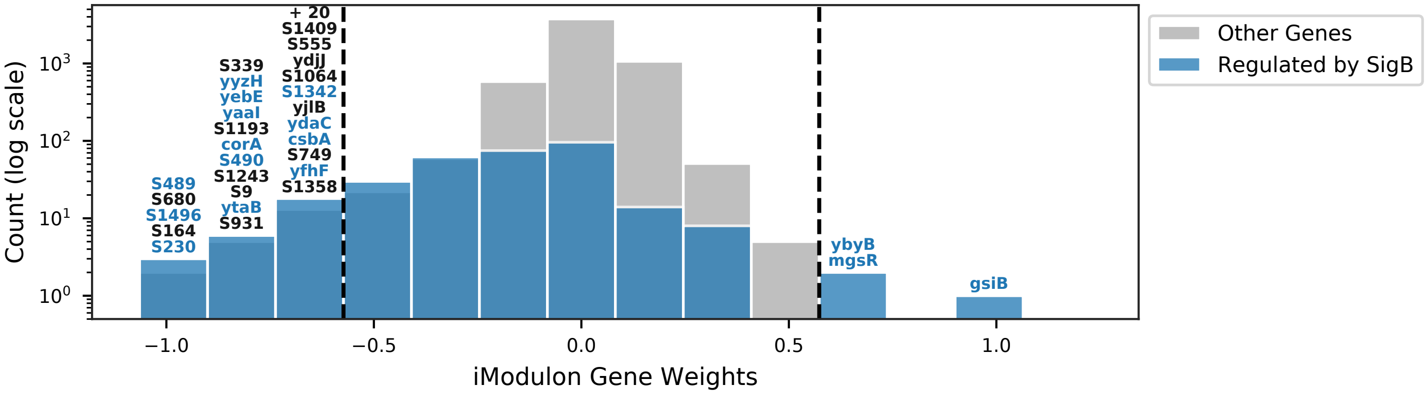

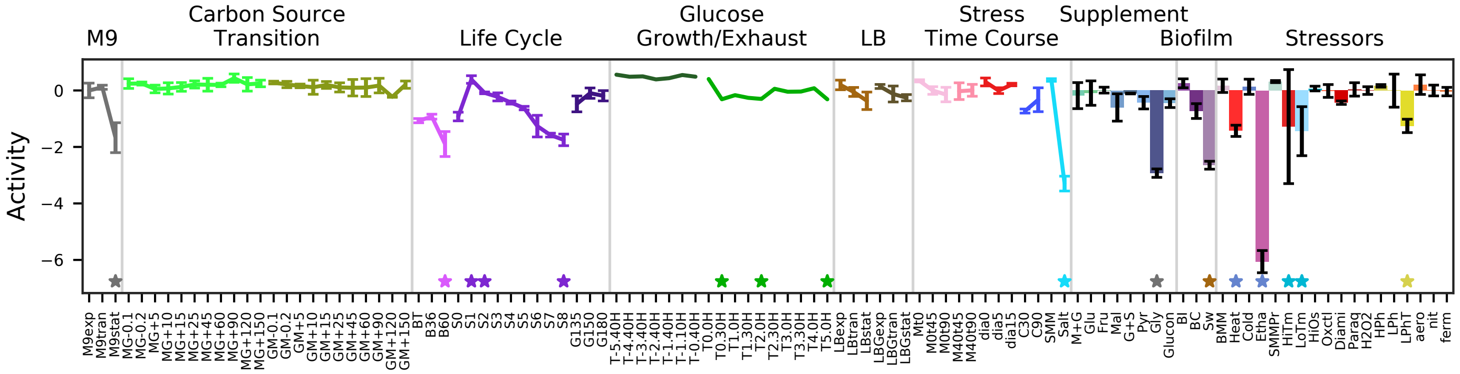

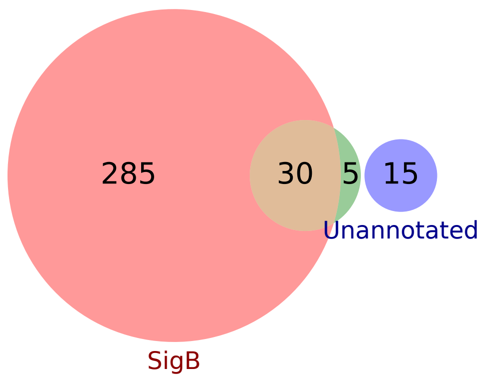

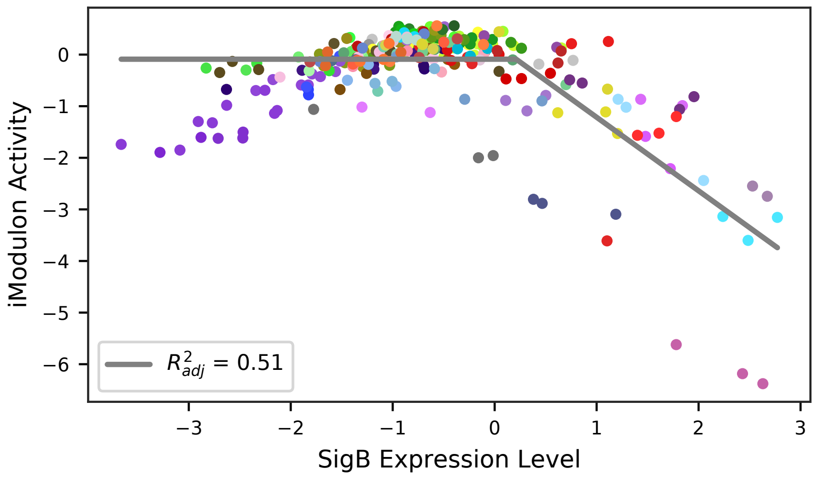

39 - StrRes – Translation

Biological Function:
Production of ribosomes, translation initiation factors, ADP formation, protein processing and secretory machinery, DNA replication, RNA polymerase

Subset of known regulon:
[stringent response] / [RplJ]

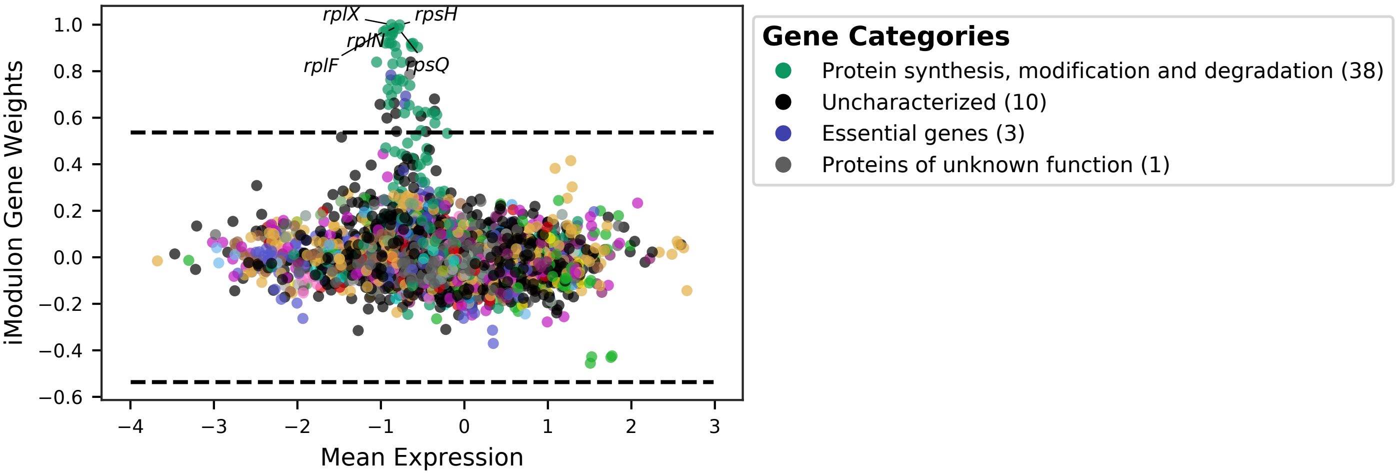

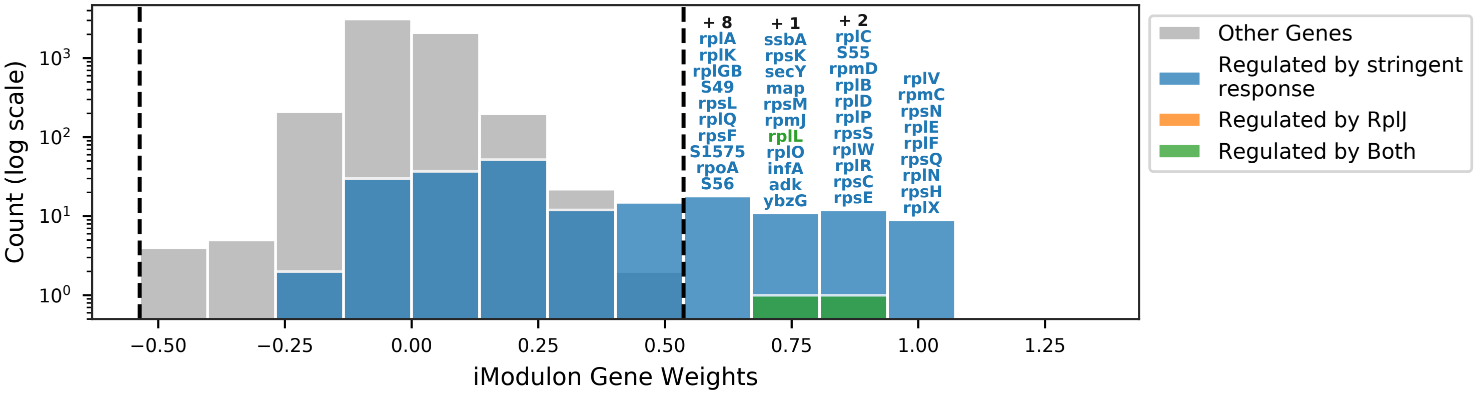

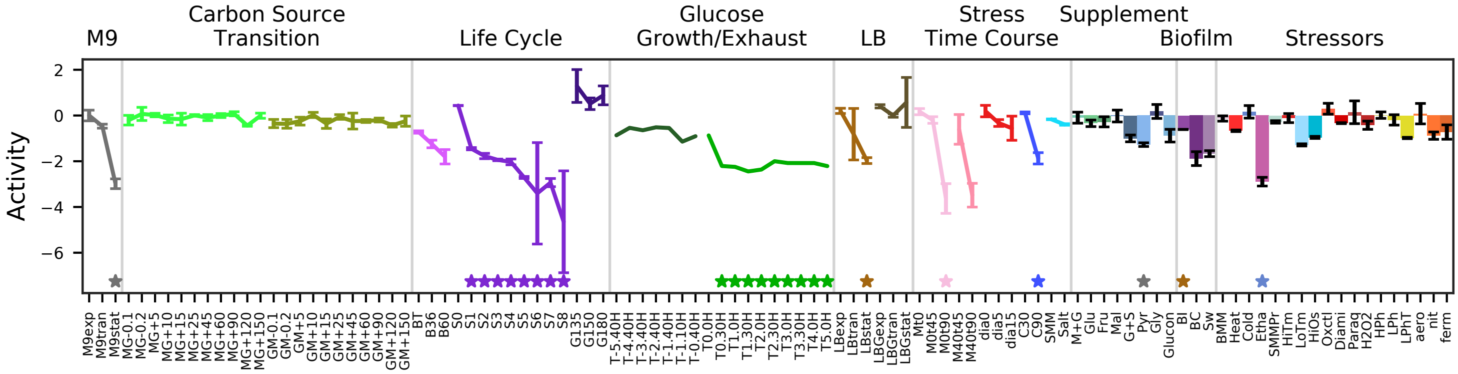

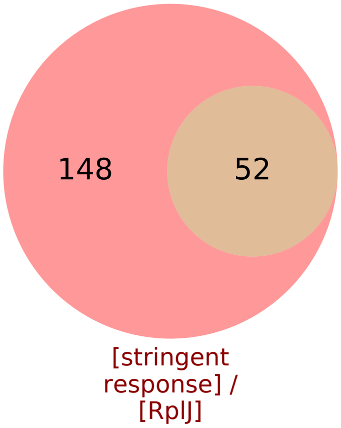

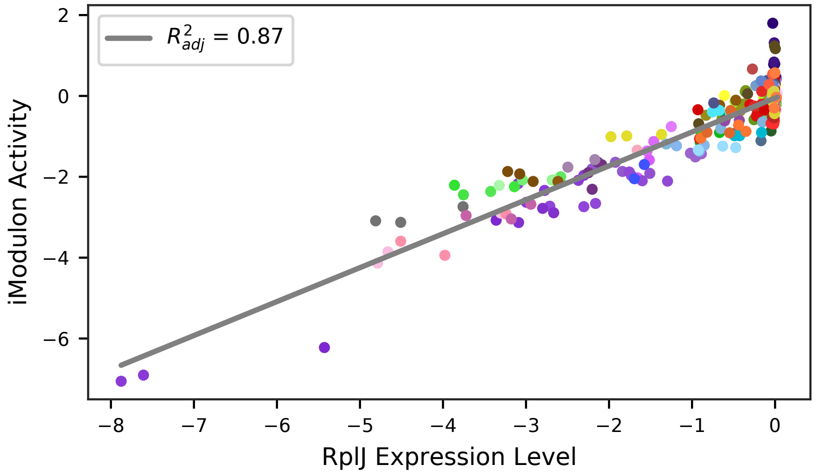

40 - SigD – Motility

Biological Function:
Production of flagella components and export machinery, chemotaxis receptors, use of extracellular energy (polyglutamic acid), regulation of swarming, sporulation inhibitors, cell wall turnover

Subset of known regulon:
SigD

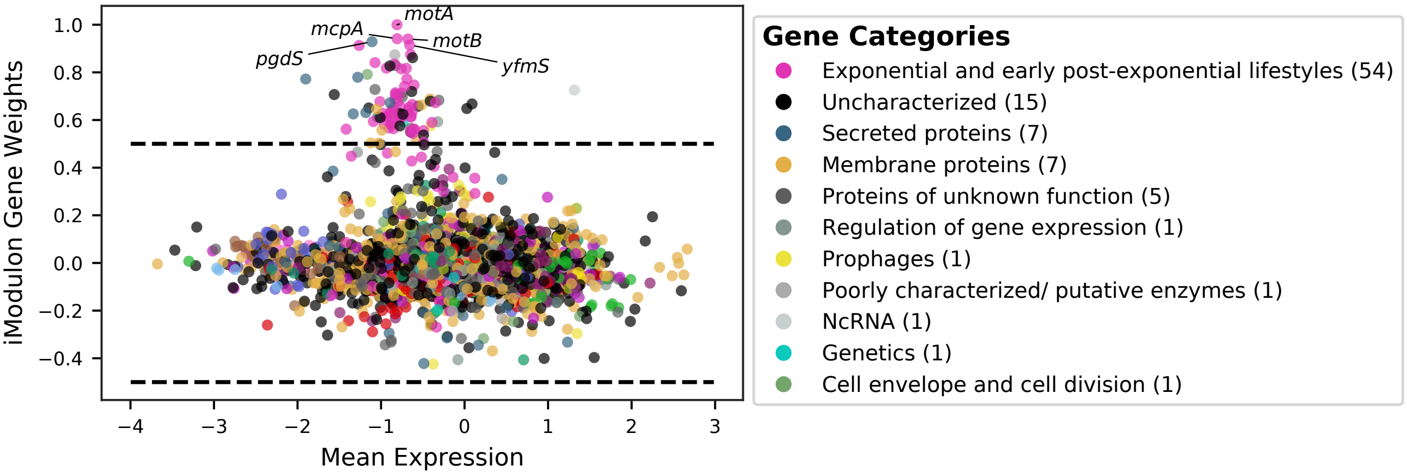

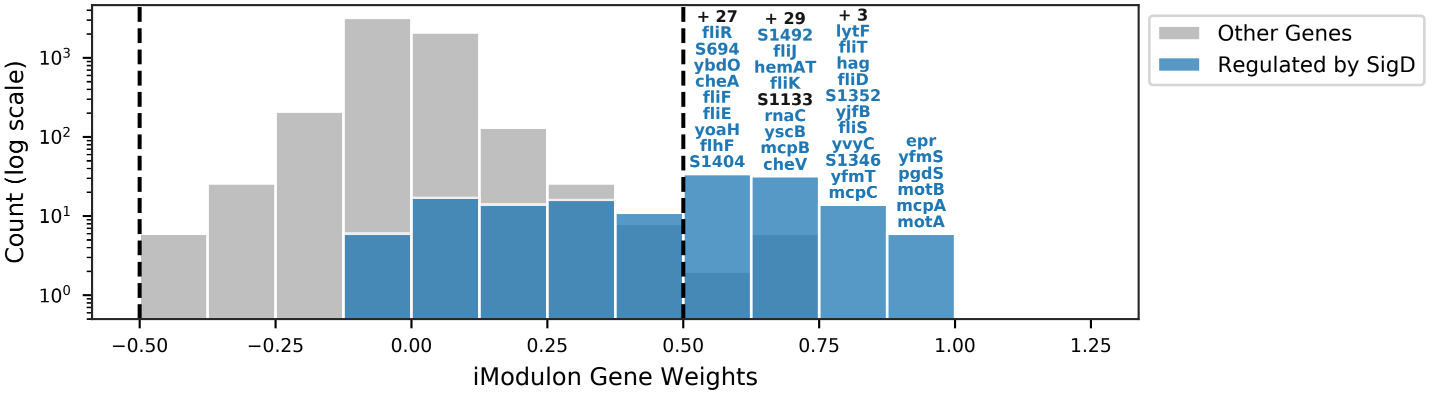

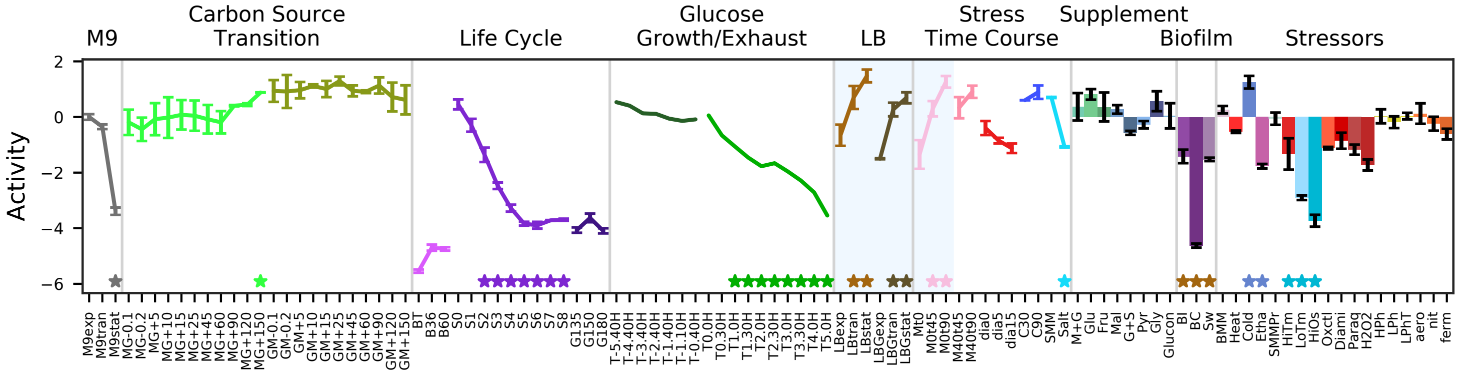

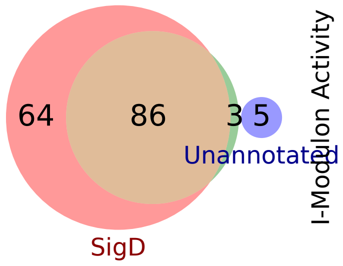

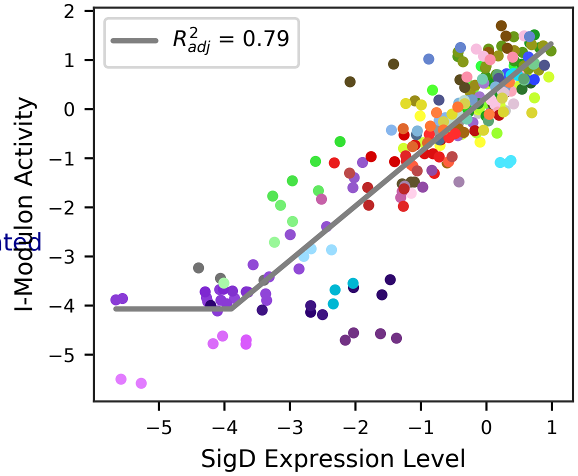

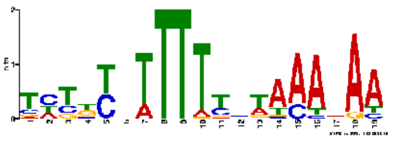

Motif E-value: 1.30e-05
Operons with Upstream Motif: 85%

41 - Eps – Exopolymeric Substances

Biological Function:
Biosynthesis of tasA and extracellular polysaccharides, the major components of the biofilm

Well-defined regulon:
SigA + AbrB + RemA + SinR

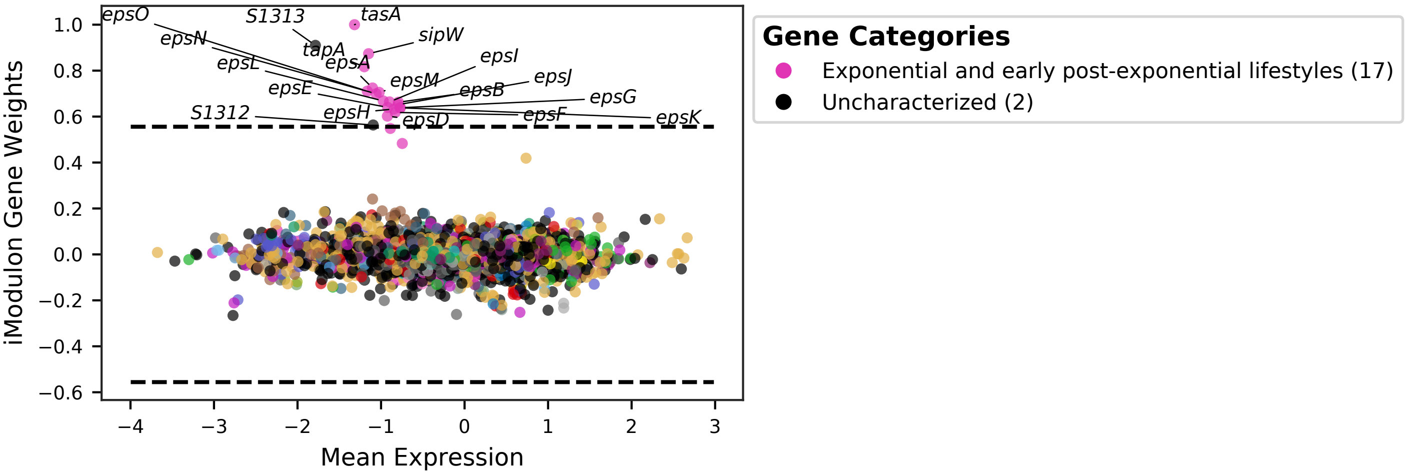

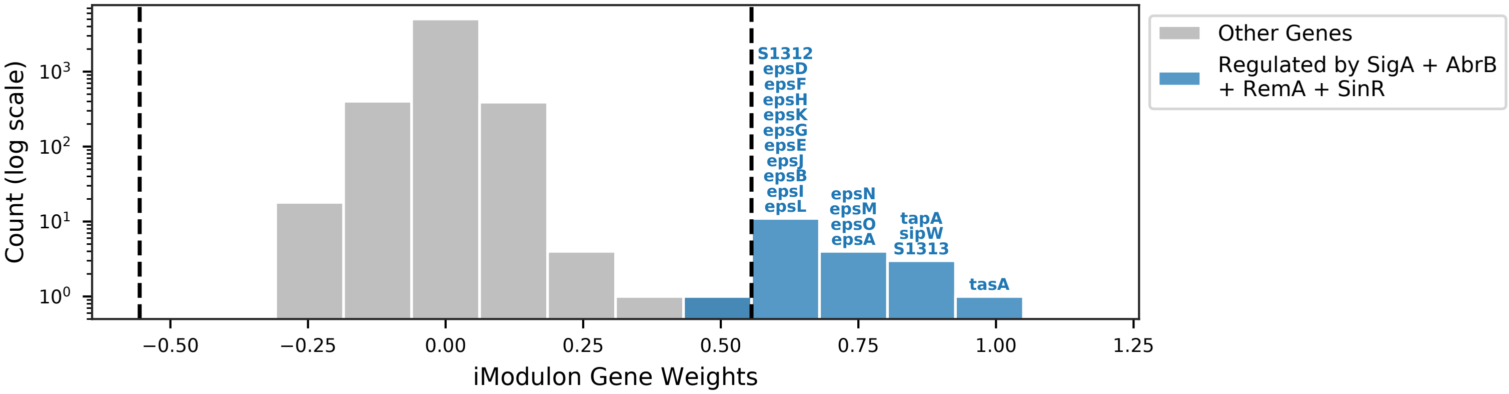

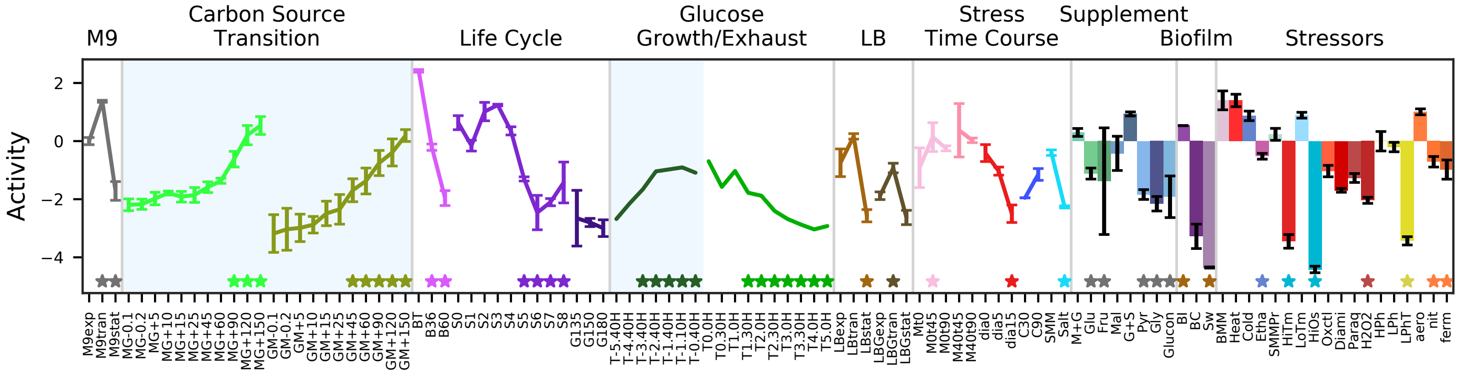

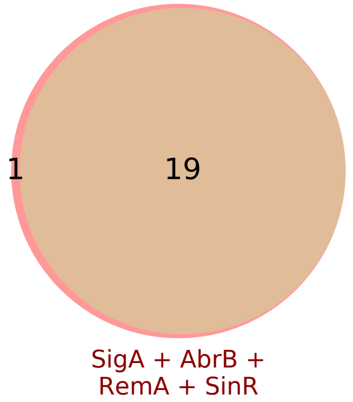

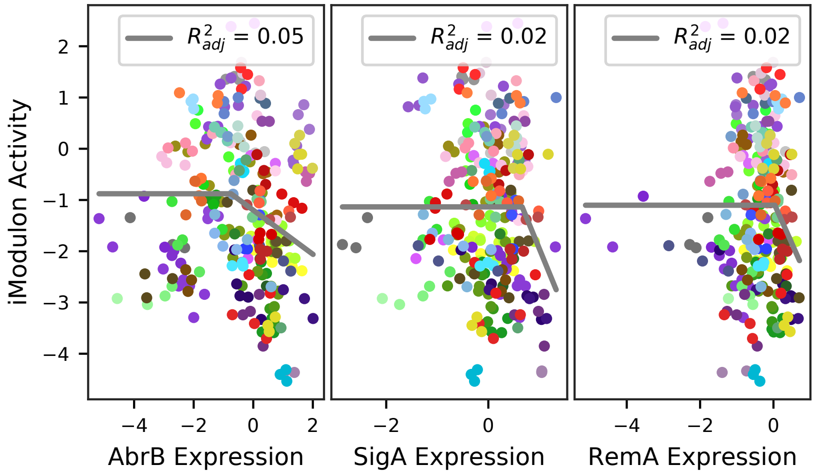

42 - ComA – Surfactants

Biological Function:
Activated during transition to stationary phase (quorum sensing), production of surfactant and exoenzymes to improve nutrient delivery in the biofilm, ComS for activation of competence

Well-defined regulon:
[ComA + CcpA + TnrA] / [Abh + SigA + ComA + Spx + PhoP + CodY + PerR]

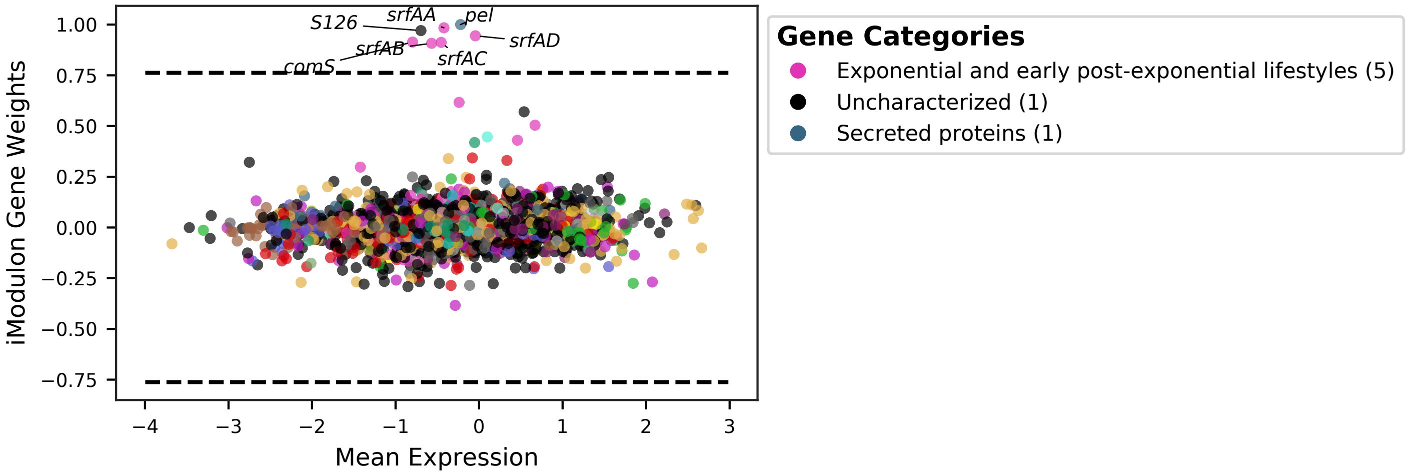

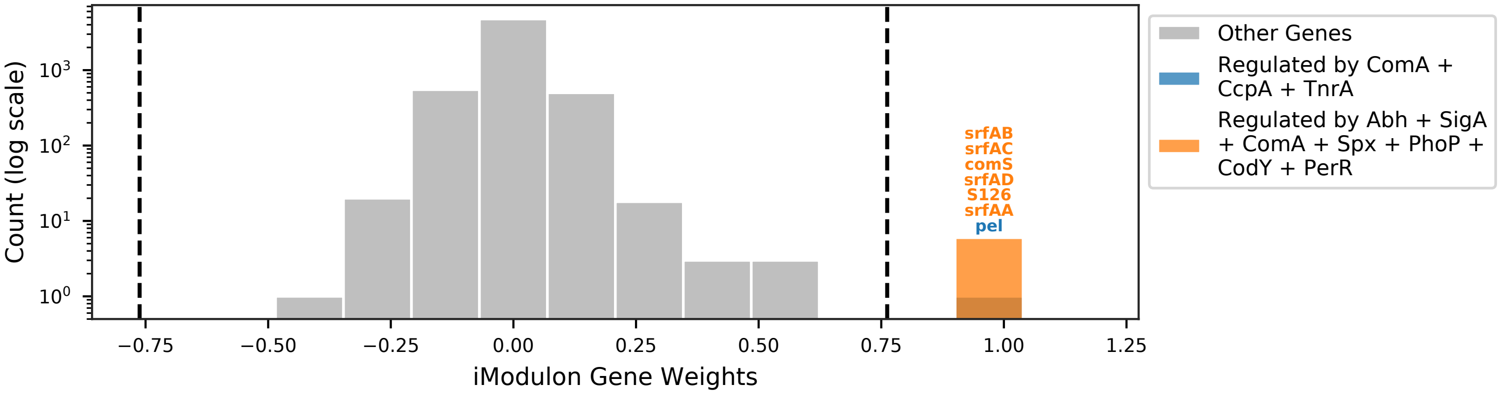

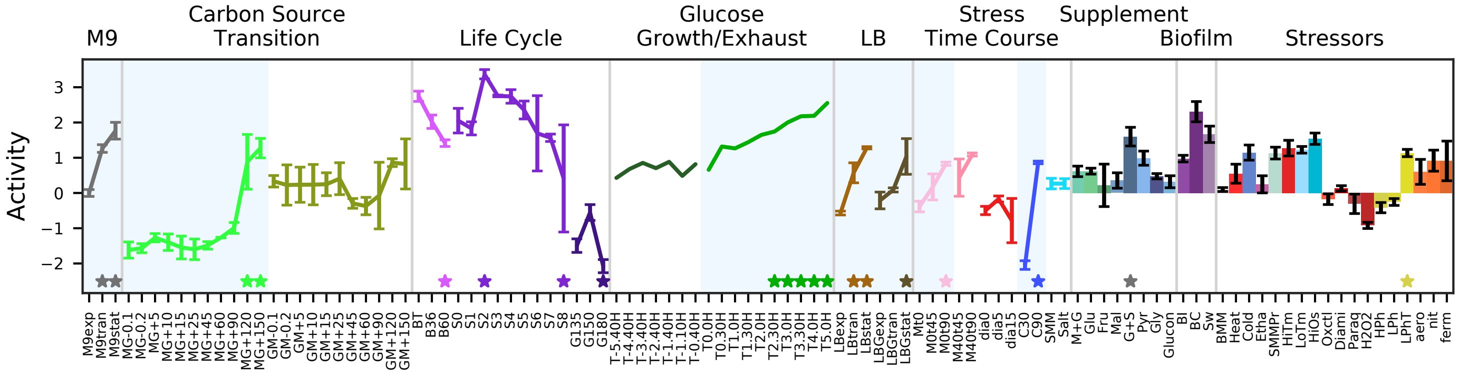

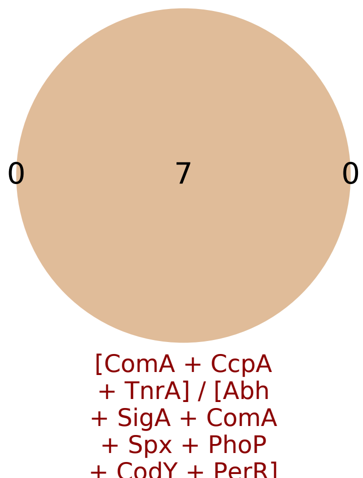

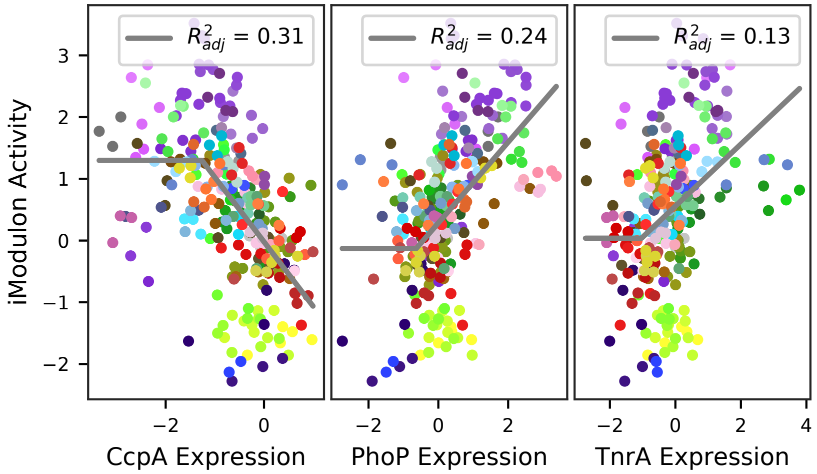

43 - DegU – Toxins and Colony Establishment

Biological Function:
Response to arrested flagellar motor. Secretion of yukE, a homolog of virulence factor EsxA. Production of antitoxins and putative toxin y-genes. Production of hydrophobic biofilm layer and lipoproteins for motility. Many unknown proteins.

Enriched for known regulon:
DegU

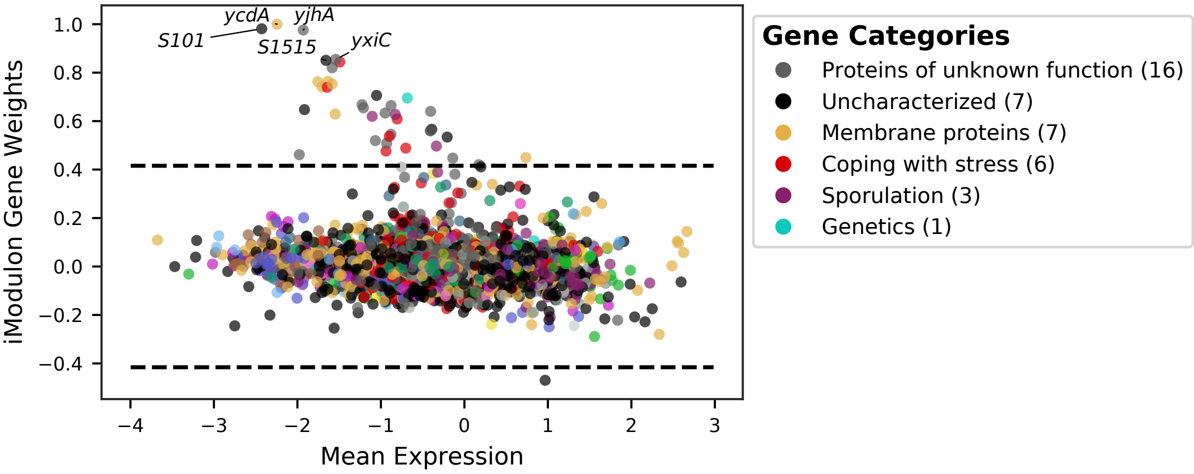

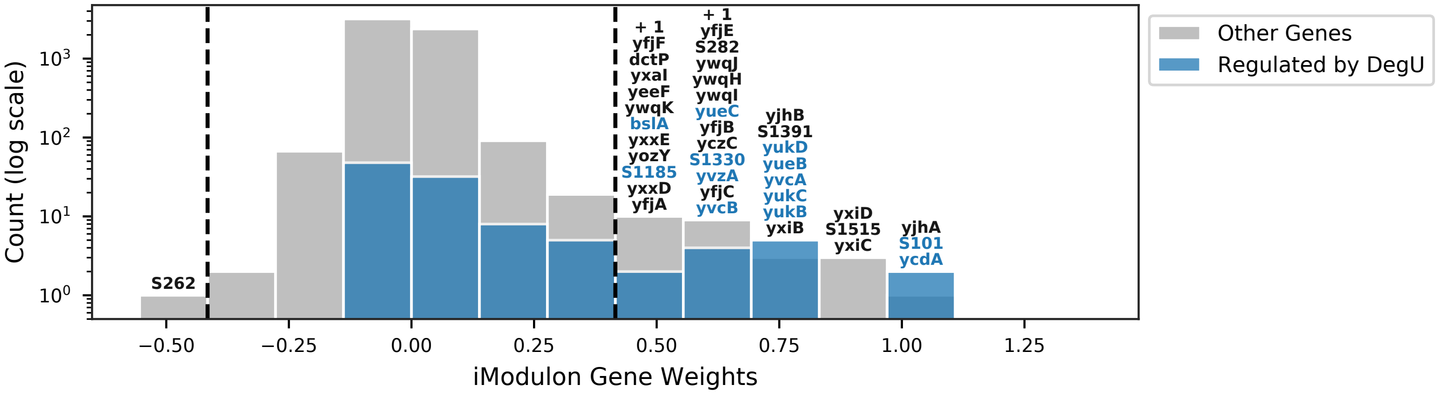

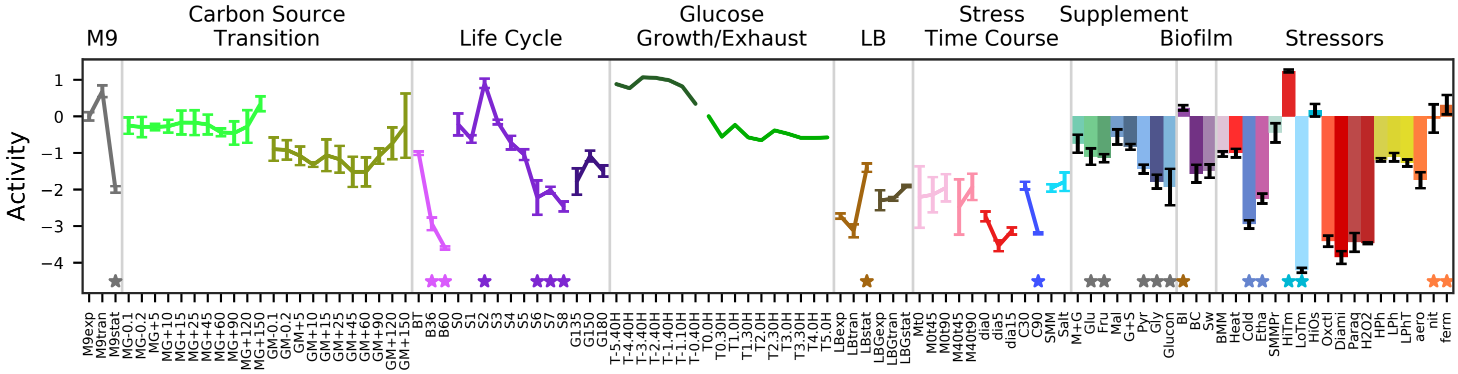

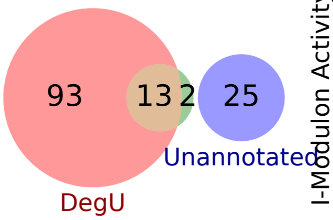

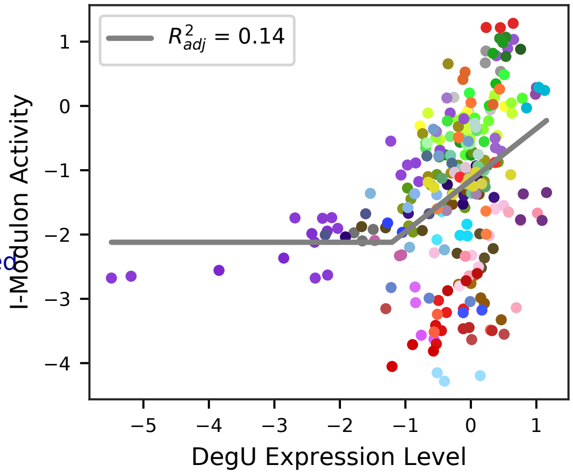

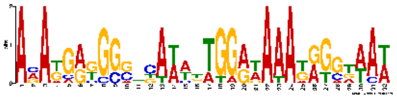

Motif E-value: 2.80e-04
Operons with Upstream Motif: 47%

44 - AbrB – Transition State

Biological Function:
Various transition state roles: spore killing, antibiotic resistance, toxin production, metabolism, protein modification, and unknown proteins.

Subset of known regulon:
AbrB

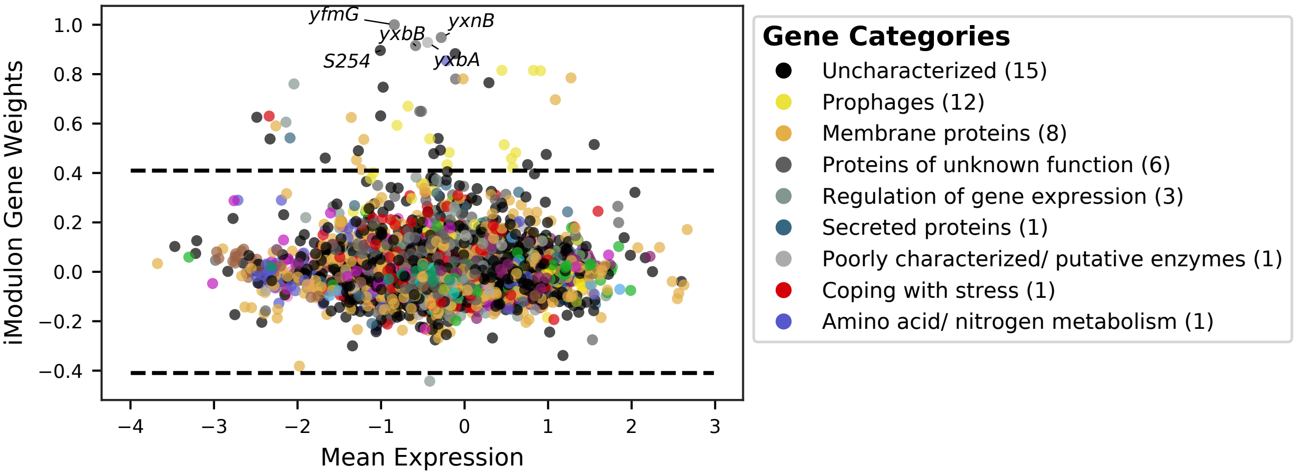

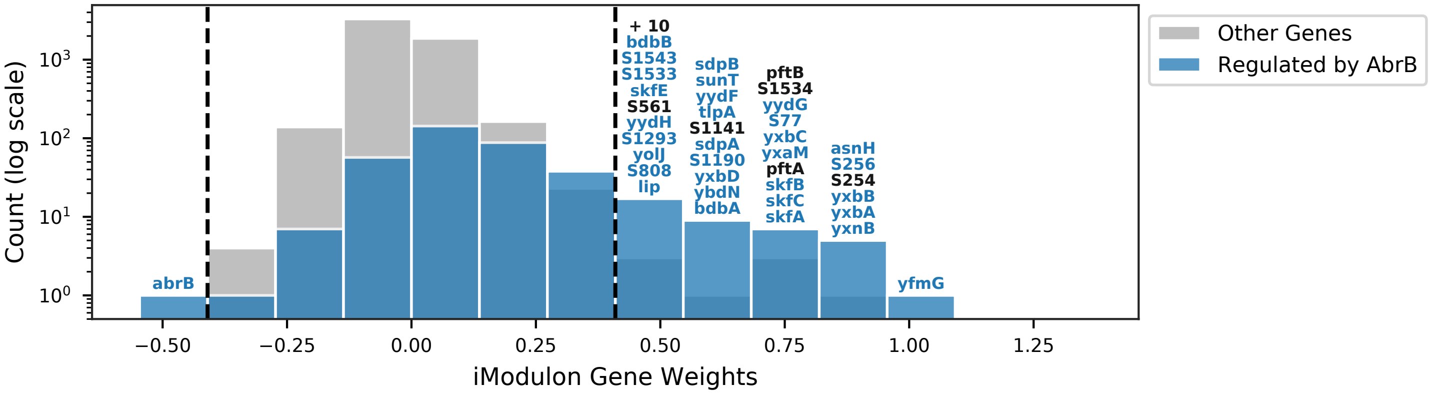

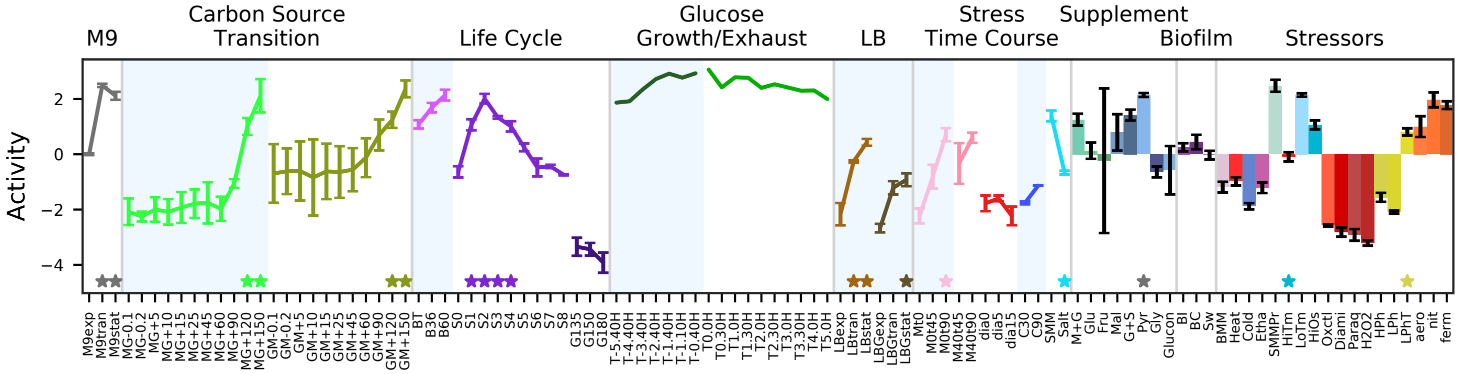

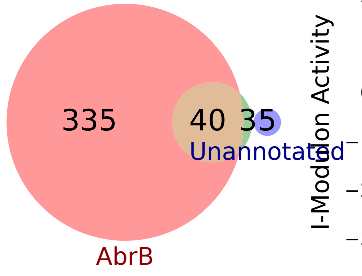

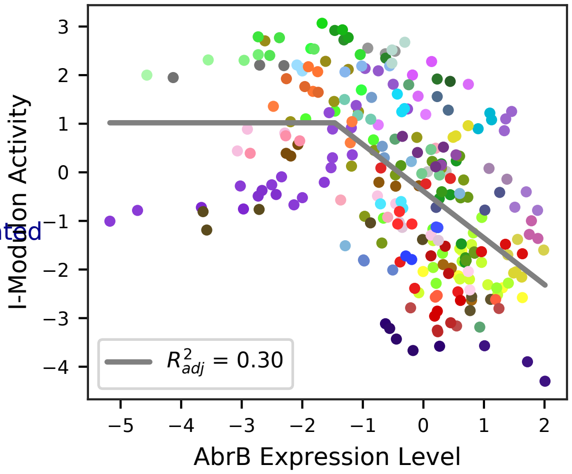

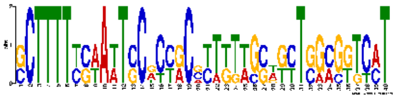

Motif E-value: 2.00e-06
Operons with Upstream Motif: 81%

45 - Alb – Antilisterial Bacteriocin

Biological Function:
Produces and exports subtilosin, which kills anaerobic listeria bacteria

Well-defined regulon:
Rok + ResD + AbrB + SigA

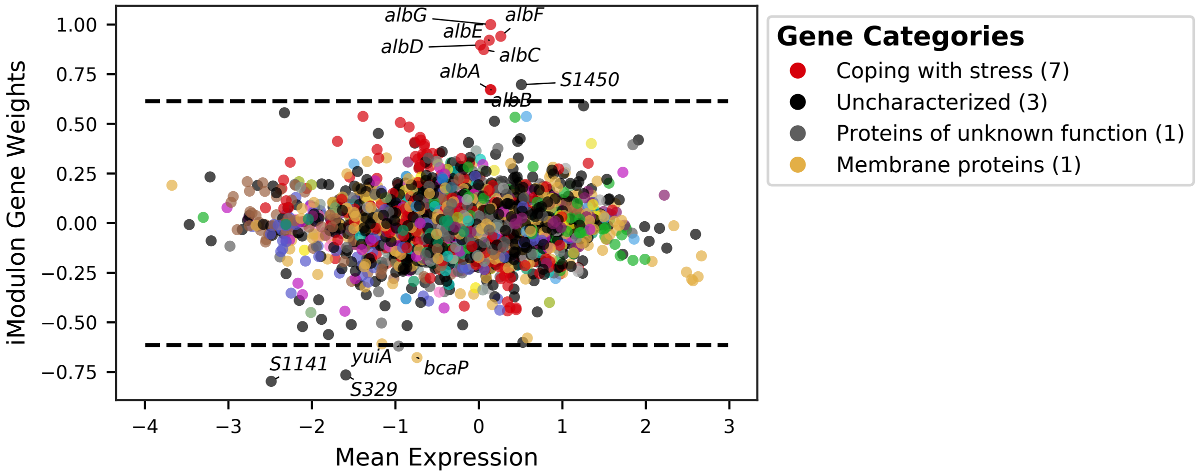

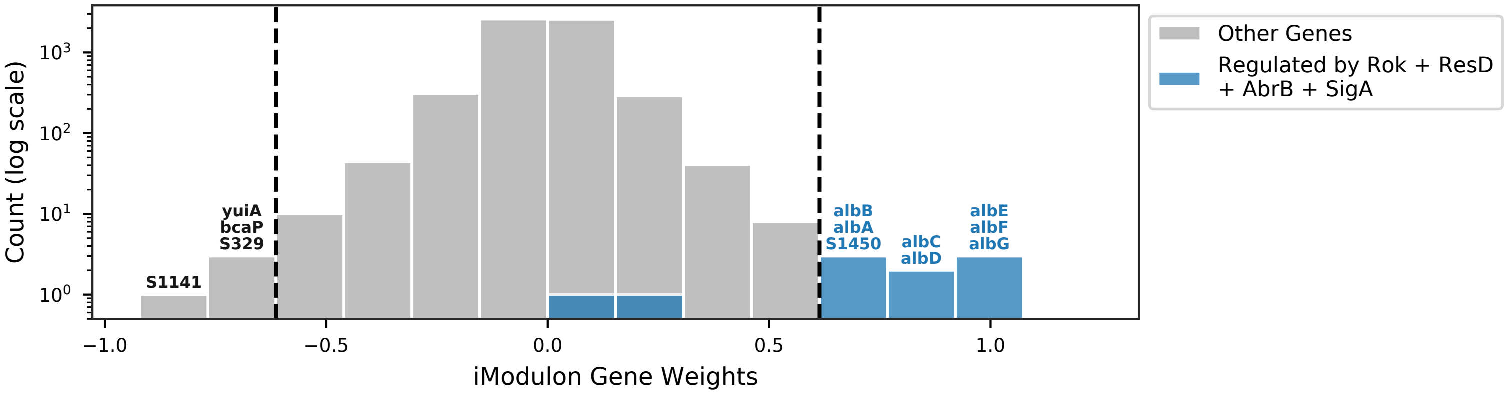

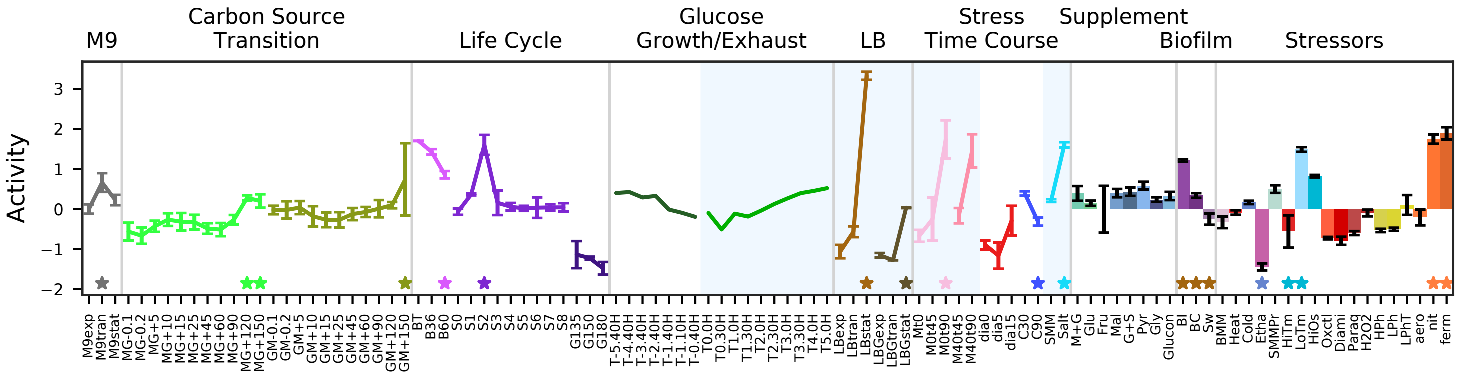

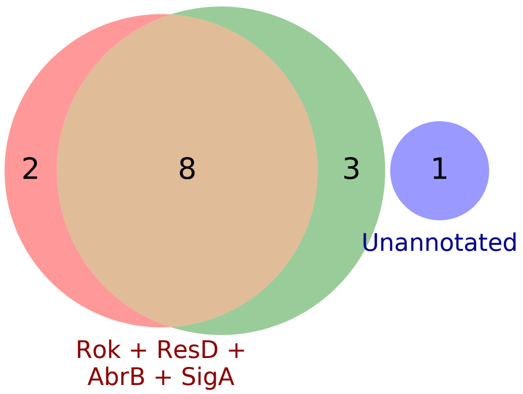

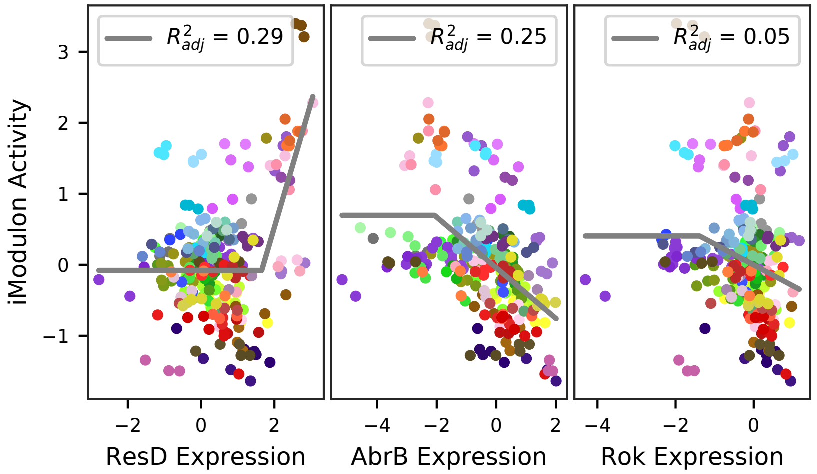

46 - WapA – Intercellular Competition

Biological Function:
Activation of WapA and its operon of unknown genes for interspecies toxin delivery

Contains unknown genes and known regulon:
SigA + YvrHb + DegU + WalR

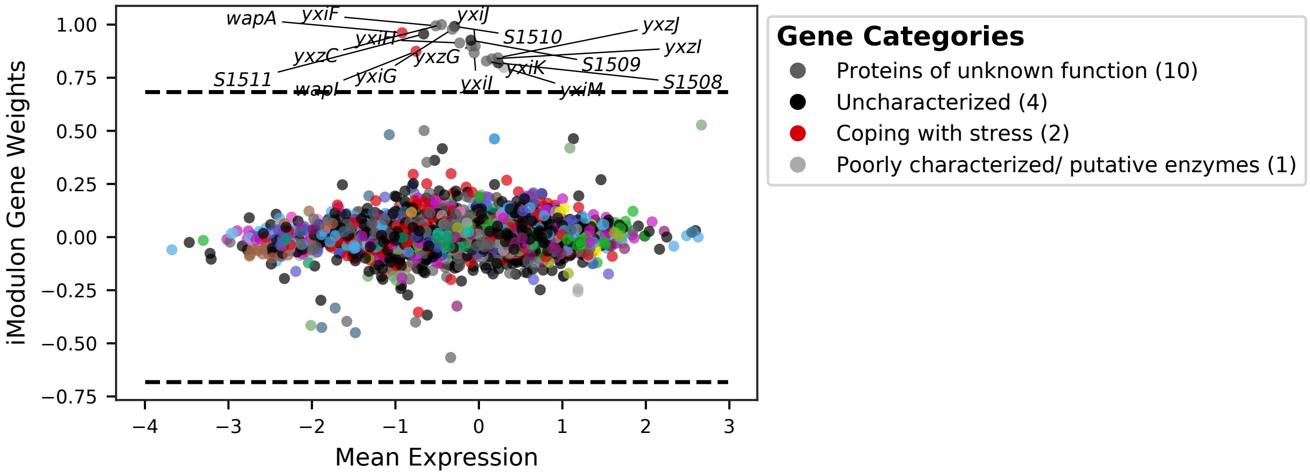

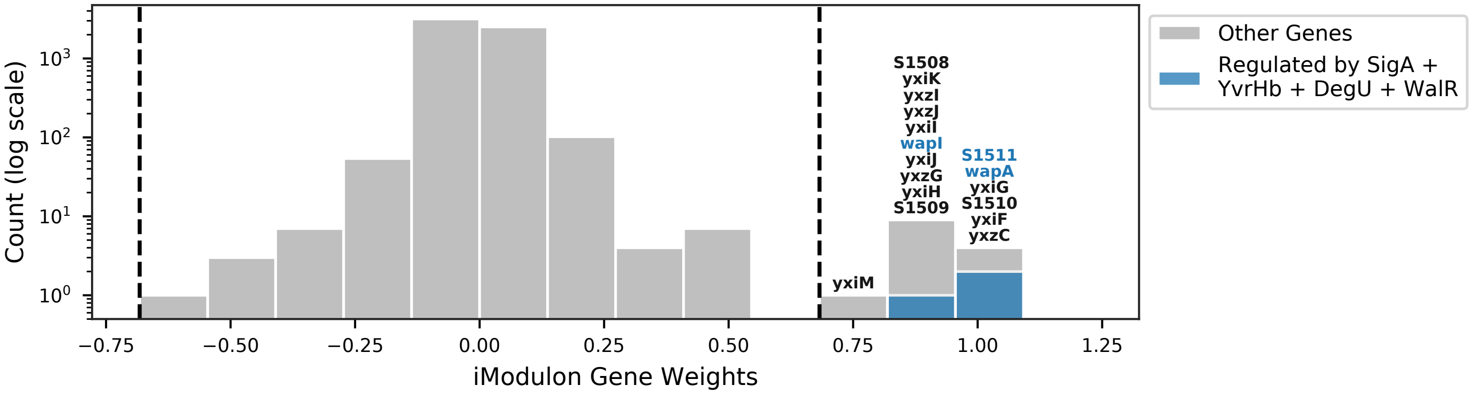

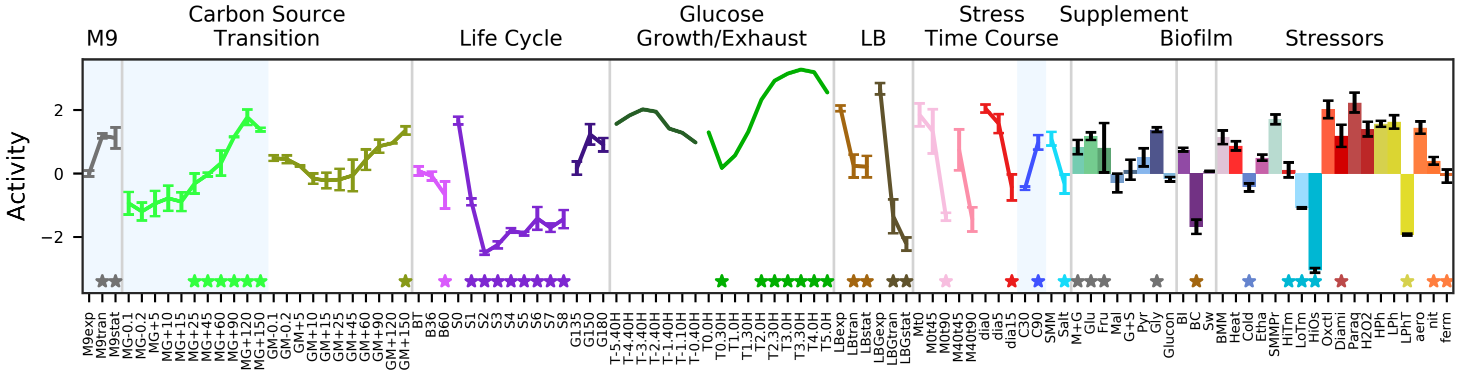

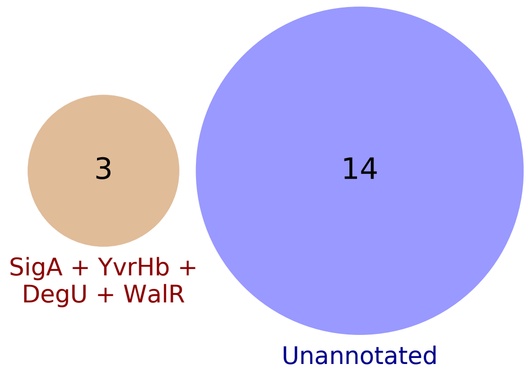

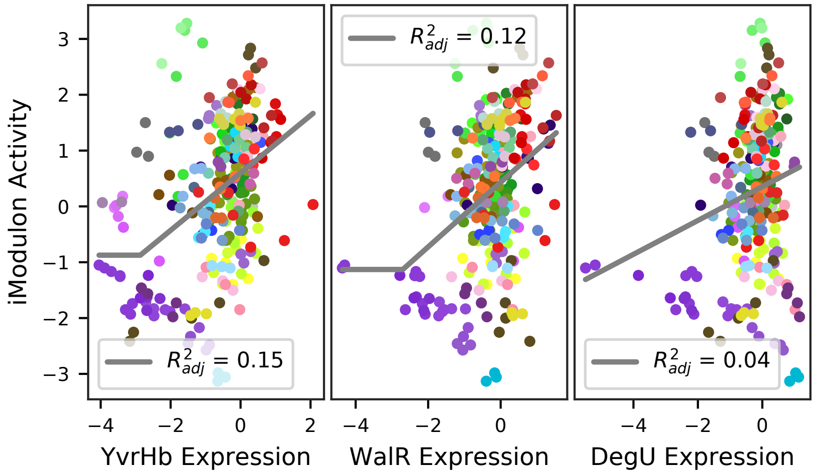

47 - ComK – Competence

Biological Function:
DNA transport, pseudopilus, recombination machinery, and regulatory proteins for competence

Enriched for known regulon:
ComK

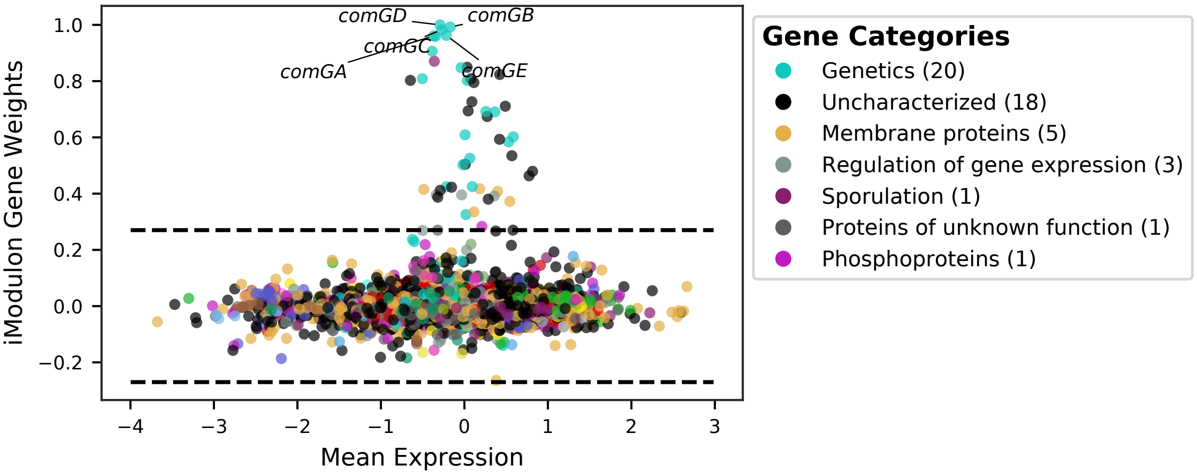

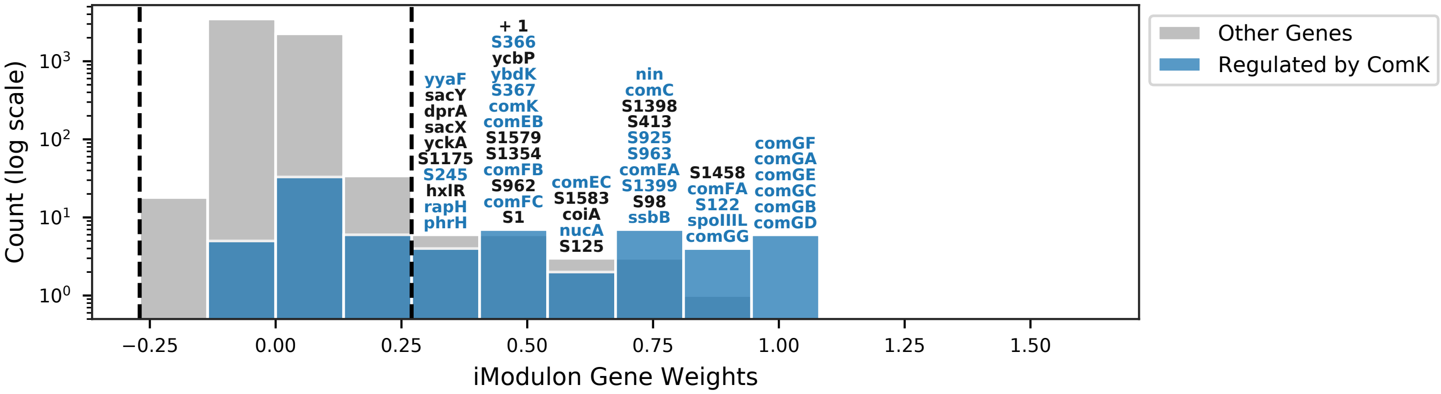

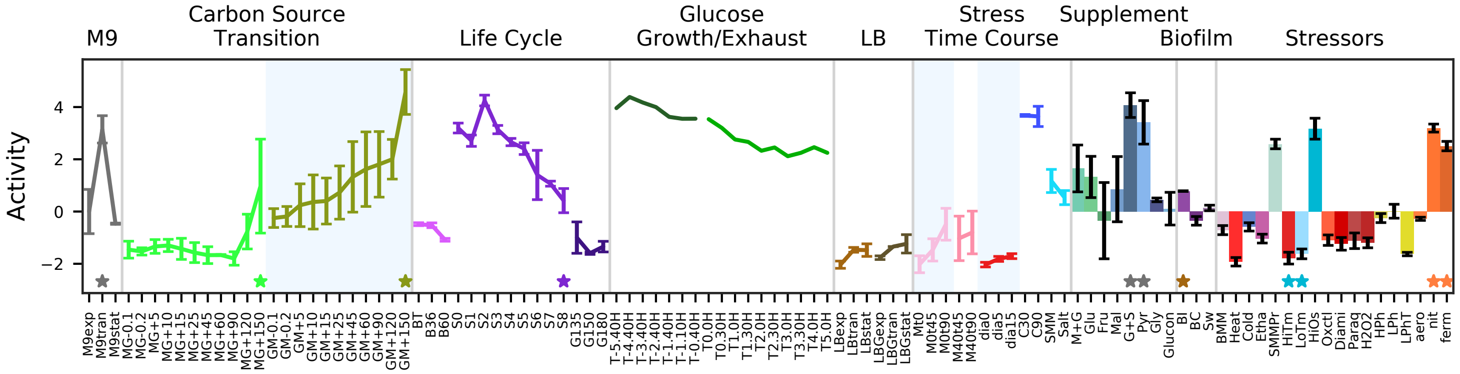

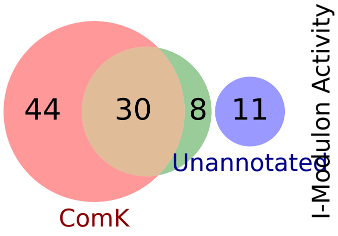

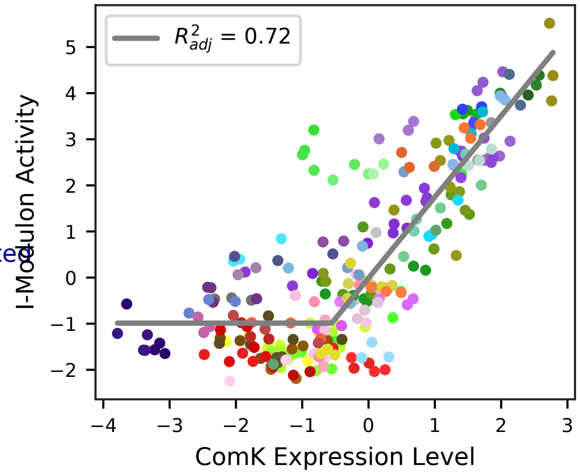

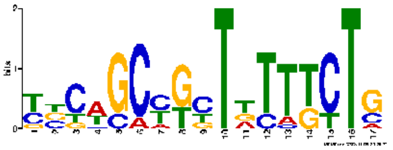

Motif E-value: 8.50e-05
Operons with Upstream Motif: 65%

48 - Spo0A – Sporulation 1

Biological Function:
Initiation of sporulation through sigma factor, transcription factor, and regulatory protein expression, as well as septum formation and chromosome anchoring

Subset of known regulon:
[Spo0A] / [DnaA]

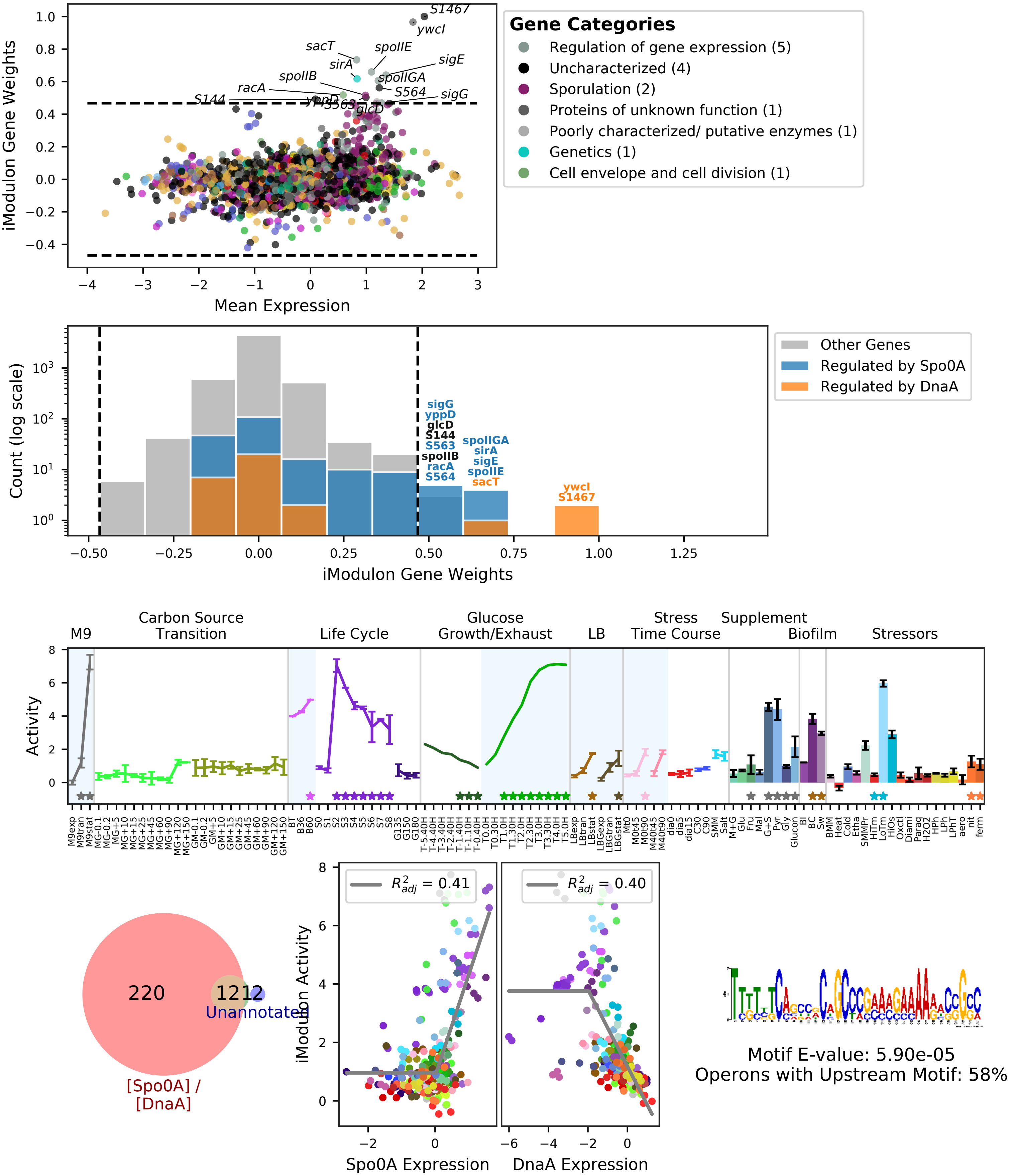

49 - SigE – Sporulation 2

Biological Function:
Spore maturation through core dehydration, nutrient transport and utilization, septum degradation, inner cortex synthesis, and regulator activation

Enriched for known regulon:
SigE

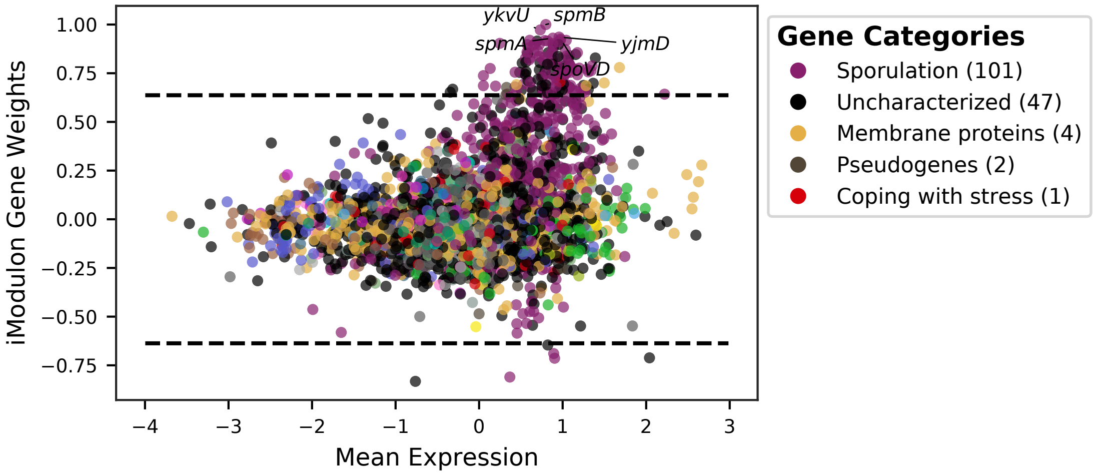

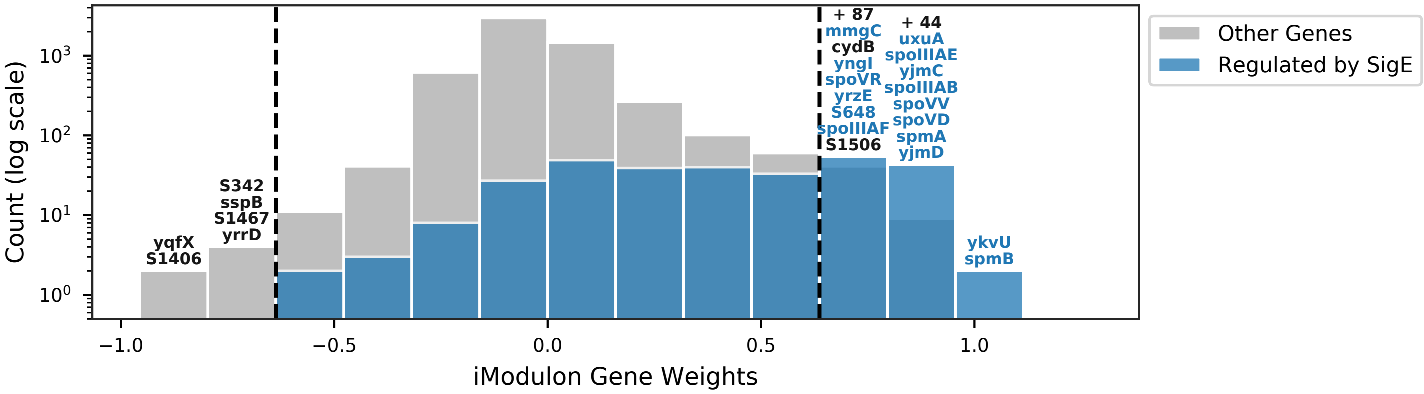

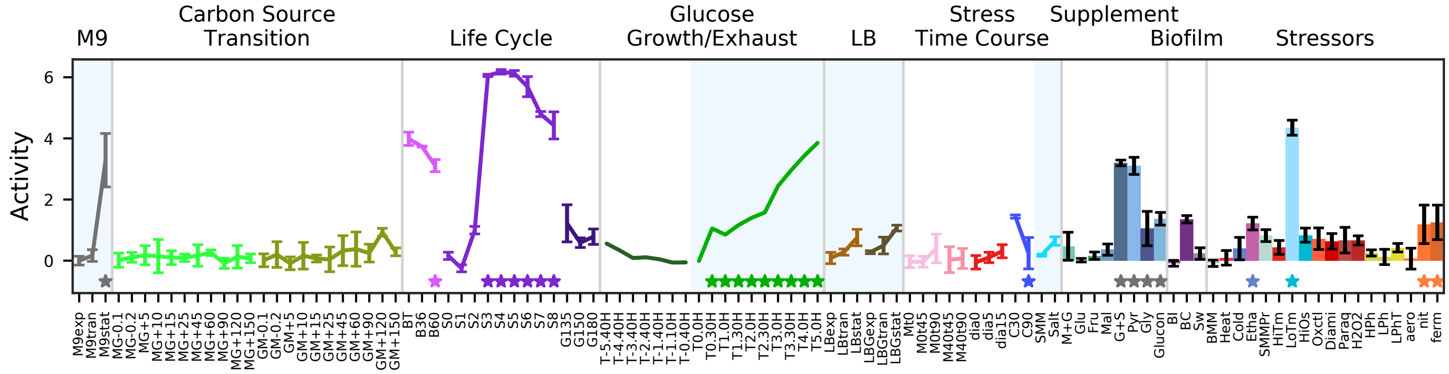

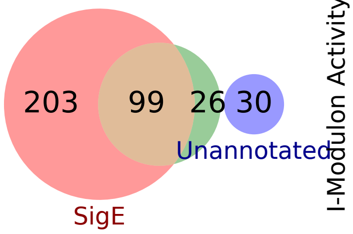

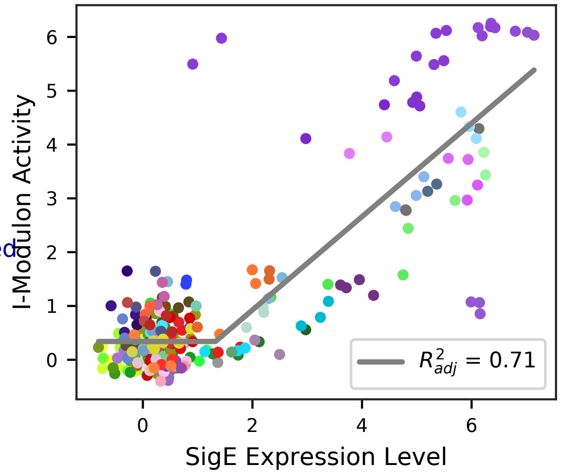

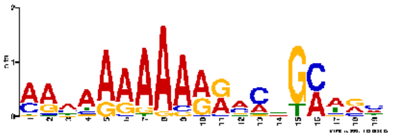

Motif E-value: 2.40e-15
Operons with Upstream Motif: 86%

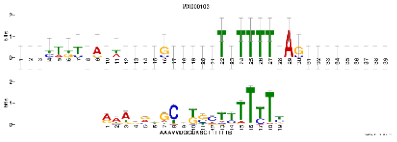

Motif similar to ExsA -
P. aeruginosa (E = 3.38e-03)

50 - SigE / G – Sporulation 3

Biological Function:
Spore coat production, transcription factor and regulator expression, spore DNA protection, spore stress resistance

Subset of known regulon:
[SigE] / [SigG]

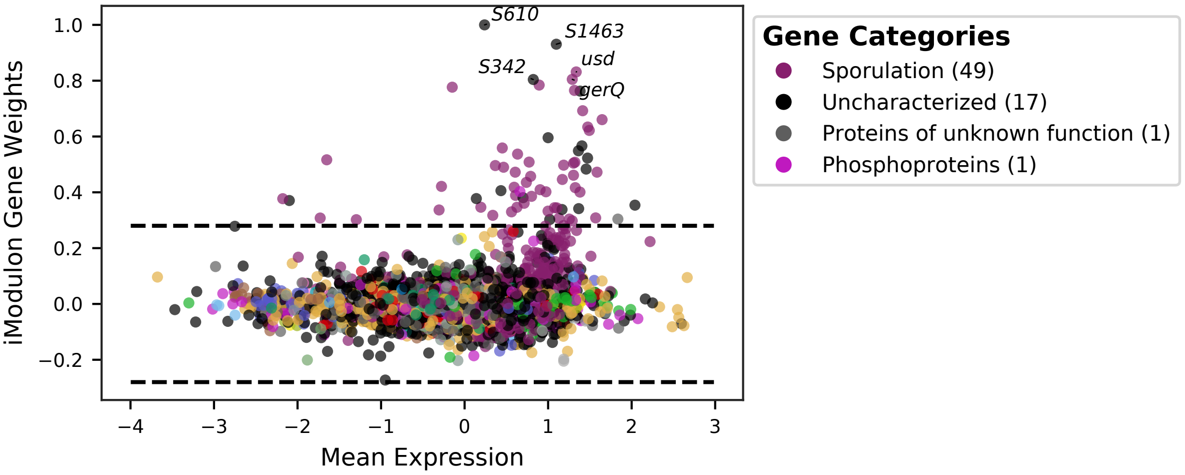

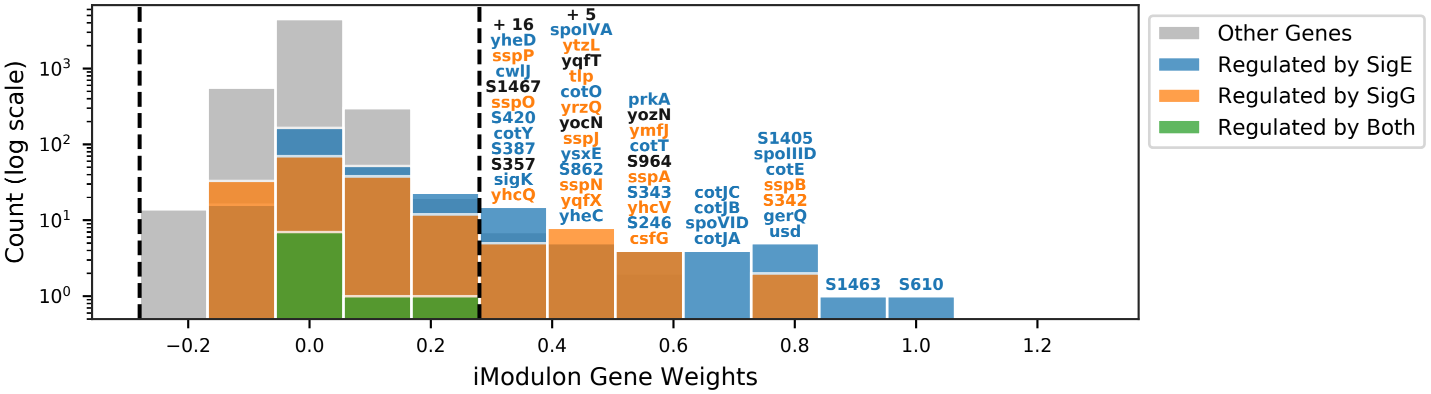

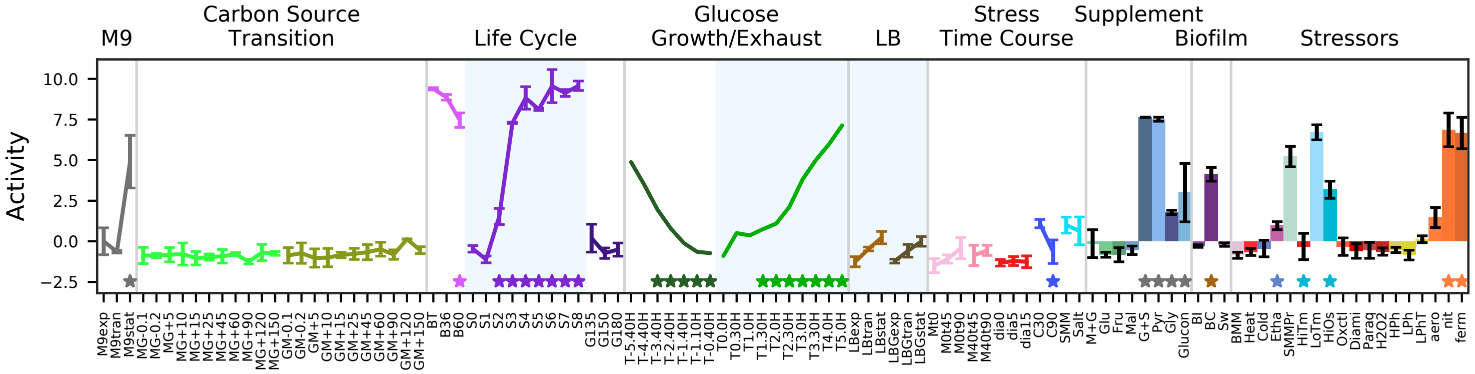

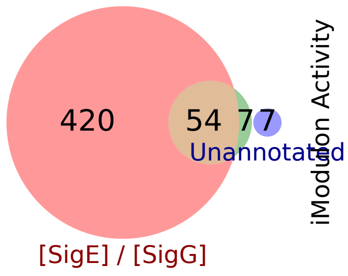

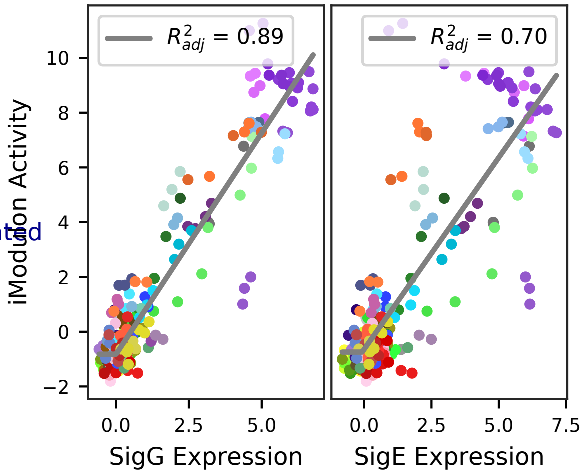

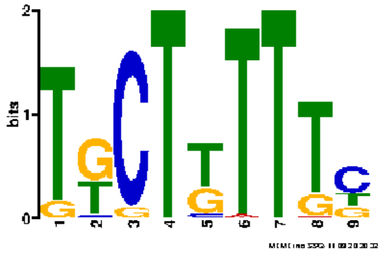

Motif E-value: 9.00e-10
Operons with Upstream Motif: 85%

51 - SigG – Sporulation 4

Biological Function:
Calcium and nutrient delivery to the spore, endospore cortex maturation, lipoproteins, forespore-specific metabolic proteins, many unknown sporulation proteins, stress resistance, proteins required for germination

Enriched for known regulon:
SigG

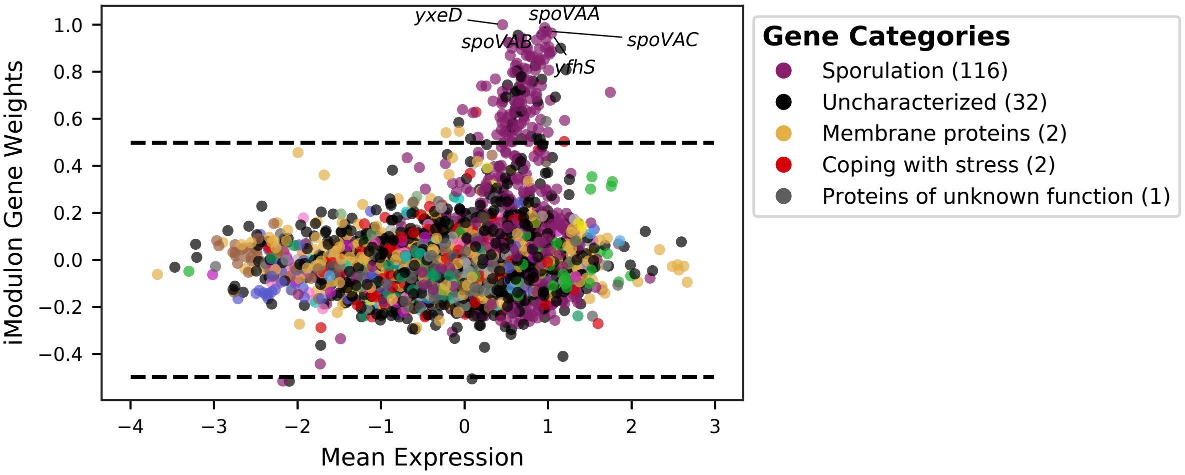

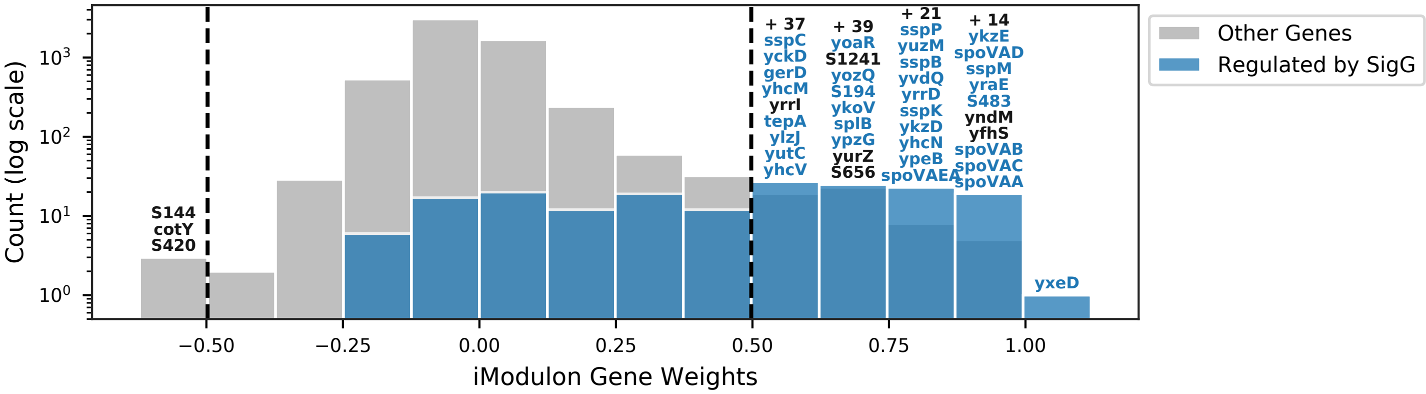

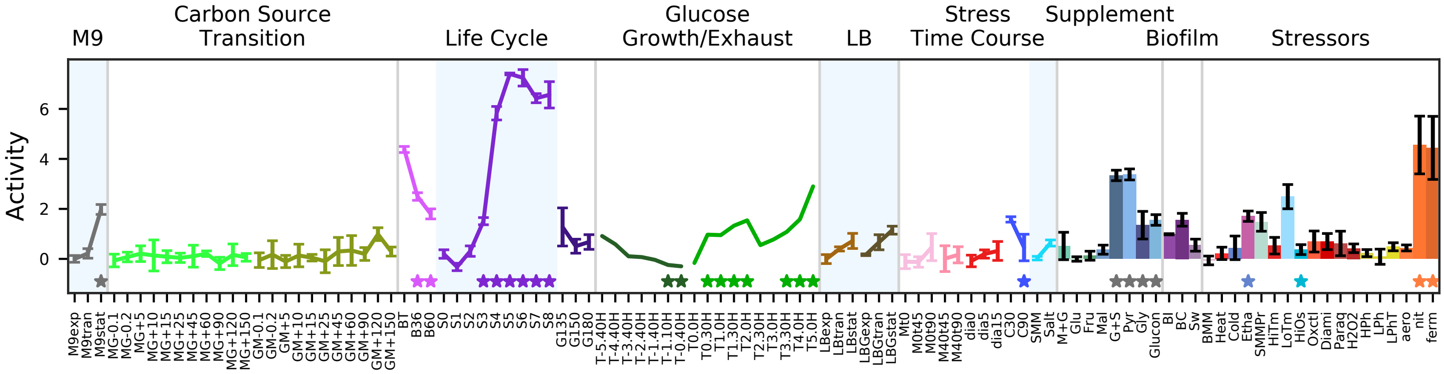

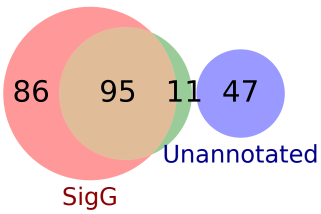

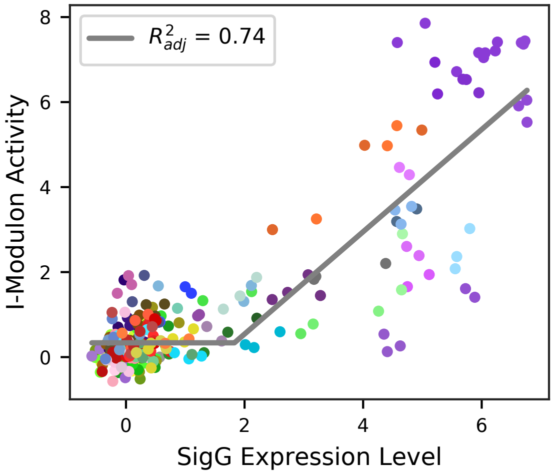

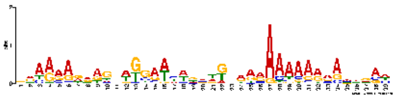

Motif E-value: 1.10e-27
Operons with Upstream Motif: 95%

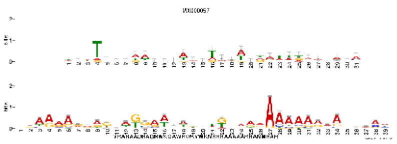

Motif similar to PhoP (E = 6.66e-06)

52 - SigK-1 – Sporulation 5

Biological Function:
Production of spore coat and crust proteins, L-sulfolactate production, spore coat cross-linking, stress resistance, many unknown proteins

Enriched for known regulon:
SigK

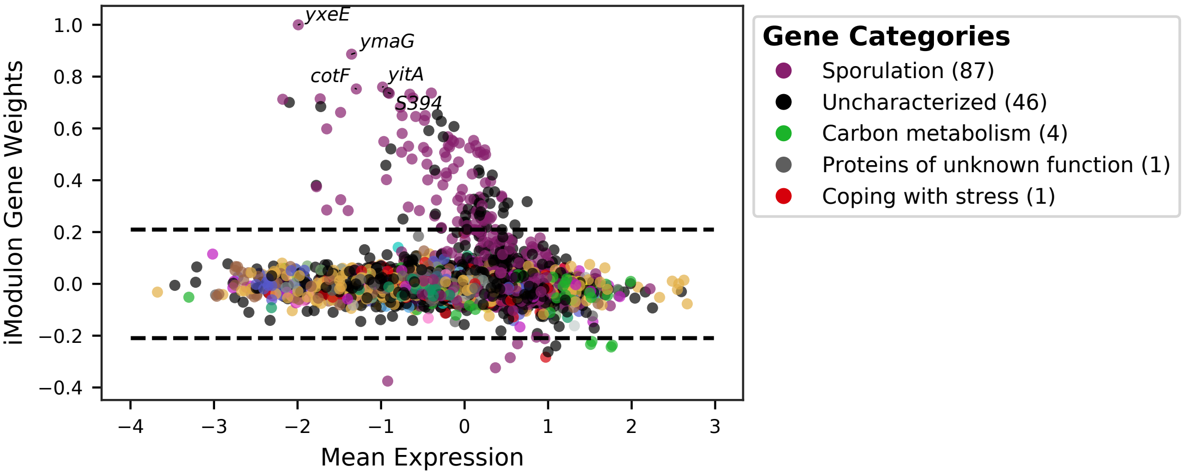

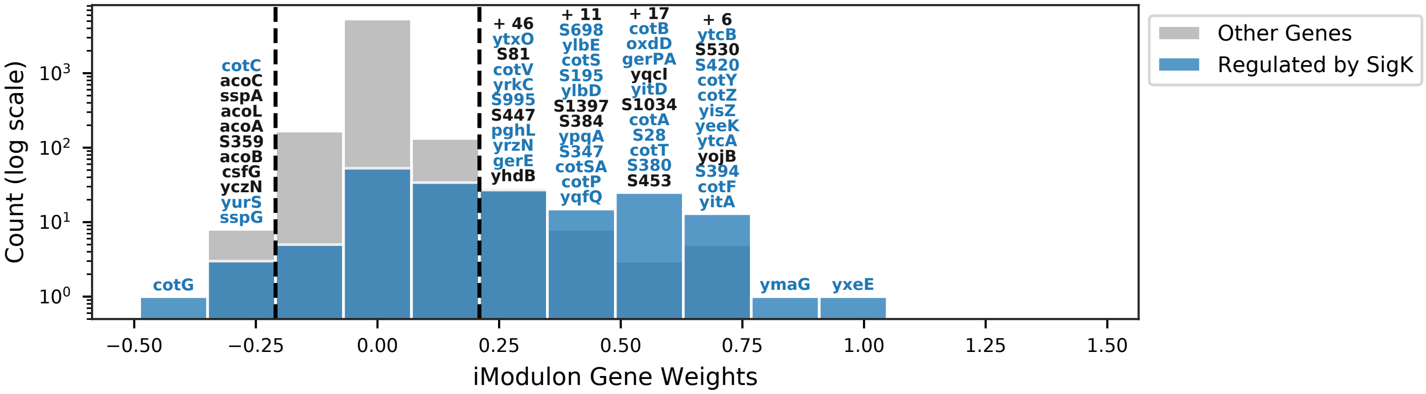

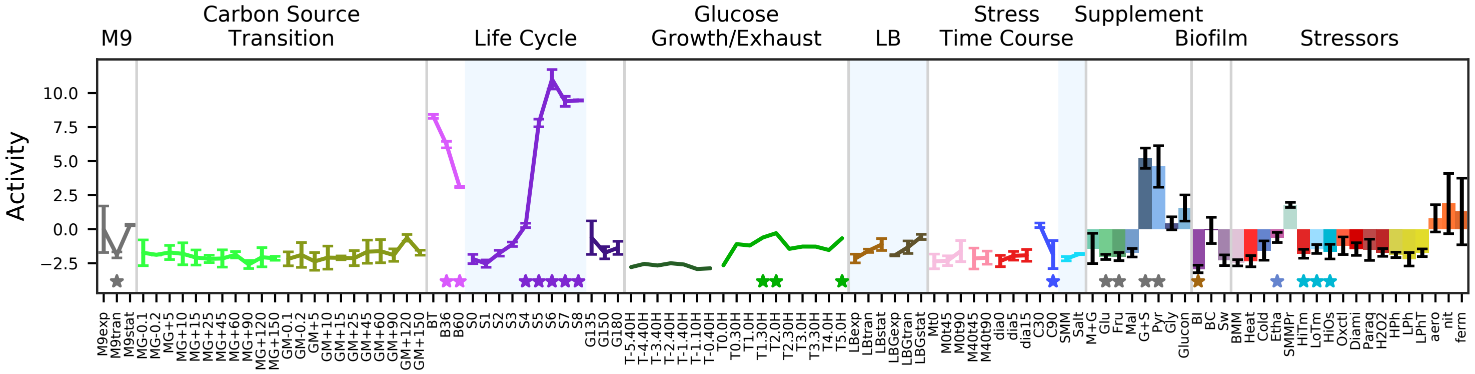

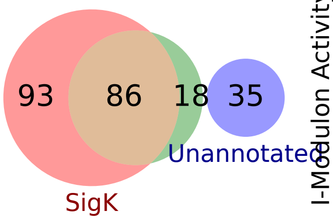

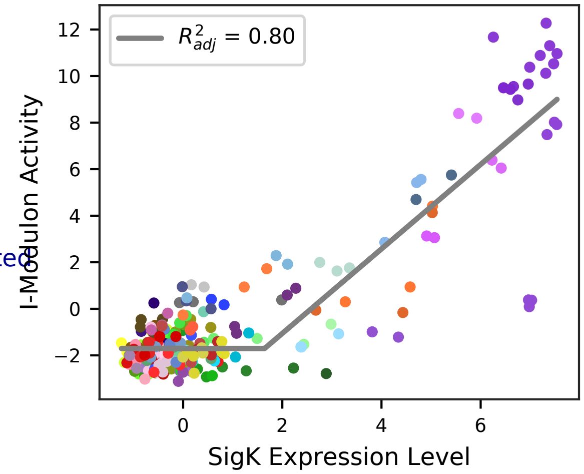

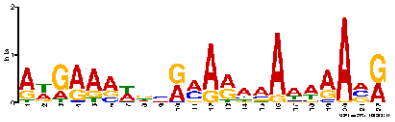

Motif E-value: 5.80e-18
Operons with Upstream Motif: 84%

53 - SigK-2 – Sporulation 6

Biological Function:
Production of outermost spore coat and crust proteins, mother cell lysis

Enriched for known regulon:
SigK

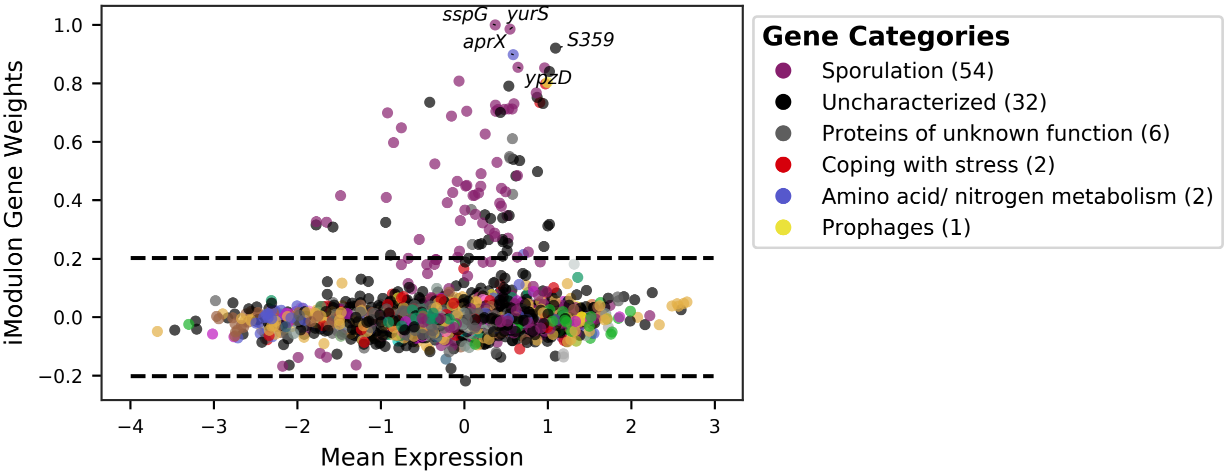

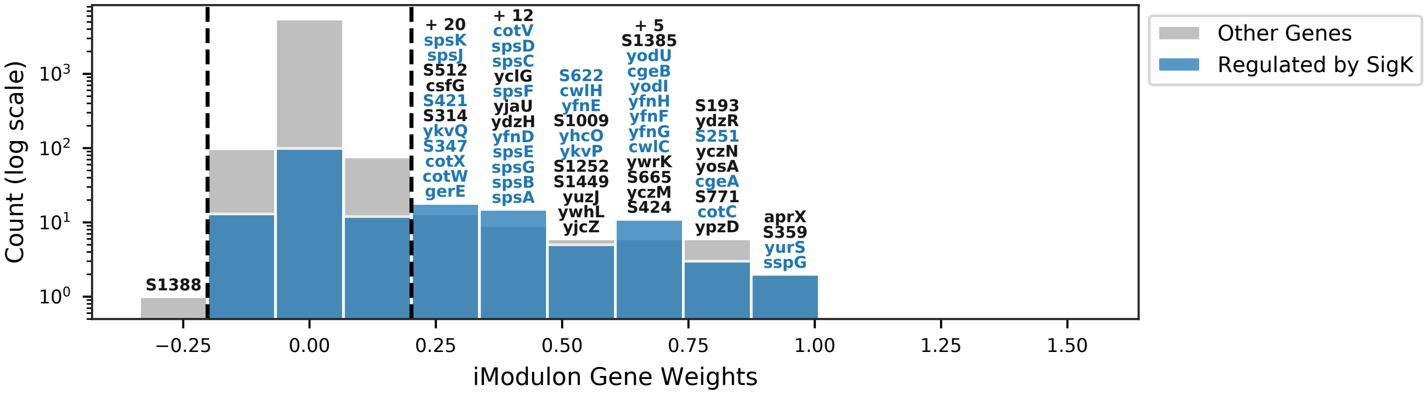

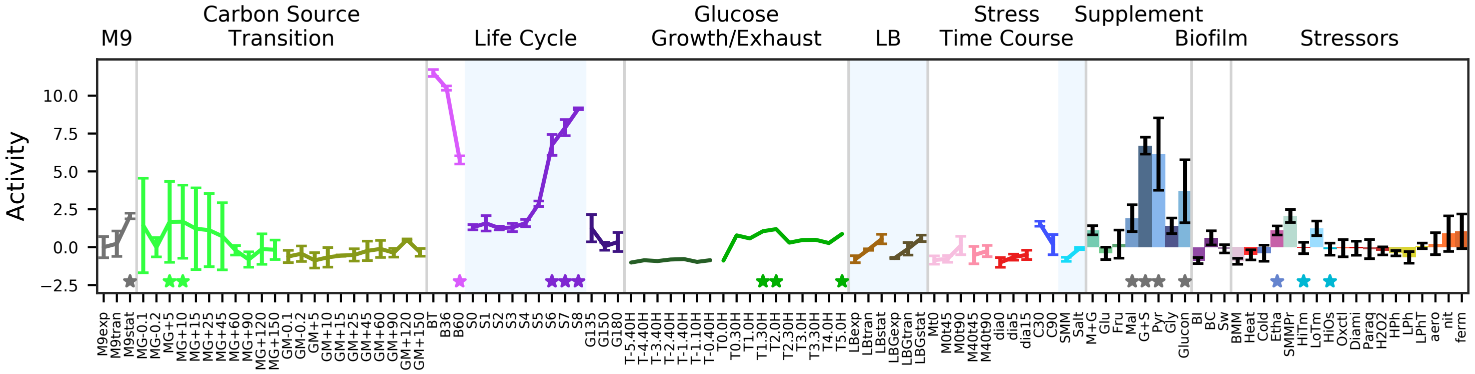

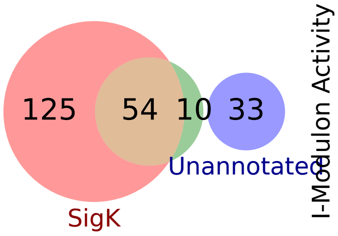

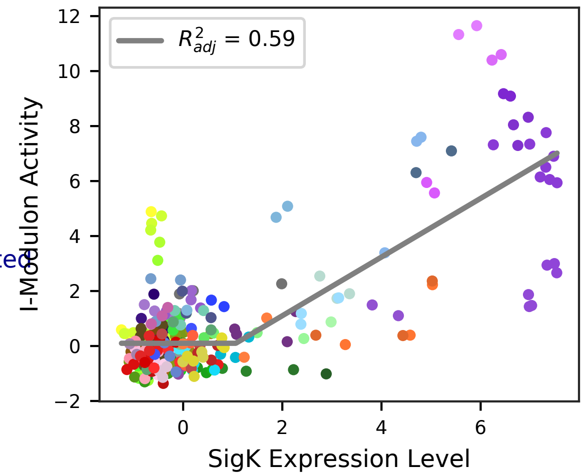

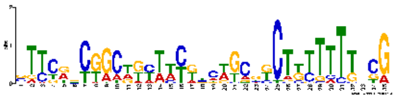

Motif E-value: 1.20e-10
Operons with Upstream Motif: 64%

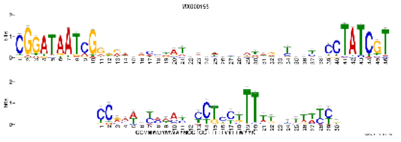

Motif similar to OxyR - E. coli (E = 5.60e-03)

54 - GerE / GerR – Mid-late Spore Coat

Biological Function:
Production of spore coat and crust proteins. Omitted from sporulation analysis because all genes are contained by SigK1 & 2, and activity levels are very noisy in non-sporulating conditions.

Subset of known regulon:
[SigK + GerE] / [SigK + GerR]

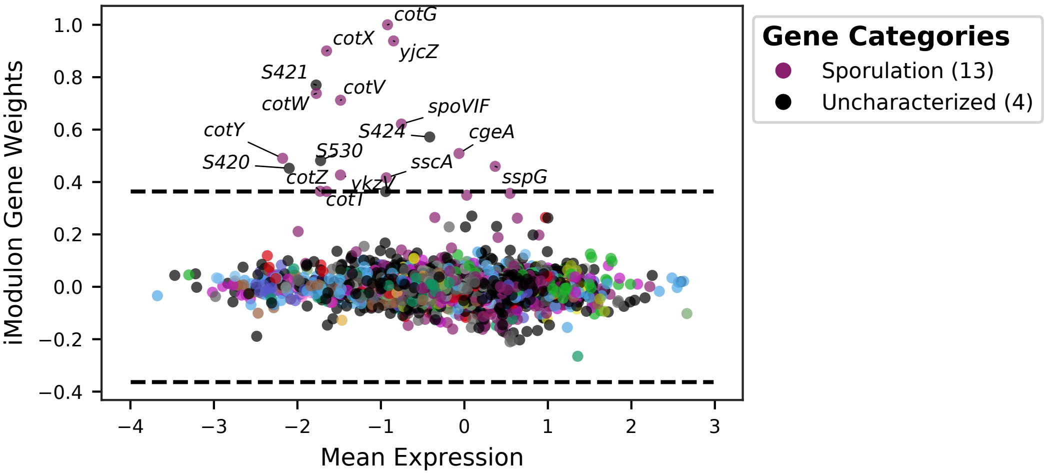

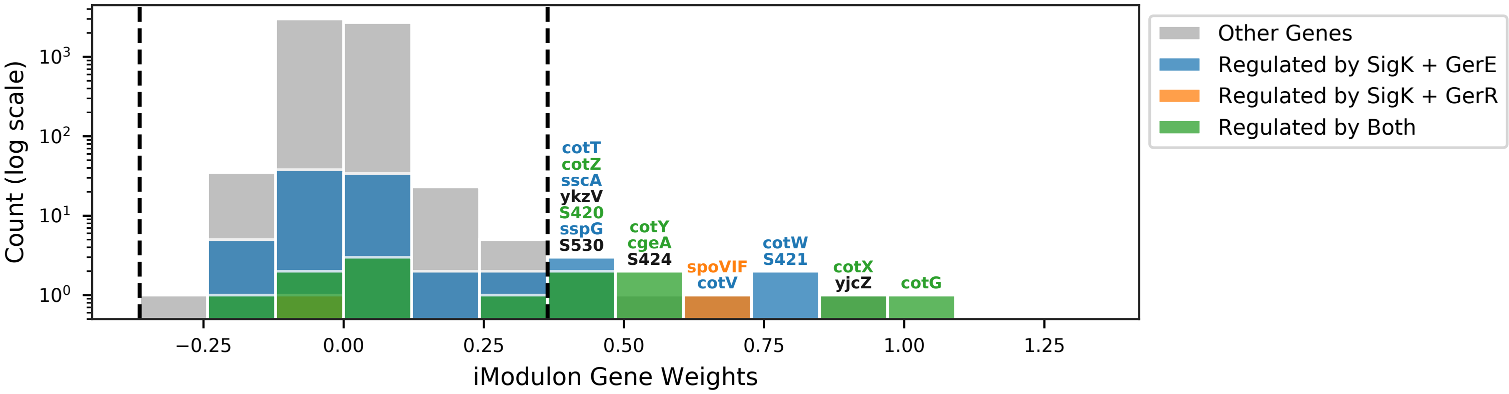

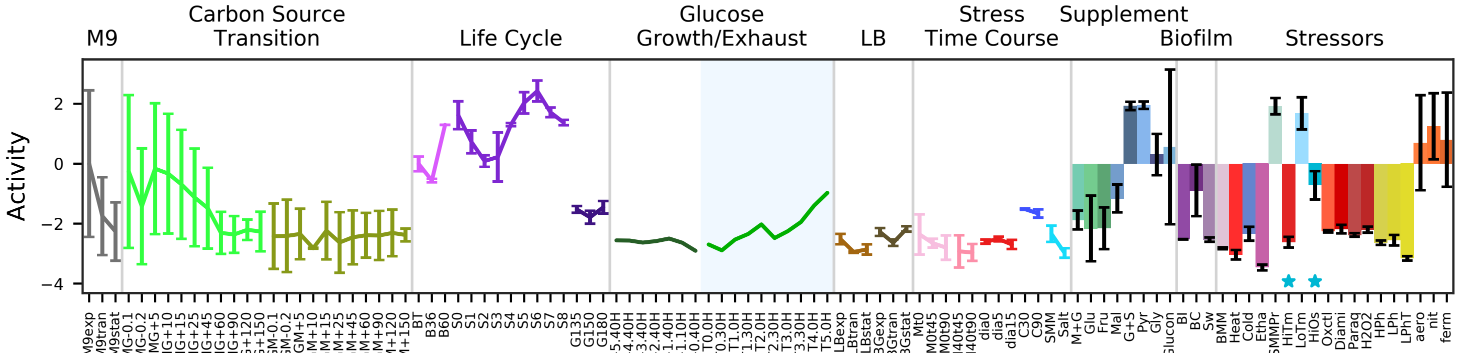

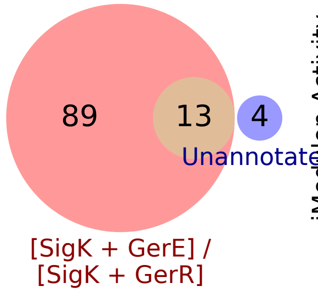

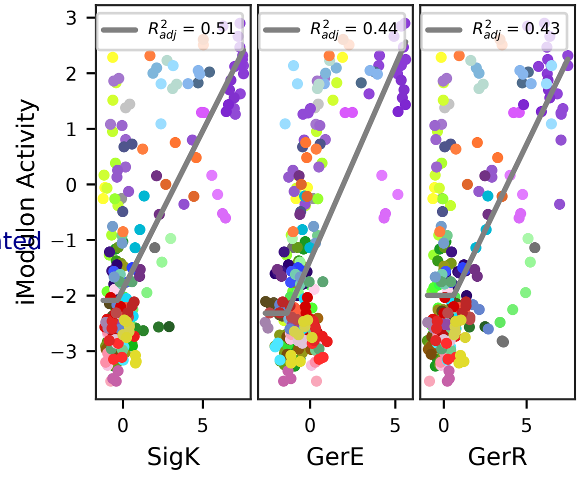

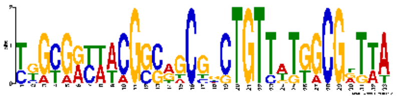

Motif E-value: 6.40e-10
Operons with Upstream Motif: 46%

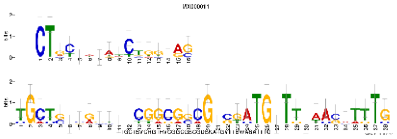

Motif similar to RhlR -
P. aeruginosa (E = 3.08e-03)

55 - Xpf – PBSX Prophage

Biological Function:
Prophage element that induces production of bacteriocin and cell lysis in response to DNA-damaging agents

Well-defined regulon:
Xpf

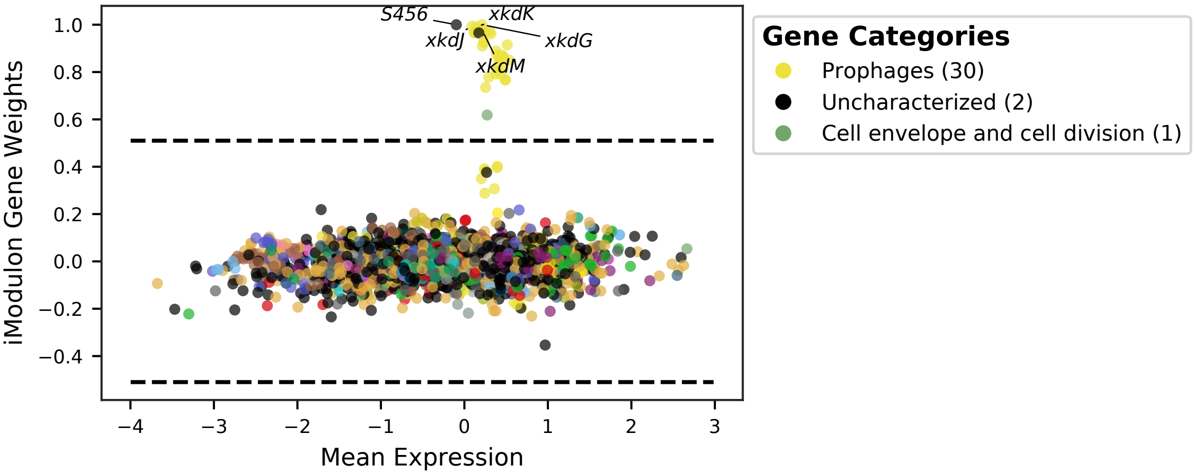

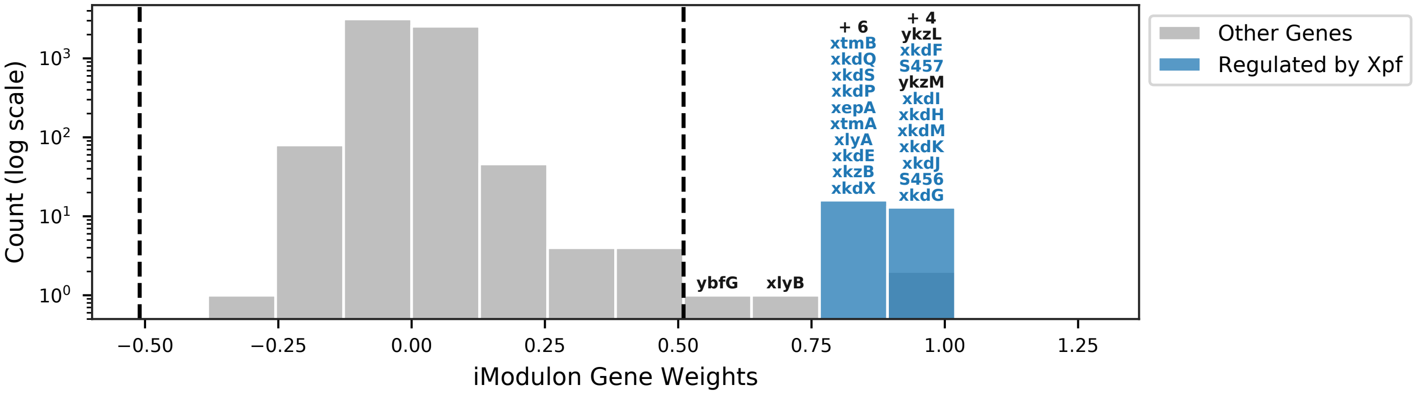

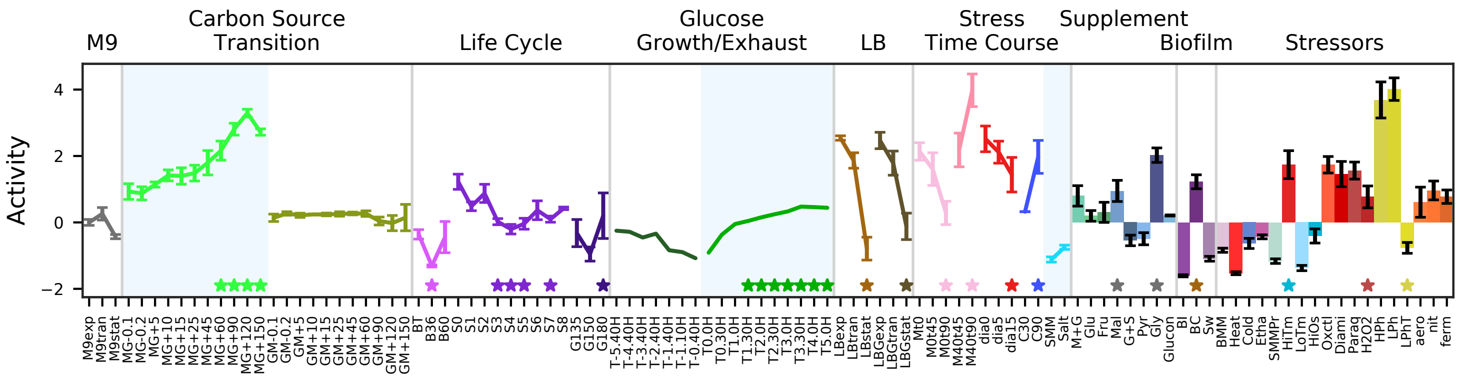

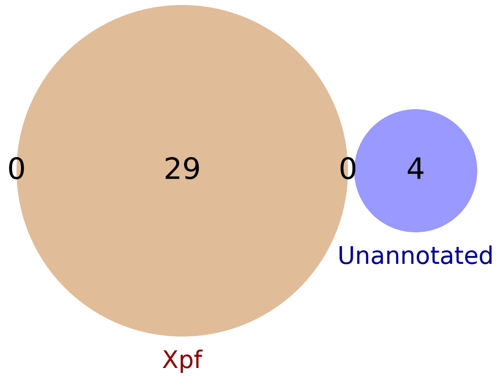

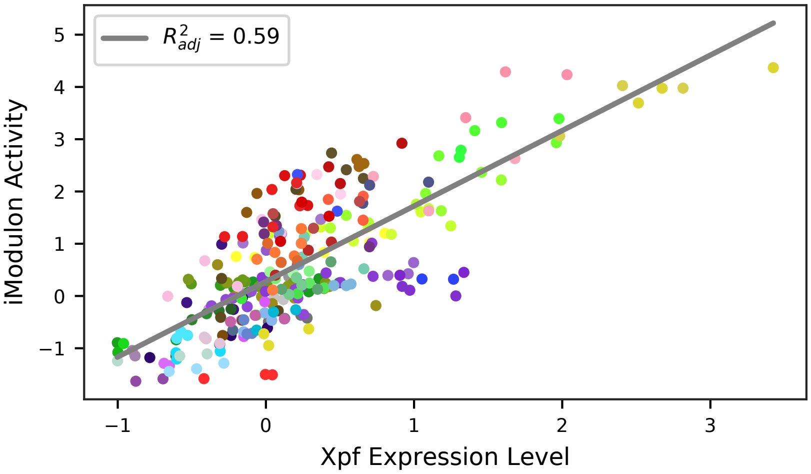

56 - Spβ-1 – SP-β Prophage 1

Biological Function:
Temperature-responsive SP-beta prophage. Response to stressors such as cold and ethanol. Subset of SPbeta Prophage 2.

No known regulator

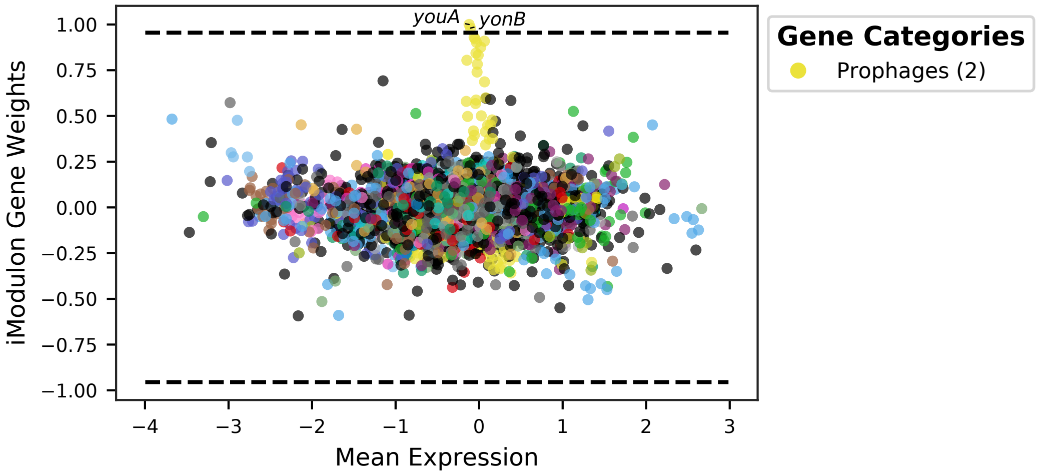

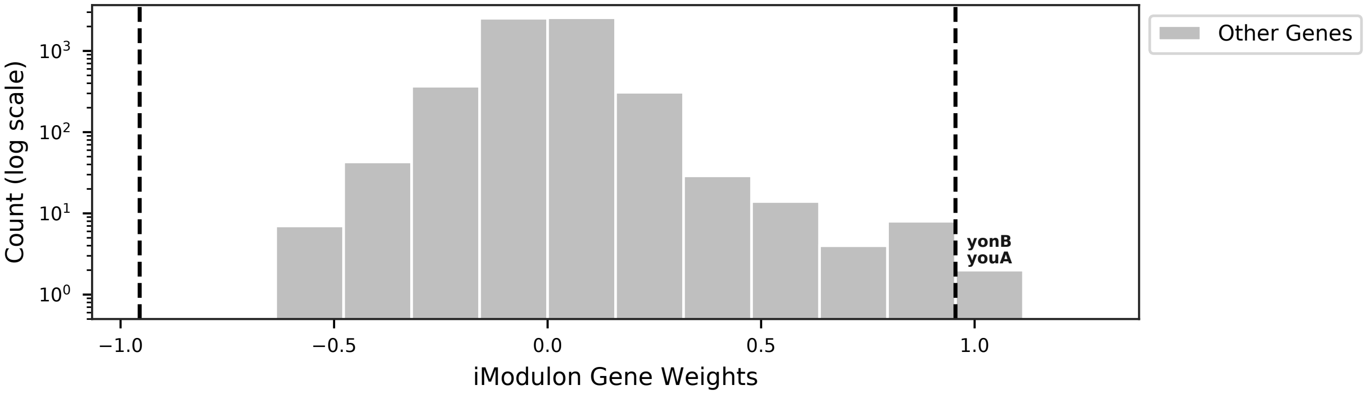

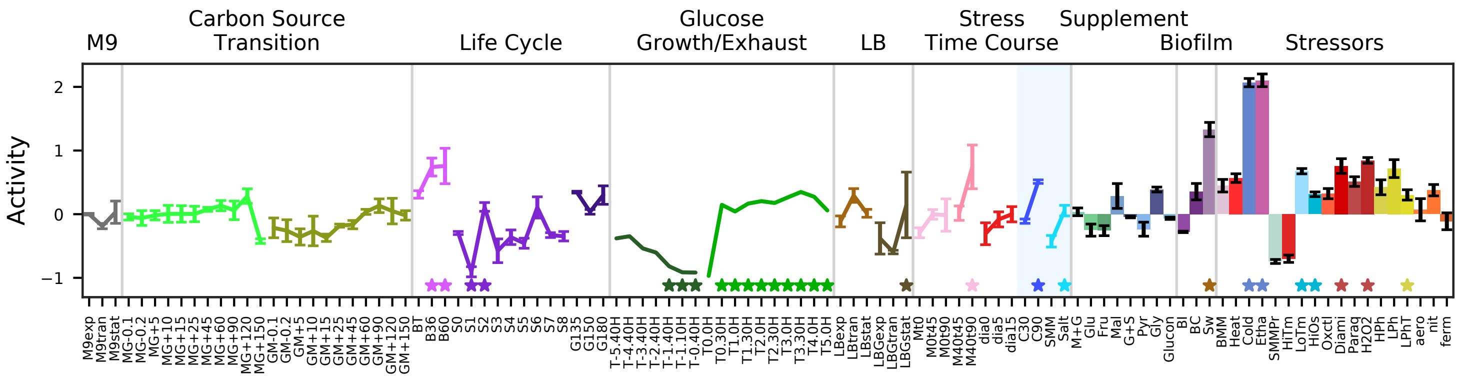

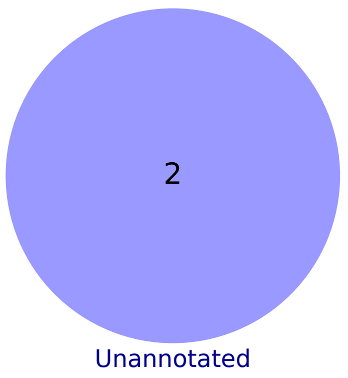

57 - Spβ-2 – SP-β Prophage 2

Biological Function:
Temperature-responsive SP-beta prophage. Prophage, unknown, and putative metabolic genes. Response to mitomycin.

Contains unknown genes and known regulon:
CsoR + SigA + SigK + SigE

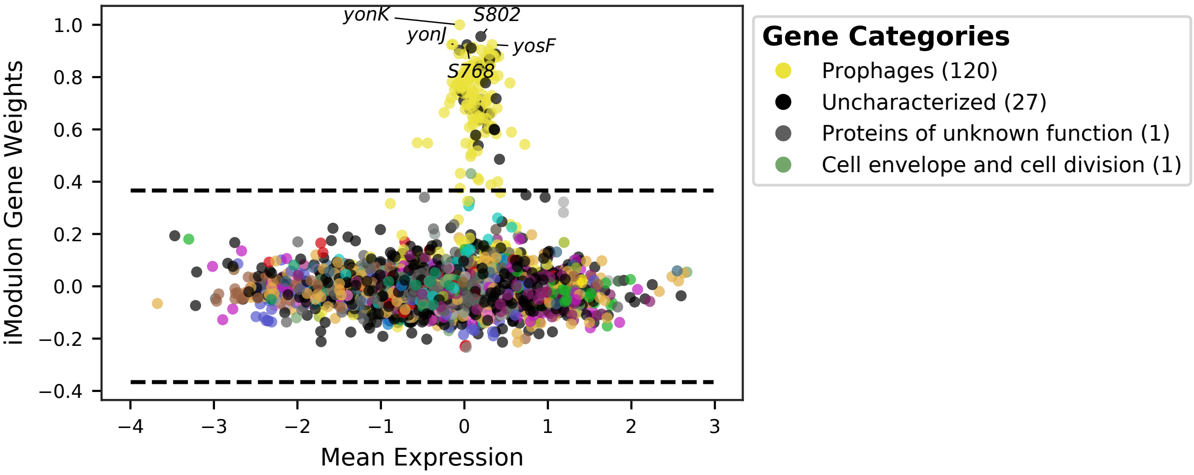

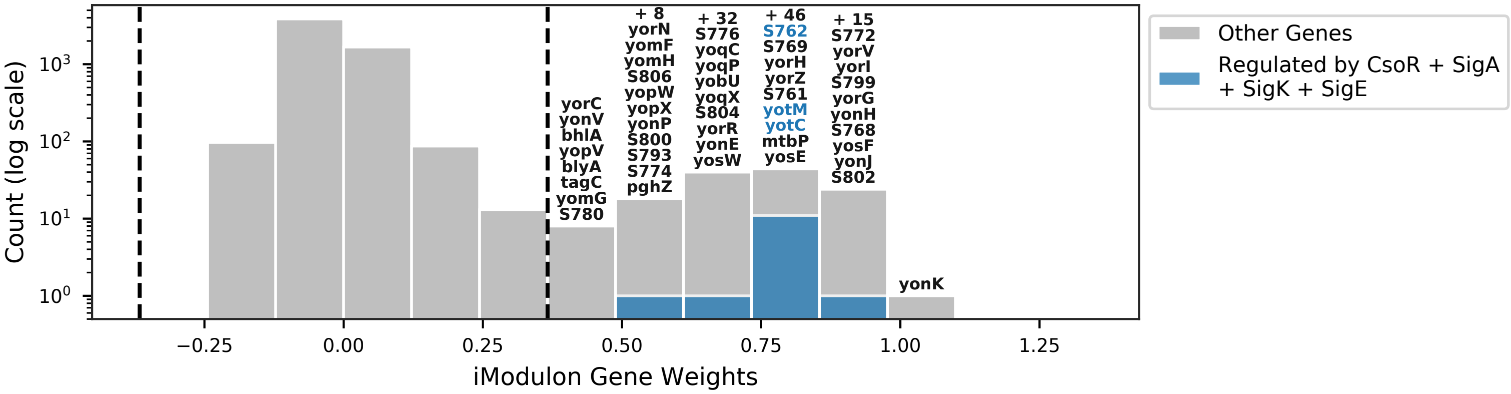

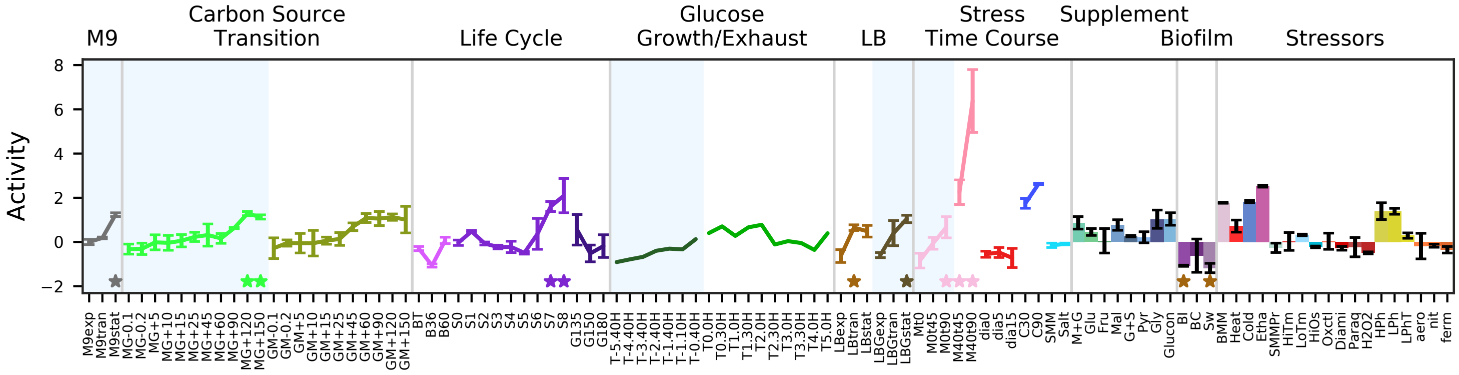

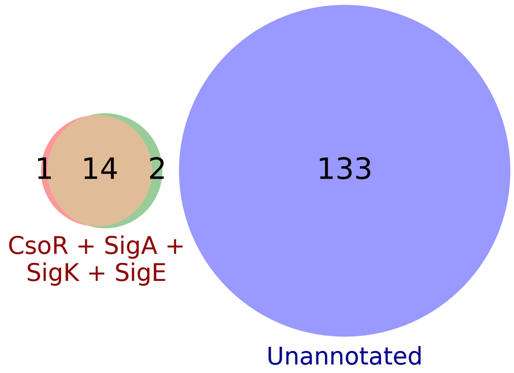

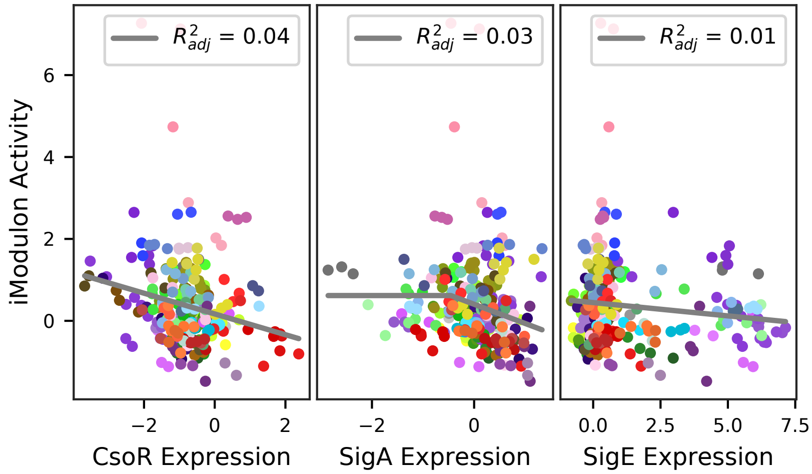

58 - ImmR – ICEBs1

Biological Function:
Mobile genetic element that participates in horizontal gene transfer

Well-defined regulon:
ImmR

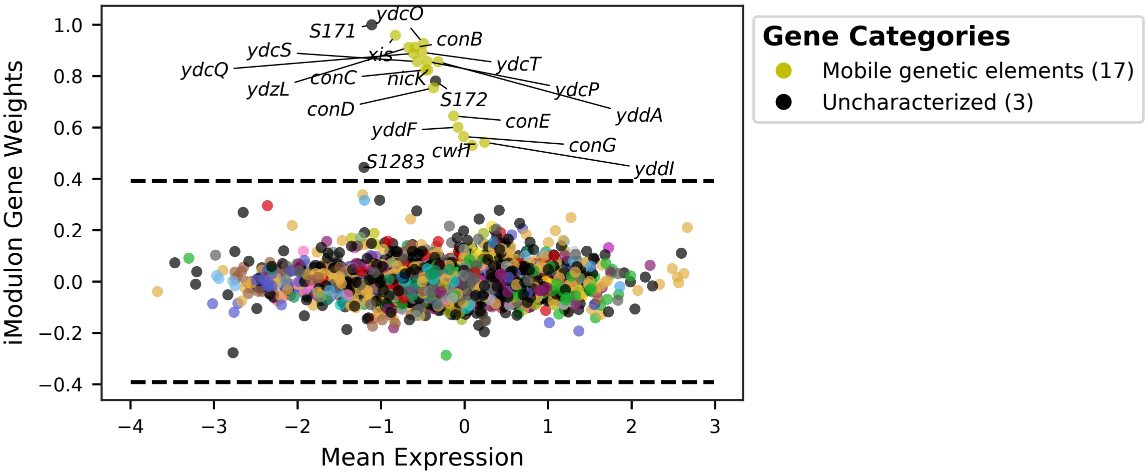

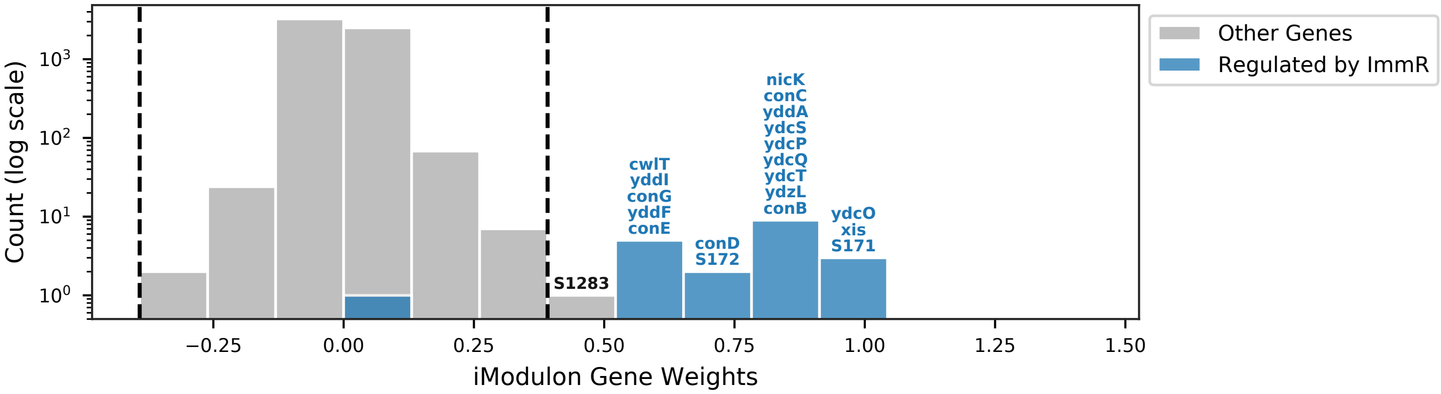

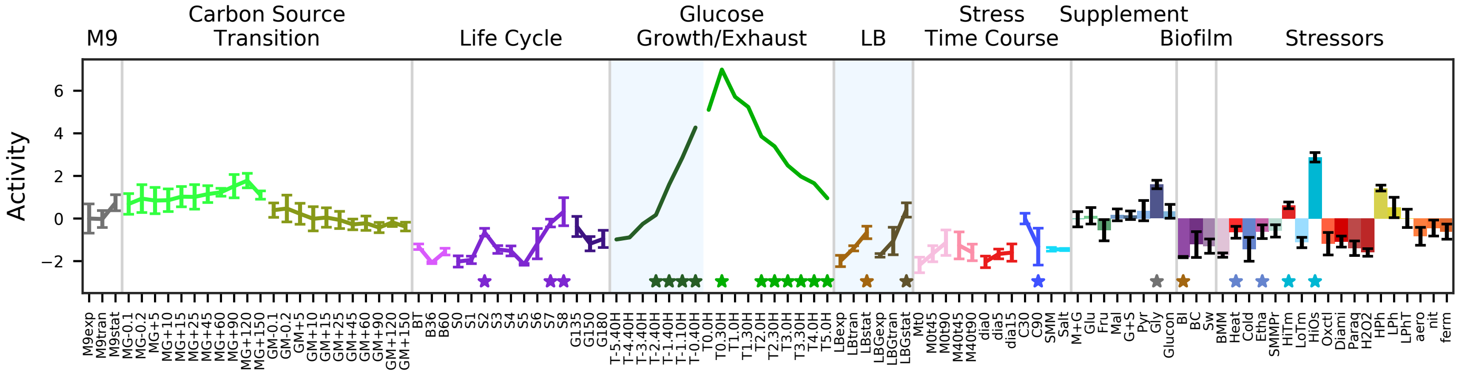

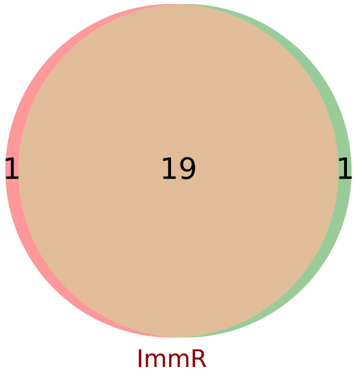

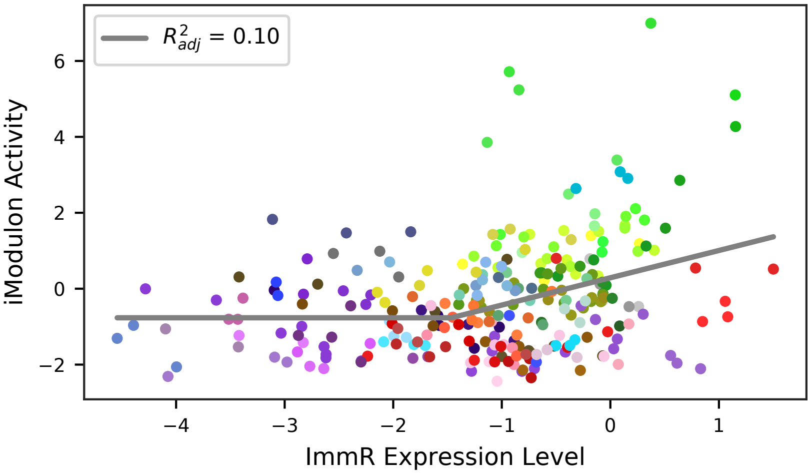

59 - SknR – Skin Element

Biological Function:
Prophage element that disrupts the SigK gene but also plays roles in preventing early germination and regulating proliferation. Unknown and toxin resistance genes.

Enriched for known regulon:
SknR

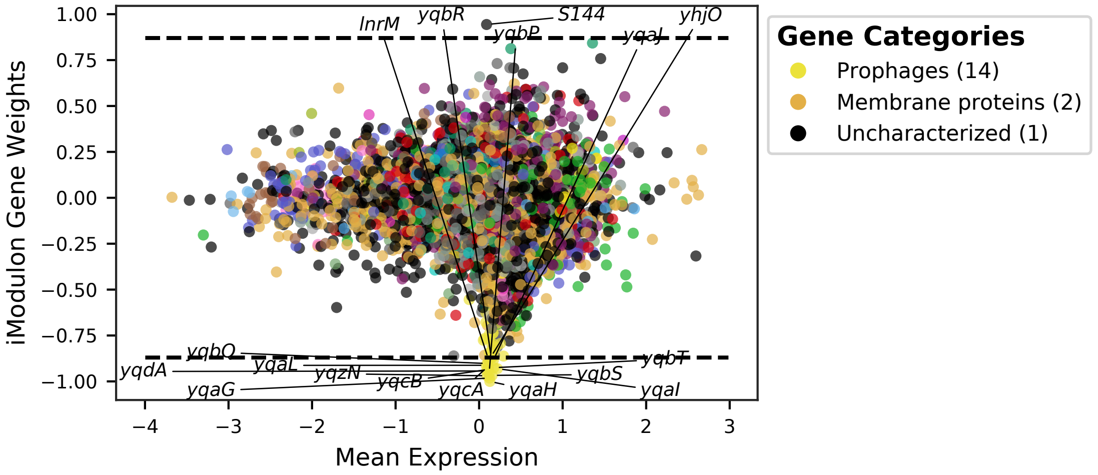

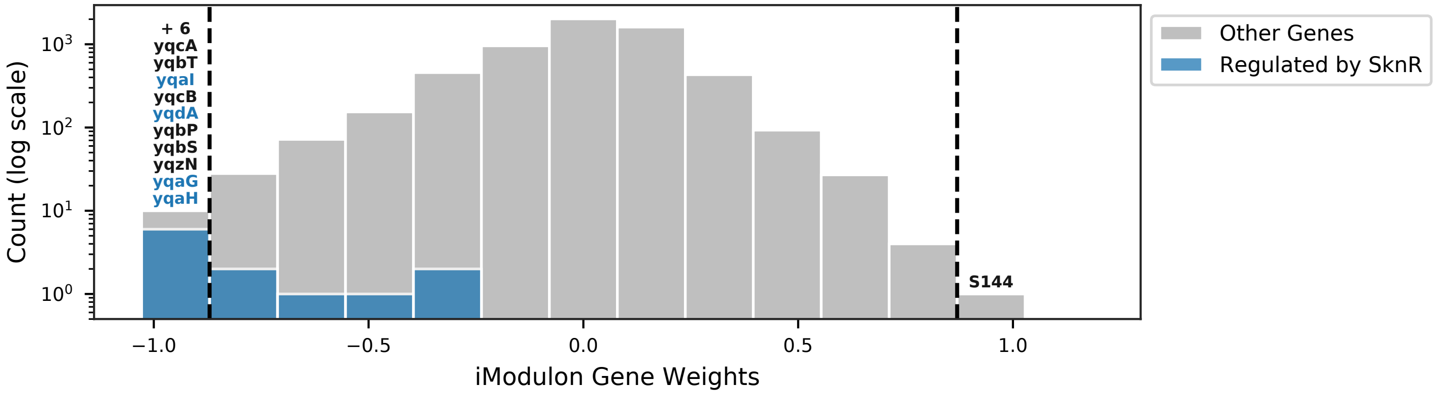

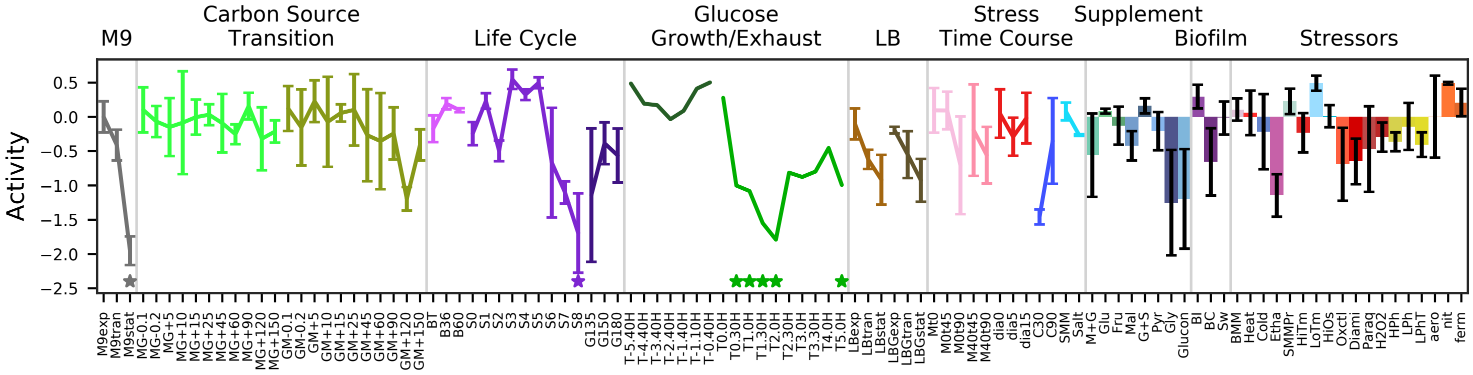

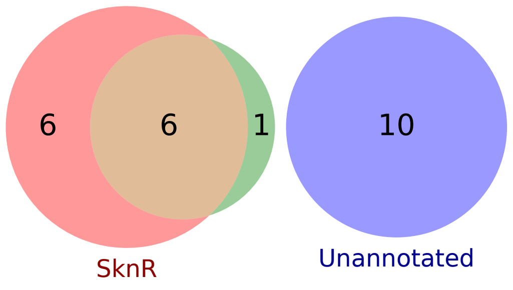

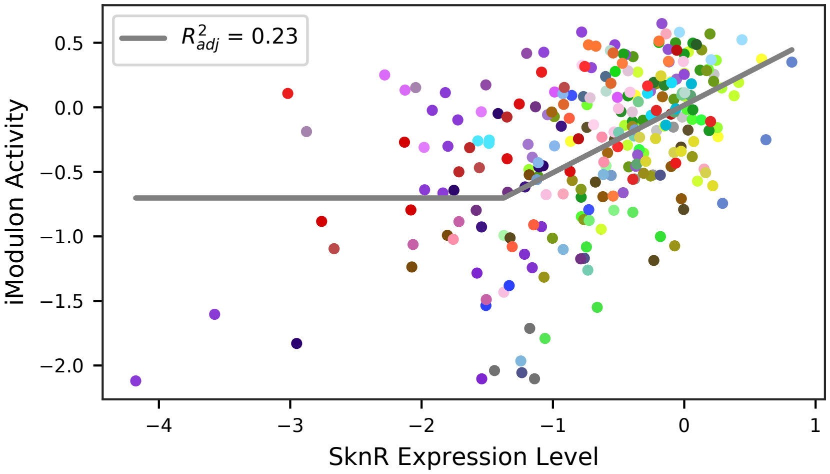

60 - IolR / MelR – Inositol, Melibiose, and Acetoin

Biological Function:
Uptake and utilization of inositol, melibiose/raffinose, and acetoin

Well-defined regulon:
[IolR] / [MelR] / [AcoR]

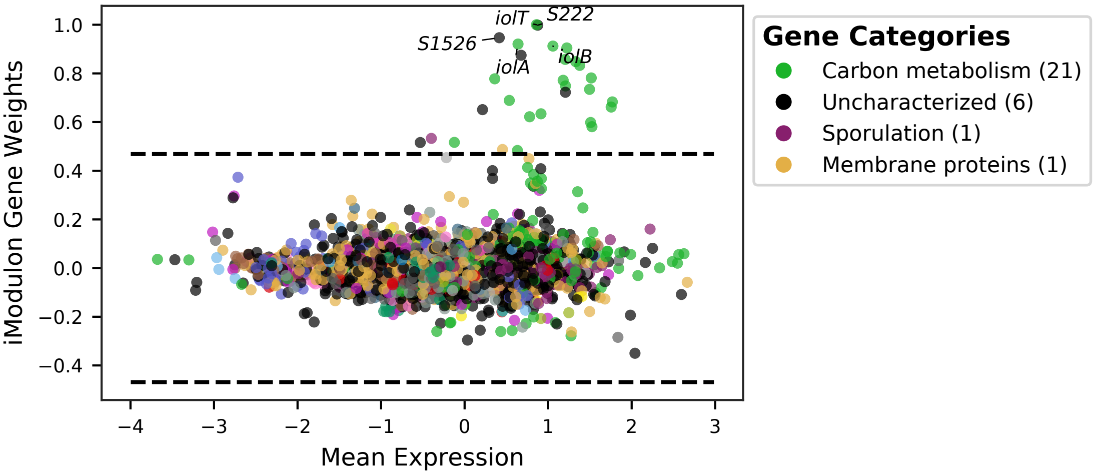

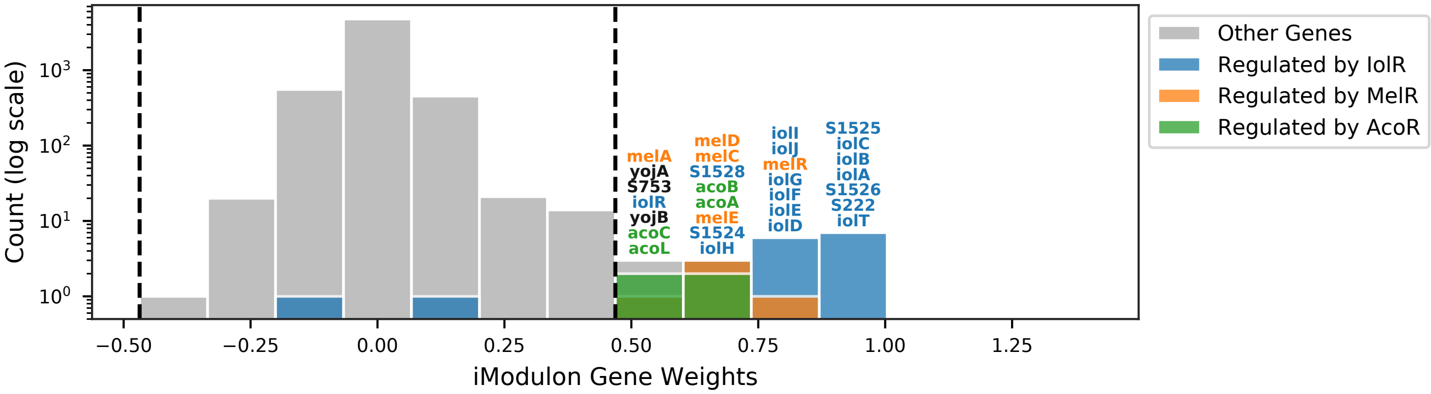

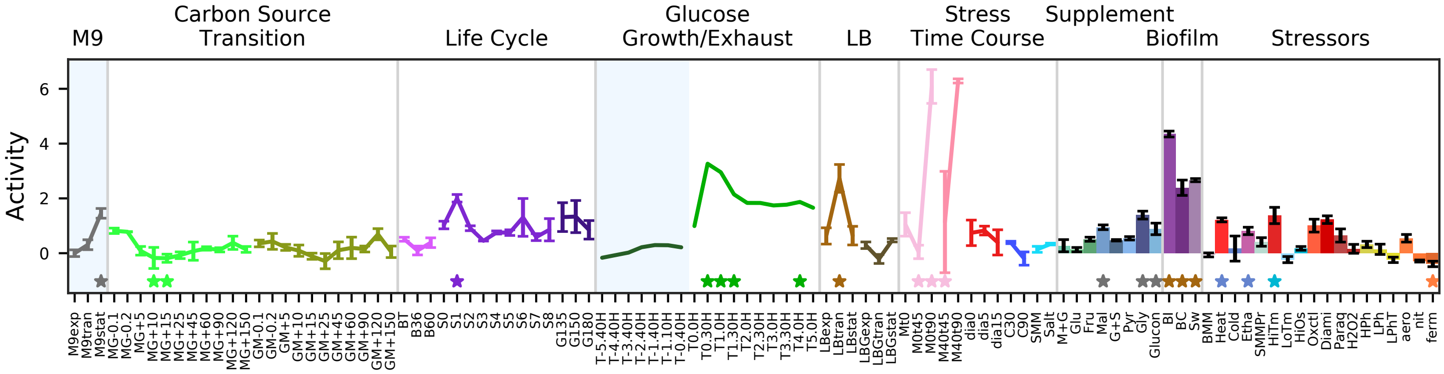

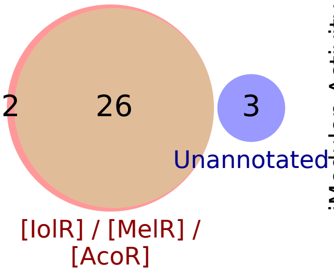

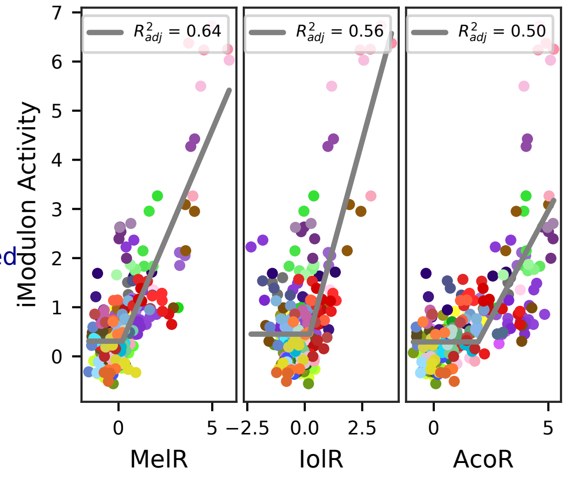

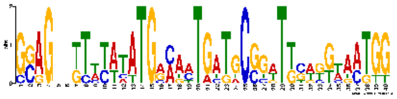

Motif E-value: 1.80e-06
Operons with Upstream Motif: 67%

61 - NadR / BirA – NAD and Biotin

Biological Function:
Biosynthesis of NAD and biotin, plus safA & coxA sporulation proteins and repression of manganese uptake

Well-defined regulon:
[NadR] / [BirA]

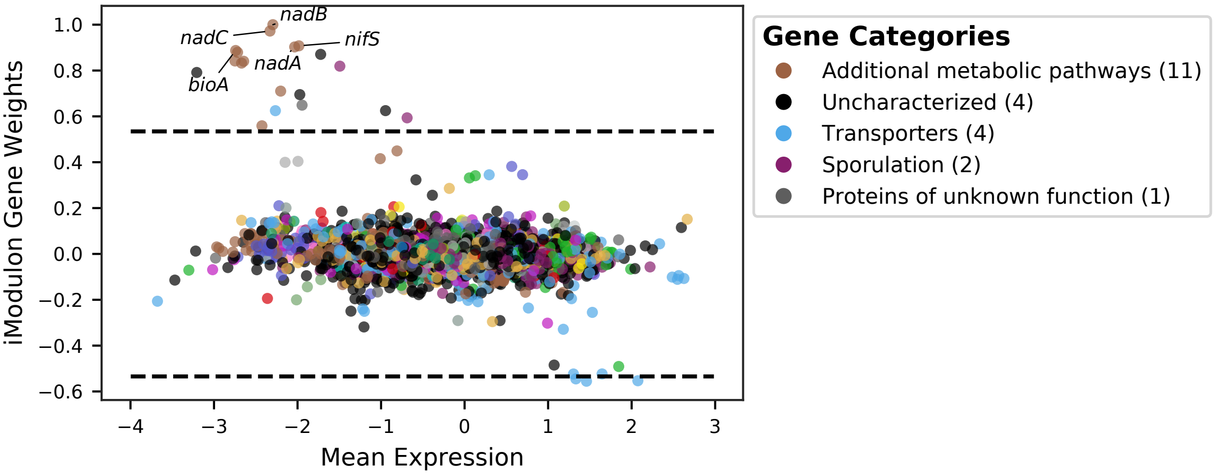

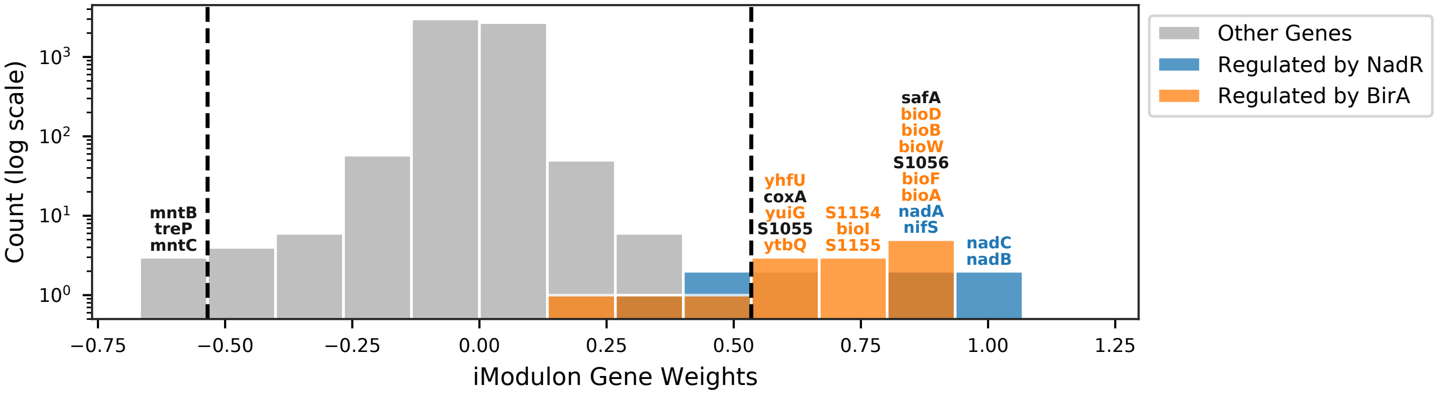

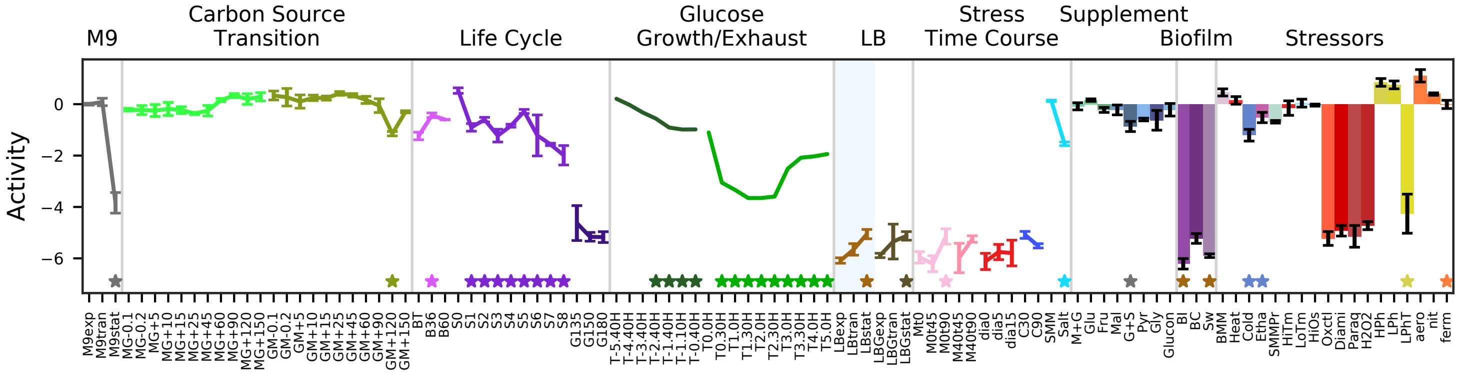

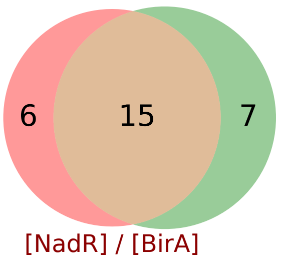

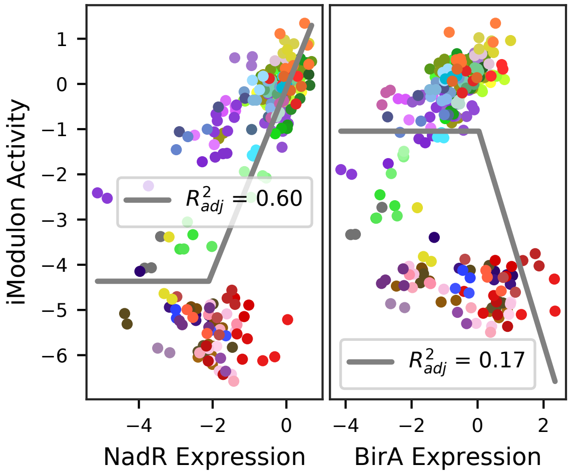

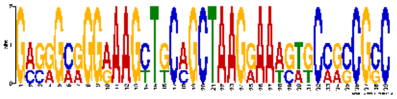

Motif E-value: 9.20e-04
Operons with Upstream Motif: 62%

62 - MtlR / AnsR – Mannitol, Asparagine, and Aspartate

Biological Function:
Uptake and utilization of mannitol, degradation of asparagine and aspartate

Well-defined regulon:
[MtlR] / [AnsR + SigA]

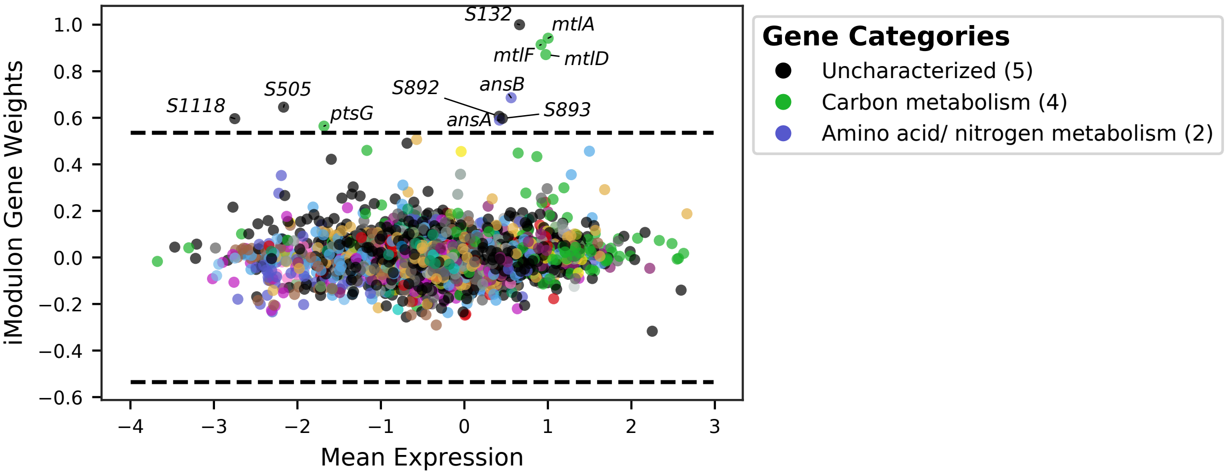

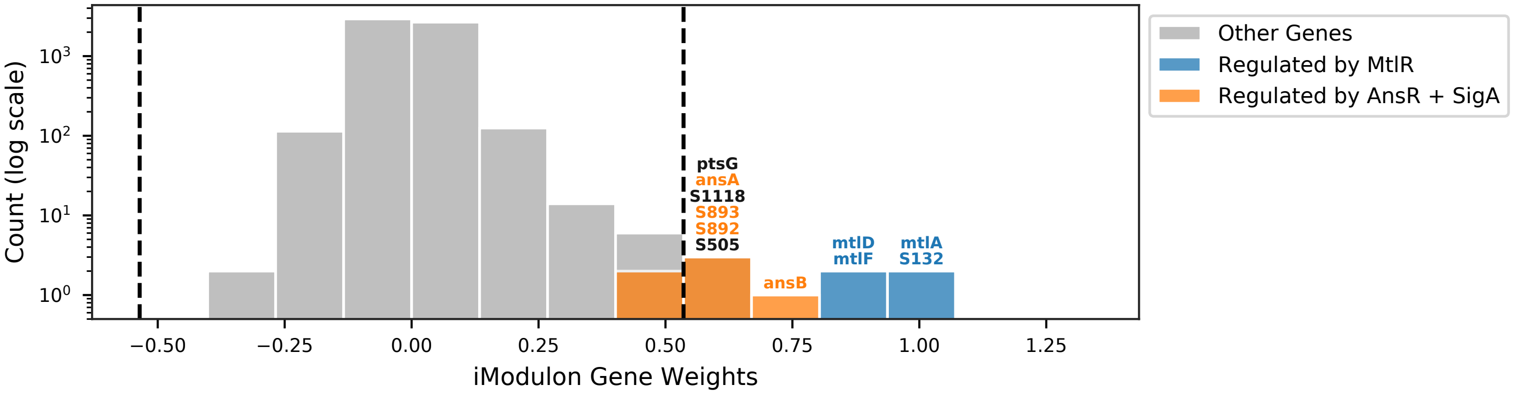

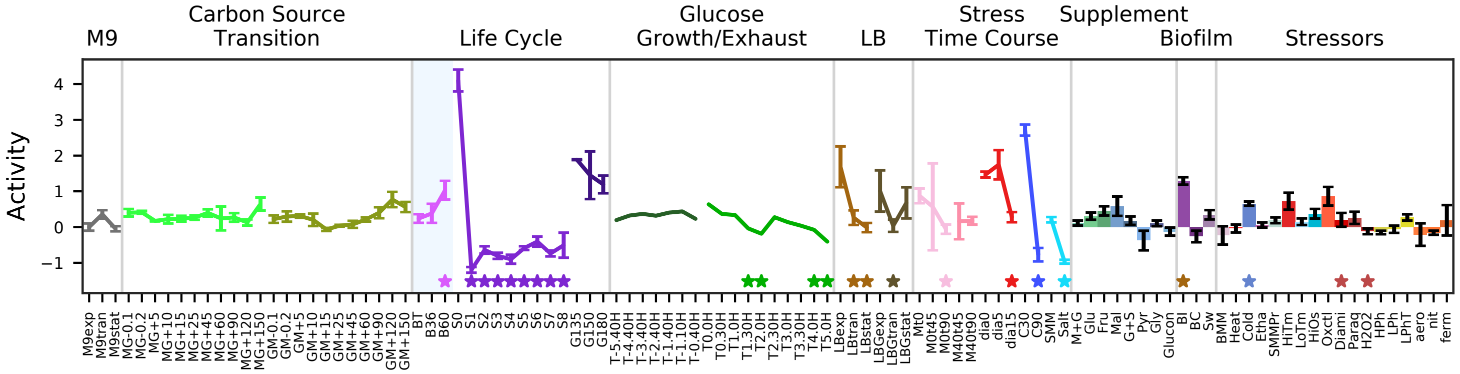

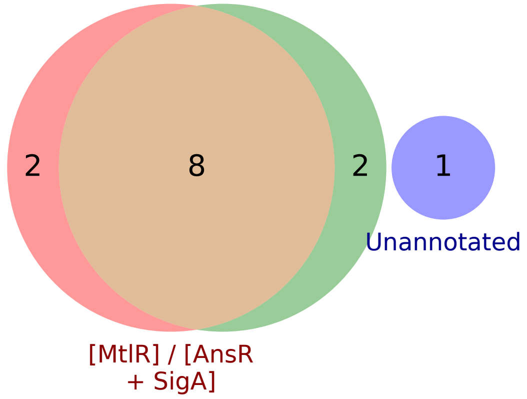

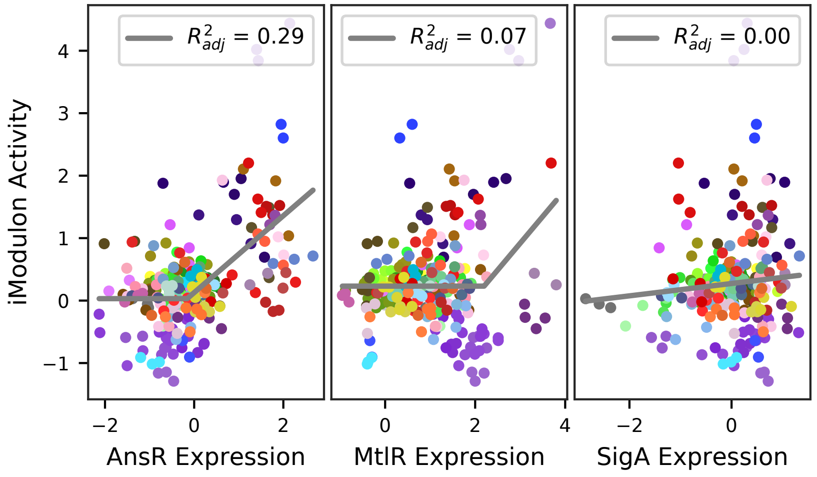

63 - TnrA / PucR – Nitrogen Limitation

Biological Function:
Ammonium uptake, utilization of nitrate and xanthine. Suggests nitrate limitation in the BT condition

Subset of known regulon:
[SigA + TnrA] / [SigA + PucR]

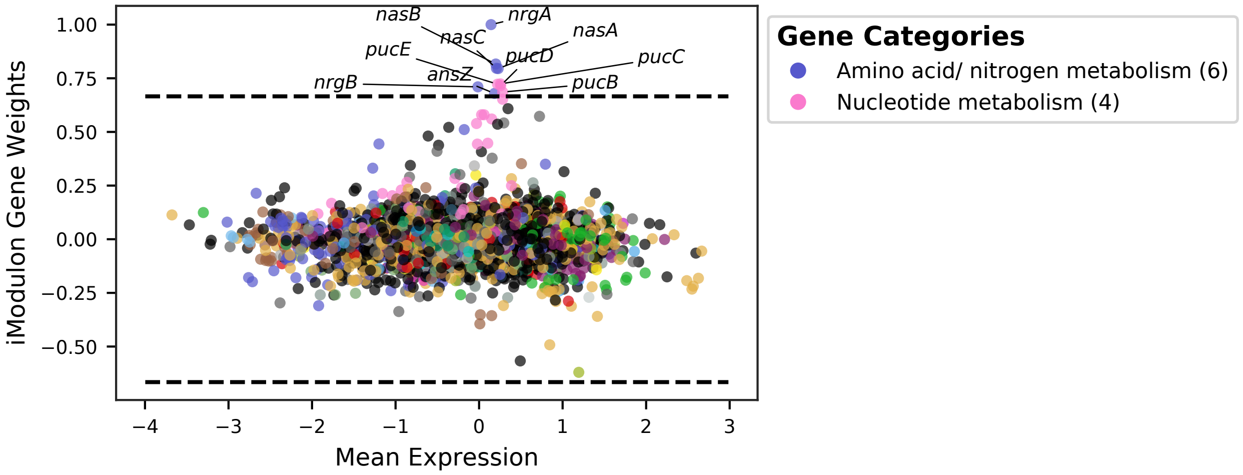

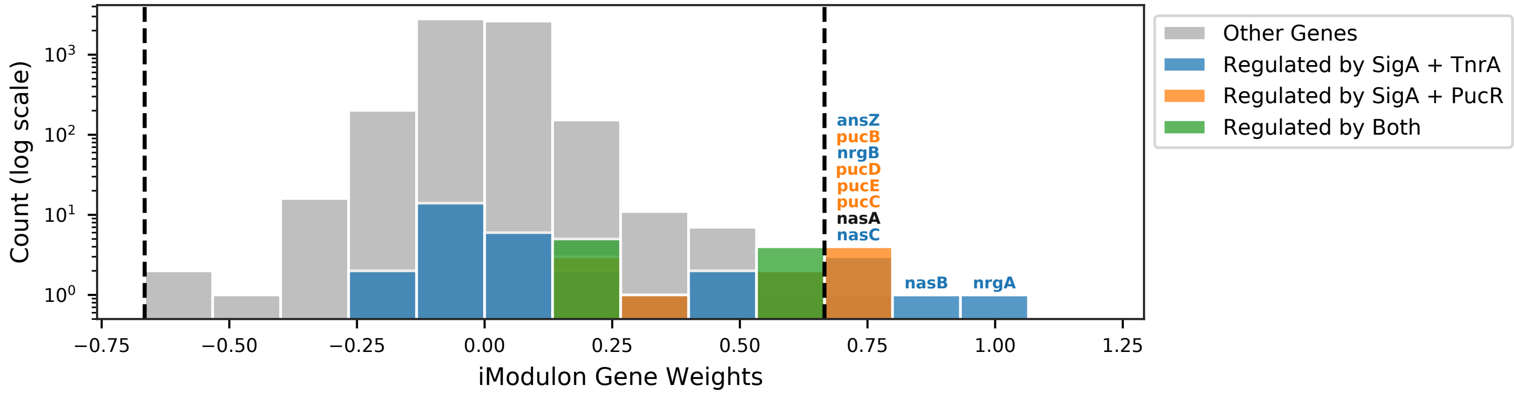

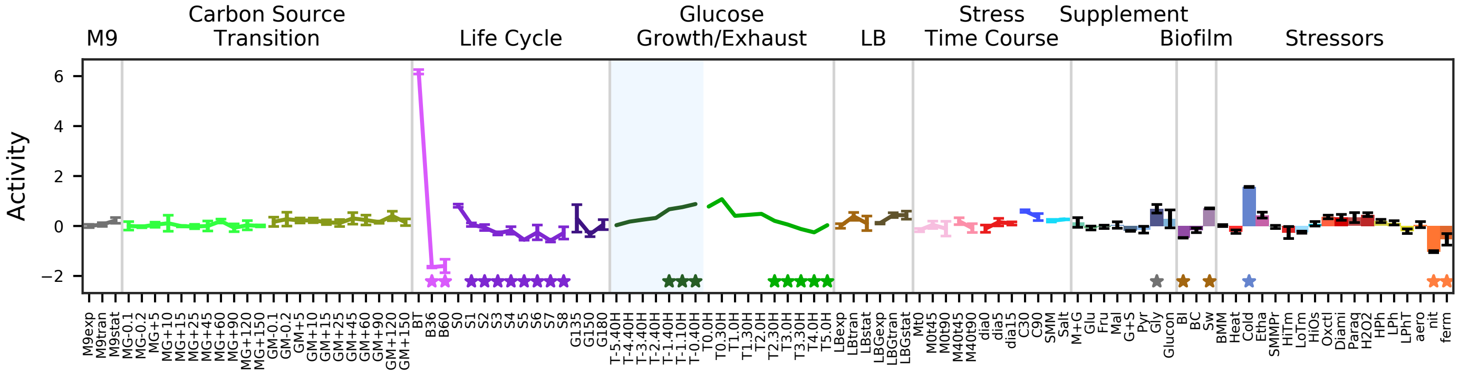

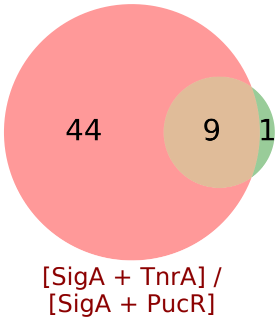

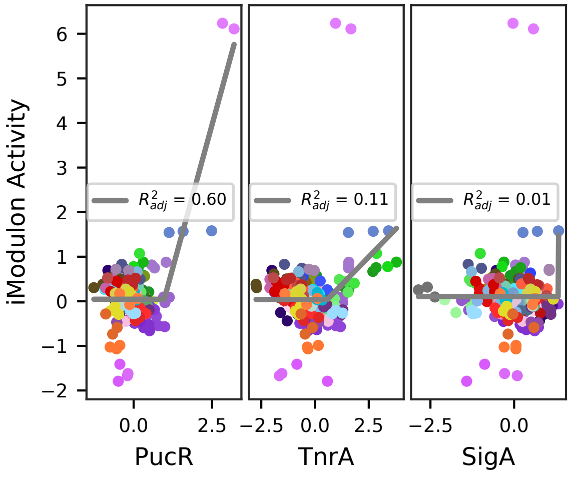

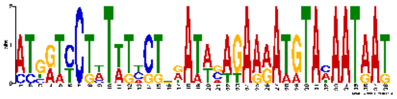

Motif E-value: 5.60e-07
Operons with Upstream Motif: 100%

64 - CymR / NsrR – Sulfur and Nitrate Respiration

Biological Function:
Import and utilization of sulfonate and cystine for cysteine production. Nitrite reduction

Subset of known regulon:
[SigA + CymR] / [ResD + SigA + NsrR]

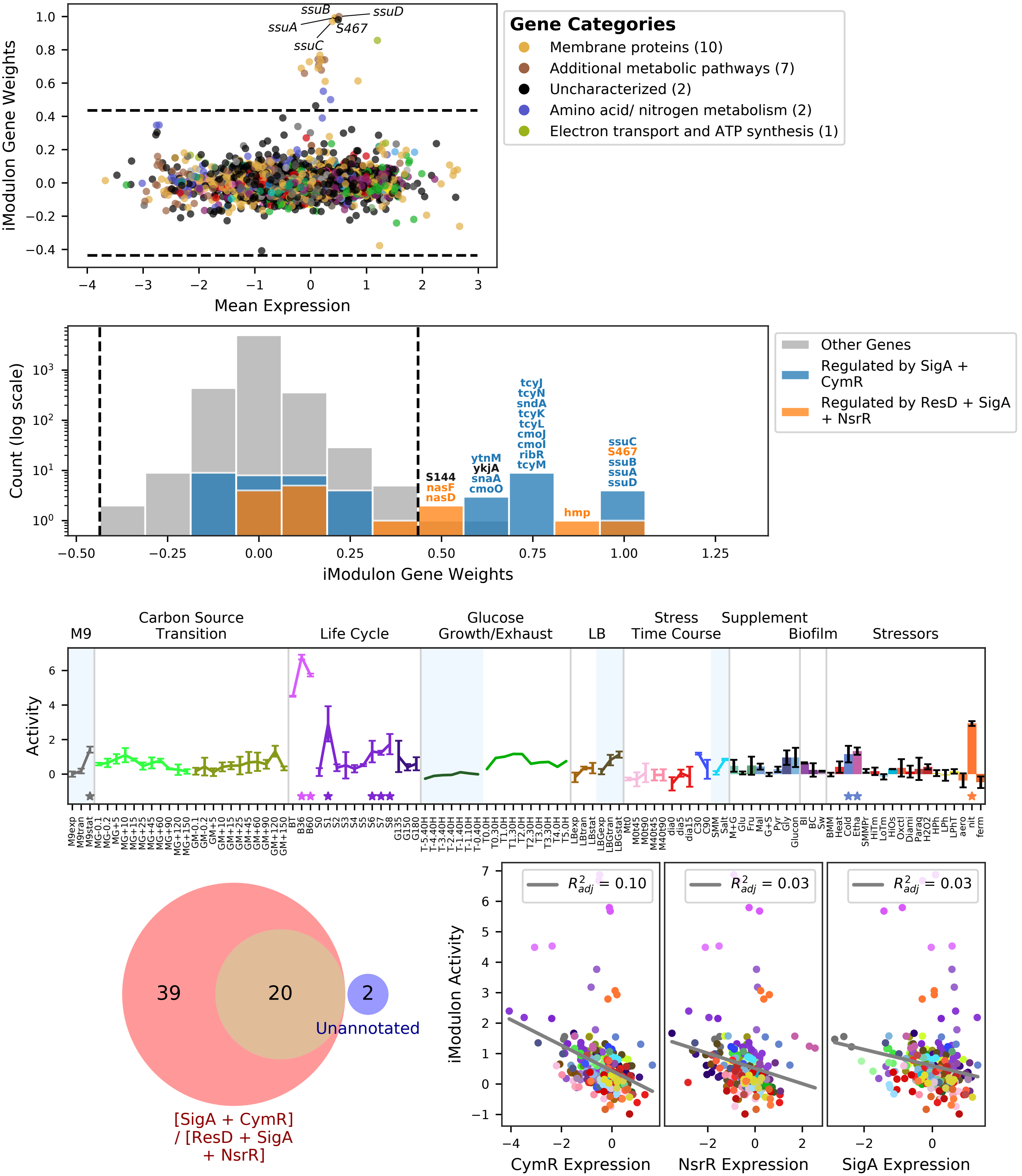

65 - ArsR+ – Diamide Stress

Biological Function:
Detoxification in response to: oxidative protein damage, arsenic, catechol, copper, toxic quinones

Enriched for known regulon:
[ArsR] / [YodB] / [SigA + CtsR] / [SigA + CsoR]

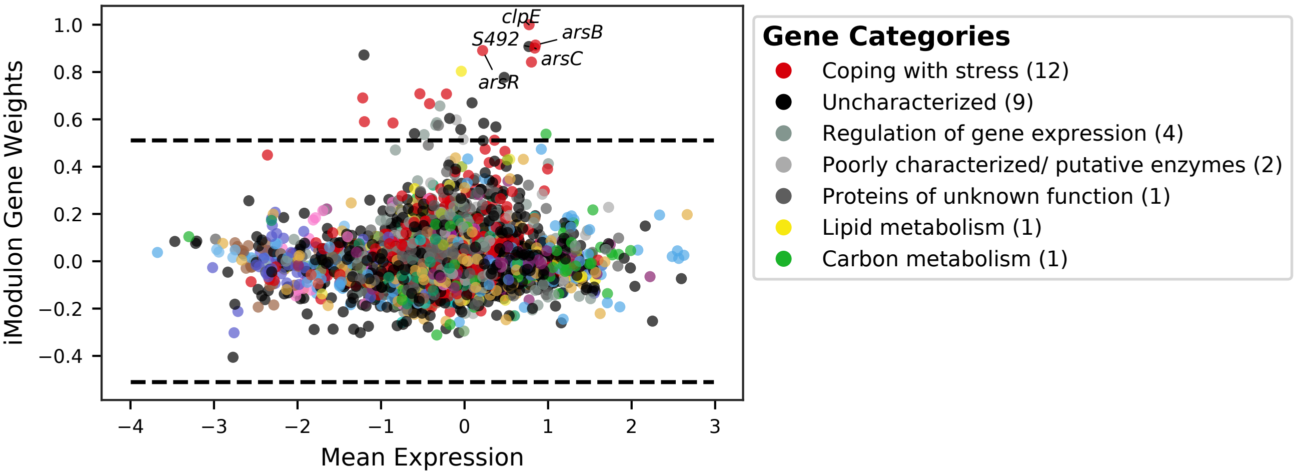

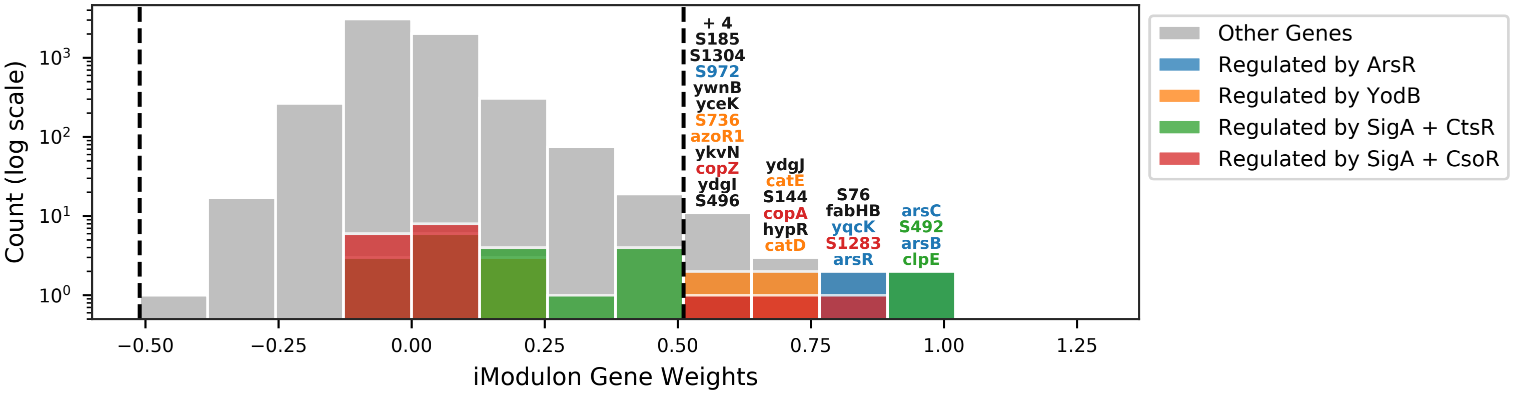

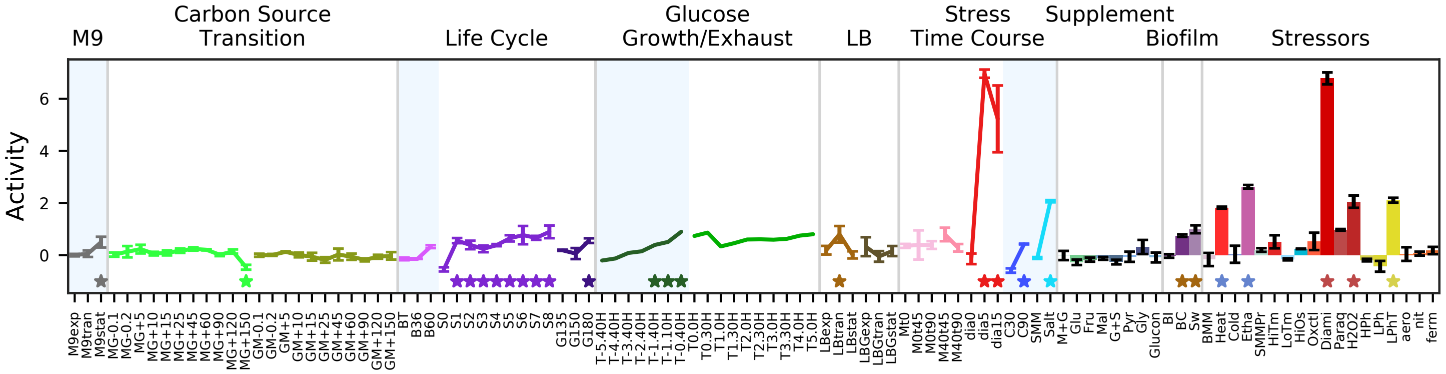

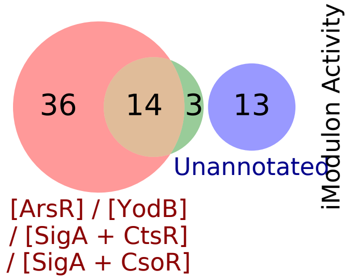

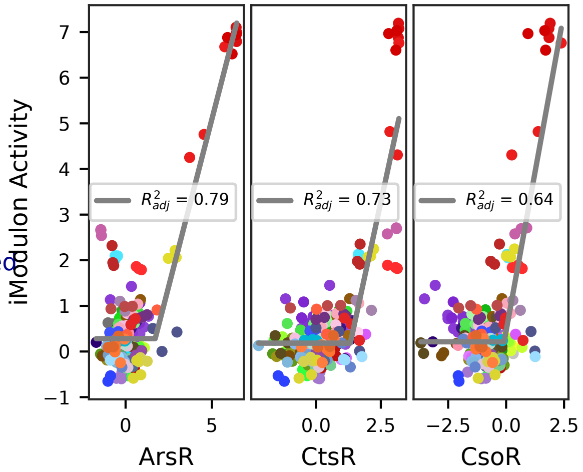

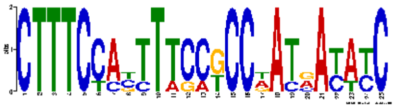

Motif E-value: 1.90e-05
Operons with Upstream Motif: 31%

66 - Uncharacterized 1

Biological Function:
Contains a SigE/SigG inhibitor, spore coat proteins, translational proteins, oxidative stress response, many RNAs

No known regulator

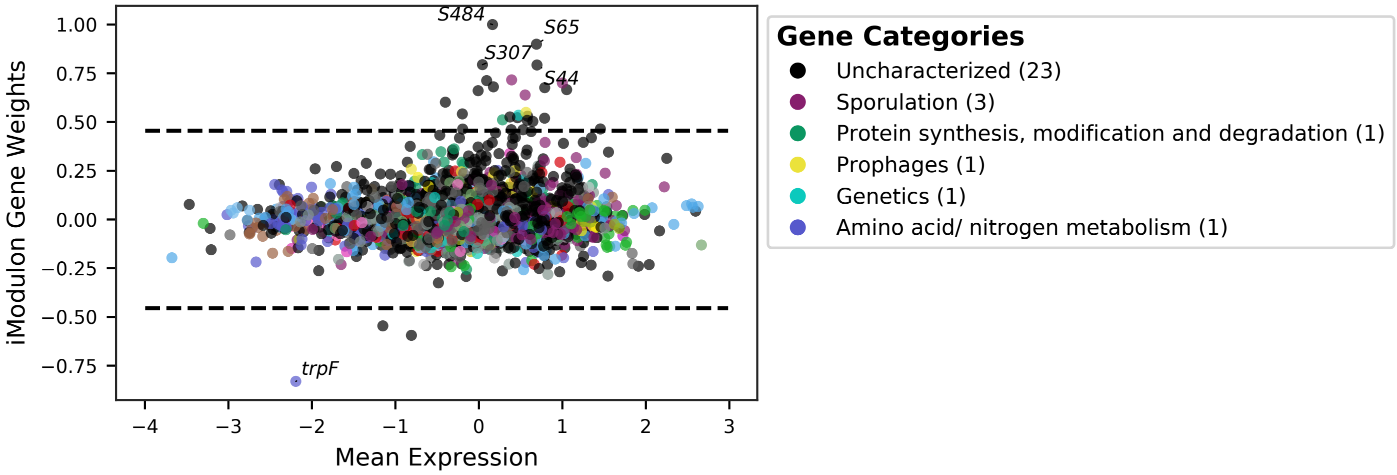

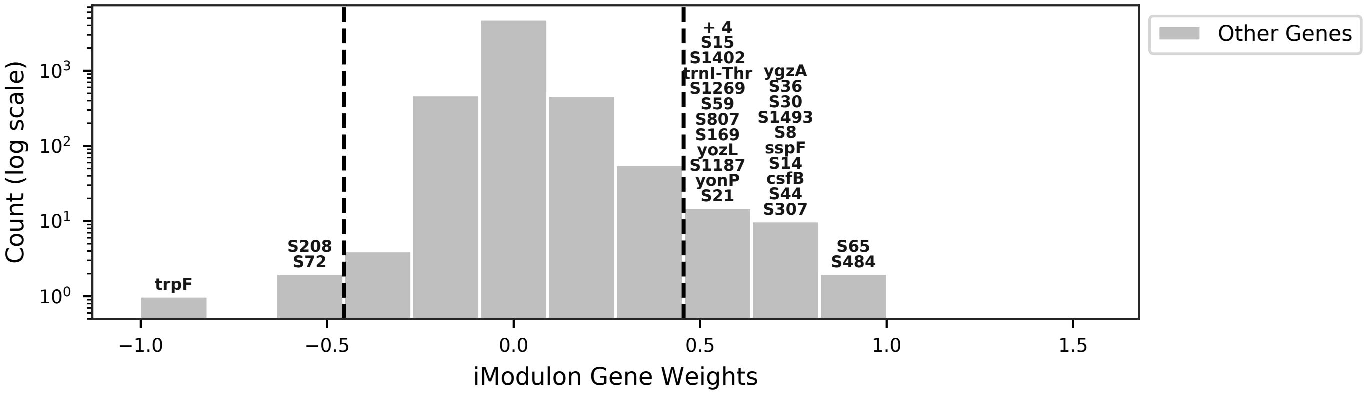

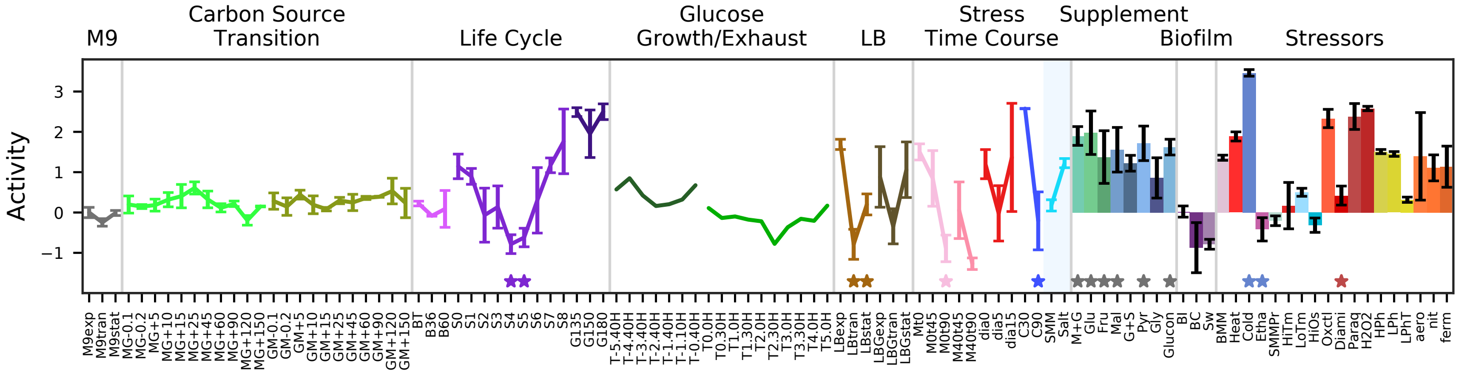

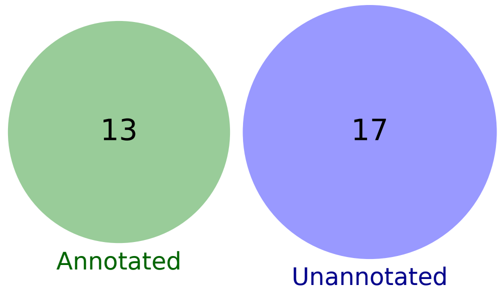

67 - Uncharacterized 2

Biological Function:
Contains cold shock, sporulation onset, toxin, amino acid homeostasis, translataional, other proteins, many RNAs

No known regulator

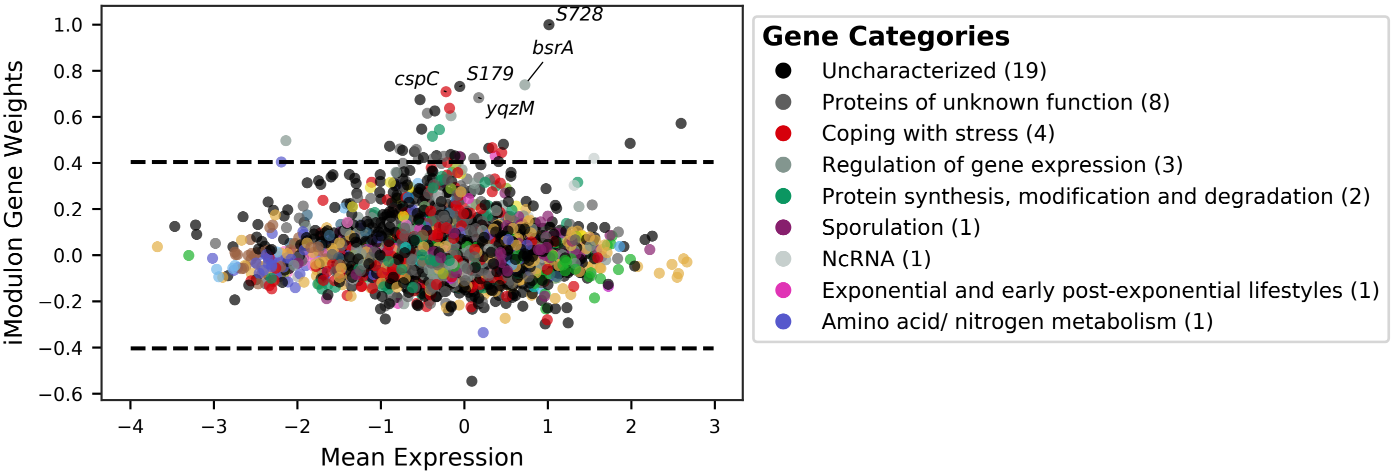

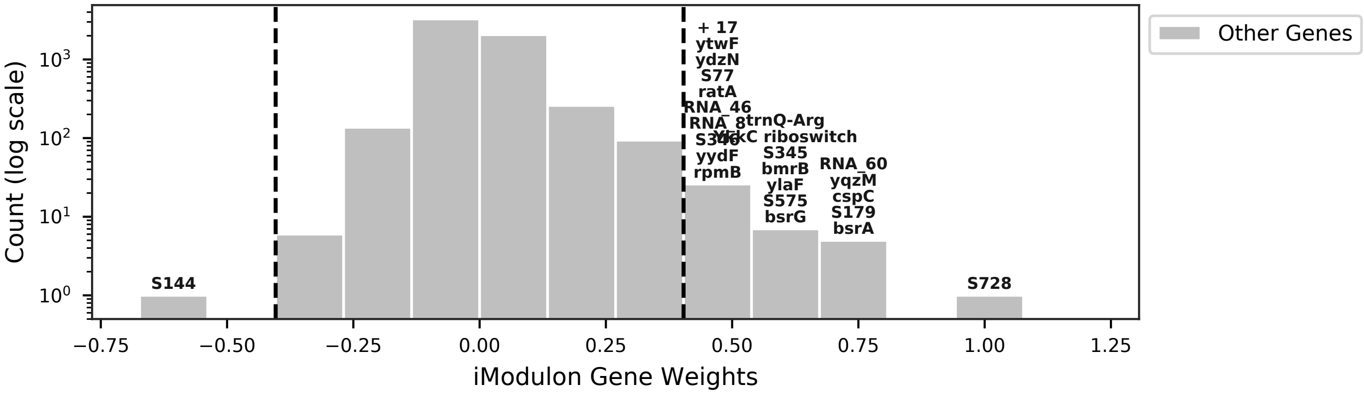

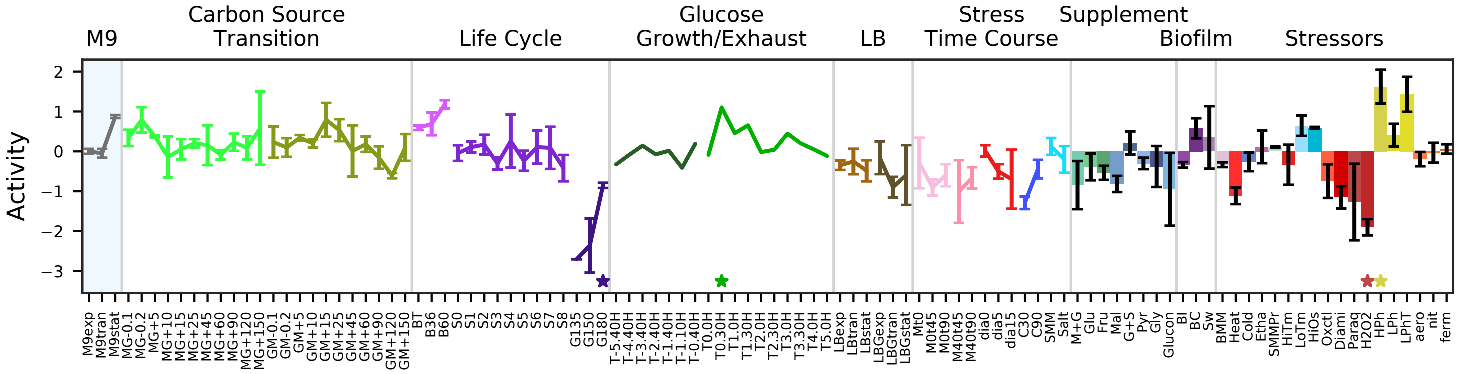

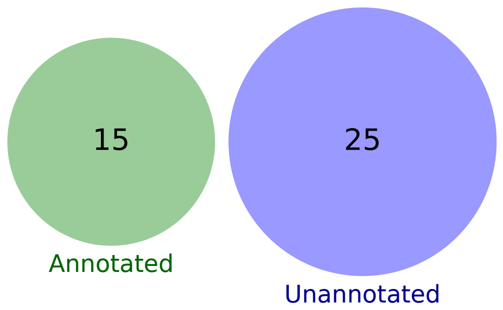

68 - Uncharacterized 3

Biological Function:
Contains many poorly characterized RNAs with noisy activity but clear time correlations, especially in sporulation. May be indicative of overall decreased translational activity

No known regulator

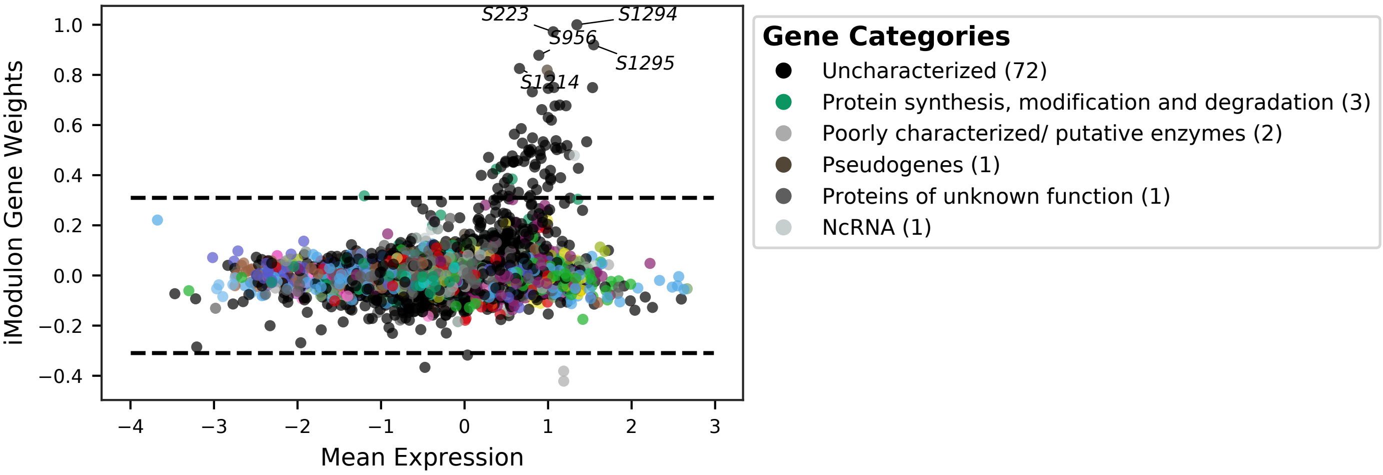

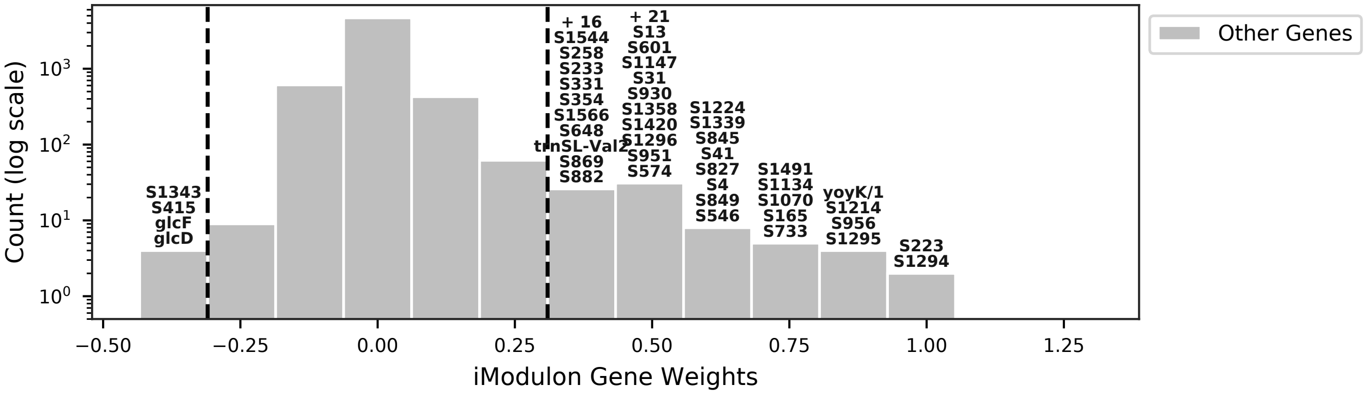

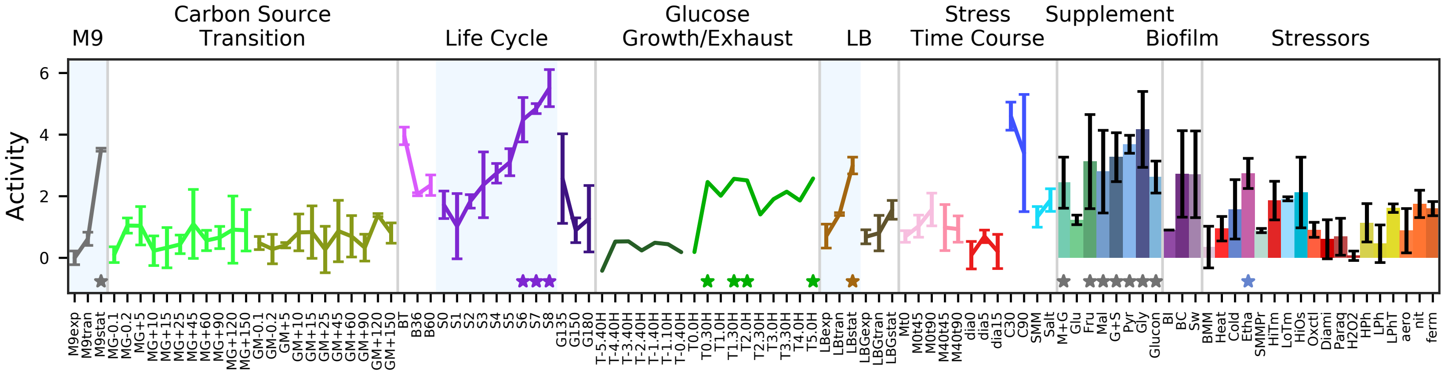

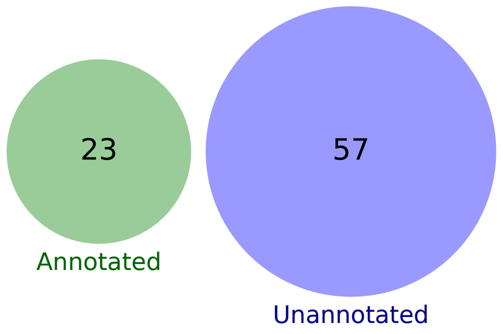

69 - Uncharacterized 4

Biological Function:
Contains many poorly characterized RNAs with noisy activity

No known regulator

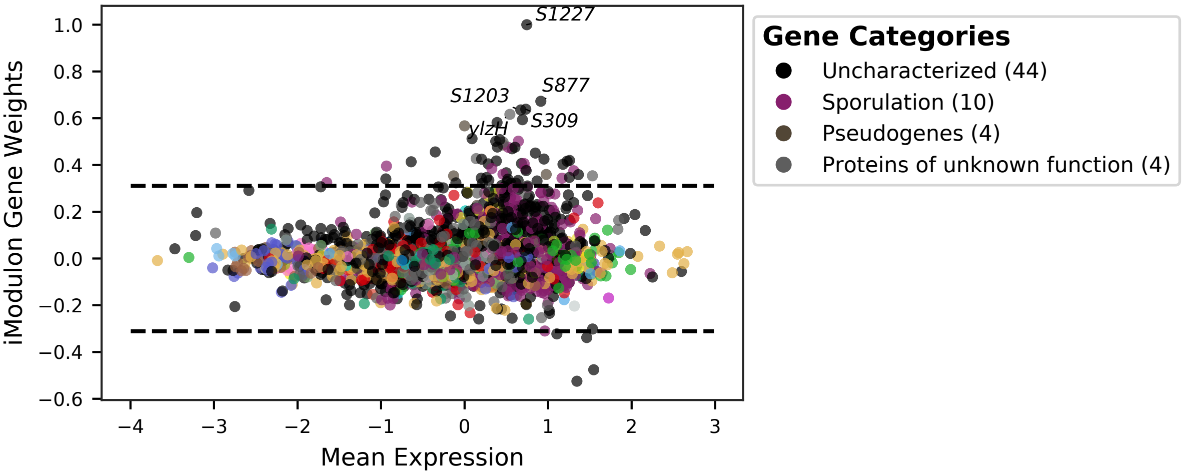

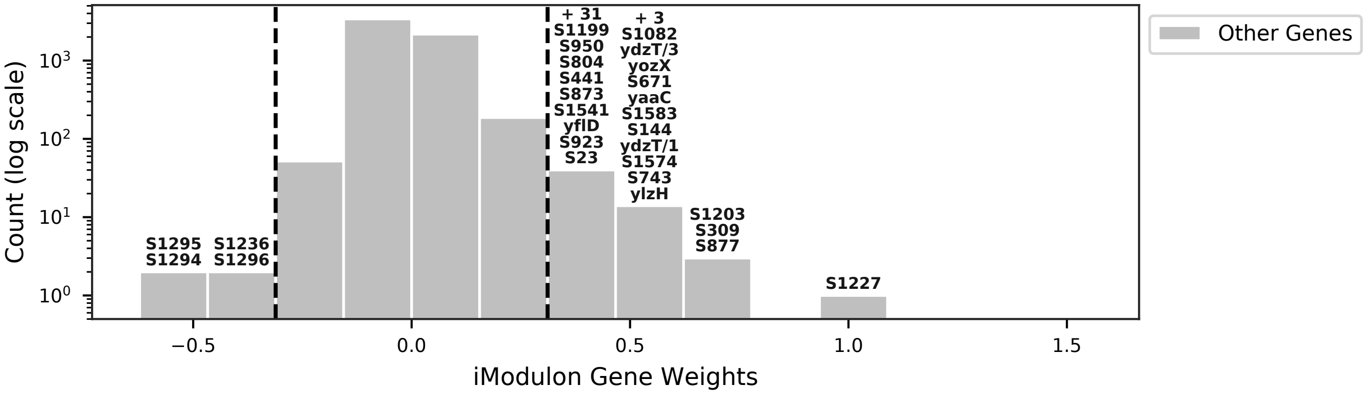

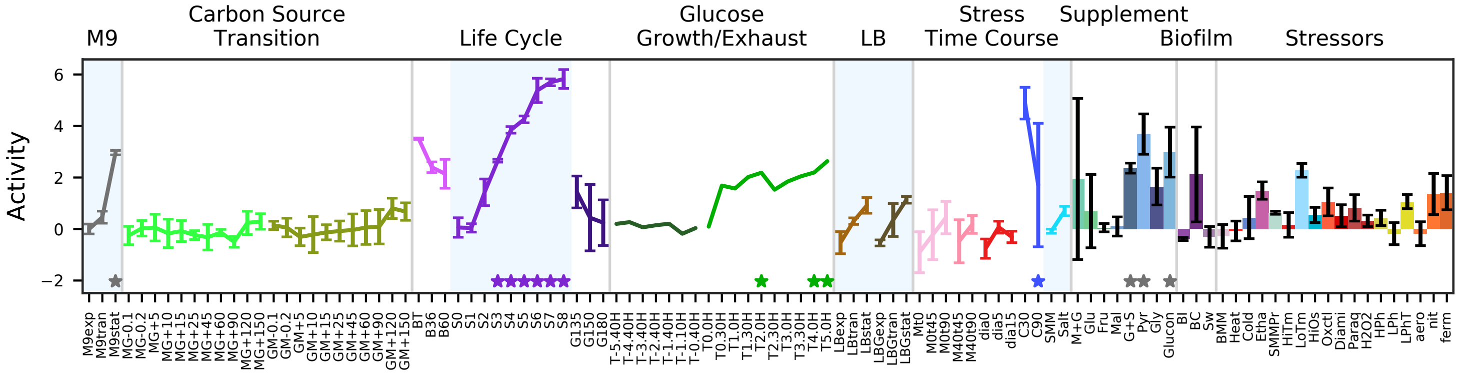

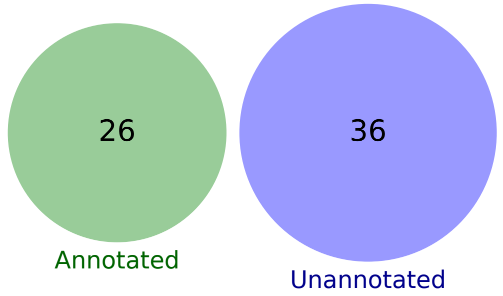

70 - Uncharacterized 5

Biological Function:
Contains many poorly characterized RNAs with noisy activity

No known regulator

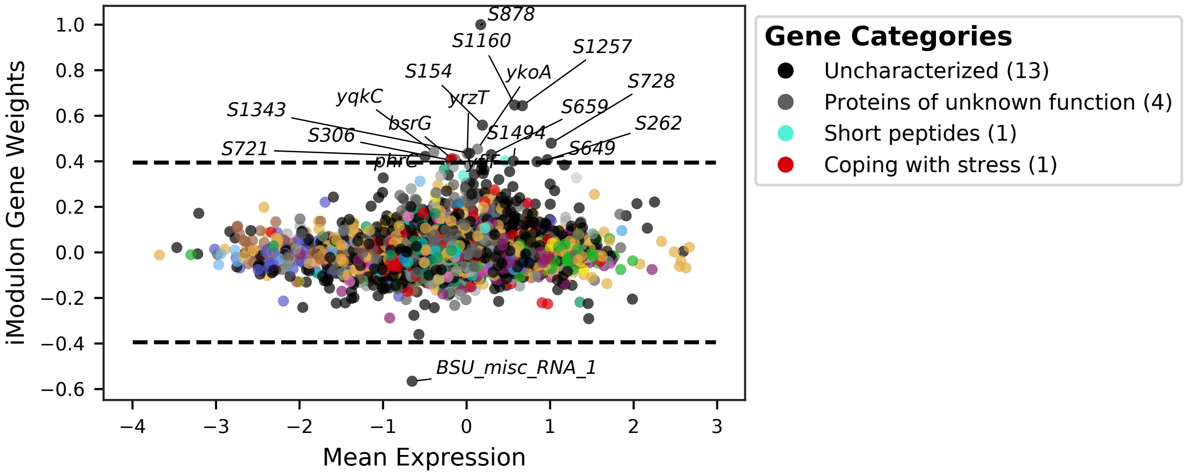

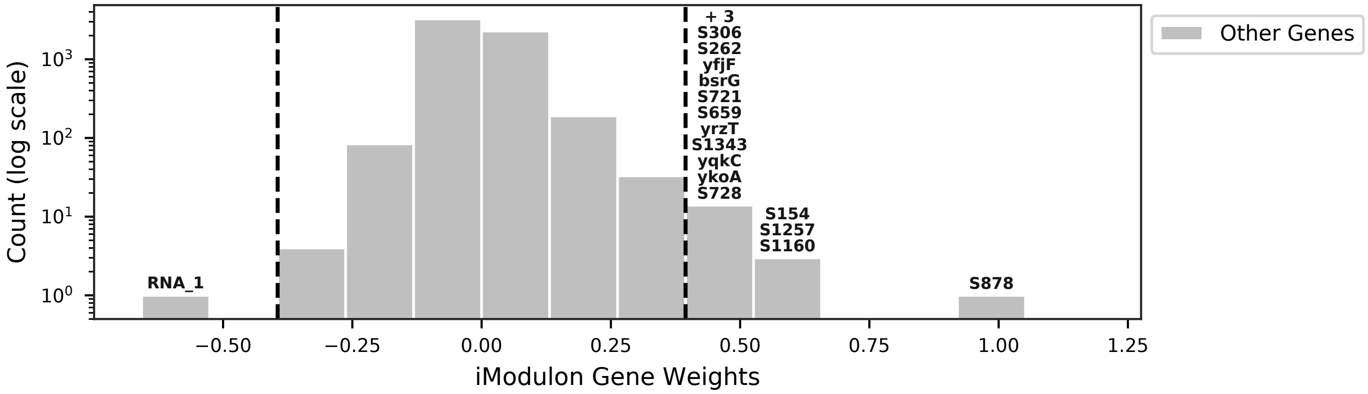

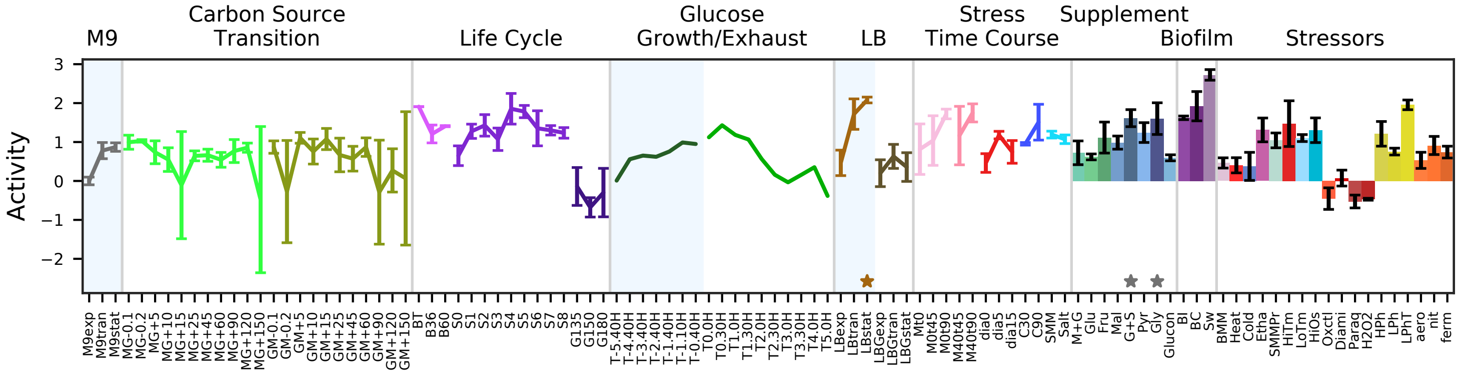

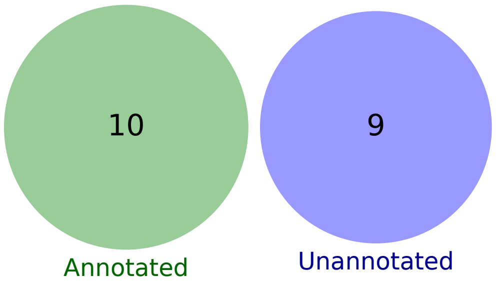

71 - Uncharacterized 6

Biological Function:
5’ ends of tRNAs and independent transcripts

No known regulator

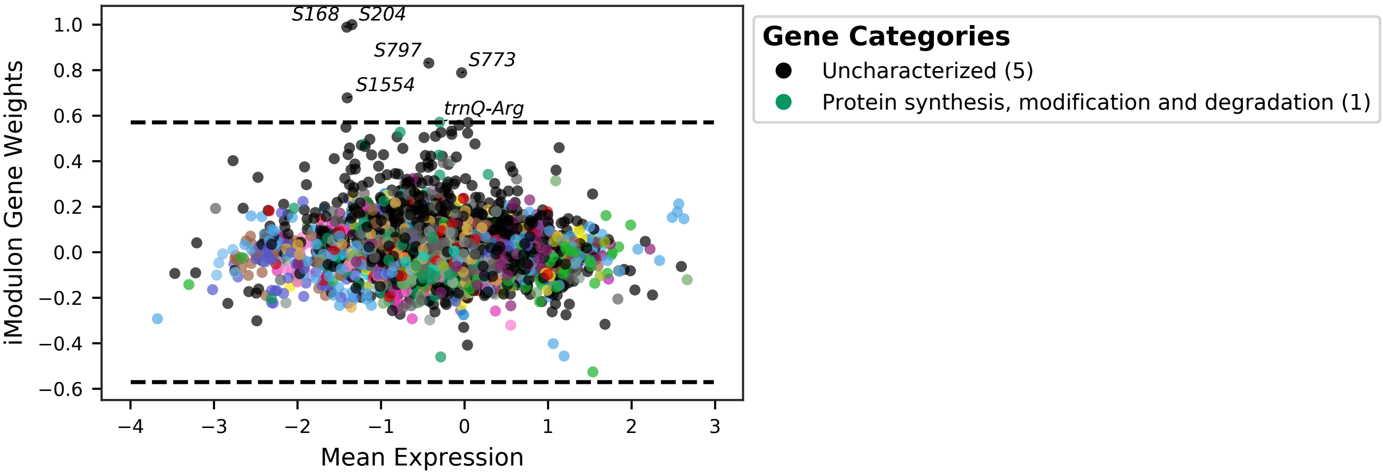

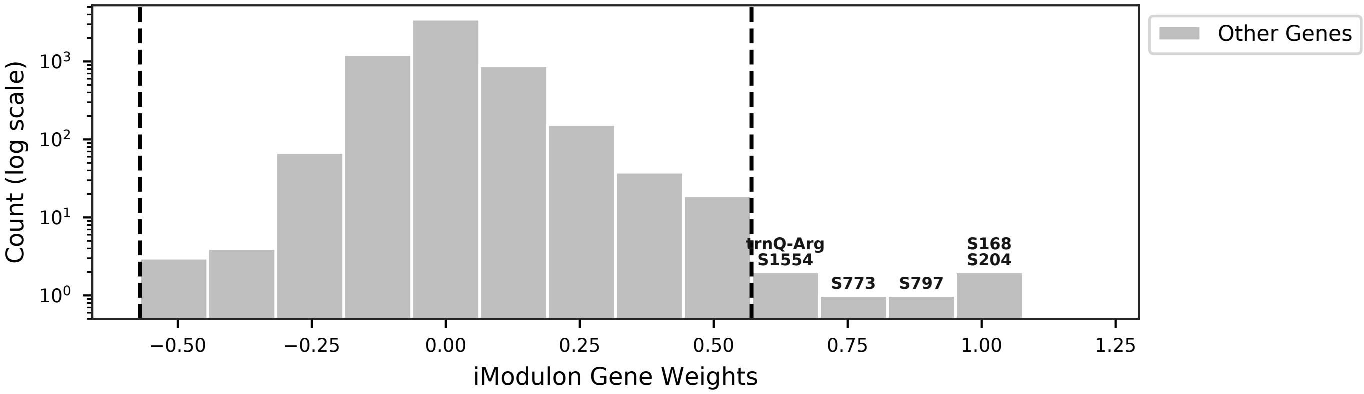

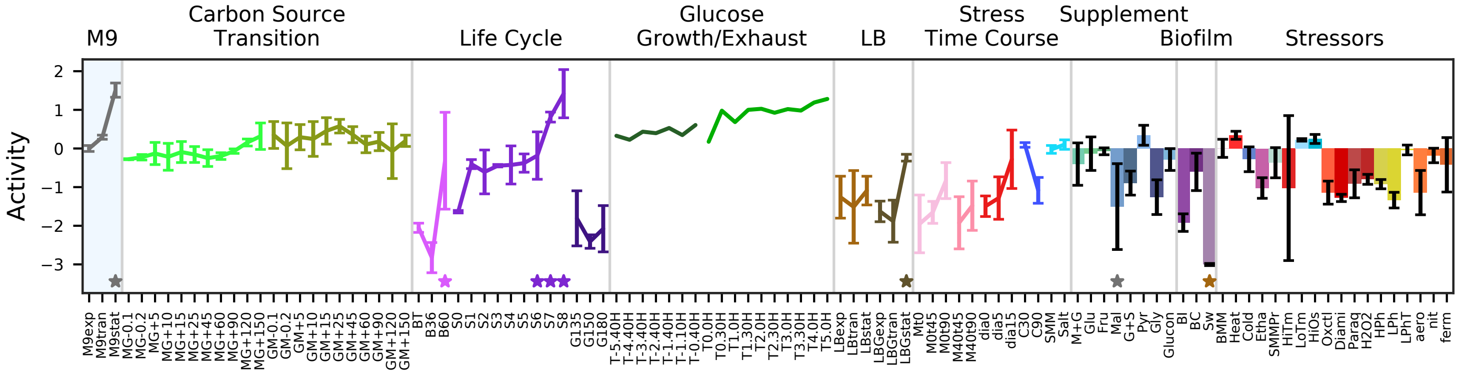

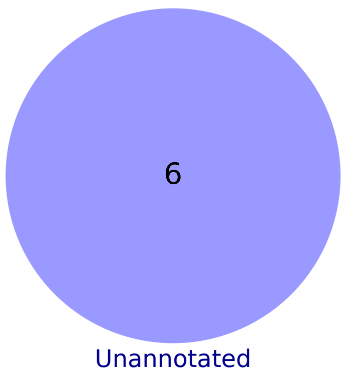

72 - Noise – S144

Biological Function:
Likely accounting for noisey RNA S144, an independent transcript

No known regulator

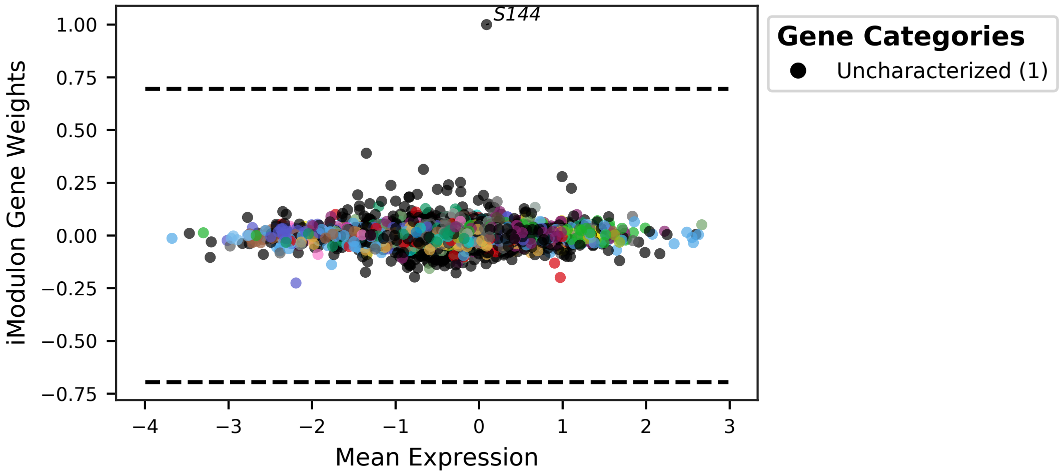

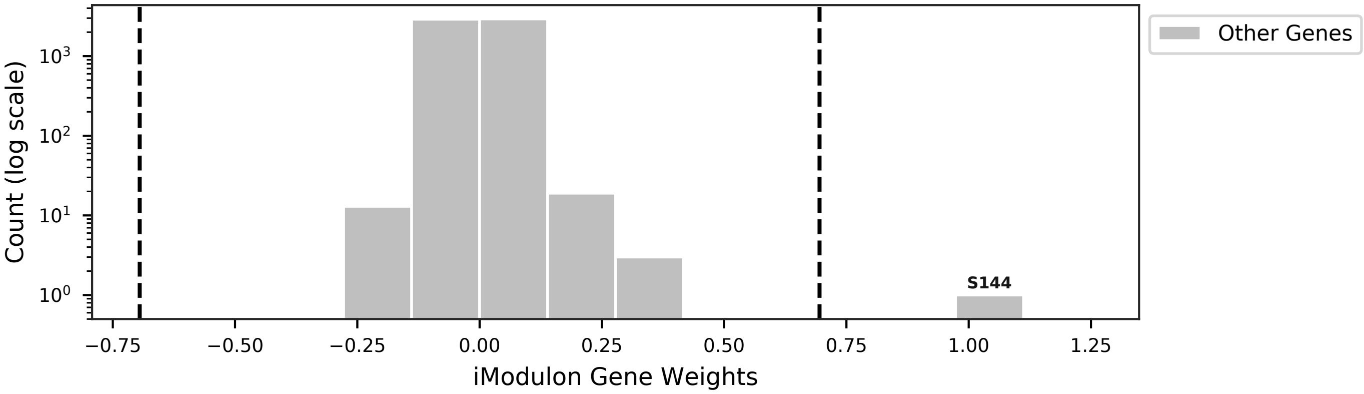

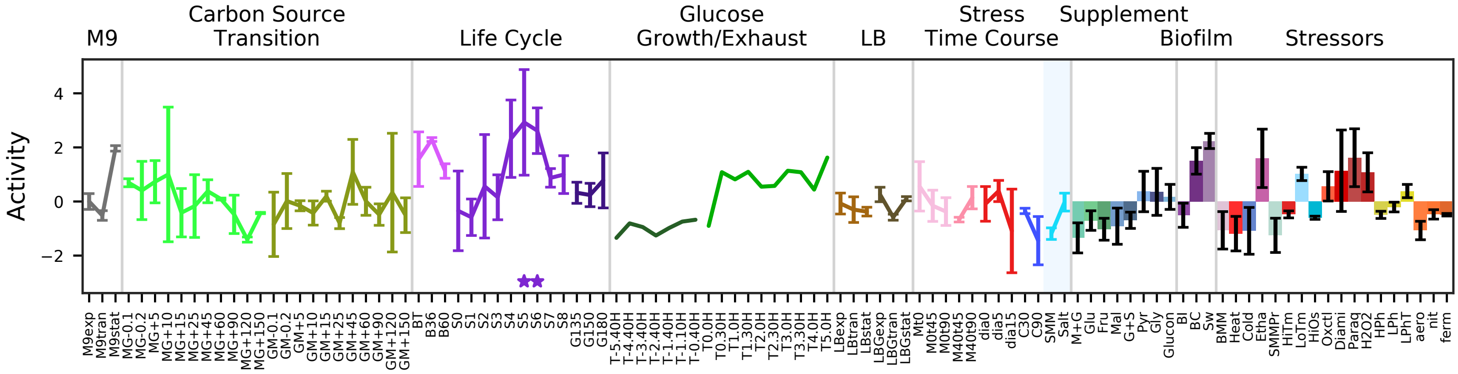

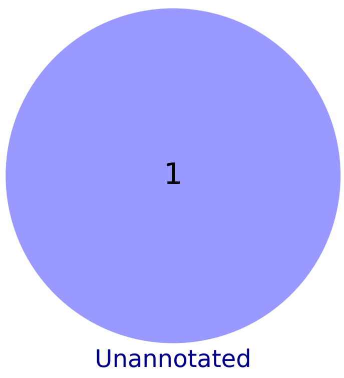

73 - Noise – S415

Biological Function:
Noisy independent transcript S415

No known regulator

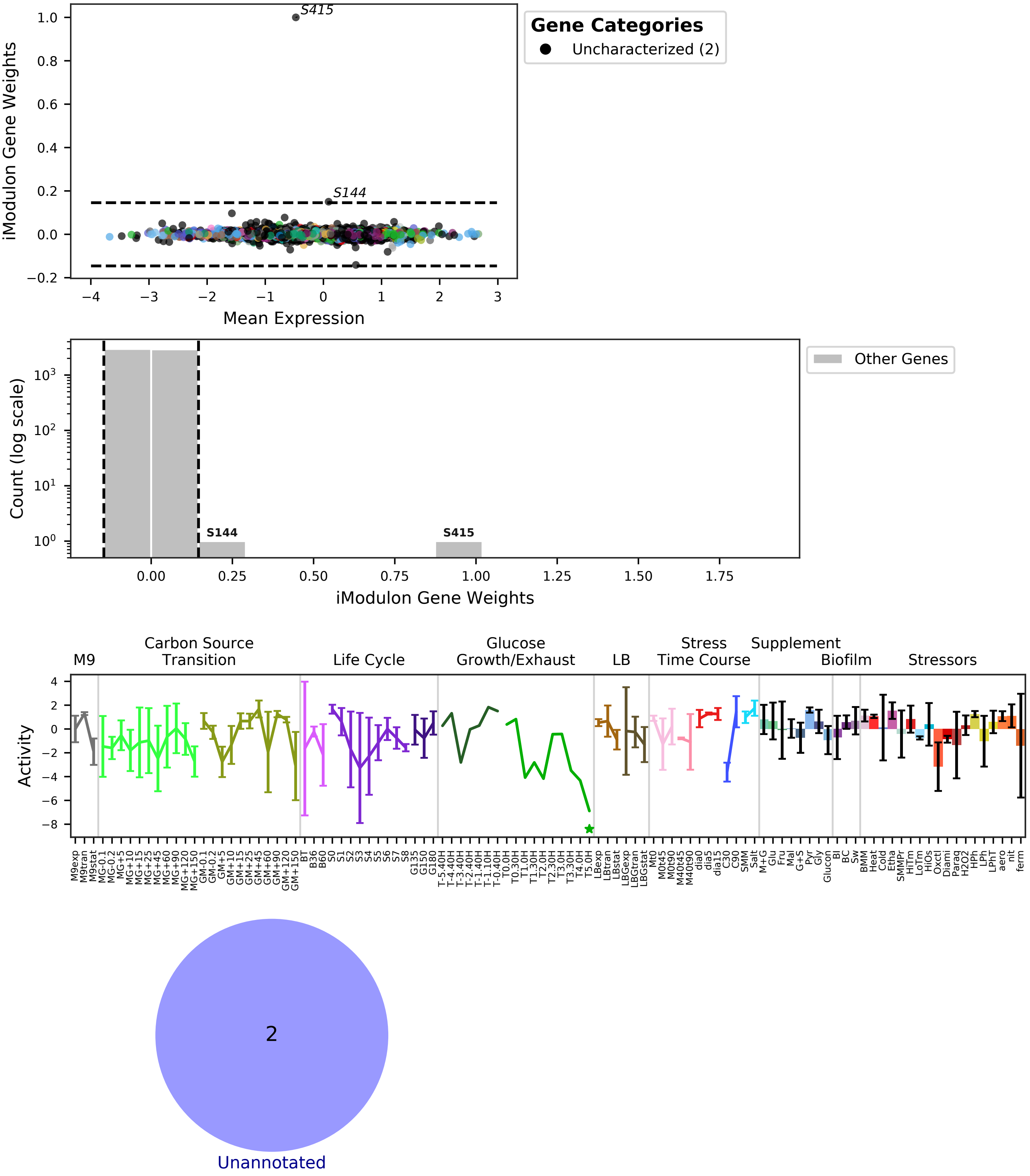

74 - Noise – S1235

Biological Function:
Independent transcript S1235, behaves similar to late sporulation iModulons

No known regulator

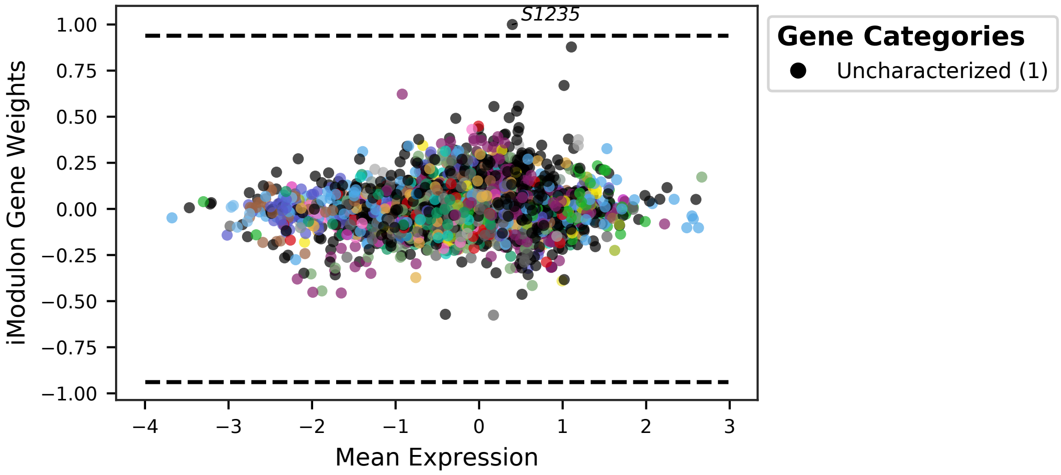

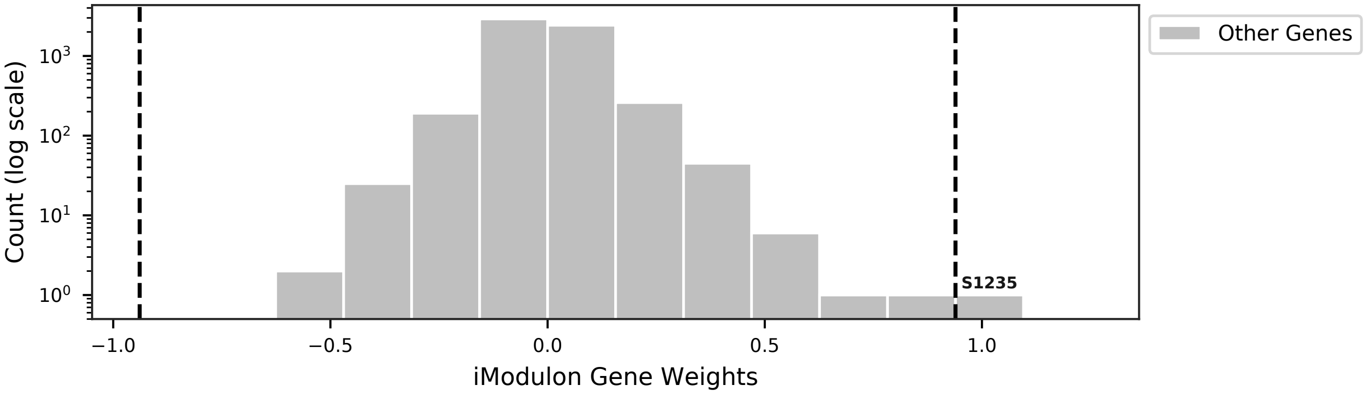

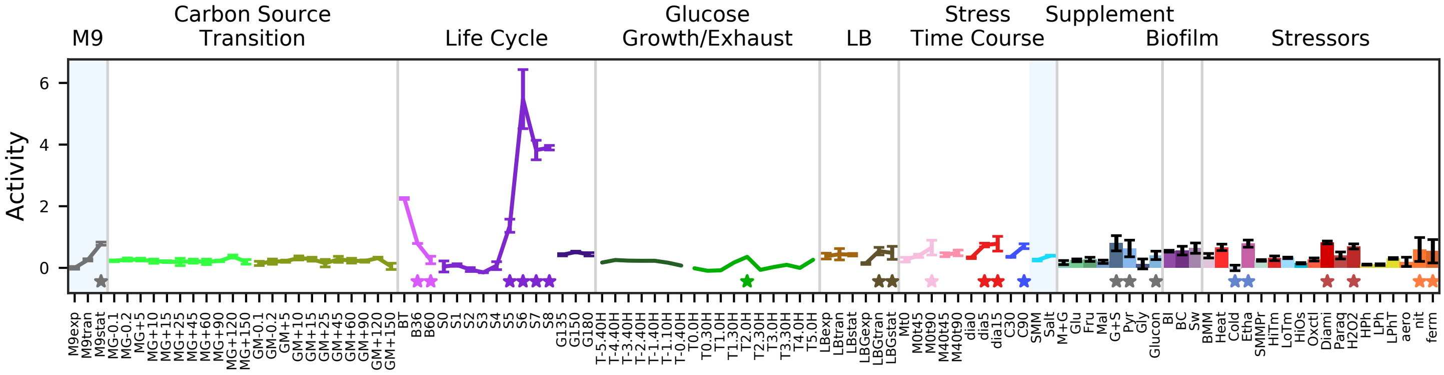

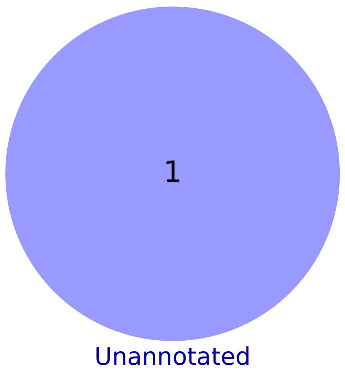

75 - Noise – S1210

Biological Function:
Noisy independent transcript S1210

No known regulator

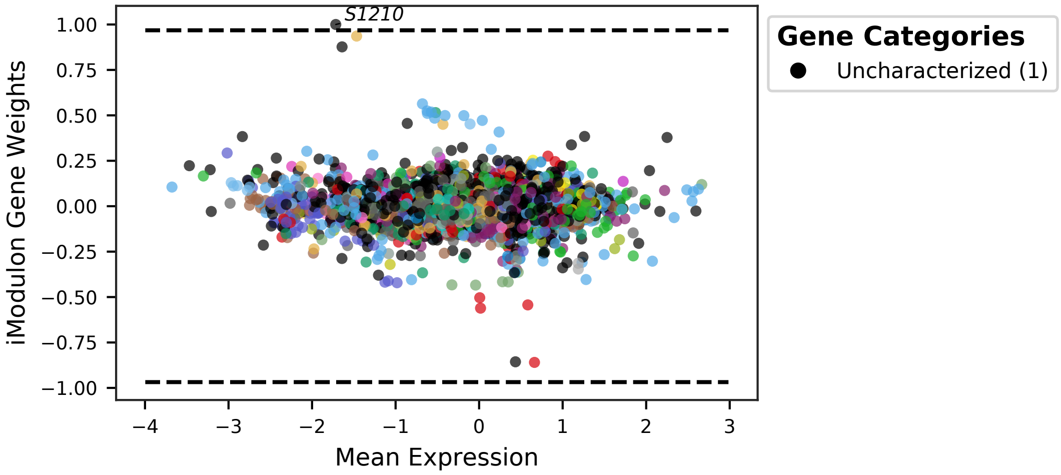

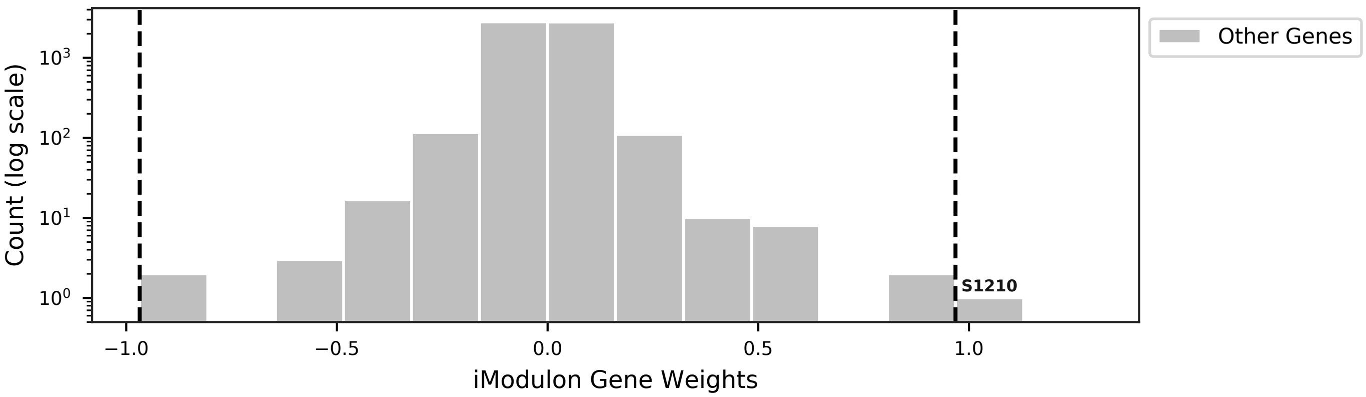

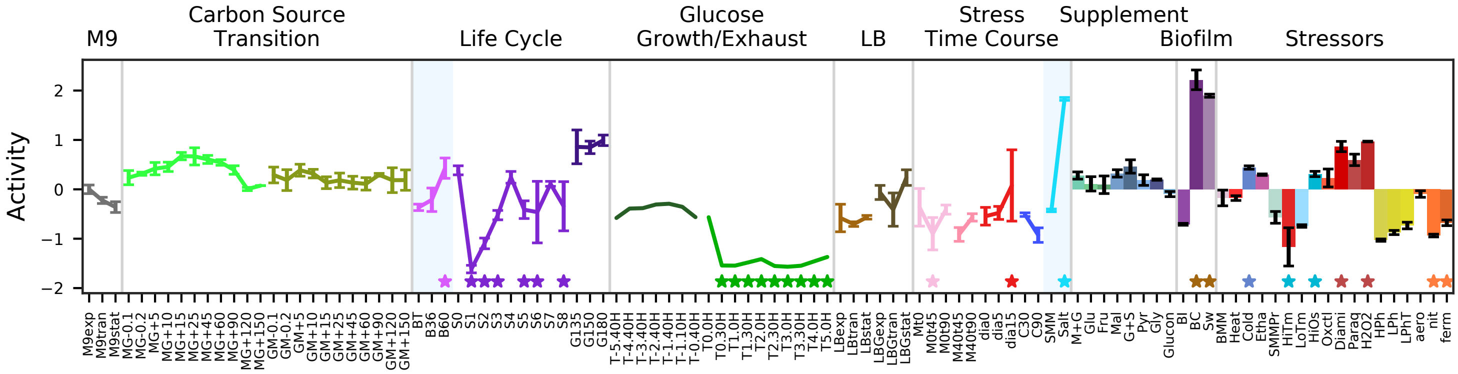

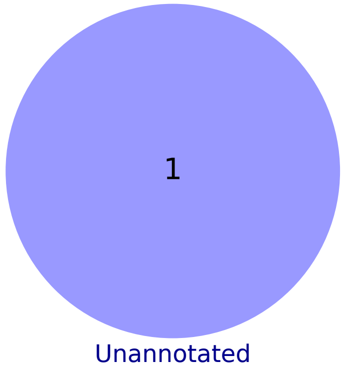

76 - Noise – Carbon Source Transition 1

Biological Function:
Likely accounting for noise in the carbon source transition experiments

No known regulator

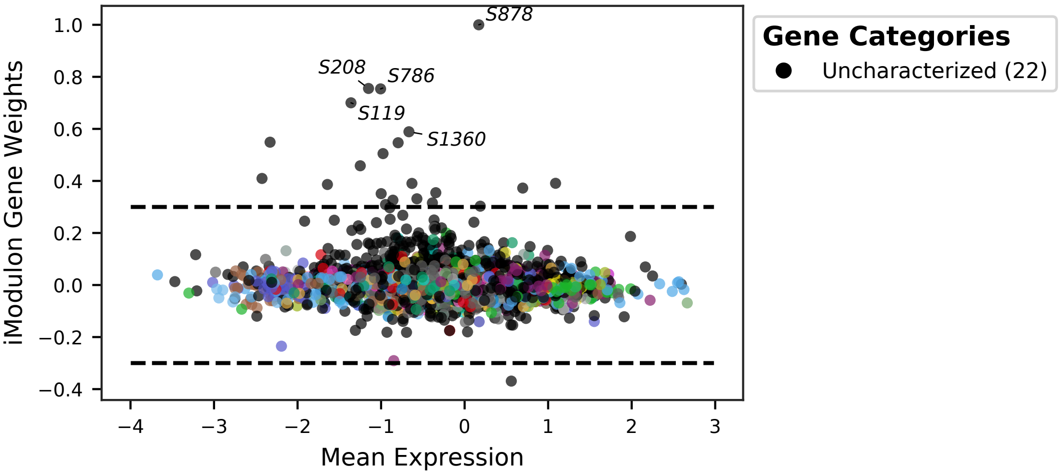

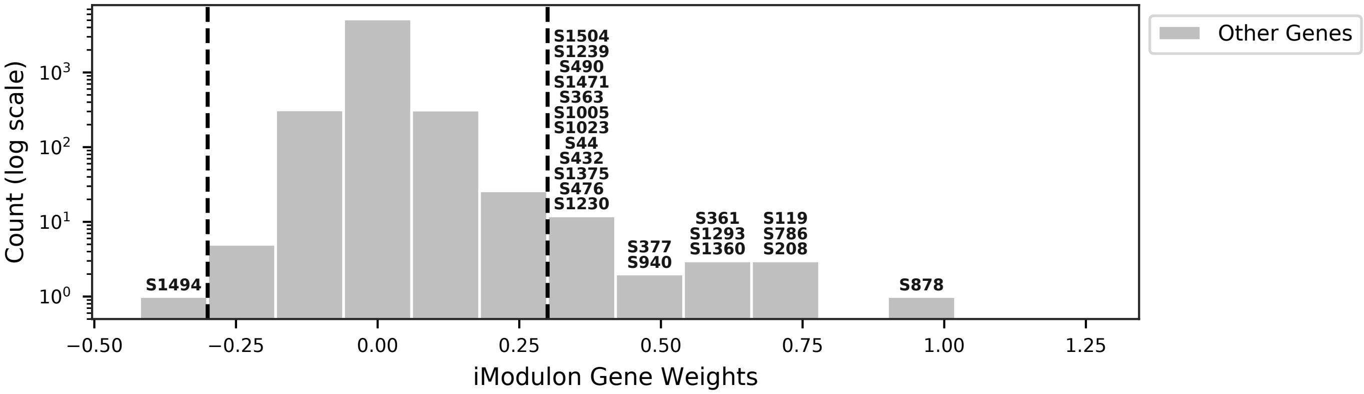

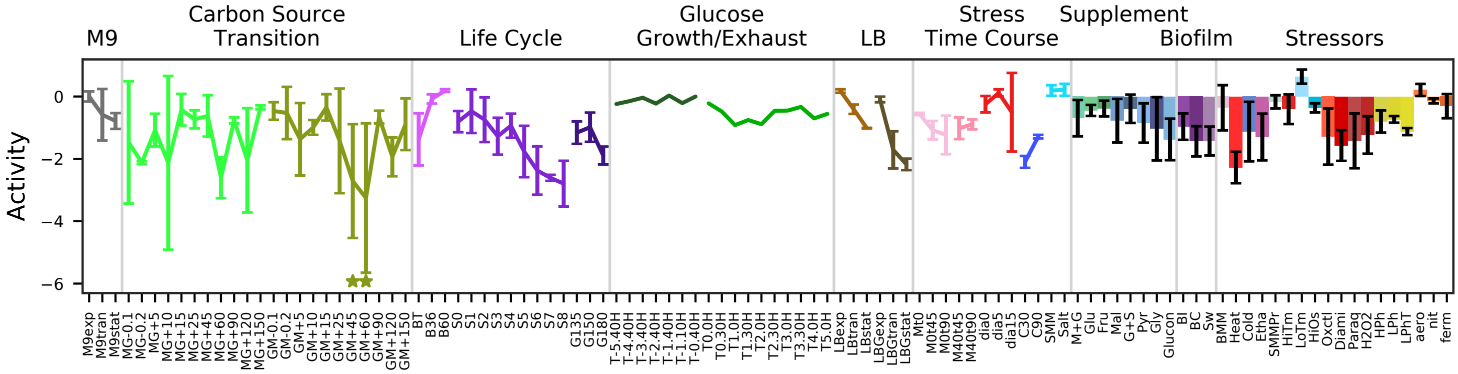

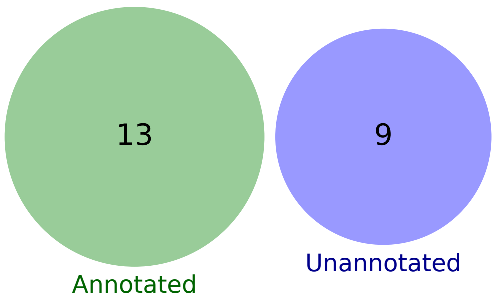

77 - Noise – Carbon Source Transition 2

Biological Function:
Likely accounting for noise in carbon source transition and gluconnate experiments

No known regulator

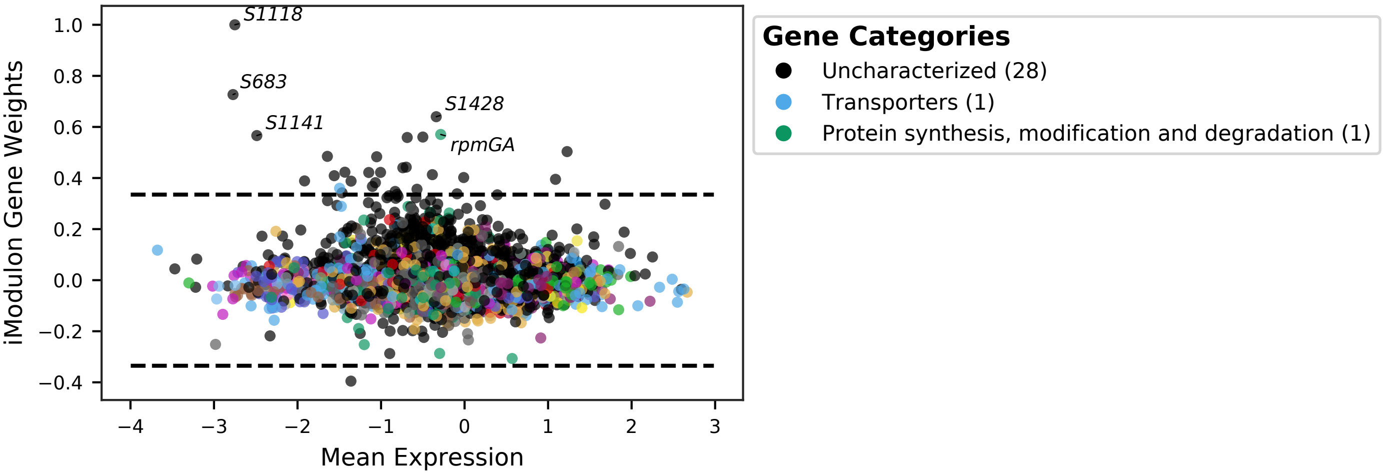

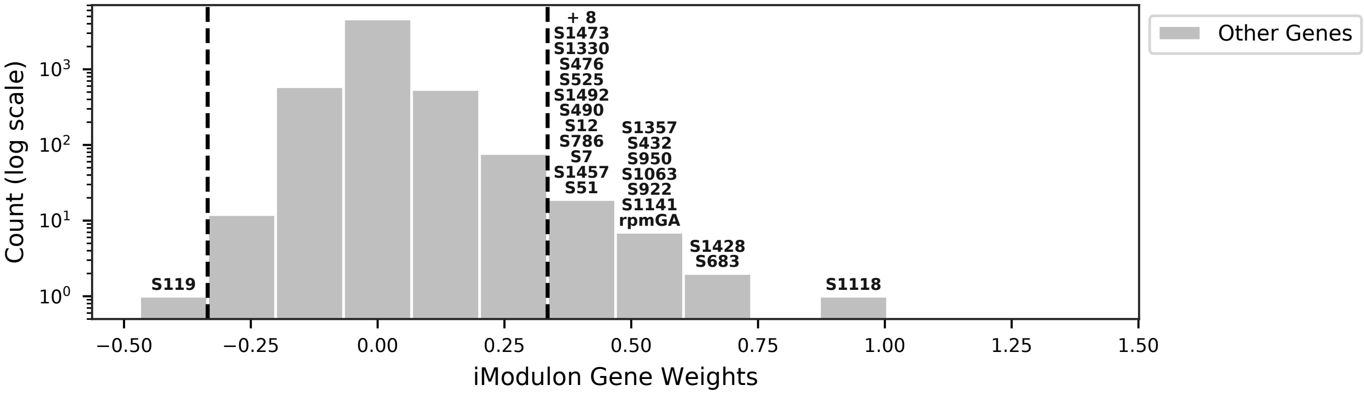

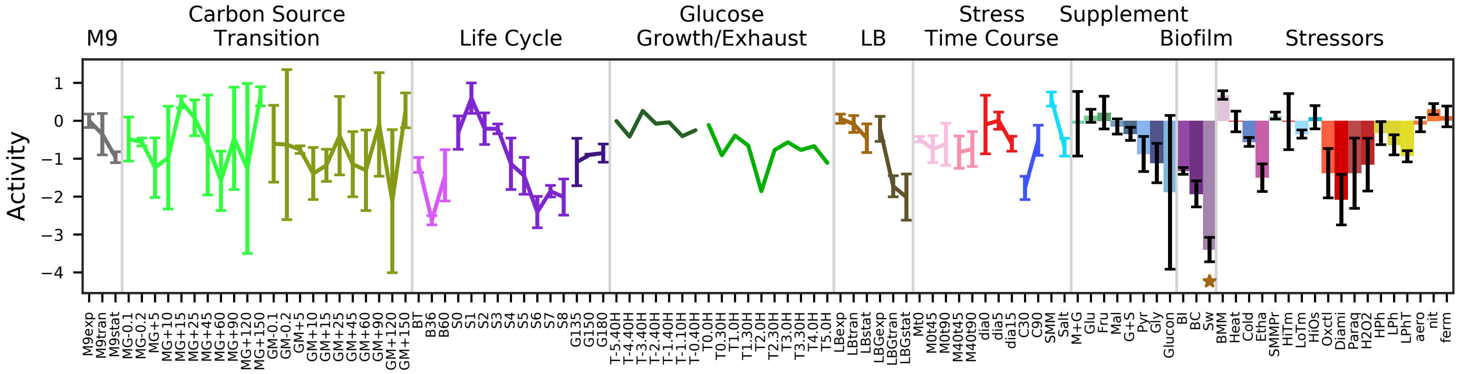

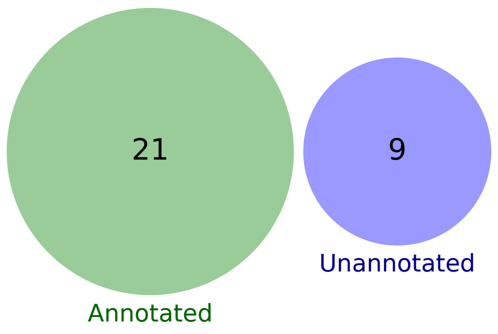

78 - Noise – LBGexp_1

Biological Function:
Likely accounting for noise in the first sample of LBG_exp. Top few RNAs are 5' UTRs of glucose metabolism genes

No known regulator

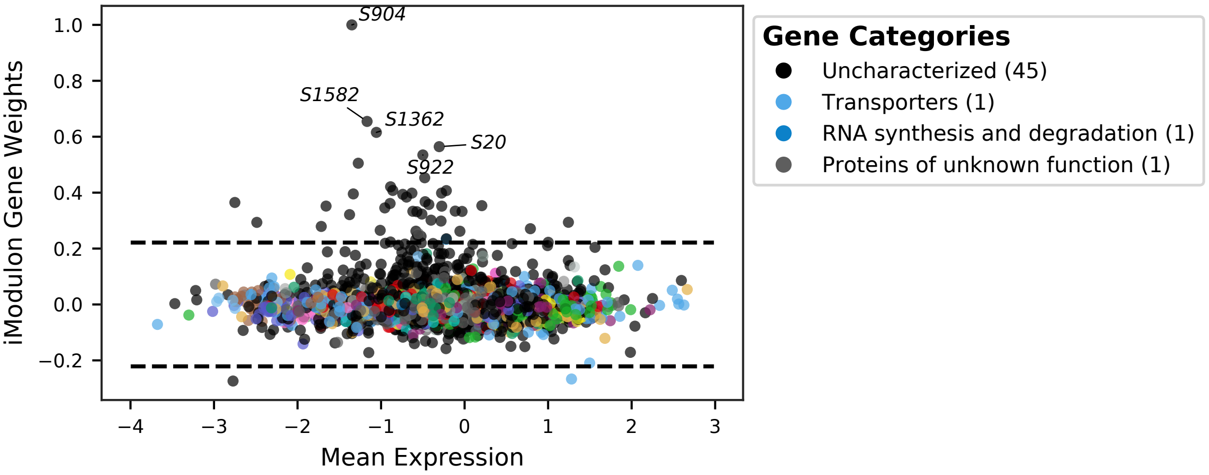

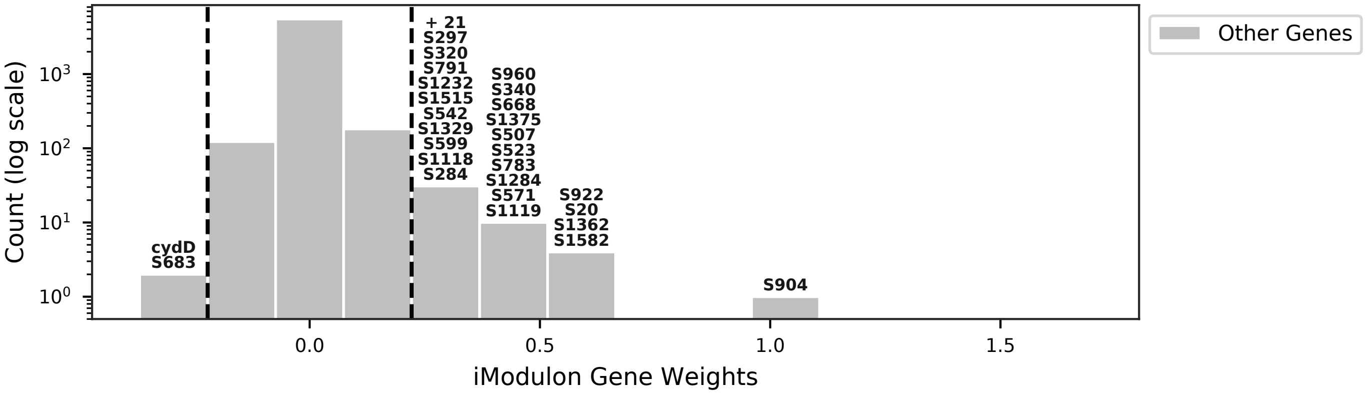

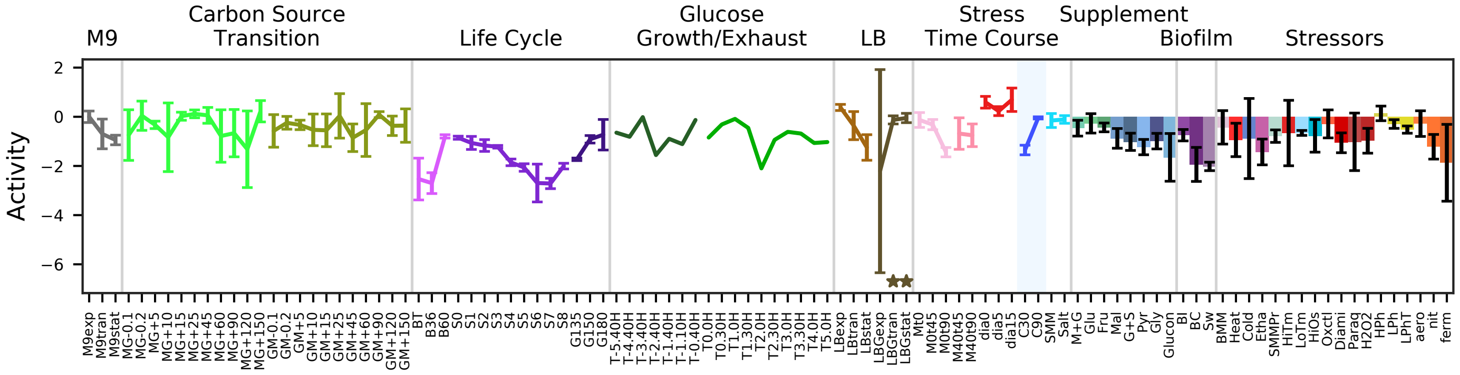

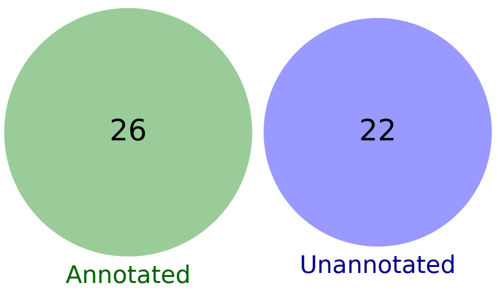

79 - Noise – Diamide 15

Biological Function:
Likely accounting for noise in the diamide after 15 minutes and LBG exponential conditions

No known regulator

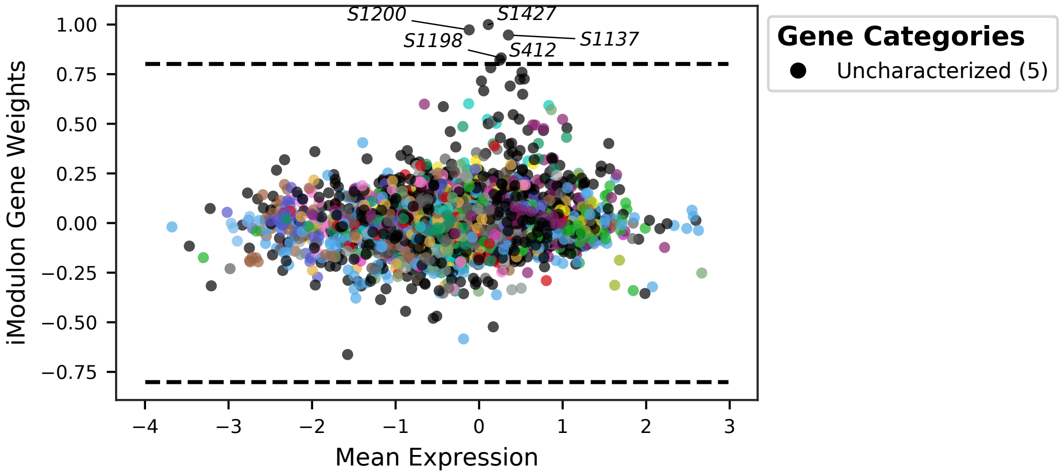

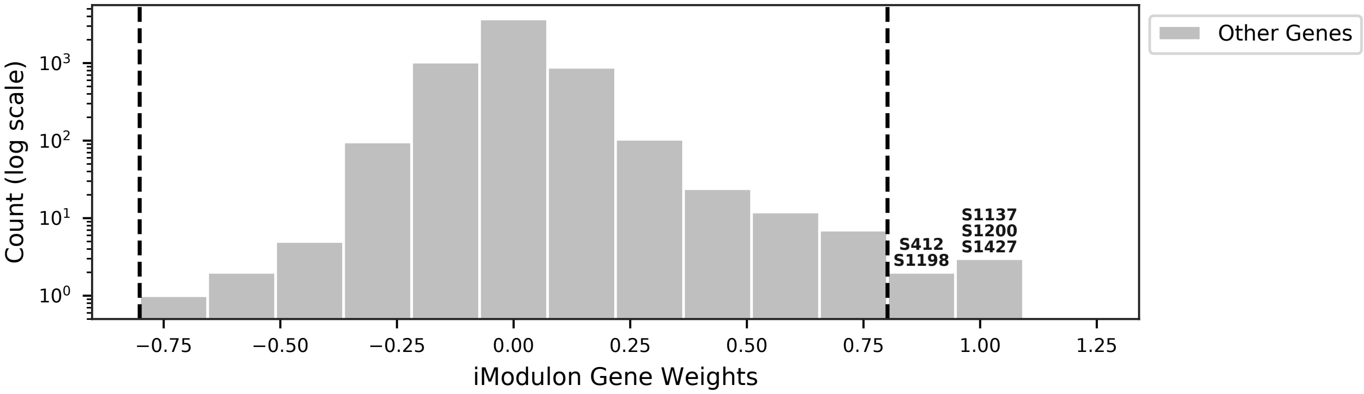

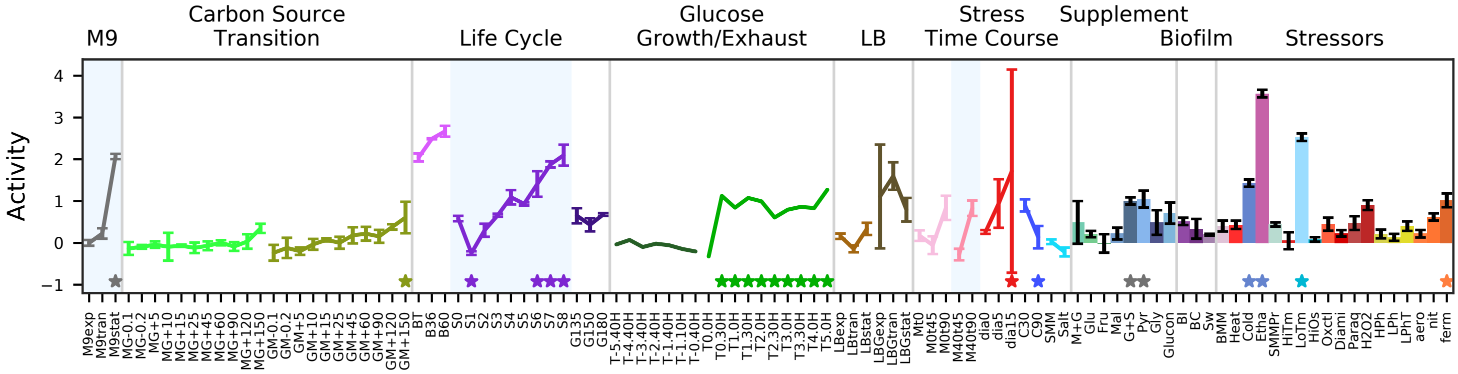

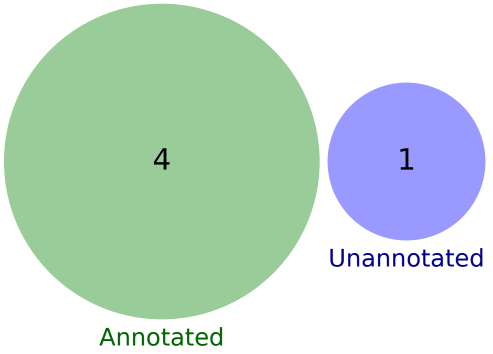

80 - Empty 1

Biological Function:
Contains no genes

No known regulator

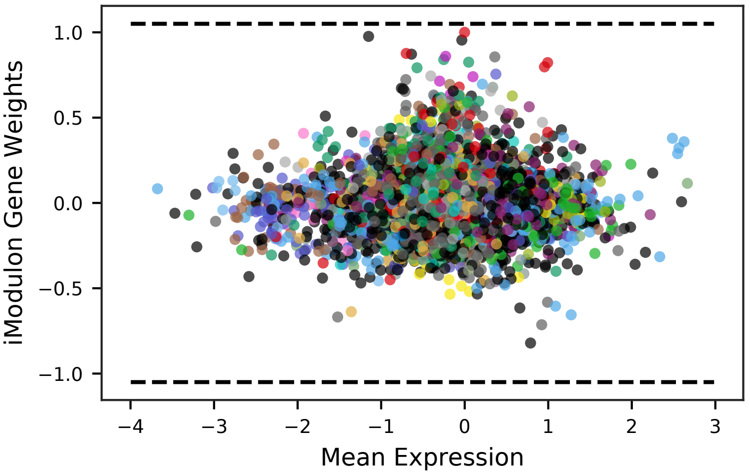

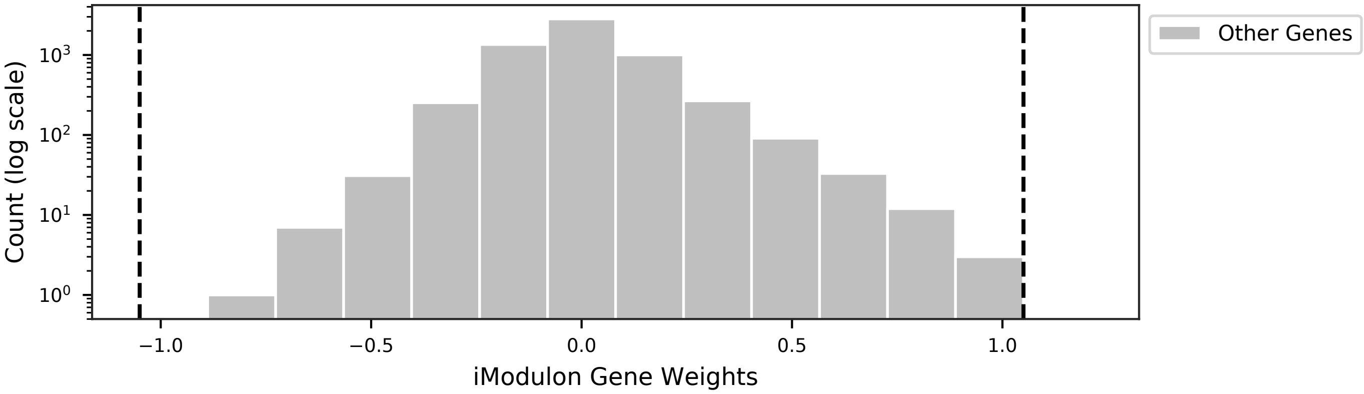

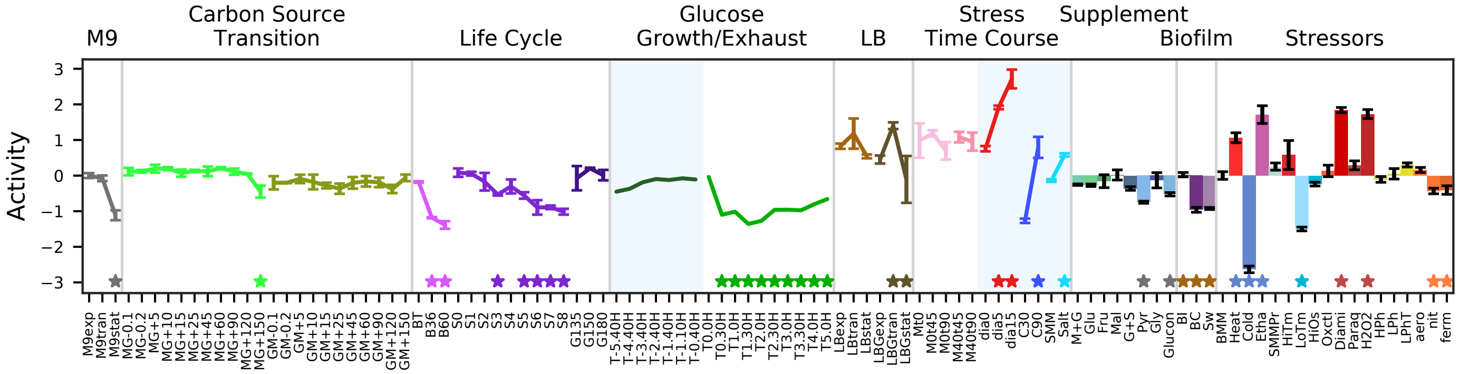

81 - Empty 2

Biological Function:
Contains no genes

No known regulator

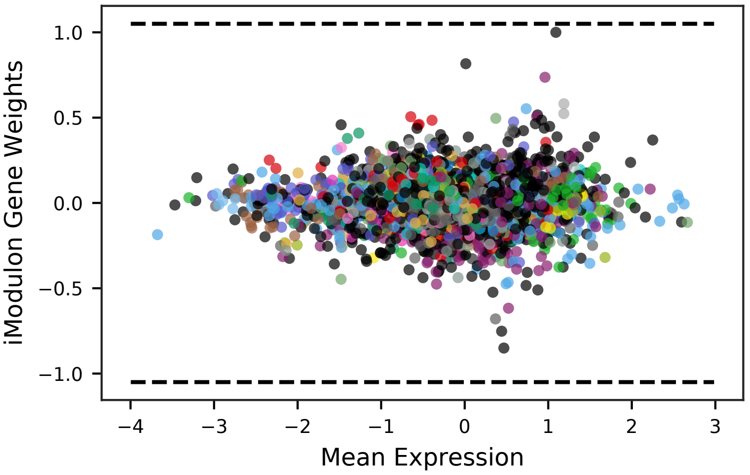

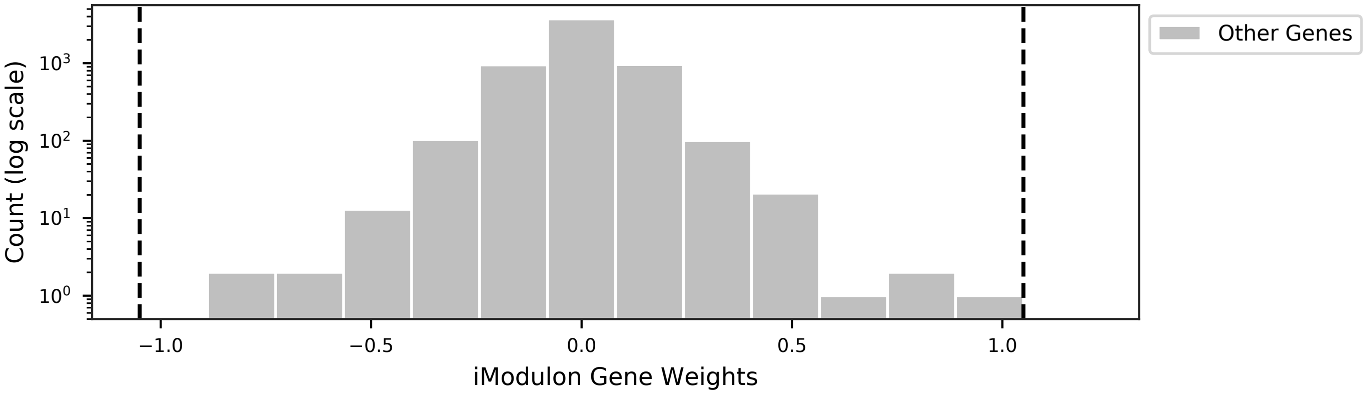

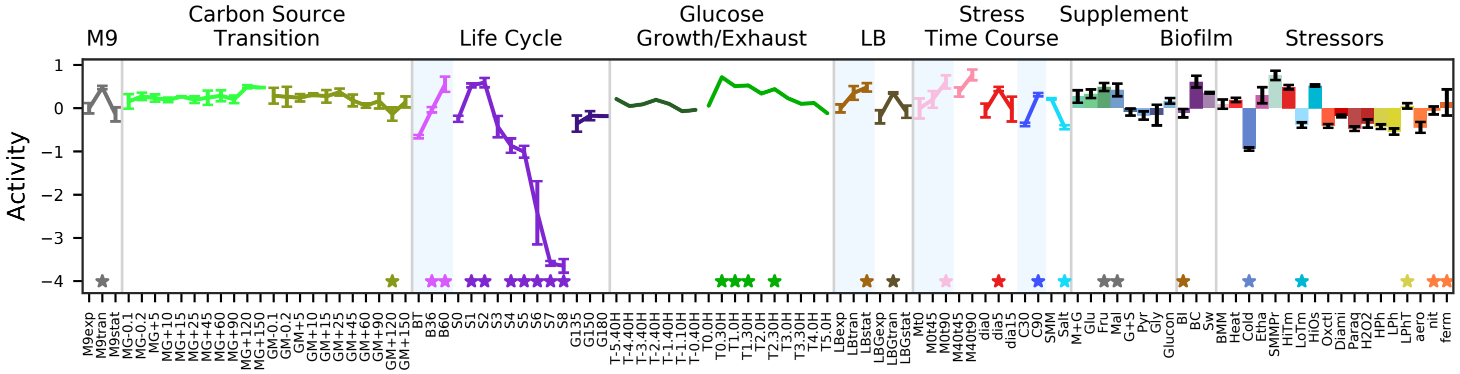

82 - Empty 3

Biological Function:
Contains no genes

No known regulator

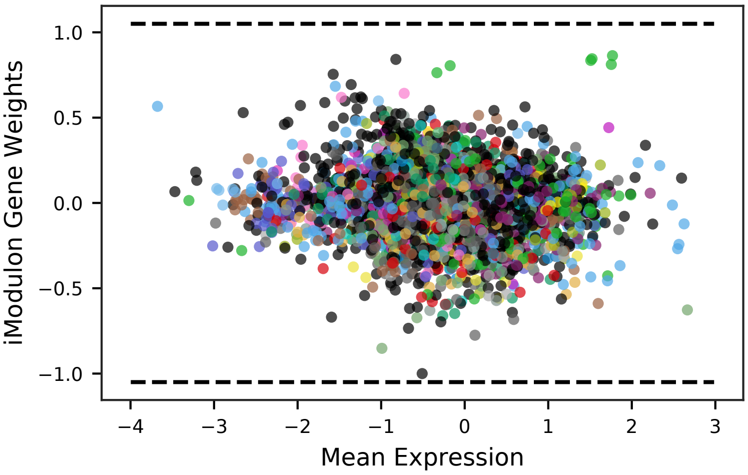

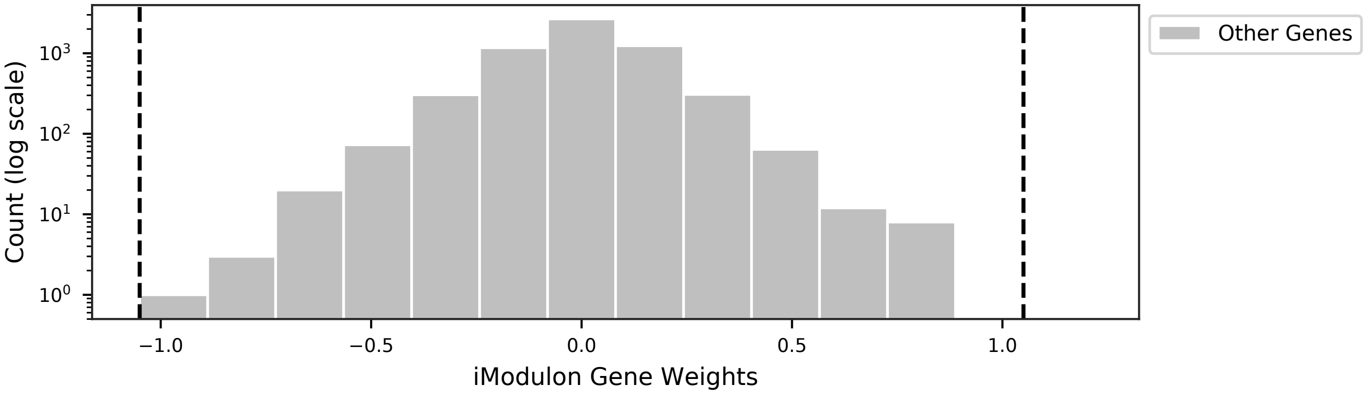

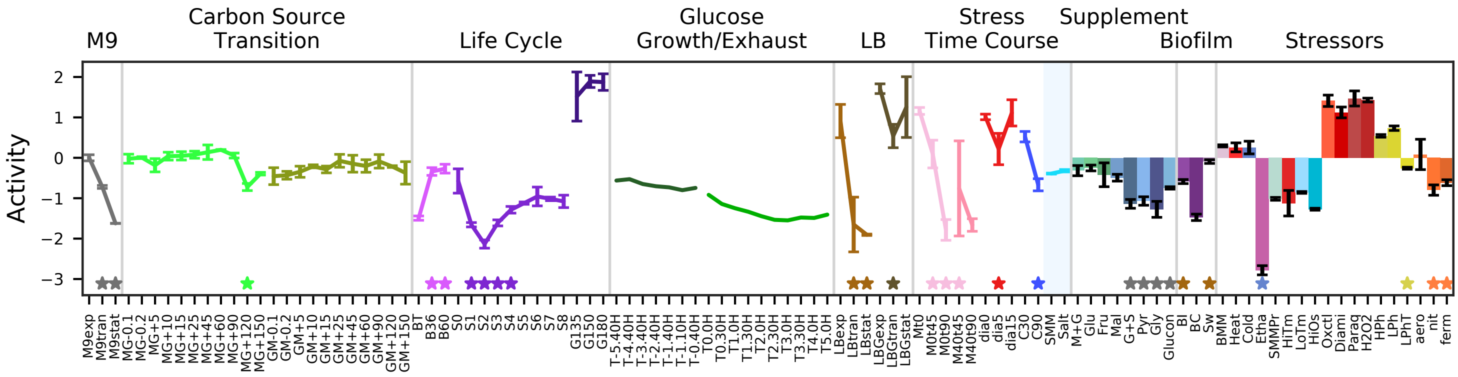

Supplement: Supplementary file 10 — Supplementary Data 11 [file 41467_2020_20153_MOESM10_ESM.pdf]
